# A shared threat-anticipation circuit is dynamically engaged at different moments by certain and uncertain threat

**DOI:** 10.1101/2024.07.10.602972

**Published:** 2024-08-12

**Authors:** Brian R. Cornwell, Paige R. Didier, Shannon E. Grogans, Allegra S. Anderson, Samiha Islam, Hyung Cho Kim, Manuel Kuhn, Rachael M. Tillman, Juyoen Hur, Zachary S. Scott, Andrew S. Fox, Kathryn A. DeYoung, Jason F. Smith, Alexander J. Shackman

**Affiliations:** 1 Department of Psychological & Brain Sciences, George Washington University, Washington, DC 20006 USA.; 2 Department of Psychology, University of Maryland, College Park, MD 20742 USA.; 3 Department of Neuroscience and Cognitive Science Program, University of Maryland, College Park, MD 20742 USA.; 4 Department of Maryland Neuroimaging Center, University of Maryland, College Park, MD 20742 USA.; 5 Department of Psychiatry and Human Behavior, Brown University, Providence, RI 02912 USA.; 6 Department of Psychology, University of Pennsylvania, Philadelphia, PA USA.; 7 Center for Depression, Anxiety and Stress Research, McLean Hospital, Harvard Medical School, Belmont, MA 02478 USA.; 8 McGill Neuropsychology, Bethesda, MD 20814 USA.; 9 Department of Psychology, Yonsei University, Seoul 03722, Republic of Korea.; 10 Department of Psychology, University of California, Davis, CA 95616 USA.; 11 California National Primate Research Center, University of California, Davis, CA 95616 USA.

**Keywords:** affective neuroscience, fear and anxiety, bed nucleus of the stria terminalis (BST/BNST), central extended amygdala (EAc), Research Domain Criteria (RDoC)

## Abstract

Temporal dynamics play a central role in models of emotion: *“fear”* is widely conceptualized as a phasic response to certain-and-imminent danger, whereas *“anxiety”* is a sustained response to uncertain-or-distal harm. Yet the underlying human neurobiology remains contentious. Leveraging an ethnoracially diverse sample, translationally relevant paradigm, and theory-driven modeling approach, we demonstrate that certain and uncertain threat recruit a shared threat-anticipation circuit. This cortico-subcortical circuit exhibits persistently elevated activation when anticipating uncertain-threat encounters and a transient burst of activation in the moments before certain encounters. For many scientists and clinicians, feelings are the defining feature of human fear and anxiety. Here we used an independently validated brain signature to covertly decode the momentary dynamics of anticipatory distress for the first time. Results mirrored the dynamics of neural activation. These observations provide fresh insights into the neurobiology of threat-elicited emotions and set the stage for more ambitious clinical and mechanistic research.

## INTRODUCTION

Fear and anxiety are evolutionarily conserved features of mammalian life that help protect us from harm^1^. But when expressed too strongly or pervasively, they can be crippling^2, 3^. Anxiety-related disorders impose a staggering burden on global public health, afflicting ~360 million individuals annually^4, 5^. In the U.S., ~1 in 3 individuals will experience a lifetime disorder, service utilization is surging, and annual healthcare costs exceed $40B, drawing the attention of the U.S. Surgeon General, White House, and other top policymakers^6–11^. Existing treatments were developed decades ago and have limited effectiveness, durability, and tolerability, underscoring the need to clarify the underlying neurobiology^12–15^.

Temporal dynamics play a central role in conceptual models of emotion, with many theorists and clinicians conceptualizing *“fear”* as a phasic response to certain-and-imminent danger and *“anxiety”* as a sustained response to uncertain-or-distal harm^1, 16–24^. Work harnessing the millisecond resolution of psychophysiological measures supports this view, showing that human defensive behaviors exhibit specific temporal patterns across different threat contexts^20, 25–31^. When the timing of threat encounters is uncertain, a sustained state of heightened reactivity is evident. In contrast, when encounters are certain and imminent, a transient burst of heightened defensive responding is triggered. Both effects are consistent with the results of behavioral studies in rodents^32–34^. Among humans, long-duration certain-threat cues, with explicit ‘count-down’ signals, elicit more complex dynamics, marked by a rapid initial rise in defensive responding, followed by a sustained level of elevated reactivity during the intermediate period, and a phasic surge just prior to the threat encounter (‘surge-trough-surge’).

Human neuroimaging studies have begun to reveal the broad contours of the neural circuitry recruited by certain and uncertain threat^35^, but have yet to plumb the moment-by-moment neural dynamics anticipated by theory and psychophysiological research. Most fMRI studies have relied on oversimplified ‘boxcar’ modeling approaches that assume static, time-invariant neural responses to anticipated threat encounters.

We and others have begun to explore finer-grained models for descriptive purposes, but have yet to use them for rigorous hypothesis testing^36, 37^. Consequently, it remains unclear whether phasic (*“fear”*) and sustained (*“anxiety”*) neural responses to threat are segregated into dissociable anatomical systems, as some have posited^1, 16, 22, 38–44^, or are co-localized to a singular system that shows distinctive activation dynamics across different threat contexts, as we have hypothesized^35, 36, 45, 46^.

**Figure 1.**
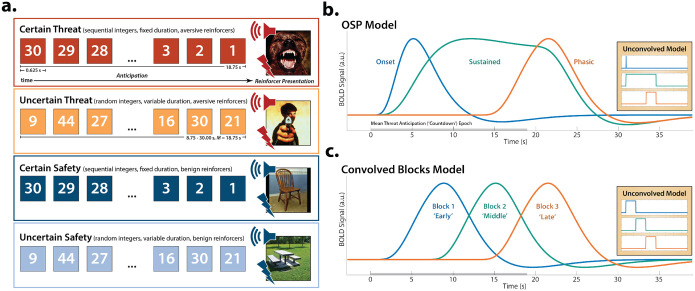
Conceptual overview. **a. *Threat-anticipation paradigm*.** The Maryland Threat Countdown (MTC) takes the form of a 2 (*Valence:* Threat, Safety) × 2 (*Temporal Certainty:* Certain, Uncertain) randomized event-related design. On certain-threat trials, participants saw a descending stream of integers or ‘countdown’ for 18.75 s. To ensure robust fear and anxiety, this period of anticipation always terminated with the presentation of a noxious electric shock, unpleasant picture, and thematically related audio clip (e.g., scream). Uncertain-threat trials were similar, but the integer stream was randomized and presented for an uncertain and variable duration (8.75–30.00 s; *M*=18.75 s). Here, participants knew that something aversive was going to occur, but they had no way of knowing precisely when. Safety trials were similar but terminated with the delivery of neutral reinforcers (e.g., just-perceptible electrical stimulation). **b. *OSP model*.** Prior research has relied on oversimplified ‘boxcar’ modeling approaches that reduce anticipatory neural dynamics to a single average response^53^. Here, we used two complementary hemodynamic modeling approaches to interrogate time-varying responses to certain and uncertain threat. The OSP model used multiple-regression to identify the variance in threat-anticipation signals uniquely associated with temporally overlapping Onset, Sustained, and Phasic regressors. The design matrix incorporated a punctate event time-locked to the onset of the anticipation epoch, a variable-duration rectangular function spanning the entirety of the anticipation epoch (to capture sustained increases in activation), and a temporally overlapping rectangular function time-locked to the offset of the anticipation epoch (to capture phasic surges in activation to acute threat). Note that conventional boxcar models are equivalent to the green signal. **c. *Convolved Blocks Model*.** To clarify interpretation, we employed a piecewise model that splits the anticipation epoch into a sequence of short (6.25 s), non-overlapping rectangular functions (‘blocks’), each convolved with a canonical hemodynamic response function. While arbitrary in timing, the Convolved Blocks model yields activation estimates that are independent, inferentially intuitive, and statistically comparable across time, enabling us to more fully assess apparent surges in activation in the moments just before certain-threat encounters. Abbreviations—a.u., arbitrary units; BOLD, blood-oxygenation-level-dependent; HRF, hemodynamic response function; OSP, Onset-Sustained-Phasic; s, seconds.

To address this fundamental question, we used a novel combination of fMRI techniques—including theory-driven hemodynamic modeling, focused region-of-interest (ROI) analyses, and multivoxel brain signature analyses—to interrogate the moment-by-moment dynamics of threat-elicited neural activity and subjective distress in 220 ethnoracially diverse adults. Data were collected using the Maryland Threat Countdown (MTC), a well-established paradigm for manipulating the temporal certainty of threat encounters (Figure 1a)^36, 47–49^. The MTC is an fMRI-optimized variant of threat assays that have been pharmacologically and psychophysiologically validated in rodents and humans^26, 50–52^, maximizing translational relevance. Prior work focused on this and other samples demonstrates that this paradigm robustly increases subjective symptoms of distress and objective signs of arousal, reinforcing its validity as an experimental probe of human fear and anxiety (Supplementary Figure S1)^36, 47–49^.

## RESULTS

### Conventional ‘boxcar’ modeling reveals a shared threat-anticipation circuit

The present sample of 220 datasets represents a superset of the 99 featured in an earlier report that relied on a conventional ‘boxcar’ modeling approach, older data-processing pipeline, and coarser spatial-smoothing kernel (6-mm)^36^. Here we used standard voxelwise GLMs to confirm that conventional modeling of the larger reprocessed dataset broadly reproduced our published results. As expected, results revealed significant activation during periods of uncertain-threat anticipation, both in subcortical regions implicated in rodent models of fear and anxiety—such as the periaqueductal gray (PAG), bed nucleus of the stria terminalis (BST), and dorsal amygdala—and in frontocortical regions that are especially well-developed in primates—including the midcingulate cortex (MCC), anterior insula/frontal operculum (AI/FrO), and rostral dorsolateral prefrontal cortex (dlPFC; FDR *q*<0.05, whole-brain corrected; Supplementary Figure S2 and Supplementary Tables S1–S5). The same pattern was evident during certain-threat anticipation, with overlapping voxels evident for both kinds of threat in each of these key regions. In short, when viewed through the macroscopic lens of conventional fMRI modeling, uncertain- and certain-threat anticipation engage co-localized neural circuits, suggesting a common neural substrate in humans.

### Sustained activation is evident during the both uncertain- and certain-threat anticipation

**Figure 2.**
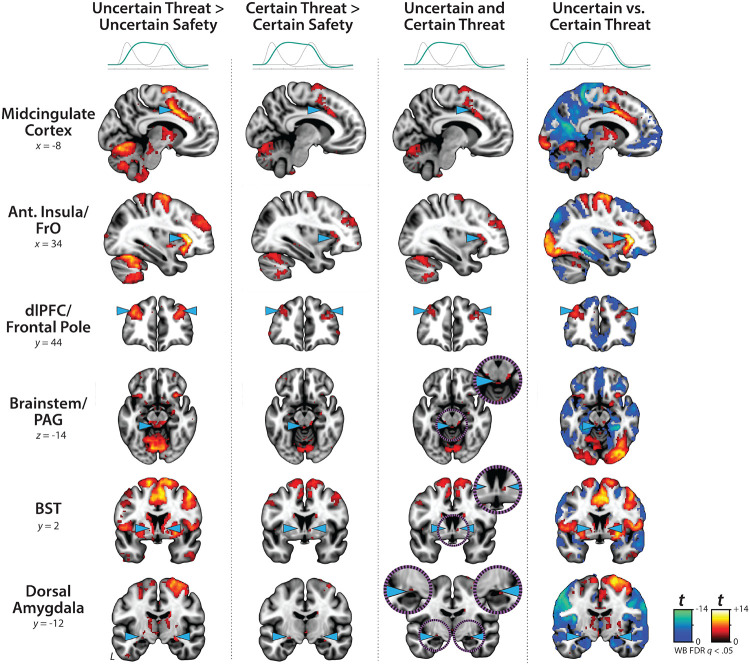
Sustained activation is evident during uncertain- and certain-threat anticipation. Key regions showing evidence of sustained hemodynamic activity during the anticipation of temporally uncertain threat (*first column*) and certain threat (*second column*) compared to their respective control conditions (FDR *q*<0.05, whole-brain corrected). A minimum-conjunction^54^ (logical ‘AND’) of the two contrasts revealed colocalization throughout the threat-anticipation circuit (*third column*). A direct contrast of the two contexts indicated that sustained signals were more pronounced during uncertain-threat anticipation (*fourth column*). Note: 4-mm smoothing kernel. Abbreviations—Ant., anterior; BST, bed nucleus of the stria terminalis; dlPFC, dorsolateral prefrontal cortex; FDR, false discovery rate; FrO, frontal operculum; L, left; PAG, periaqueductal gray; t, Student’s *t*-test; vs., versus; WB, whole-brain corrected.

While useful, conventional hemodynamic modeling approaches cannot resolve time-varying neural responses to anticipated threat encounters. To address this, we used a multiple-regression framework to transform the measured hemodynamic signal into a weighted linear combination of Onset, Sustained, and Phasic responses (Figure 1b and Supplementary Figure S3). Standard voxelwise GLMs were then used to identify regions showing sustained activation during the anticipation of uncertain and certain threat (FDR *q*<0.05, whole-brain corrected). Results closely resembled those yielded by conventional ‘boxcar’ analyses (Supplementary Figure S2), with sustained activation evident throughout the canonical threat-anticipation circuit^35^—including the BST and dorsal amygdala—during the anticipation of uncertain and certain threat (Figure 2; Supplementary Tables S6–S12). Despite this qualitative similarity, direct comparison of the two threats indicated that sustained responses were significantly stronger when the timing of threat encounters was uncertain (Figure 2).

### Phasic responses to certain-and-imminent threat are evident in the same regions that show sustained responses to uncertain threat, indicating a shared threat-anticipation circuit

**Figure 3.**
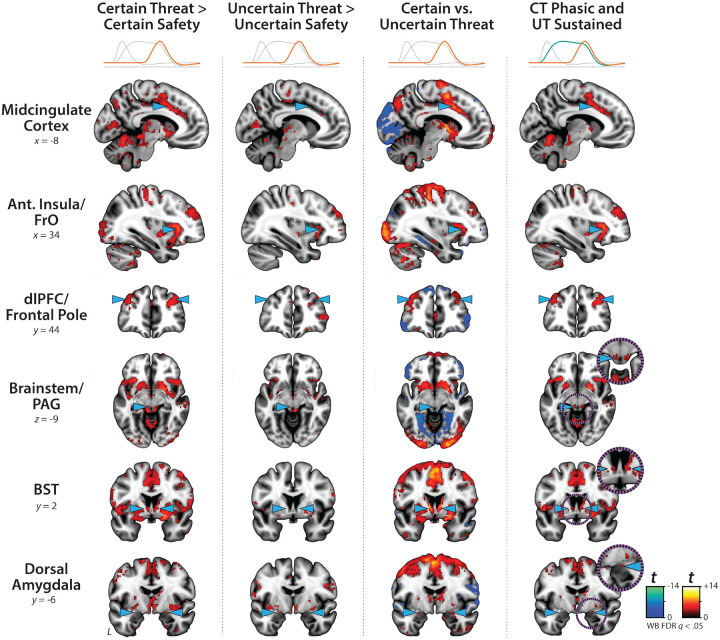
Phasic responses to certain-and-imminent threat are evident in the same regions that show sustained responses during the uncertain anticipation of threat. Regions showing significant phasic activation during the final seconds of certain-threat anticipation (*first column*) and uncertain-threat anticipation (*second column*) compared to their respective control conditions (FDR *q*<0.05, whole-brain corrected). With the exception of the PAG, every key region showed significantly stronger phasic responses to certain threat (*third column*). Visual inspection suggests that the regions showing phasic responses to certain-and-imminent threat (*first column*) largely recapitulate the circuit showing sustained responses to uncertain-threat anticipation (Figure 2). Indeed, a minimum-conjunction^54^ (logical ‘AND’) of the two thresholded contrasts revealed voxelwise overlap in all regions (*fourth column*), suggesting that certain and uncertain threat are anatomically colocalized in a shared threat-anticipation circuit. Note: 4-mm smoothing kernel. Abbreviations—Ant., anterior; BST, bed nucleus of the stria terminalis; CT, certain-threat anticipation greater than certain-safety anticipation; dlPFC, dorsolateral prefrontal cortex; FDR, false discovery rate; FrO, frontal operculum; L, left; PAG, periaqueductal gray; t, Student’s *t*-test; UT, uncertain-threat anticipation greater than uncertain-safety anticipation; vs., versus; WB, whole-brain corrected.

Emotion theory and psychophysiological research suggest that defensive responses surge in the moments just before certain threat encounters, but the underlying human neurobiology has remained unclear. Here we used a voxelwise GLM focused on the Phasic component of the OSP model—which is time-locked to the *offset* of the anticipation epoch, regardless of duration or threat certainty—to identify regions showing significantly increased activation to certain-and-imminent threat (FDR *q*<0.05, whole-brain corrected; Supplementary Figure S3). Results revealed robust phasic responses during the terminal portion of certain-threat anticipation in every key region, including the BST and right dorsal amygdala (Figure 3, *left column*). As expected, phasic responses were notably weaker (e.g., midcingulate) or nonsignificant (e.g., BST) in the corresponding moments of uncertain-threat anticipation, when threat imminence is unknown to participants (Figure 3, *middle columns*). Visual inspection of the results suggests that the regions showing phasic responses to certain-and-imminent threat recapitulate those showing sustained responses during uncertain-threat anticipation (Figure 2). Consistent with this impression, a minimum-conjunction^54^ (logical ‘AND’) of the two contrasts revealed voxelwise overlap in all regions (Figure 3, *right column*). The noteworthy degree of co-localization suggests that both kinds of threat recruit a shared threat-anticipation circuit that exhibits context-specific dynamics: sustained levels of heightened activation when threat encounters are uncertain and distal, and phasic surges in activation when threat encounters are certain and imminent (Supplementary Tables S13–S19). Furthermore, because both conditions ultimately culminate in threat encounters (Figure 1a), the absence of robust hemodynamic responses to uncertain threat indicates that phasic recruitment of the threat-anticipation circuit to acute threat is not an artifact of reinforcer (e.g., shock) delivery.

While not the focus of the present report, exploratory analyses of the OSP Onset regressor (Figure 1b) revealed significant responses to both certain- and uncertain-threat anticipation in the right dorsal amygdala in the region of the basal and cortical nuclei, consistent with an attentional orienting or salience-related function (Supplementary Figure S4 and Supplementary Tables S20–S24)^55, 56^.

### Phasic responses to acute threat reflect genuine surges in activation

The OSP Phasic results imply that activation significantly increased from the middle to the end of certain-threat anticipation, and suggest that this difference is more pronounced for certain than uncertain threat. Yet neither inference is licensed by the results, which are focused on *within*-moment statistical contrasts (e.g., *OSP Phasic regressor:* certain vs. uncertain threat). The absence of *between*-moment tests reflects the fact that the partial-regression coefficients yielded by the OSP model do not allow straightforward interpretation of between-moment contrasts (Supplementary Figure S3). To sidestep this inferential limitation, follow-up analyses leveraged a second hemodynamic model, which split the anticipation epoch into a sequence of short (6.25 s), non-overlapping blocks (rectangular functions), each convolved with a canonical hemodynamic response function (Figure 1c). Although arbitrary in timing, this Convolved Blocks model yields activation estimates that are independent, inferentially intuitive, and statistically comparable across moments in time (Supplementary Figure S3). A standard voxelwise GLM was then used to identify regions showing significant surges in activation during the late relative to the middle portion of certain-threat anticipation (FDR *q*<0.05, whole-brain corrected). Results revealed significant activation in every key region except for the PAG, with a similar pattern evident for the between-moments comparison of certain to uncertain threat (Supplementary Figure S5 and Supplementary Tables S25–S30). Taken together, these observations indicate that phasic hemodynamic responses to acute threat (Figure 3) reflect genuine surges in activation in the final moments of the anticipation epoch.

### The BST and Ce show statistically indistinguishable neural dynamics

**Figure 4.**
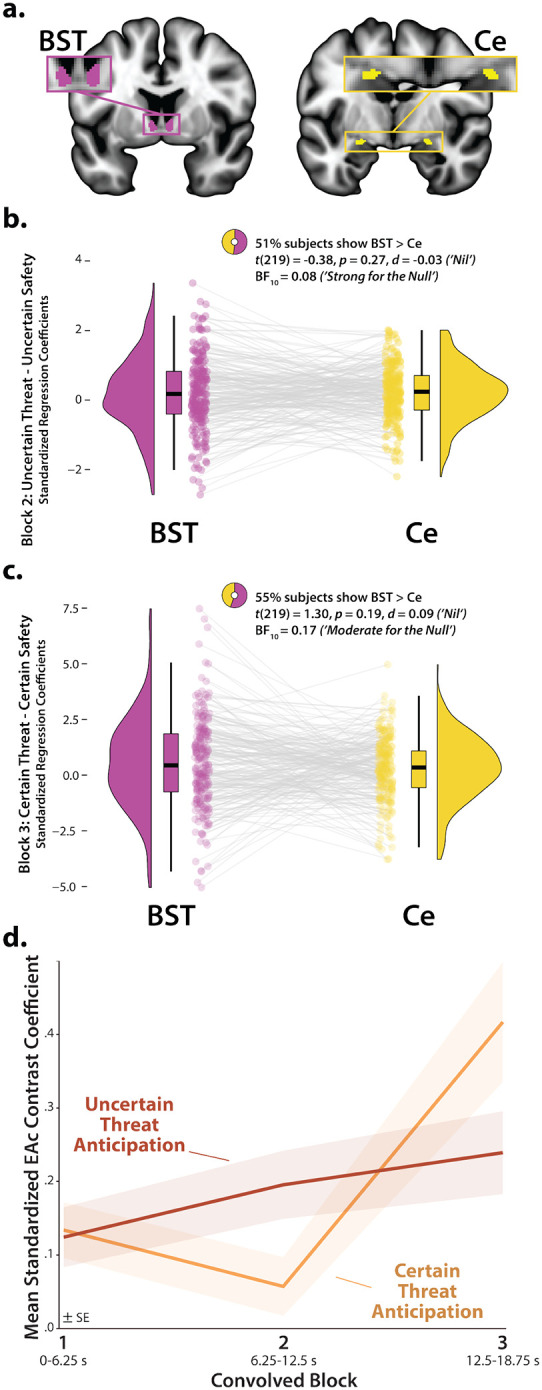
The BST and Ce show statistically indistinguishable neural dynamics. **a. *Probabilistic EAc anatomical ROIs*.** The BST (*magenta*) and Ce (*yellow*) ROIs. **b. *Uncertain-threat anticipation, second convolved block*.** The BST and Ce show negligible differences in activation during the second block (6.25–12.5 s) of uncertain-threat anticipation **c. *Certain-threat anticipation, third convolved block*.** The BST and Ce show negligible differences during the final block (12.5–18.75 s) of certain-threat anticipation. **d. *The EAc shows context-dependent dynamics*.** In aggregate, the EAc evinced a marginally significant linear increase in EAc activation during uncertain-threat anticipation (*red*; *p*=0.06) and a pronounced quadratic (‘V-shaped’) trend during certain-threat anticipation (*orange*; *p*=0.001). Colored envelopes depict the SE. Note. Raincloud plots indicate the medians (*horizontal lines*), interquartile ranges (*boxes*), and smoothed density distributions. Whiskers depict 1.5× the interquartile range. Colored dots connected by gray lines indicate mean regional activation for each participant. Note: No spatial smoothing kernel was employed for ROI analyses. Abbreviations—BF, Bayes’ factor; BST, bed nucleus of the stria terminalis; Ce, central nucleus of the amygdala; *d*, Cohen’s *dz*; EAc, central extended amygdala; SE, standard error of the mean; *t*, Student’s *t*-test.

Our approach also afforded a well-powered opportunity to revisit the functional architecture of the human central extended amygdala (EAc), a macrocircuit encompassing the bed nucleus of the stria terminalis (BST) and dorsal amygdala in the region of the central nucleus (Ce). There is widespread consensus that the EAc plays a critical role in assembling defensive responses to a broad spectrum of threats and contributes to the etiology of emotional illness^16, 18, 20, 21, 35, 43, 57^. Yet confusion persists about the respective contributions of its two major subdivisions^46, 58^. Inspired by an earlier wave of loss-of-function studies in rats^59^, it is widely believed that these regions are dissociable, with the Ce mediating phasic responses to certain-and-imminent harm and the BST mediating sustained responses to uncertain-or-remote danger^22, 41, 42, 60, 61^. This double-dissociation hypothesis has even been enshrined in the National Institute of Mental Health’s (NIMH) influential Research Domain Criteria (RDoC) framework as Acute Threat (*“fear”*) and Potential Threat (*“anxiety”*)^38–40^. Yet a growing body of mechanistic and neuroimaging evidence motivates the competing hypothesis that the BST and Ce both play a role in organizing phasic and sustained responses to threat^18, 35, 36, 45, 46, 62, 63^. Because conventional voxelwise analyses do not permit inferences about regional differences in activation, we used *a priori* probabilistic anatomical regions of interest (ROIs) to rigorously assess these competing predictions (Figure 4a). This approach has the added advantage of providing statistically unbiased effect-size estimates^64^, in contrast to earlier work by our group and others^36^. To maximize anatomical resolution, mean activation was computed for bilateral BST and Ce ROIs using spatially unsmoothed data^65^. Hypothesis testing focused on regional responses to certain and uncertain threat, relative to their respective reference conditions, using activation estimates derived using the Convolved Blocks model (Figure 1c).

As a precursor to hypothesis testing, we used one-sample Student’s *t*-tests to confirm that the BST and Ce are nominally engaged by anticipated threat (*p*<0.05, uncorrected). With one exception, results revealed uniformly significant activation (*t*(219)>2.08, *p*<0.04). The Ce did not show significant evidence of activation during the middle third of certain-threat anticipation (*t*(219)=−0.40, *p*=0.69). These observations indicate that both subdivisions of the EAc are sensitive to anticipated threat, regardless of the temporal certainty of encounters.

Next, we used a standard 2 (*Region:* BST, Ce) × 2 (*Threat Certainty:* Certain, Uncertain) × 3 (*Block:* Early, Middle, Late) repeated-measures GLM to formally test the double-dissociation hypothesis embodied in RDoC and other ‘strict-segregation’ models. None of the Regional effects were significant (*p*>0.13), including the conceptually critical Region × Threat Certainty × Block interaction (*F*(2,438)=0.72, *p*=0.46). Consistent with this, the BST and Ce showed negligible differences in activation during the second block (6.25–12.5 s) of uncertain-threat, an indicator of sustained activation, or the final block of certain-threat (12.5–18.75 s), an indicator of phasic surges in activation (|*t*|(219)<1.31, *p*>0.18; Figure 4b–c and Supplementary Figure S6). Frequentist effects were in the nil range (|*d*|=0.03–0.09) and Bayesian effects signaled moderate-to-strong evidence for the null (*BF*_*10*_=0.08–0.17). Descriptively, participants were just as likely as not to show the RDoC-predicted regional differences; for example, 51% showed stronger BST-than-Ce activation during the second block of uncertain-threat anticipation. In sum, we uncovered no evidence for the popular double-dissociation hypothesis, despite being powered to detect small regional differences in activation (Supplementary Method).

### The central extended amygdala (EAc) exhibits context-dependent neural dynamics

On the other hand, GLM results did provide evidence that the EAc in aggregate—averaged across BST and Ce—shows context-dependent neural dynamics, as indexed by significant Block and Threat-Certainty × Block effects (*F*(2,438)>5.18, *p*<0.01). As shown in Figure 4d, polynomial-trend analyses revealed a marginally significant linear increase in EAc activation during uncertain-threat anticipation (*Linear: F*(1,219)=3.58, *p*=0.06; *Quadratic: F*(1,219)=0.05, *p*=0.82). In contrast, the EAc showed a pronounced quadratic (‘V-shaped’) trend during certain-threat anticipation, manifesting as a dip in the middle third, followed by a surge of activation in the final third, when the threat encounter was most imminent (*Linear: F*(1,219)=11.30, *p*<0.001; *Quadratic: F*(1,219)=10.38, *p*=0.001).

### Brain-signature estimates of subjective distress show the same pattern of context-dependent dynamics

**Figure 5.**
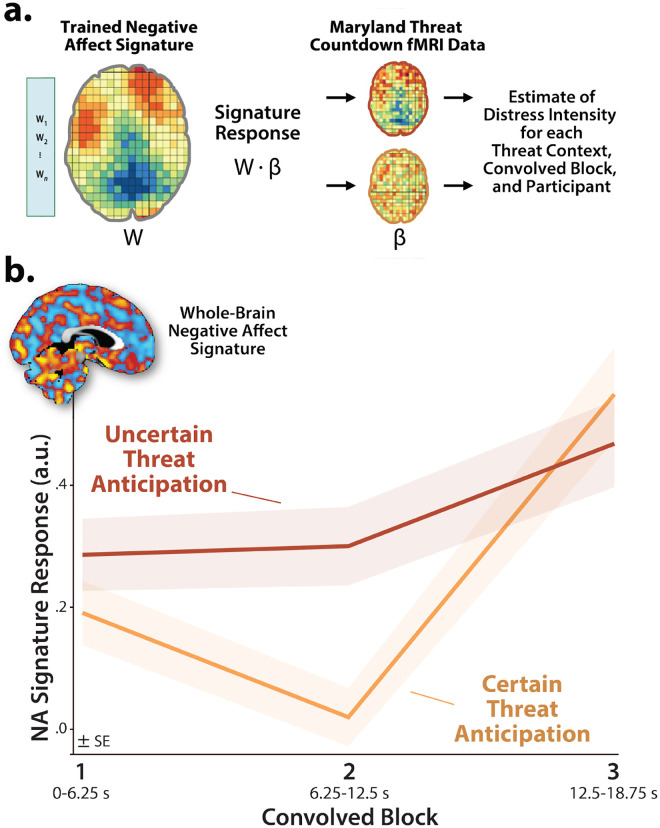
Using a multivoxel brain signature to probabilistically estimate dynamic fluctuations in threat-elicited distress. **a. *An independently trained and validated whole-brain signature of subjective negative affect was used to estimate threat-evoked distress*.** Čeko, Wager, and colleagues used machine-learning to develop a whole-brain ‘signature’—a pattern of voxelwise weights (*w*)—that is predictive of negative affect intensity in unseen data across a variety of noxious stimuli^71^. In effect, the signature treats each voxel as a weighted source of information and the overall pattern as a collective ‘best guess.’ Computing the dot-product (•) between the pattern of weights (*W*) and voxelwise activation estimates (*β*) derived for the present sample using the Convolved Blocks model generates a signature response—a probabilistic estimate of distress intensity—for every combination of threat certainty, block, and participant. This made it possible to covertly estimate moment-by-moment fluctuations in threat-elicited distress and test whether distress dynamics are sensitive to the temporal certainty of threat encounters. **b. *Subjective distress shows context-dependent dynamics*.** The estimated intensity of distress was significantly greater, on average, when anticipating uncertain encounters with threat (*p*=0.004). Significant linear and quadratic polynomial trends were evident for both certain- and uncertain-threat anticipation (*p*<0.03), but the V-shaped quadratic effect was more than an order of magnitude stronger for certain threat (*Certain: pη*^*2*^=0.31; *Uncertain: pη*^*2*^=0.02). Inset depicts the whole-brain multivoxel signature of negative affect. Hot and cool colors indicate positive and negative signature weights, respectively. Colored envelopes depict the SE. Portions of this figure were reproduced with permission from Ref.^71^. Note: 4-mm smoothing kernel. Abbreviations—fMRI, functional magnetic resonance imaging; SE, standard error of the mean.

It is tempting to interpret our neuroimaging results in terms of conscious feelings—to infer that participants experience a sustained state of elevated anxiety when the timing of threat encounters is uncertain and a surge of fear in the seconds before certain encounters. Yet standard fMRI analyses cannot address the momentary dynamics of threat-evoked distress, a limitation shared with other behavioral and psychophysiological measures, and with mechanistic models in animals^1, 66^. Likewise, more intensive continuous or intermittent ratings have the potential to fundamentally alter the nature of the experience^67, 68^. Here we used activation estimates derived from the Convolved Blocks model and an independently trained and validated whole-brain ‘signature’ of subjective negative affect to unobtrusively probe the momentary dynamics of threat-evoked feelings for the first time (Figure 5a)^69, 70^. Prior work demonstrates that this signature is a sensitive indicator of distress elicited by a variety of noxious experiences—including thermal and mechanical pain, unpleasant photographs, and aversive auditory stimuli—but is unrelated to the intensity of feelings triggered by positive stimuli, indicating specificity^69^. Conceptually similar multivoxel pattern analysis (MVPA) approaches have been successfully used in other areas of the cognitive neurosciences; for example, to covertly decode the contents of working memory or the focus of selective attention without disrupting on-going performance^70^.

As a first step, we used one-sample Student’s *t*-tests to confirm that the whole-brain signature—which was trained using data time-locked to the *presentation* of noxious stimuli—is nominally sensitive to the *anticipation* of multimodal threat (*p*<0.05, uncorrected). With one exception, results revealed robust signature responses, signaling more intense negative affect (*t*(219)>6.67, *p*<0.001). Paralleling the EAc ROI results, the signature did not show evidence of sustained distress in the middle third of certain-threat anticipation (*t*(219)=0.62, *p*=0.54). Taken with our other measures of threat-elicited distress and arousal (Supplementary Figure S1), these observations suggest that the signature is a valid index of threat-evoked anticipatory distress.

Next, we used a standard 2 (*Threat Certainty:* Certain, Uncertain) × 3 (*Block:* Early, Middle, Late) GLM to estimate momentary-by-moment fluctuations in probable distress across the two threat contexts. Consistent with our comparatively sparse retrospective ratings data (Supplementary Figure S1), results revealed significantly greater distress estimates, on average, when anticipating temporally uncertain threat encounters (*Threat Certainty: F*(1,219)=8.47, *p*=0.004; Figure 5b). The Block effect and Threat Certainty × Block interaction were also significant (*F*(2,438)>19.34, *p*<0.001). Although significant linear and quadratic polynomial trends were evident for both kinds of anticipated threat (*F*(1,219)>5.00, *p*<0.03), the V-shaped (‘surge-trough-surge’) quadratic effect was more than an order of magnitude stronger during the certain anticipation of threat encounters (*Certain: pη*^*2*^=0.31, *p*=4.71 × 10^−19^; *Uncertain: pη*^*2*^=0.02, *p*=0.02; Figure 5b). In combination with the one-sample *t*-test results (see above), this indicates that temporally uncertain-threat anticipation elicits a sustained state of heightened negative affect, whereas certain threat is associated with more complex distress dynamics, with negligible distress evident in the middle period and a phasic surge when threat is acute.

## DISCUSSION

Since the time of Freud, the fear-versus-anxiety distinction has been a hallmark of prominent models of emotion and emotional illness, including the DSM and RDoC^1, 2, 24, 38–40, 72^. Despite the enormous significance of threat-elicited emotions for public health, the neural systems underlying phasic responses to acute danger and sustained responses to uncertain harm are contentious^1, 46, 58^. Some posit that *“fear”* and *“anxiety”* are phenomenologically distinct states mediated by anatomically dissociable circuits^1, 16, 20, 41–44, 73^, whereas others suggest that they are more biologically alike than different^35, 36^. Leveraging a relatively large and ethnoracially diverse sample, translationally relevant fMRI paradigm, and theory-driven hemodynamic modeling approach (Figure 1), our results demonstrate that the anticipation of temporally certain and uncertain threat encounters recruit a remarkably overlapping distributed circuit, with anatomical colocalization evident in many previously implicated cortical and subcortical regions, including the PAG, EAc, MCC, AI/FrO, and dlPFC (Figures S2 and 3). This shared threat-anticipation circuit exhibits context-specific dynamics, evincing sustained levels of heightened activation when threat encounters are uncertain and distal (Figure 2), and phasic surges in activation when encounters are certain and imminent (Figures 3 and S5).

Among the regions highlighted by our results, the BST and Ce occupy center-stage in neurobiological models of fear and anxiety. Yet their precise contributions remain a matter of active debate^46, 53, 74^. Leveraging anatomical ROIs and spatially unsmoothed data, our results demonstrate that the BST and Ce exhibit statistically indistinguishable responses to anticipated threat—with frequentist effects in the nil range (|*d*|=0.03–0.09) and Bayesian effects indicating moderate-to-strong evidence for the null hypothesis (*BF*_*10*_=0.08–0.17)—reinforcing the possibility that these two regions make broadly similar contributions to human fear and anxiety (Figure 4)^36, 45, 63^. Both regions exhibit activation dynamics that run counter to popular double-dissociation models, with the Ce showing sustained responses to uncertain-and-distal threat and the BST showing phasic responses to acute threat.

Pathological fear and anxiety is defined, diagnosed, and treated on the basis of subjective symptoms, and for many theorists, clinicians, and laypeople, conscious feelings are the defining feature of these emotions^1, 41, 75–78^. Yet standard fMRI analyses, like animal models, do not permit strong inferences about conscious feelings. Here we used an independently trained and validated brain signature to unobtrusively decode the momentary dynamics of threat-evoked distress for the first time^69^. Results indicated that uncertain-threat anticipation is associated with a sustained state of elevated negative affect, whereas certain-threat anticipation elicits more complex dynamics, with a phasic surge of distress evident just before threat encounters (Figure 5). These observations reinforce the conclusion that human fear and anxiety, while showing distinct patterns of context-dependent temporal dynamics, reflect the operation of a common threat-anticipation circuit.

The core threat-anticipation circuit encompasses subcortical regions, such as the BST and Ce, that are critical for assembling defensive responses to anticipated threat in animals^18, 45, 79^. But it also includes frontocortical regions—including the MCC, AI/FrO, and dlPFC/FP—that have received less empirical attention and are challenging or impossible to study in rodents^80–83^. These regions have traditionally been associated with the controlled processing and regulation of emotion and cognition^48, 83–86^ and more recently implicated in the conscious experience of emotion^87^. The present findings extend past work focused on descriptive hemodynamic modeling approaches in smaller samples^36, 37^, and dovetail with meta-analytic evidence that Pavlovian fear-conditioning tasks (the prototypical experimental model of certain-and-imminent threat) and instructed threat-of-shock tasks (the prototypical experimental model of uncertain threat) recruit strongly overlapping cortico-subcortical networks in humans, including the BST^35, 88, 89^.

The present results provide a keyboard of regions and activation-dynamics, setting the stage for identifying the functional-neuroanatomical combinations most relevant to the development of pathological fear and anxiety and to the efficacy of established therapeutics. Consider the widely prescribed anxiolytic, diazepam. As yet, the neurodynamic mechanisms that underlie the blockade of threat-elicited distress by diazepam and other benzodiazepines remain unsettled. Does anxiolysis primarily reflect the dampening of sustained responses to uncertain threat in the Ce, as implied by recent work in mice^90^, or widespread changes across multiple activation metrics, as implied by our signature results?

Our results add to a growing body of evidence that the BST and Ce, while certainly not interchangeable, are more functionally alike than different^45^. The two regions are characterized by broadly similar patterns of anatomical connectivity, cellular composition, neurochemistry, and gene expression^62^. Both are poised to trigger behavioral, psychophysiological, and neuroendocrine responses to threat via dense projections to downstream effector regions^62, 91^. Both are recruited by a broad spectrum of threatening and aversive stimuli^35–37, 45, 92^. Perturbation studies in rodents demonstrate that microcircuits within and between the Ce and BST are critical for orchestrating defensive responses to both acute and uncertain threats^18, 45, 52, 93–98^. While our understanding remains far from complete, this emerging body of observations underscores the need to reformulate RDoC and other models that imply a strict segregation of certain and uncertain threat processing in the EAc. A key challenge for future research will be to determine whether our conclusions generalize to other types of threat (e.g., social), other kinds of threat uncertainty (e.g., probability, risk, ambiguity), and more naturalistic paradigms that span longer and more ecologically valid periods of threat anticipation^99^. Moving forward, an enhanced emphasis on computationally tractable paradigms has the potential to address fundamental questions about the function of the regions highlighted by our results and foster a common mathematical framework (*‘lingua franca’*) for integrating research across assays, read-outs, and species^1, 100, 101^. The Ce and BST are complex and can be subdivided into multiple subdivisions, each containing intermingled cell types with distinct, even opposing functional roles (e.g., anxiogenic vs. anxiolytic)^18, 45^. Animal models will be critical for identifying the molecules, cell types, and microcircuits governing activation dynamics in human neuroimaging studies^45^.

In conclusion, the neural circuits recruited by temporally uncertain and certain threat are not categorically different, at least when viewed through the macroscopic lens of human neuroimaging. We see evidence of anatomical colocalization—*not* segregation—in the EAc and key frontocortical regions. This shared threat-anticipation circuit shows persistently elevated activation when anticipating temporally uncertain encounters with threat and an acute burst of activation in the moments before certain encounters. Subjective distress shows parallel dynamics. These observations provide a neurobiologically grounded framework for conceptualizing typical and pathological fear and anxiety in humans and lay the groundwork for more ambitious prospective-longitudinal, clinical, computational, and mechanistic work.

## Supplementary Material

Supplement 1

## SIGNIFICANCE STATEMENT

*“Fear”* is often viewed as a phasic response to certain-and-imminent danger, whereas *“anxiety”* is a sustained response to uncertain-or-distal harm. Prior work begun to reveal the neural systems recruited by different threats, but has yet to plumb the moment-by-moment dynamics anticipated by theory and behavioral research. Here we used a novel combination of neuroimaging techniques to demonstrate that certain and uncertain threat recruit a shared threat-anticipation circuit. Activity in this circuit and covert measures of distress showed similar patterns of context-dependent dynamics, exhibiting persistent increases when anticipating uncertain-threat encounters and transient surges in the seconds before certain encounters. These observations provide fresh insights into the neurobiology of fear and anxiety, laying the groundwork for more ambitious clinical and mechanistic research.
