# Supplementary material for: A shared threat-anticipation circuit is dynamically engaged at different moments by certain and uncertain threat
Source: bioRxiv. 2024 Jul 11:2024.07.10.602972. Preprint. [Version 1] doi: 10.1101/2024.07.10.602972 (PMC11257510; doi:10.1101/2024.07.10.602972)
Supplement: Supplementary file 1 [file media-1.docx]

**Supplementary Method and Results**

Brian R. Cornwell^1 ^

Paige R. Didier^2^

Shannon E. Grogans^2^

Allegra S. Anderson^5^

Samiha Islam^6^

Hyung Cho Kim^2,3^

Manuel Kuhn^7^

Rachael M. Tillman^8^

Juyoen Hur^9^	

Zachary S. Scott^2^

Andrew S. Fox^10,11^

Kathryn A. DeYoung^2^

Jason F. Smith^2^ *

Alexander J. Shackman^2,3,4 ^*

^1^Department of Psychological & Brain Sciences, George Washington University, Washington, DC 20006 USA. Department of ^2^Psychology; ^3^Neuroscience and Cognitive Science Program; and ^4^Maryland Neuroimaging Center, University of Maryland, College Park, MD 20742 USA. ^5^Department of Psychiatry and Human Behavior, Brown University, Providence, RI 02912 USA. ^6^Department of Psychology, University of Pennsylvania, Philadelphia, PA USA. ^7^Center for Depression, Anxiety and Stress Research, McLean Hospital, Harvard Medical School, Belmont, MA 02478 USA. ^8^McGill Neuropsychology, Bethesda, MD 20814 USA. ^9^Department of Psychology, Yonsei University, Seoul 03722, Republic of Korea. ^10^Department of Psychology and ^11^California National Primate Research Center, University of California, Davis, CA 95616 USA. 

* contributed equally

**Please address manuscript correspondence to** 

Alexander J. Shackman ([shackman@umd.edu](mailto:shackman@umd.edu))

**Supplementary Method,** ***pp. 3-18***										

**Supplementary Figures,** ***pp. 19-25***

Supplementary Figure S1. 							

Supplementary Figure S2. 									

Supplementary Figure S3.										

Supplementary Figure S4.										

Supplementary Figure S5.										

Supplementary Figure S6.											

**Supplementary Tables,** ***pp. 26-268***

Supplementary Table S1. [UT > US]. 								

Supplementary Table S2. [CT > CS]. 							

Supplementary Table S3. [UT > US] $∩$  [CT > CS]. 						

Supplementary Table S4. [US > UT]. 							

Supplementary Table S5. [CS > CT]. 								

Supplementary Table S6. [UT_sustained _> US_sustained_]. 						

Supplementary Table S7. [CT_sustained _> CS_sustained_]. 						

Supplementary Table S8. [UT_sustained _> US_sustained_] $∩$ [CT_sustained _> CS_sustained_]. 	

Supplementary Table S9. [UT_sustained _> CT_sustained_]. 						

Supplementary Table S10. [US_sustained _> UT_sustained_]. 					

Supplementary Table S11. [CS_sustained _> CT_sustained_]. 					

Supplementary Table S12. [CT_sustained _> UT_sustained_]. 					

Supplementary Table S13. [CT_phasic _> CS_phasic_]. 							

Supplementary Table S14. [UT_phasic _> US_phasic_]. 							

Supplementary Table S15. [CT_phasic _> UT_phasic_]. 							

Supplementary Table S16. [UT_sustained _> US_sustained_] $∩$ [CT_phasic _> CS_phasic_]. 			

Supplementary Table S17. [CS_phasic _> CT_phasic_]. 							

# Supplementary Table S18. [US_phasic_ > UT_phasic_]. 							

# Supplementary Table S19. [UT_phasic_ > CT_phasic_]. 							

# Supplementary Table S20. [UT_onset_ > US_onset_]. 							

# Supplementary Table S21. [CT_onset_ > CS_onset_]. 							

# Supplementary Table S22. [UT_onset_ > US_onset_] $\boldsymbol{∩}$ [CT_onset_ > CS_onset_]. 				

# Supplementary Table S23. [US_onset_ > UT_onset_]. 							

# Supplementary Table S24. [CS_onset_ > CT_onset_].							

# Supplementary Table S25. [CT_CB3_ > CT_CB2_]. 							

# Supplementary Table S26. [UT_CB3_ > UT_CB2_]. 							

# Supplementary Table S27. [CT_CB3_ - CT_CB2_] > [UT_CB3_ - UT_CB2_].					

# Supplementary Table S28. [CT_CB2_ > CT_CB3_]. 							

# Supplementary Table S29. [UT_CB2_ > UT_CB3_]. 							

# Supplementary Table S30. [UT_CB3_ - UT_CB2_] > [CT_CB3_ - CT_CB2_]. 					

# Supplementary References, *pp. 269-274*								

# Note. Abbreviations—CB, convolved block (1-3); CS, certain-safety anticipation; CT, certain-threat 

# anticipation; Onset, onset regressor for the OSP model; Phasic, phasic regressor for the OSP model; 

# Sustained, sustained regressor for the OSP model; US, uncertain-safety anticipation; UT, uncertain-threat 

# anticipation. 

**SUPPLEMENTARY METHOD**

**Study Overview and Recruitment** 

As part of a recently completed prospective-longitudinal study focused on individuals at risk for the development of anxiety disorders and depression (R01-MH107444), we used well-established psychometric measures of Neuroticism/Negative Emotionality (N/NE) to screen 6,594 first-year university students (57.1% female; 59.0% White, 19.0% Asian, 9.9% African American, 6.3% Hispanic, 5.8% Multiracial/Other; *M*=19.2 years, *SD*=1.1 years) (Grogans et al., *in press*). Screening data were stratified into quartiles (top quartile, middle quartiles, bottom quartile), separately for males and females. Individuals who met preliminary inclusion criteria were independently and randomly recruited via email from each of the resulting six strata. Because of the parent project’s focus on internalizing risk, approximately half the participants were recruited from the top quartile, with the remainder evenly split between the middle and bottom quartiles. This enabled us to sample a broad spectrum of the risk-conferring N/NE phenotype without gaps or discontinuities—in contrast to prior work focused on convenience samples (Charpentier et al., 2021)—while balancing biological sex. Simulation work suggests that this over-sampling (‘enrichment’) approach does not bias statistical tests to a degree that would compromise their validity (Hauner et al., 2014). All participants had normal or corrected-to-normal color vision, and reported the absence of lifetime neurological symptoms, pervasive developmental disorder, very premature birth, medical conditions that would contraindicate MRI, and prior experience with noxious electrical stimulation. All participants were free from a lifetime history of psychotic and bipolar disorders; a current diagnosis of a mood, anxiety, or trauma disorder (past 2 months); severe substance abuse;  active suicidality; and on-going psychiatric treatment as determined by an experienced masters-level diagnostician using the Structured Clinical Interview for DSM-5 (First et al., 2015). Participants provided informed written consent and all procedures were approved by the Institutional Review Board at the University of Maryland, College Park (Protocol #659385). Data from this study were featured in prior work focused on validation of the threat-anticipation paradigm (Hur, Smith, et al., 2020; Shackman et al., *in press*), neuroanatomical correlates of childhood anxiety (Bas-Hoogendam et al., 2022), threat-related neural activity and negative affect (Grogans et al., *in press**;* Hur et al., 2022), personality and internalizing symptoms (Conway et al., *in press*), and social anxiety and negative affect (Hur, DeYoung, et al., 2020), but have never been used to address the present aims. 

**Power Analyses**

Sample size was determined *a priori* as part of the application for the grant that supported data collection (R01-MH107444). The target sample size (*N*≈240) was chosen to afford acceptable power and precision given available resources. At the time of study design, G-power (version 3.1.9.2) indicated >99% power to detect a benchmark (‘generic’) medium-sized effect (*r*=0.30) with up to 20% planned attrition (*N*=192 usable datasets) using α=0.05, two-tailed (Erdfelder et al., 1996). In practice, the final sample of 220 usable fMRI datasets (see below) provides 80% power to detect a mean difference as small as Cohen’s *d*=0.19, traditionally considered a small effect (Cohen, 1988). The study was not preregistered.

**Participants**

A total of 241 participants were recruited and scanned. Of these, 6 withdrew due to excess distress in the scanner and 1 withdrew from the study after the imaging session. Another 14 participants were excluded from fMRI analyses because of incidental neurological findings (*n*=4), technical problems (*n*=2), or insufficient usable data (*n*=8; see below), yielding an ethnoracially diverse final sample of 220 participants (49.5% female; 61.4% White, 18.2% Asian, 8.6% African American, 4.1% Hispanic, 7.3% Multiracial/Other; *M*=18.8 years, *SD*=0.4 years).  

**Threat-Anticipation Paradigm**

***Paradigm Structure and Design Considerations.*** The Maryland Threat Countdown paradigm is a well-established, fMRI-optimized variant of temporally uncertain-threat assays that have been validated using fear-potentiated startle and acute anxiolytic administration (e.g., benzodiazepine) in mice, rats, and humans (Daldrup et al., 2015; Hefner et al., 2013; Lange et al., 2017; Miles et al., 2011; Moberg et al., 2017). The paradigm has been successfully deployed in several prior fMRI studies (Grogans, Hur, et al., 2023; Hur et al., 2022; Hur, Smith, et al., 2020; Kim et al., 2023).  

As shown schematically in **Figure 1** in the main report, the paradigm takes the form of a 2 (*Valence:* Threat, Safety) × 2 (*Temporal Certainty:* Uncertain, Certain) randomized, event-related, repeated-measures design (3 scans; 6 trials/condition/scan). Participants were completely informed about the task design and contingencies prior to scanning. Simulations were used to optimize the detection and deconvolution of task-related hemodynamic signals. Stimulus presentation was controlled using Presentation software (version 19.0, Neurobehavioral Systems, Berkeley, CA).

On certain-threat trials, participants saw a descending stream of integers (‘count-down;’ e.g., 30, 29, 28...3, 2, 1) for 18.75 s. To ensure robust fear and anxiety, this anticipation epoch culminated with the presentation of a noxious electric shock, unpleasant photograph (e.g., mutilated body), and thematically related audio clip (e.g., scream, gunshot). Uncertain-threat trials were similar, but the integer stream was randomized and presented for an uncertain and variable duration (8.75-30.00 s; *M*=18.75 s). Participants knew that something aversive was going to occur, but they had no way of knowing precisely when. Consistent with methodological recommendations (Shackman & Fox, 2016), the average duration of the anticipation epoch was identical across conditions, ensuring an equal number of measurements (TRs/condition). The specific mean duration was chosen to enhance detection of task-related differences in the blood oxygen level-dependent (BOLD) signal (‘activation’) (Henson, 2007) and to allow sufficient time for sustained responses to become evident. Safety trials were similar, but terminated with the presentation of benign reinforcers (see below). Valence was continuously signaled during the anticipation epoch (‘countdown’) by the background color of the display. Temporal certainty was signaled by the nature of the integer stream. Certain trials always began with the presentation of the number 30. On uncertain trials, integers were randomly drawn from a near-uniform distribution ranging from 1 to 45 to reinforce the impression that they could be much shorter or longer than Certain trials and to minimize incidental temporal learning (‘time-keeping’). To concretely demonstrate the variable duration of Uncertain trials, during scanning, the first three uncertain trials featured short (8.75 s), medium (15.00 s), and long (28.75 s) anticipation epochs. To mitigate potential confusion and eliminate mnemonic demands, a lower-case ‘c’ or ‘u’ was presented at the lower edge of the display throughout the anticipatory epoch. White-noise visual masks (3.2 s) were presented between trials to minimize the persistence of visual reinforcers in iconic memory. Subjective distress ratings and skin conductance were also acquired, as detailed in a prior report and summarized in **Supplementary Figure S1** (Grogans et al., *in press*).    

***Procedures.*** Prior to scanning, participants practiced an abbreviated version of the paradigm (without electrical stimulation) until they indicated and staff confirmed understanding. Benign and aversive electrical stimulation levels were individually titrated. *Benign Stimulation.* Participants were asked whether they could “reliably detect” a 20 V stimulus and whether it was “at all unpleasant.” If the participant could not detect the stimulus, the voltage was increased by 4 V and the process repeated. If the participant indicated that the stimulus was unpleasant, the voltage was reduced by 4 V and the process was repeated. The final level chosen served as the benign electrical stimulation during the imaging assessment (*M*=21.06 V, *SD*=5.06). *Aversive Stimulation.* Participants received a 100 V stimulus and were asked whether it was “as unpleasant as you are willing to tolerate”—an instruction specifically chosen to maximize anxious distress and arousal. If the participant indicated that they were willing to tolerate more intense stimulation, the voltage was increased by 10 V and the process repeated. If the participant indicated that the stimulus was too intense, the voltage was reduced by 5 V and the process repeated. The final level chosen served as the aversive electrical stimulation during the imaging assessment (*M*=117.85 V, *SD*=26.10). Following each scan, staff re-assessed whether stimulation was sufficiently intense and increased the level as necessary. 

***Electrical Stimuli.*** Electrical stimuli (100 ms; 2 ms pulses every 10 ms) were generated using an MRI-compatible constant-voltage stimulator system (STMEPM-MRI; Biopac Systems, Inc., Goleta, CA) and delivered using MRI-compatible, disposable carbon electrodes (Biopac) attached to the fourth and fifth digits of the non-dominant hand. 

***Visual Stimuli.*** A total of 72 aversive and benign photographs (1.8 s) were selected from the International Affective Picture System (for details, see Hur, Smith, et al., 2020). Visual stimuli were digitally back-projected (Powerlite Pro G5550, Epson America, Inc., Long Beach, CA) onto a semi-opaque screen mounted at the head-end of the scanner bore and viewed using a mirror mounted on the head-coil.   

***Auditory Stimuli.*** Seventy-two aversive and benign auditory stimuli (0.8 s) were adapted from open-access online sources and delivered using an amplifier (PA-1 Whirlwind) with in-line noise-reducing filters and ear buds (S14; Sensimetrics, Gloucester, MA) fitted with noise-reducing ear plugs (Hearing Components, Inc., St. Paul, MN).  

**MRI Data Acquisition**

MRI data were acquired using a Siemens Magnetom TIM Trio 3 Tesla scanner (32-channel head-coil). During scanning, foam inserts were used to immobilize the participant’s head within the head-coil and mitigate potential motion artifact. Participants were continuously monitored using an MRI-compatible eye-tracker (Eyelink 1000; SR Research, Ottawa, Ontario, Canada) and the AFNI real-time motion plugin (Cox, 1996). Sagittal T1-weighted anatomical images were acquired using a magnetization prepared rapid acquisition gradient echo sequence (TR=2,400 ms; TE=2.01 ms; inversion time=1,060 ms; flip=8°; slice thickness=0.8 mm; in-plane=0.8 × 0.8 mm; matrix=300 × 320; field-of-view=240 × 256). A T2-weighted image was collected co-planar to the T1-weighted image (TR=3,200 ms; TE=564 ms; flip angle=120°). To enhance resolution, a multi-band sequence was used to collect oblique-axial EPI volumes (multiband acceleration=6; TR=1,250 ms; TE=39.4 ms; flip=36.4°; slice thickness=2.2 mm, number of slices=60; in-plane resolution=2.1875 × 2.1875 mm; matrix=96 × 96). Images were collected in the oblique-axial plane (approximately −20° relative to the AC-PC plane) to minimize potential susceptibility artifacts. For the threat-anticipation task, three 478-volume EPI scans were acquired. The scanner automatically discarded 7 volumes prior to the first recorded volume. To enable fieldmap correction, two oblique-axial spin echo (SE) images were collected in opposing phase-encoding directions (rostral-to-caudal and caudal-to-rostral) at the same location and resolution as the functional volumes (i.e., co-planar; TR=7,220 ms; TE=73 ms). Measures of respiration and pulse were continuously acquired during scanning using a respiration belt and photo-plethysmograph affixed to the first digit of the non-dominant hand. Following the last scan, participants were removed from the scanner, debriefed, compensated, and discharged.

**MRI Pipeline**

Methods were optimized to minimize spatial normalization error and other potential sources of noise and are similar to other recent work by our group (Grogans et al., *in press*). Data were visually inspected before and after processing for quality assurance.

***Anatomical Data Processing.*** T1- and T2-weighted images were inhomogeneity corrected using *N4* (Tustison et al., 2010) and denoised using *ANTS* (Avants et al., 2011). The brain was then extracted using *BEaST* (Eskildsen et al., 2012) and brain-extracted and normalized reference brains from *IXI* (BIAC, 2022). Brain-extracted T1 images were normalized to a version of the brain-extracted 1-mm T1-weighted MNI152 (version 6) template (Grabner et al., 2006) modified to remove extracerebral tissue. Normalization was performed using the diffeomorphic approach implemented in *SyN* (version 2.3.4) (Avants et al., 2011). T2-weighted images were rigidly co-registered with the corresponding T1 prior to normalization. The brain extraction mask from the T1 was then applied. Tissue priors were unwarped to native space using the inverse of the diffeomorphic transformation (Lorio et al., 2016). Brain-extracted T1 and T2 images were segmented using native-space priors generated in *FAST* (version 6.0.4) (Jenkinson et al., 2012) for subsequent use in T1-EPI co-registration (see below). 

***Fieldmap Data Processing.*** SE images and *topup* were used to create fieldmaps. Fieldmaps were converted to radians, median-filtered, and smoothed (2-mm). The average of the distortion-corrected SE images was inhomogeneity corrected using *N4* and masked to remove extracerebral voxels using *3dSkullStrip* (*AFNI* version 23.1.10). The resulting mask was minimally eroded to further exclude extracerebral voxels.

***Functional Data Processing.*** EPI files were de-spiked (*3dDespike*), slice-time corrected to the TR-center using *3dTshift*, and motion-corrected to the first volume and inhomogeneity corrected using *ANTS* (12-parameter affine). Transformations were saved in ITK-compatible format for subsequent processing (McCormick et al., 2014). The first volume was extracted for EPI-T1 co-registration. The reference EPI volume was simultaneously co-registered with the corresponding T1-weighted image in native space and corrected for geometric distortions using boundary-based registration (Jenkinson et al., 2012). This step incorporated the previously created fieldmap, undistorted SE, T1, white matter (WM) image, and masks. The spatial transformations necessary to transform each EPI volume from native space to the reference EPI, from the reference EPI to the T1, and from the T1 to the template were concatenated and applied to the processed EPI data in a single step to minimize incidental spatial blurring. Normalized EPI data were resampled (2 mm^3^) using fifth-order b-splines. Voxelwise analyses employed data that were spatially smoothed (4-mm) using *3DblurInMask*. To minimize signal mixing, smoothing was confined to the gray-matter compartment, defined using a variant of the Harvard-Oxford cortical and subcortical atlases that was expanded to include the bed nucleus of the stria terminalis (BST) and periaqueductal gray (PAG) (Desikan et al., 2006; Edlow et al., 2012; Frazier et al., 2005; Makris et al., 2006; Theiss et al., 2017). Focal analyses of the central extended amygdala (EAc) leveraged spatially unsmoothed data and anatomically defined regions of interest (ROIs; see below), consistent with prior work by our group (Grogans et al., *in press**;* Kim et al., 2023; Tillman et al., 2018). 

**fMRI Data Exclusions and Hemodynamic Modeling**

***Data Exclusions.*** Volume-to-volume displacement (>0.5 mm) was used to assess residual motion artifact. Scans with excessively frequent residual artifacts (>2 *SD*) were discarded. Participants with insufficient usable fMRI data (<2 scans) were excluded from analyses (see above).

***Overview of First-Level (Single-Subject) fMRI Modeling.*** For each participant, first-level modeling was performed using general linear models (GLMs) implemented in *3dREMLfit* (ARMA_1,1_; 4th-order Legendre high-pass filter). Regressors were convolved with the *SPM12* canonical HRF. Epochs corresponding to the presentation of the four types of reinforcers, white-noise visual masks, and rating prompts were simultaneously modeled using the same approach. Consistent with our prior work, nuisance variates included volume-to-volume displacement and first derivative, 6 motion parameters and first derivatives, cerebrospinal fluid (CSF) signal, instantaneous pulse and respiration rates, and nuisance signals (e.g., brain edge, CSF edge, global motion, white matter, extracerebral soft tissue) (Anderson et al., 2011; Pruim et al., 2015). Volumes with excessive volume-to-volume displacement (>0.75 mm) and those during and immediately following reinforcer delivery were censored. EPI volumes acquired before the first trial and following the final trial were unmodeled and contributed to the baseline estimate.

***Conventional ‘Boxcar’ Model.*** The present sample of 220 datasets represents a superset of the 99 featured in an earlier report from our group that employed a conventional ‘boxcar’ fMRI modeling approach and an older data-processing pipeline (Hur, Smith, et al., 2020). As a precursor to hypothesis testing, we used a conventional first-level modeling approach to confirm that the larger, reprocessed dataset broadly reproduced our published observations. Hemodynamic reactivity to the threat-anticipation paradigm was modeled using variable-duration rectangular (‘boxcar’) regressors that spanned the anticipation (‘countdown’) epochs of uncertain-threat, certain-threat, and uncertain-safety trials. To maximize design efficiency, certain-safety anticipation served as the reference condition and contributed to the baseline estimate (Poline et al., 2007). 

***OSP Model.*** Neuroimaging research by our group and others has relied on oversimplified ‘boxcar’ modeling approaches that reduce the neural dynamics anticipated by theory and psychophysiological research to a single average response (Wang et al., *in press*). Here, we used two complementary hemodynamic modeling approaches to characterize the dynamic signals elicited by certain- and uncertain-threat anticipation. The OSP model used a multiple-regression framework to identify the variance in threat-anticipation signals uniquely associated—in the partial-correlation sense—with temporally overlapping Onset, Sustained, and Phasic regressors (**Figure 1b**). The first-level design matrix incorporated a punctate event time-locked to the onset of the anticipation epoch, a variable-duration rectangular function that spanned the entirety of the anticipation epoch (to capture sustained increases in activation), and a temporally overlapping rectangular-function time-locked to the *offset* of the anticipation epoch (to capture phasic surges in activation to acute threat, independent of threat certainty or trial duration). Certain-safety anticipation served as the implicit baseline. The duration of the phasic regressor (6.15 s) was selected on the basis of theory and simulations aimed at minimizing regressor co-linearity (mean condition-wise variance inflation factor<1.93). As described in more detail in **Supplementary Figure S3**, the Sustained regressor captures variance in the hemodynamic signal associated with a particular trial type (e.g., uncertain-threat anticipation) above-and-beyond that captured by the Onset and Phasic regressors. In contrast to conventional boxcar models, this provides an estimate of sustained activation that is unconfounded by non-specific orienting or salience responses reflexively triggered by the trial onset (Menon, 2015; Sokolov et al., 2002). Likewise, the Phasic regressor captures variance in the hemodynamic signal above-and-beyond that captured by the Onset and Sustained regressors, with positive coefficients indicating an increase in activation in the final moments of the anticipation epoch relative to that explained by the Sustained and Onset regressors. In sum, this model casts the overall magnitude of the hemodynamic signal as a linear combination of the O, S, and P regressors; nuisance regressors; and error (**Supplementary Figure S3**). This provides a unified, non-arbitrary way to address the variable duration and timing of uncertain-threat and -safety trials.  

***Convolved Blocks Model.*** To clarify interpretation, we employed a piecewise approach that arbitrarily split the anticipation epoch into a sequence of 2-5 short (6.25 s), non-overlapping rectangular functions or ‘blocks’, each convolved with a canonical HRF (**Figure 1c**). Here, the second certain-safety block (6.25-12.5 s) served as the reference condition. While arbitrary in timing, the Convolved Blocks model yields activation estimates that are independent, intuitive, and statistically comparable across time—unlike the partial correlations yielded by the OSP model—enabling us to more fully assess apparent surges in activation in the seconds just prior to certain threat encounters (**Supplementary Figure S3**). Hypothesis testing focused on the first three blocks, corresponding to the mean duration of certain-and uncertain-threat anticipation. Regressor co-linearity was acceptable (mean condition-wise variance inflation factor<1.91) (Mumford et al., 2015).

***EAc ROIs.*** BST and Ce activation was quantified using well-established, anatomically defined ROIs (Theiss et al., 2017; Tillman et al., 2018).  The probabilistic BST ROI was thresholded at 25%. It mostly encompasses the supra-commissural BST, given the difficulty of reliably discriminating the borders of regions below the anterior commissure in T1-weighted images (Kruger et al., 2015). Bilateral ROIs were decimated to the 2-mm resolution of the fMRI data. 

**Analytic Strategy**

***Overview.*** Analyses were performed using a combination of *SPM12* (Wellcome Centre for Human Neuroimaging, 2022), *SPSS* (version 27.0.1), and *JASP* (version 0.18.3.0) (Love et al., 2019). Diagnostic procedures and data visualizations were used to confirm that test assumptions were satisfied (Tukey, 1977). Some figures were created using *R* (version 4.0.2), *Rstudio* (version 1.2.1335), *tidyverse* (version 2.0), *ggplot2* (version 3.3.6) *ggpubr* (version 0.6.0), *plotrix* (version 3.8-4), and *MRIcron (*version 1.0.20190902*)* (Kassambara, 2023; Lemon, 2006; R Core Team, 2022; Rorden, 2019; RStudio Team, 2022; Wickham, 2016; Wickham et al., 2019). Clusters and peaks were labeled using the Harvard–Oxford and Mai atlases (Desikan et al., 2006; Frazier et al., 2005; Mai et al., 2015; Makris et al., 2006).

***Resource Sharing.*** Raw data are available at the National Institute of Mental Health Data Archive ([https://nda.nih.gov/edit_collection.html?id=2447](https://nda.nih.gov/edit_collection.html?id=2447)). Neuroimaging maps are available at NeuroVault ([https://neurovault.org/collections/15274](https://neurovault.org/collections/15274)). Task materials, statistical code, and de-identified processed data are available at OSF ([https://osf.io/e2ngf](https://osf.io/e2ngf)). The negative affect brain signature is available at Github ([https://github.com/canlab/Neuroimaging_Pattern_Masks/tree/master/Multivariate_signature_patterns/2021_Ceko_MPA2_multiaversive](https://github.com/canlab/Neuroimaging_Pattern_Masks/tree/master/Multivariate_signature_patterns/2021_Ceko_MPA2_multiaversive)). 

***Conventional Hemodynamic Model.*** The present sample of 220 datasets represents a superset of the 99 featured in an earlier report that employed a conventional ‘boxcar’ modeling approach, older data-processing pipeline, and larger spatial-smoothing kernel (6-mm) (Hur, Smith, et al., 2020). Here we used standard voxelwise GLMs to confirm that conventional modeling of the larger, reprocessed dataset broadly reproduced our published results (FDR *q*<0.05, whole-brain corrected). A minimum-conjunction analysis (Logical ‘AND’) was used to identify regions showing significant activation during the anticipation of certain *and* uncertain threat relative to their respective reference conditions (Nichols et al., 2005).

***Voxelwise Analyses of the Theory-Driven OSP and Convolved-Blocks Models.*** Standard voxelwise GLMs were used for all analyses (FDR *q*<0.05, whole-brain corrected). For activation estimates derived from the OSP model, hypothesis testing focused on the Sustained and Phasic regressors. In this context, Phasic activation to uncertain-threat—where the precise timing of threat encounters is unknown, making it impossible to mount a transient ‘alarm’ response—represents a natural control condition for Phasic responses to certain-threat, where the timing of encounters is known. Likewise, hypothesis testing for the Convolved Blocks model focused on the second (6.25-12.5 s) and third (12.5-18.75) blocks. 

The two hemodynamic models have complementary strengths and weaknesses. The OSP Phasic regressor is time-locked to the *offset* of the anticipation epoch, ensuring that it always indexes neural activation in the seconds just before threat is encountered, regardless of threat certainty or the overall duration of the anticipation epoch. Furthermore, because it is effectively a partial correlation, the OSP Phasic regressor captures variance in activation above-and-beyond that captured by the temporally overlapping Sustained regressor, providing a relatively conservative estimate of phasic surges in activation. In contrast, the Convolved Blocks regressors are fixed in time, matched for latency across conditions, and non-overlapping, allowing straightforward statistical comparisons of different moments in time (e.g., later vs. earlier). 

   

***EAc ROI Analyses.*** ROI analyses used activation estimates (i.e., standardized regression coefficients) for Convolved Blocks 1-3, extracted and averaged for each contrast (e.g., uncertain-threat vs. uncertain-safety anticipation), region, and participant. Unlike conventional whole-brain voxelwise analyses—which screen thousands of voxels for statistical significance and yield optimistically biased associations—anatomically defined ROIs ‘fix’ the measurements-of-interest *a priori*, providing statistically unbiased estimates of brain-phenotype associations (Poldrack et al., 2017). As a precursor to hypothesis testing, we used one-sample Student’s *t*-tests to confirm that both ROIs—which capitalized on spatially unsmoothed data—are nominally engaged by anticipated threat (*p*<0.05, uncorrected). For hypothesis testing, we used a standard 2 (*Region:* BST, Ce) × 2 (*Threat* *Certainty:* Certain, Uncertain) × 3 (*Convolved* *Block:* 1, 2, 3) repeated-measures GLM (Huynh–Feldt correction) to interrogate potential regional differences in activation dynamics. Hypothesis testing focused on the second block (6.25-12.5 s) of uncertain-threat anticipation (an index of sustained activation) and the final block (12.5-18.75 s) of certain-threat anticipation (an index of phasic surges in activation). Significant GLM effects were interrogated using linear and quadratic polynomial contrasts, consistent with past psychophysiological research (Grillon, Ameli, Foot, et al., 1993; Grillon, Ameli, Merikangas, et al., 1993; Löw et al., 2015). Polynomial-trend analyses enabled us to evaluate whether the time-course of activation differed as a function of Region or Threat Certainty; in particular, to test whether certain-and-imminent threat is associated with phasic surges in activation in the final third of the anticipation epoch (relative to the middle third) and whether this hypothesized quadratic trend is stronger than that evident during the identical moments of uncertain-threat anticipation. Frequentist (Cohen’s *d*) and Bayesian (*BF*_*10*_) effect sizes and standard interpretive benchmarks were used to clarify non-significant regional differences (*Cohen’s d:* Small=0.200 to .101; Nil≤0.100; *BF*_*10*_*:* Weak evidence for H_0_=0.332-1.000; Moderate evidence for H_0_=0.099-0.333; Strong evidence for H_0_≤0.100) (Schimmack, 2019; van Doorn et al., 2021). 

***Brain Signature Analyses.*** Standard fMRI analyses cannot address the momentary dynamics of threat-evoked emotions (Grogans, Bliss-Moreau, et al., 2023; Poldrack, 2011). Here we used an independently trained and validated multivoxel ‘signature’ of negative affect to covertly probe moment-by-moment fluctuations in threat-elicited distress. Čeko, Wager, and colleagues used machine-learning to develop a pattern of voxelwise weights predictive of the intensity of negative affect in unseen data (Čeko et al., 2022). They demonstrated that the signature is a sensitive indicator of distress elicited by a range of noxious experiences—including thermal and mechanical pain, unpleasant photographs, and aversive auditory stimuli—but is unrelated to the intensity of feelings triggered by positive stimuli, indicating specificity. We computed the dot-product between the signature and activation estimates derived for the present sample using the Convolved Blocks model to generate signature responses (a probabilistic estimate of negative affect intensity) for every combination of threat certainty (certain, uncertain), block (1, 2, 3), and participant. We used one-sample Student’s *t*-tests to confirm that the signature, which was trained using activation estimates time-locked to the *presentation* of aversive stimuli, is nominally sensitive to the *anticipation* of threat encounters (*p*<0.05, uncorrected). For hypothesis testing, we used a standard 2 (*Threat* *Certainty:* Certain, Uncertain) × 3 (*Convolved* *Block:* 1, 2, 3) repeated-measures GLM (Huynh–Feldt correction) to assess dynamic fluctuations in signature-estimated distress across threat contexts. Hypothesis testing again focused on the second block of uncertain-threat anticipation and the final block of certain-threat anticipation. Significant GLM effects were decomposed using polynomial-trend analyses, as detailed in the prior section.  

**Supplementary Figure S1. The Maryland Threat Countdown paradigm is a valid experimental probe of anticipatory fear and anxiety.** Participants were periodically prompted to rate the intensity of fear/anxiety during the anticipation (‘countdown’) period of the prior trial, using a 1 (*minimal*) to 4 (*maximal*) scale (16.7% trials). Skin conductance was continuously acquired throughout. As detailed elsewhere, we used a series of repeated-measures GLMs to confirm that the threat-anticipation paradigm had the intended impact on subjective symptoms of distress and objective signs of arousal (Grogans et al., *in press*). **a.** ***Threat anticipation evokes subjective distress.*** Participants Fearful and anxious feelings were significantly elevated during the anticipation of Threat compared to Safety, and this was particularly evident when threat encounters were uncertain in their timing (*Valence:* *F*(1,219)=1,108.5, *p*<0.001; *Certainty:* *F*(1,219)=208.4, *p*<0.001; *Valence × Certainty:* *F*(1,219)=31.9, *p*<0.001; *Threat, Uncertain vs. Certain:* *F*(1,219)=148.5, *p*<0.001; *Safety, Uncertain vs. Certain: F*(1,219)=76.6, *p*<0.001). **b.** ***Threat anticipation evokes objective signs of arousal.*** A similar pattern was evident for skin conductance level, an objective psychophysiological index of arousal (*Valence:* *F*(1,217)=792.79, *p*<0.001; *Certainty:* *F*(1,217)=139.6, *p*<0.001; *Valence × Certainty:* *F*(1,217)=664.4, *p*<0.001; *Threat, Uncertain vs. Certain: F*(1,217)=457.8, *p*<0.001; *Safety, Uncertain vs. Certain:* *F*(1,217)=271.2, *p*<0.001). Two participants were excluded from skin-conductance analyses due to insufficient usable data. Taken together, these observations confirm the validity of the Maryland Threat Countdown as an experimental probe of fear and anxiety, replicating prior work in community volunteers (Kim et al., 2023). Bars indicate the means (*colored bars*), Bayesian 95% highest density intervals (HDI; *gray bands*), and individual participants (*gray dots*).   

# Supplementary Figure S2. Uncertain- and certain-threat anticipation recruit a shared cortico-subcortical circuit. To facilitate comparison with prior work, we computed a conventional ‘boxcar’ analysis, which models the anticipation epoch (‘countdown’) as a single average response. As shown in the *left column*, uncertain-threat anticipation was associated with significant activation across a widely distributed network of regions previously implicated in the expression and regulation of human fear and anxiety (FDR *q*<.05, whole-brain corrected) (Shackman & Fox, 2021). As shown in the *middle column*, similar results were evident for certain-threat anticipation. In fact, as shown in the right column, a minimum-conjunction analysis (Nichols et al., 2005) (Logical ‘AND’ of the 2 thresholded contrasts) confirmed voxelwise co-localization in each of these key regions. These observations replicate prior work in university and community samples, confirm that our paradigm robustly engages the canonical threat-anticipation circuit, and set the stage for detailed analyses of more complex neural dynamics (Grogans et al., *in press**;* Hur, Smith, et al., 2020; Kim et al., 2023; Shackman et al., *in press*). Note. To enhance neuroanatomical resolution, these analyses leveraged a smaller spatial-smoothing kernel (4-mm) than prior work by our group (6-mm). Abbreviations—Ant., anterior; BST, bed nucleus of the stria terminalis; dlPFC, dorsolateral prefrontal cortex; FDR, false discovery rate; FrO, frontal operculum; L, left; PAG, periaqueductal gray; t, Student’s *t*-test; WB, whole-brain corrected. 

**Supplementary Figure S3. Theory-driven hemodynamic modeling.** The vast majority of neuroimaging studies have relied on oversimplified ‘boxcar’ modeling approaches that reduce threat-related activation to a single average response (Wang et al., *in press*). Here we used two complementary hemodynamic modeling approaches to quantify and statistically interrogate time-varying responses to certain- and uncertain-threat anticipation. Panels ***a*** and ***b*** provide a general overview of the two theory-driven models. Panels ***c-h*** illustrate key interpretive nuances. **a.** ***Overview of the OSP Model.*** The OSP model used multiple-regression to partition the variance in threat-anticipation signals uniquely associated with overlapping Onset, Sustained, and Phasic regressors. The design matrix incorporated a punctate event time-locked to the onset of the anticipation (‘countdown’) epoch, a variable-duration rectangular function spanning the entirety of the anticipation epoch (to capture sustained increases in activation), and a temporally overlapping rectangular-function time-locked to the *offset* of the anticipation epoch (to capture phasic surges in activation to acute threat, independent of threat certainty or trial duration). This approach provides a unified, non-arbitrary means of addressing the variable duration and timing of uncertain trials (**Figure 1a**). **c.** ***Overview of the*** ***Convolved Blocks (CB) Model.*** To clarify interpretation, we employed a piecewise hemodynamic model that arbitrarily splits the anticipation epoch into a sequence of short (6.25 s), non-overlapping rectangular functions (‘blocks’), each convolved with a canonical hemodynamic response function. Hypothesis testing focused on the first three blocks, corresponding to the average duration of certain-and uncertain-threat anticipation (cf. **Figure 1a**). While arbitrary in timing, the Convolved Blocks model yields activation estimates that are independent, intuitive, and statistically comparable across time, making it possible to more completely assess apparent surges in activation in the moments just prior to certain-threat encounters. **c.** Consider a voxel that shows a sustained level of heightened activation during the certain anticipation of threat (*red*). In the OSP model, this is captured by a strong loading or ‘weight’ on the Sustained regressor and nil loadings on the Onset and Phasic regressors. **d.** In the CB model, the same pattern of weights is instead associated with a transient increase in hemodynamic signal in the middle of the anticipation epoch. **f.** In the CB model, sustained activation is captured by uniformly strong loadings across the early, middle, and late regressors. **e.** In the OSP model, this pattern of weights is associated with transient onset and phasic responses, superimposed on a large sustained response. In effect, the Onset and Phasic regressors serve to modulate the leading and trailing edges of a sustained wave of activation. It also merits comment that, although the three regression weights are equally strong (*O* ≈ *S* ≈ *P*), the momentary height of the hemodynamic signal is not (*O* > *S* < *P*). This reflects the fact that the OSP model casts the threat-anticipation signal as the linear combination of 3 temporally overlapping regressors. **g.** Likewise, in the OSP model the relative height of activation (*S* < *P*) can be reversed from the rank order of the fitted regression weights (*S* > *P*). **h.** In contrast, the CB model provides a one-to-one mapping between the momentary height of the observed hemodynamic signal and the early, middle, and late regression weights, enabling straightforward statistical comparisons across moments in time. Abbreviations—BOLD, blood-oxygenation-level-dependent; CB, Convolved Blocks model; HRF, hemodynamic response function; OSP, Onset-Sustained-Phasic; s, seconds. 

**Supplementary Figure S4. The dorsal amygdala is sensitive to the onset of the threat-anticipation epoch, independent of temporal certainty.** Exploratory analyses of the OSP Onset regressor revealed significant responses to both certain- and uncertain-threat anticipation in the right dorsal amygdala in the region of the basal and cortical nuclei. Figure depicts the minimum conjunction (logical ‘AND’) of certain and uncertain threat relative to their respective control conditions (FDR *q*<0.05, whole-brain corrected).    Note: 4-mm smoothing kernel. Abbreviations—FDR, False discovery rate; L, left; t, Student’s *t*-test; WB, whole-brain corrected. 

**Supplementary Figure S5. Statistically interrogating time-varying responses to certain-and-imminent threat.** Regions showing significant activation during the third 6.25-s block of certain-threat anticipation (*left column*) and uncertain-threat anticipation (*middle column*) compared to the second block (FDR *q*<0.05, whole-brain corrected). The *right column* depicts regions where surges in activation are significantly stronger for certain compared to uncertain threat (equivalent to testing the Block × Threat Certainty interaction). Results revealed significant activation surges in every key region except the PAG (*left column*), with a similar pattern evident for the between-moments comparison of certain to uncertain threat anticipation (*right column*).  Note: 4-mm smoothing kernel. Abbreviations—Ant., anterior; BST, bed nucleus of the stria terminalis; dlPFC, dorsolateral prefrontal cortex; FDR, false discovery rate; FrO, frontal operculum; L, left; PAG, periaqueductal gray; t, Student’s *t*-test; WB, whole-brain corrected.

**Supplementary Figure S6. The BST and Ce show statistically similar responses to uncertain- and certain-threat anticipation. a.** ***Uncertain-threat anticipation, third convolved block.*** The BST and Ce show negligible differences in activation during the third block (12.5-18.75 s) of uncertain-threat anticipation **c.** ***Certain-threat anticipation, second convolved block.*** The BST and Ce show modest differences during the middle (6.25-12.5 s) of certain-threat anticipation. Raincloud plots indicate the medians (*horizontal lines*), interquartile ranges (*boxes*), and smoothed density distributions. Whiskers depict 1.5× the interquartile range. Colored dots connected by gray lines indicate mean regional for each participant. Note: No spatial smoothing kernel was employed for ROI analyses. Abbreviations—BF, Bayes’ factor; BST, bed nucleus of the stria terminalis; Ce, central nucleus of the amygdala; *d*, Cohen’s *dz*; EAc, central extended amygdala; *t*, Student’s *t*-test.

# Supplementary Table S1. [UT > US]. Descriptive statistics for clusters and local maxima from the conventional ‘boxcar’ analysis showing greater activation for Uncertain-Threat relative to Uncertain-Safety anticipation (FDR *q*<0.05, whole-brain corrected). 

| **mm**^**3**^ | **Label** | ***t*** | ***x*** | ***y*** | ***z*** |
| --- | --- | --- | --- | --- | --- |
| 113,264 |  |  |  |  |  |
|  | R Accumbens | 3.83 | 10 | 6 | -8 |
|  | L Caudate | 7.17 | -16 | 4 | 22 |
|  | L Bed Nucleus of Stria Terminalis | 4.79 | -6 | 2 | -4 |
|  | R Caudate | 8.48 | 18 | -2 | 22 |
|  | R Thalamus | 7.03 | 10 | -2 | 10 |
|  | L Thalamus | 6.24 | -6 | -4 | 6 |
|  | R Left Thalamus | 6.42 | 0 | -4 | 6 |
|  | R Brain-Stem | 10.39 | 2 | -30 | -2 |
|  | L Brain-Stem | 6.91 | -4 | -34 | -26 |
|  | R Temporal Occipital Fusiform Cortex | 10.16 | 34 | -60 | -20 |
|  | L Lingual Gyrus | 4.11 | -10 | -86 | -10 |
|  | R Occipital Fusiform Gyrus | 4.05 | 14 | -86 | -12 |
|  | R Lingual Gyrus | 3.77 | 6 | -86 | -10 |
|  | R Occipital Pole | 3.66 | 10 | -92 | -12 |
| 66,328 |  |  |  |  |  |
|  | R Frontal Pole | 3.26 | 6 | 60 | 32 |
|  | L Cingulate Gyrus, anterior division | 11.03 | -8 | 20 | 32 |
|  | R Paracingulate Gyrus | 13.55 | 8 | 20 | 36 |
|  | R Cingulate Gyrus, anterior division | 12.95 | 10 | 12 | 38 |
|  | L Paracingulate Gyrus | 11.54 | -2 | 10 | 42 |
|  | R Middle Frontal Gyrus | 3.75 | 34 | 10 | 32 |
|  | L Superior Frontal Gyrus | 11.38 | -14 | 4 | 70 |
|  | L Juxtapositional Lobule Cortex (formerly Supplementary Motor Cortex) | 10.42 | -4 | 4 | 46 |
|  | R Juxtapositional Lobule Cortex (formerly Supplementary Motor Cortex) | 12.73 | 8 | 4 | 54 |
|  | R Superior Frontal Gyrus | 11.75 | 18 | 4 | 68 |
|  | R Precentral Gyrus | 11.85 | 46 | 0 | 50 |
|  | L Precentral Gyrus | 7.67 | -40 | -2 | 48 |
|  | R Cingulate Gyrus, posterior division | 6.35 | 8 | -20 | 42 |
| 26,672 |  |  |  |  |  |
|  | R Frontal Pole | 6.28 | 52 | 36 | -6 |
|  | R Frontal Orbital Cortex | 15.4 | 34 | 28 | 2 |
|  | R Frontal Operculum Cortex | 14.49 | 42 | 22 | 4 |
|  | R Inferior Frontal Gyrus, pars opercularis | 10.21 | 54 | 12 | 2 |
|  | R Precentral Gyrus | 3.32 | 60 | 12 | 24 |
|  | R Putamen | 10.67 | 20 | 10 | -6 |
|  | R Central Opercular Cortex | 7.49 | 46 | 2 | 10 |
|  | R Pallidum | 5.72 | 14 | -2 | -6 |
|  | R Amygdala | 5.59 | 30 | -8 | -14 |
|  | R Amygdala | 6.36 | 26 | -10 | -12 |
| 22,136 |  |  |  |  |  |
|  | R Parietal Operculum Cortex | 6.49 | 54 | -26 | 24 |
|  | R Middle Temporal Gyrus, posterior division | 10.82 | 52 | -30 | -4 |
|  | R Supramarginal Gyrus, anterior division | 6.57 | 56 | -32 | 34 |
|  | R Middle Temporal Gyrus, temporooccipital part | 7.99 | 56 | -40 | 4 |
|  | R Supramarginal Gyrus, posterior division | 11.5 | 66 | -42 | 28 |
|  | R Angular Gyrus | 6.26 | 56 | -48 | 48 |
| 20,736 |  |  |  |  |  |
|  | L Frontal Operculum Cortex | 14.26 | -32 | 24 | 8 |
|  | L Frontal Orbital Cortex | 10.07 | -30 | 22 | -8 |
|  | L Caudate | 4.17 | -16 | 18 | 4 |
|  | L Putamen | 9.33 | -20 | 10 | -2 |
|  | L Inferior Frontal Gyrus, pars opercularis | 9.06 | -54 | 10 | 6 |
|  | L Central Opercular Cortex | 9.21 | -48 | 4 | 2 |
|  | L Hippocampus | 3.39 | -30 | -18 | -12 |
| 11,008 |  |  |  |  |  |
|  | L Postcentral Gyrus | 4.23 | -56 | -22 | 24 |
|  | L Supramarginal Gyrus, anterior division | 4.58 | -62 | -28 | 20 |
|  | L Supramarginal Gyrus, posterior division | 8.96 | -54 | -42 | 32 |
| 9,696 |  |  |  |  |  |
|  | L Frontal Pole | 9.94 | -30 | 52 | 28 |
|  | L Middle Frontal Gyrus | 6.82 | -36 | 32 | 38 |
| 7,216 |  |  |  |  |  |
|  | R Frontal Pole | 8.17 | 32 | 52 | 30 |
| 3,680 |  |  |  |  |  |
|  | R Lateral Occipital Cortex, inferior division | 3.66 | 42 | -82 | -14 |
|  | R Occipital Pole | 6.62 | 24 | -96 | 14 |
| 3,608 |  |  |  |  |  |
|  | R Postcentral Gyrus | 2.75 | 32 | -34 | 46 |
|  | R Superior Parietal Lobule | 6.73 | 26 | -44 | 66 |
| 2,496 |  |  |  |  |  |
|  | R Temporal Pole | 4.73 | 48 | 10 | -40 |
| 1,384 |  |  |  |  |  |
|  | L Temporal Pole | 4.73 | -42 | 6 | -44 |
|  | L Middle Temporal Gyrus, anterior division | 3.91 | -50 | -2 | -32 |
|  | L Inferior Temporal Gyrus, anterior division | 3.08 | -50 | -2 | -44 |
| 792 |  |  |  |  |  |
|  | R Postcentral Gyrus | 4.34 | 46 | -24 | 44 |
| 784 |  |  |  |  |  |
|  | R Occipital Pole | 3.62 | -18 | -100 | 8 |
| 712 |  |  |  |  |  |
|  | R Frontal Pole | 4.61 | 28 | 58 | -8 |
| 552 |  |  |  |  |  |
|  | L Superior Parietal Lobule | 4.46 | -22 | -50 | 64 |
| 376 |  |  |  |  |  |
|  | L Cingulate Gyrus, posterior division | 4.66 | -10 | -22 | 40 |
| 344 |  |  |  |  |  |
|  | R Hippocampus | 4.36 | 34 | -28 | -6 |
| 280 |  |  |  |  |  |
|  | L Pallidum | 3.86 | -16 | -6 | -2 |
| 176 |  |  |  |  |  |
|  | R Parahippocampal Gyrus, anterior division | 3.99 | 24 | -8 | -32 |
| 128 |  |  |  |  |  |
|  | L Middle Temporal Gyrus, posterior division | 3.19 | -48 | -28 | -6 |
| 120 |  |  |  |  |  |
|  | L Frontal Pole | 3.13 | -30 | 54 | -12 |
| 112 |  |  |  |  |  |
|  | R Thalamus | 4.62 | 8 | -30 | 12 |
| 104 |  |  |  |  |  |
|  | L Cingulate Gyrus, posterior division | 3.48 | -2 | -18 | 30 |
| 88 |  |  |  |  |  |
|  | L Subcallosal Cortex | 3.99 | -10 | 16 | -22 |
| 80 |  |  |  |  |  |
|  | L Thalamus | 4.23 | -6 | -26 | 14 |
| 72 |  |  |  |  |  |
|  | R Inferior Temporal Gyrus, posterior division | 3.32 | 52 | -34 | -28 |
| 56 |  |  |  |  |  |
|  | L Temporal Pole | 3.1 | -36 | 22 | -38 |
| 48 |  |  |  |  |  |
|  | L Temporal Pole | 2.87 | -32 | 16 | -44 |
| 48 |  |  |  |  |  |
|  | L Middle Temporal Gyrus, temporooccipital part | 2.62 | -54 | -46 | 2 |
| 40 |  |  |  |  |  |
|  | L Temporal Pole | 2.65 | -28 | 22 | -34 |
| 32 |  |  |  |  |  |
|  | L Temporal Pole | 3.64 | -32 | 12 | -34 |
| 32 |  |  |  |  |  |
|  | R Thalamus | 3.15 | 22 | -34 | 0 |
| 32 |  |  |  |  |  |
|  | Postcentral Gyrus | 2.57 | -42 | -32 | 44 |
| 32 |  |  |  |  |  |
|  | L Precuneus Cortex | 3.33 | -12 | -46 | 48 |
| 32 |  |  |  |  |  |
|  | L Precuneus Cortex | 2.55 | -10 | -54 | 60 |
| 32 |  |  |  |  |  |
|  | L Superior Parietal Lobule | 2.9 | -32 | -48 | 70 |
| 24 |  |  |  |  |  |
|  | L Temporal Pole | 2.41 | -20 | 2 | -40 |
| 24 |  |  |  |  |  |
|  | L Frontal Orbital Cortex | 2.95 | -22 | 18 | -24 |
| 24 |  |  |  |  |  |
|  | R Frontal Orbital Cortex | 2.58 | 12 | 18 | -22 |
| 24 |  |  |  |  |  |
|  | L Temporal Pole | 2.69 | -48 | 18 | -18 |
| 16 |  |  |  |  |  |
|  | L Temporal Pole | 2.48 | -28 | 6 | -48 |
| 16 |  |  |  |  |  |
|  | R Inferior Temporal Gyrus, anterior division | 2.44 | 48 | -6 | -42 |
| 16 |  |  |  |  |  |
|  | R Brain-Stem | 2.64 | 18 | -32 | -38 |
| 16 |  |  |  |  |  |
|  | R Inferior Temporal Gyrus, temporooccipital part | 2.93 | 58 | -40 | -20 |
| 16 |  |  |  |  |  |
|  | R Lateral Occipital Cortex, inferior division | 2.86 | 42 | -86 | -8 |
| 16 |  |  |  |  |  |
|  | L Middle Temporal Gyrus, posterior division | 2.67 | -58 | -28 | -6 |
| 16 |  |  |  |  |  |
|  | R Putamen | 3.43 | 32 | -18 | -2 |
| 16 |  |  |  |  |  |
|  | R Supramarginal Gyrus, anterior division | 3.36 | 52 | -22 | 32 |
| 8 |  |  |  |  |  |
|  | R Temporal Fusiform Cortex, anterior division | 2.51 | 26 | -4 | -42 |
| 8 |  |  |  |  |  |
|  | L Temporal Pole | 2.31 | -28 | 16 | -36 |
| 8 |  |  |  |  |  |
|  | L Inferior Temporal Gyrus, posterior division | 2.56 | -50 | -28 | -24 |
| 8 |  |  |  |  |  |
|  | L Inferior Temporal Gyrus, posterior division | 2.51 | -50 | -38 | -20 |
| 8 |  |  |  |  |  |
|  | R Cingulate Gyrus, posterior division | 2.79 | 52 | 8 | -20 |
| 8 |  |  |  |  |  |
|  | R Occipital Pole | 2.31 | 20 | -98 | -16 |
| 8 |  |  |  |  |  |
|  | L Frontal Orbital Cortex | 2.53 | -20 | 24 | -16 |
| 8 |  |  |  |  |  |
|  | L Middle Temporal Gyrus, posterior division | 2.38 | -56 | -32 | -12 |
| 8 |  |  |  |  |  |
|  | L Frontal Pole | 2.36 | -22 | 54 | -12 |
| 8 |  |  |  |  |  |
|  | R Frontal Pole | 2.58 | 36 | 54 | -8 |
| 8 |  |  |  |  |  |
|  | L Thalamus | 2.84 | -20 | -36 | 0 |
| 8 |  |  |  |  |  |
|  | L Paracingulate Gyrus | 2.49 | -10 | 54 | 12 |
| 8 |  |  |  |  |  |
|  | R Inferior Frontal Gyrus, pars opercularis | 2.32 | 52 | 20 | 22 |

# Supplementary Table S2. [CT > CS]. Descriptive statistics for clusters and local maxima from the conventional ‘boxcar’ analysis showing greater activation for Certain-Threat relative to Certain-Safety anticipation (FDR *q*<0.05, whole-brain corrected). 

| **mm**^**3**^ | **Label** | ***t*** | ***x*** | ***y*** | ***z*** |
| --- | --- | --- | --- | --- | --- |
| 65,384 |  |  |  |  |  |
|  | R Brain-Stem | 5.78 | 12 | -28 | -16 |
|  | L Brain-Stem | 4.15 | -4 | -40 | -22 |
|  | R Lingual Gyrus | 6.87 | 6 | -72 | -10 |
| 25,304 |  |  |  |  |  |
|  | L Paracingulate Gyrus | 7.36 | -8 | 20 | 34 |
|  | R Paracingulate Gyrus | 6.75 | 10 | 18 | 36 |
|  | R Juxtapositional Lobule Cortex (formerly Supplementary Motor Cortex) | 6.99 | 8 | 6 | 64 |
|  | L Superior Frontal Gyrus | 6.64 | -16 | 4 | 68 |
|  | L Juxtapositional Lobule Cortex (formerly Supplementary Motor Cortex) | 6.26 | -6 | 2 | 52 |
|  | R Superior Frontal Gyrus | 7.79 | 14 | 2 | 68 |
|  | R Precentral Gyrus | 6.68 | 48 | 2 | 52 |
|  | R Middle Frontal Gyrus | 4.52 | 38 | 0 | 62 |
| 10,168 |  |  |  |  |  |
|  | R Frontal Pole | 4.23 | 52 | 46 | 0 |
|  | R Inferior Frontal Gyrus, pars triangularis | 4.94 | 50 | 32 | -2 |
|  | R Frontal Orbital Cortex | 6.09 | 40 | 28 | 0 |
|  | R Temporal Pole | 3.56 | 50 | 20 | -16 |
|  | R Frontal Operculum Cortex | 8.32 | 48 | 18 | 0 |
| 10,000 |  |  |  |  |  |
|  | L Supramarginal Gyrus, anterior division | 2.82 | -50 | -30 | 38 |
|  | L Postcentral Gyrus | 4.46 | -36 | -38 | 42 |
|  | L Supramarginal Gyrus, posterior division | 6.65 | -56 | -52 | 40 |
|  | L Superior Parietal Lobule | 3.52 | -32 | -54 | 44 |
|  | L Angular Gyrus | 4.95 | -52 | -56 | 50 |
|  | L Lateral Occipital Cortex, superior division | 3.31 | -34 | -62 | 54 |
| 9,296 |  |  |  |  |  |
|  | L Frontal Pole | 6.84 | -40 | 54 | 14 |
|  | L Middle Frontal Gyrus | 5.39 | -34 | 30 | 38 |
| 9,192 |  |  |  |  |  |
|  | R Postcentral Gyrus | 4.25 | 40 | -28 | 42 |
|  | R Supramarginal Gyrus, anterior division | 3.46 | 50 | -28 | 42 |
|  | R Supramarginal Gyrus, posterior division | 6.52 | 64 | -40 | 26 |
|  | R Superior Parietal Lobule | 3.71 | 36 | -44 | 58 |
|  | R Angular Gyrus | 5.64 | 52 | -46 | 34 |
| 7,016 |  |  |  |  |  |
|  | L Frontal Orbital Cortex | 5.51 | -34 | 28 | 0 |
|  | L Insular Cortex | 4.91 | -32 | 26 | 4 |
|  | L Inferior Frontal Gyrus, pars opercularis | 5.49 | -54 | 18 | 0 |
|  | L Frontal Operculum Cortex | 8.84 | -40 | 14 | 2 |
|  | L Precentral Gyrus | 3.05 | -56 | 8 | 22 |
| 6,936 |  |  |  |  |  |
|  | R Frontal Pole | 6.91 | 30 | 50 | 28 |
|  | R Middle Frontal Gyrus | 3.27 | 36 | 34 | 36 |
| 5,232 |  |  |  |  |  |
|  | R Accumbens | 4.2 | 10 | 12 | -4 |
|  | R Putamen | 6.58 | 22 | 10 | -4 |
|  | R Insular Cortex | 3.1 | 38 | 10 | -6 |
|  | L Bed Nucleus of Stria Terminalis | 4.4 | -6 | 4 | 0 |
|  | L Caudate | 3.12 | -8 | 2 | 6 |
|  | L Thalamus | 4.48 | -2 | -2 | 0 |
|  | R Left Thalamus | 6.03 | 0 | -4 | 6 |
|  | R Thalamus | 6.7 | 2 | -18 | 0 |
|  | L Brain-Stem | 5.16 | -2 | -30 | -4 |
| 3,608 |  |  |  |  |  |
|  | R Middle Temporal Gyrus, posterior division | 4.19 | 60 | -24 | -6 |
|  | R Superior Temporal Gyrus, posterior division | 5.95 | 48 | -26 | -4 |
|  | R Middle Temporal Gyrus, temporooccipital part | 4.84 | 52 | -42 | 4 |
|  | R Supramarginal Gyrus, posterior division | 4.19 | 48 | -42 | 12 |
| 2,904 |  |  |  |  |  |
|  | R Brain-Stem | 6.14 | 6 | -46 | -58 |
|  | L Brain-Stem | 4.33 | -6 | -48 | -58 |
| 1,984 |  |  |  |  |  |
|  | L Caudate | 3.62 | -18 | 20 | -2 |
|  | L Putamen | 6 | -22 | 16 | -4 |
| 1,744 |  |  |  |  |  |
|  | R Lateral Occipital Cortex, inferior division | 3.83 | 30 | -86 | 0 |
|  | R Occipital Pole | 5.12 | 22 | -102 | 12 |
| 1,048 |  |  |  |  |  |
|  | R Caudate | 6.9 | 18 | 8 | 20 |
| 848 |  |  |  |  |  |
|  | L Precentral Gyrus | 4.58 | -48 | 4 | 42 |
| 640 |  |  |  |  |  |
|  | L Frontal Pole | 4 | -32 | 48 | -16 |
| 632 |  |  |  |  |  |
|  | R Hippocampus | 4.45 | 24 | -32 | -4 |
|  | R Thalamus | 5.17 | 12 | -34 | 8 |
| 576 |  |  |  |  |  |
|  | L Caudate | 5.65 | -16 | 2 | 22 |
| 368 |  |  |  |  |  |
|  | L Middle Frontal Gyrus | 3.24 | -26 | -4 | 50 |
|  | L Precentral Gyrus | 3.93 | -28 | -8 | 48 |
| 360 |  |  |  |  |  |
|  | R Amygdala | 3.79 | 30 | -4 | -20 |
|  | R Amygdala | 4.93 | 30 | -8 | -16 |
|  | R Amygdala | 4.79 | 28 | -12 | -14 |
| 240 |  |  |  |  |  |
|  | R Lateral Occipital Cortex, inferior division | 3.65 | 48 | -72 | -12 |
| 200 |  |  |  |  |  |
|  | L Thalamus | 4.43 | -20 | -36 | 2 |
| 192 |  |  |  |  |  |
|  | L Amygdala | 4.43 | -24 | -12 | -10 |
|  | L Amygdala | 3.53 | -24 | -12 | -14 |
|  | L Hippocampus | 3.08 | -30 | -16 | -14 |
| 192 |  |  |  |  |  |
|  | L Caudate | 4.69 | -18 | 16 | 12 |
| 168 |  |  |  |  |  |
|  | L Brain-Stem | 4.24 | -14 | -26 | -14 |
| 160 |  |  |  |  |  |
|  | L Thalamus | 4.33 | -12 | -10 | 6 |
| 152 |  |  |  |  |  |
|  | L Precuneus Cortex | 2.71 | -10 | -72 | 44 |
| 136 |  |  |  |  |  |
|  | R Cingulate Gyrus, anterior division | 3.53 | 4 | 16 | 26 |
|  | L Cingulate Gyrus, anterior division | 3.54 | -2 | 14 | 26 |
| 136 |  |  |  |  |  |
|  | R Precuneus Cortex | 3.44 | 12 | -68 | 48 |
|  | R Lateral Occipital Cortex, superior division | 3.24 | 14 | -70 | 52 |
| 128 |  |  |  |  |  |
|  | L Frontal Pole | 3.61 | -52 | 44 | 0 |
| 104 |  |  |  |  |  |
|  | L Brain-Stem | 3.57 | -6 | -38 | -30 |
| 96 |  |  |  |  |  |
|  | L Inferior Temporal Gyrus, posterior division | 4.13 | -54 | -36 | -18 |
| 80 |  |  |  |  |  |
|  | R Putamen | 3.38 | 28 | -2 | 12 |
| 72 |  |  |  |  |  |
|  | R Frontal Pole | 3.18 | 38 | 62 | -4 |
| 72 |  |  |  |  |  |
|  | L Caudate | 3.43 | -12 | 12 | 2 |
| 72 |  |  |  |  |  |
|  | R Caudate | 3.04 | 10 | 2 | 12 |
|  | R Thalamus | 2.59 | 10 | -2 | 10 |
| 72 |  |  |  |  |  |
|  | R Precuneus Cortex | 2.82 | 16 | -70 | 38 |
| 64 |  |  |  |  |  |
|  | L Pallidum | 3.5 | -14 | -2 | -4 |
| 64 |  |  |  |  |  |
|  | R Precentral Gyrus | 3.1 | 38 | -22 | 62 |
| 56 |  |  |  |  |  |
|  | L Temporal Fusiform Cortex, anterior division | 4.28 | -32 | 0 | -50 |
| 56 |  |  |  |  |  |
|  | R Frontal Pole | 3.33 | 26 | 60 | -12 |
| 56 |  |  |  |  |  |
|  | L Caudate | 2.66 | -18 | -14 | 24 |
| 56 |  |  |  |  |  |
|  | R Superior Parietal Lobule | 3.05 | 26 | -48 | 62 |
| 48 |  |  |  |  |  |
|  | L Brain-Stem | 2.61 | -12 | -16 | -22 |
| 48 |  |  |  |  |  |
|  | R Hippocampus/Amygdala | 2.76 | 16 | -12 | -16 |
| 48 |  |  |  |  |  |
|  | R Hippocampus | 3.85 | 36 | -32 | -6 |
| 48 |  |  |  |  |  |
|  | L Lateral Occipital Cortex, inferior division | 3.62 | -42 | -80 | -4 |
| 48 |  |  |  |  |  |
|  | R Inferior Frontal Gyrus, pars opercularis | 3.06 | 56 | 20 | 14 |
| 48 |  |  |  |  |  |
|  | L Cingulate Gyrus, anterior division | 3.22 | -4 | 22 | 20 |
| 40 |  |  |  |  |  |
|  | L Brain-Stem | 4.11 | -10 | -34 | -14 |
| 40 |  |  |  |  |  |
|  | R Frontal Pole | 3.46 | 32 | 60 | -6 |
| 40 |  |  |  |  |  |
|  | L Frontal Pole | 3.19 | -32 | 64 | 6 |
| 40 |  |  |  |  |  |
|  | R Thalamus | 3.37 | 12 | -10 | 8 |
| 40 |  |  |  |  |  |
|  | R Frontal Pole | 2.78 | 8 | 62 | 26 |
| 40 |  |  |  |  |  |
|  | R Middle Frontal Gyrus | 2.89 | 40 | 18 | 34 |
| 32 |  |  |  |  |  |
|  | R Inferior Temporal Gyrus, temporooccipital part | 2.88 | 58 | -58 | -18 |
| 32 |  |  |  |  |  |
|  | L Thalamus | 2.94 | -4 | -28 | 6 |
| 32 |  |  |  |  |  |
|  | L Precentral Gyrus | 2.88 | -48 | 2 | 20 |
| 32 |  |  |  |  |  |
|  | L Supramarginal Gyrus, anterior division | 2.96 | -54 | -34 | 36 |
| 32 |  |  |  |  |  |
|  | L Superior Frontal Gyrus | 2.9 | -10 | 32 | 52 |
| 32 |  |  |  |  |  |
|  | R Frontal Pole | 2.87 | 10 | 40 | 56 |
| 32 |  |  |  |  |  |
|  | L Precentral Gyrus | 2.65 | -32 | -12 | 64 |
| 24 |  |  |  |  |  |
|  | L Subcallosal Cortex | 2.8 | -8 | 28 | -24 |
| 24 |  |  |  |  |  |
|  | R Inferior Temporal Gyrus, temporooccipital part | 2.87 | 56 | -40 | -18 |
| 24 |  |  |  |  |  |
|  | R Frontal Pole | 2.9 | 36 | 52 | -14 |
| 24 |  |  |  |  |  |
|  | L Frontal Pole | 2.97 | -50 | 46 | -12 |
| 24 |  |  |  |  |  |
|  | R Lateral Occipital Cortex, inferior division | 2.89 | 38 | -86 | -10 |
| 24 |  |  |  |  |  |
|  | R Hippocampus | 2.73 | 34 | -26 | -8 |
| 24 |  |  |  |  |  |
|  | L Middle Temporal Gyrus, posterior division | 2.57 | -70 | -28 | -4 |
| 24 |  |  |  |  |  |
|  | L Occipital Pole | 2.71 | -28 | -92 | 0 |
|  | L Lateral Occipital Cortex, inferior division | 2.61 | -30 | -90 | -2 |
| 24 |  |  |  |  |  |
|  | R Inferior Frontal Gyrus, pars triangularis | 2.7 | 52 | 30 | 6 |
| 24 |  |  |  |  |  |
|  | L Cingulate Gyrus, posterior division | 2.74 | -2 | -18 | 32 |
| 24 |  |  |  |  |  |
|  | R Superior Parietal Lobule | 2.62 | 30 | -48 | 46 |
| 24 |  |  |  |  |  |
|  | L Lateral Occipital Cortex, superior division | 2.75 | -26 | -64 | 62 |
| 16 |  |  |  |  |  |
|  | R Temporal Pole | 2.85 | 34 | 20 | -46 |
| 16 |  |  |  |  |  |
|  | R Temporal Pole | 2.99 | 28 | 22 | -40 |
| 16 |  |  |  |  |  |
|  | R Temporal Pole | 2.73 | 22 | 16 | -38 |
| 16 |  |  |  |  |  |
|  | L Temporal Pole | 2.61 | -50 | 12 | -36 |
| 16 |  |  |  |  |  |
|  | R Temporal Pole | 3.05 | 30 | 24 | -34 |
| 16 |  |  |  |  |  |
|  | L Temporal Pole | 3.26 | -26 | 18 | -34 |
| 16 |  |  |  |  |  |
|  | R Brain-Stem | 2.89 | 0 | -38 | -30 |
| 16 |  |  |  |  |  |
|  | R Brain-Stem | 2.6 | 8 | -38 | -28 |
| 16 |  |  |  |  |  |
|  | L Middle Temporal Gyrus, anterior division | 2.98 | -54 | -8 | -26 |
| 16 |  |  |  |  |  |
|  | R Frontal Pole | 2.82 | 8 | 46 | -28 |
| 16 |  |  |  |  |  |
|  | R Frontal Orbital Cortex | 2.99 | 38 | 20 | -16 |
| 16 |  |  |  |  |  |
|  | L Occipital Fusiform Gyrus | 2.77 | -14 | -86 | -12 |
| 16 |  |  |  |  |  |
|  | L Middle Temporal Gyrus, posterior division | 3.15 | -62 | -22 | -12 |
| 16 |  |  |  |  |  |
|  | L Putamen | 2.73 | -32 | -14 | -10 |
| 16 |  |  |  |  |  |
|  | L Middle Temporal Gyrus, posterior division | 2.88 | -54 | -32 | -6 |
| 16 |  |  |  |  |  |
|  | L Pallidum | 2.85 | -20 | -10 | -4 |
| 16 |  |  |  |  |  |
|  | R Frontal Pole | 2.76 | 26 | 52 | -2 |
| 16 |  |  |  |  |  |
|  | L Frontal Pole | 2.65 | -34 | 60 | 2 |
| 16 |  |  |  |  |  |
|  | R Supramarginal Gyrus, posterior division | 2.86 | 58 | -42 | 14 |
| 16 |  |  |  |  |  |
|  | L Inferior Frontal Gyrus, pars triangularis | 3.18 | -50 | 24 | 14 |
| 16 |  |  |  |  |  |
|  | R Precentral Gyrus | 3.57 | 54 | 4 | 20 |
| 16 |  |  |  |  |  |
|  | R Lateral Occipital Cortex, superior division | 2.62 | 26 | -78 | 30 |
| 16 |  |  |  |  |  |
|  | L Lateral Occipital Cortex, superior division | 2.77 | -26 | -60 | 46 |
| 16 |  |  |  |  |  |
|  | R Superior Parietal Lobule | 2.75 | 30 | -56 | 50 |
| 16 |  |  |  |  |  |
|  | L Superior Frontal Gyrus | 2.66 | -24 | -8 | 60 |
| 16 |  |  |  |  |  |
|  | L Lateral Occipital Cortex, superior division | 3.02 | -24 | -64 | 68 |
| 16 |  |  |  |  |  |
|  | L Precentral Gyrus | 2.59 | -34 | -6 | 66 |
| 8 |  |  |  |  |  |
|  | R Brain-Stem | 2.57 | 0 | -32 | -60 |
| 8 |  |  |  |  |  |
|  | L Brain-Stem | 2.6 | -2 | -36 | -56 |
| 8 |  |  |  |  |  |
|  | R Brain-Stem | 2.58 | 4 | -38 | -40 |
| 8 |  |  |  |  |  |
|  | L Temporal Pole | 2.52 | -38 | 22 | -40 |
| 8 |  |  |  |  |  |
|  | R Temporal Fusiform Cortex, posterior division | 2.59 | 36 | -18 | -32 |
| 8 |  |  |  |  |  |
|  | L Inferior Temporal Gyrus, posterior division | 2.63 | -62 | -24 | -30 |
| 8 |  |  |  |  |  |
|  | L Brain-Stem | 2.75 | -6 | -20 | -24 |
| 8 |  |  |  |  |  |
|  | L Parahippocampal Gyrus, anterior division | 2.57 | -12 | -4 | -24 |
| 8 |  |  |  |  |  |
|  | L Frontal Orbital Cortex | 2.95 | -32 | 24 | -24 |
| 8 |  |  |  |  |  |
|  | R Temporal Occipital Fusiform Cortex | 2.55 | 48 | -54 | -22 |
| 8 |  |  |  |  |  |
|  | L Temporal Pole | 2.64 | -50 | 16 | -18 |
| 8 |  |  |  |  |  |
|  | R Inferior Temporal Gyrus, temporooccipital part | 2.6 | 62 | -46 | -16 |
| 8 |  |  |  |  |  |
|  | L Middle Temporal Gyrus, posterior division | 2.6 | -56 | -26 | -16 |
| 8 |  |  |  |  |  |
|  | L Insular Cortex | 2.89 | -28 | 14 | -12 |
| 8 |  |  |  |  |  |
|  | L Middle Temporal Gyrus, posterior division | 2.85 | -70 | -26 | -10 |
| 8 |  |  |  |  |  |
|  | L Lingual Gyrus | 2.58 | -12 | -84 | -8 |
| 8 |  |  |  |  |  |
|  | R Frontal Pole | 2.59 | 28 | 58 | -8 |
| 8 |  |  |  |  |  |
|  | R Thalamus | 2.55 | 6 | -10 | 0 |
| 8 |  |  |  |  |  |
|  | L Caudate | 2.54 | -8 | 10 | 6 |
| 8 |  |  |  |  |  |
|  | L Thalamus | 2.84 | -8 | -34 | 8 |
| 8 |  |  |  |  |  |
|  | L Inferior Frontal Gyrus, pars triangularis | 2.6 | -54 | 22 | 8 |
| 8 |  |  |  |  |  |
|  | L Occipital Pole | 2.7 | -20 | -102 | 10 |
| 8 |  |  |  |  |  |
|  | L Occipital Pole | 2.53 | -26 | -98 | 12 |
| 8 |  |  |  |  |  |
|  | R Central Opercular Cortex | 2.63 | 46 | 2 | 12 |
| 8 |  |  |  |  |  |
|  | L Caudate | 2.82 | -8 | 4 | 12 |
| 8 |  |  |  |  |  |
|  | R Supramarginal Gyrus, posterior division | 2.6 | 66 | -38 | 14 |
| 8 |  |  |  |  |  |
|  | R Frontal Pole | 2.51 | 46 | 46 | 16 |
| 8 |  |  |  |  |  |
|  | R Caudate | 2.72 | 18 | -18 | 22 |
| 8 |  |  |  |  |  |
|  | R Cingulate Gyrus, anterior division | 2.65 | 6 | 20 | 22 |
| 8 |  |  |  |  |  |
|  | R Frontal Pole | 3.18 | 44 | 36 | 24 |
| 8 |  |  |  |  |  |
|  | R Lateral Occipital Cortex, superior division | 2.78 | 28 | -74 | 26 |
| 8 |  |  |  |  |  |
|  | R Cingulate Gyrus, posterior division | 2.76 | 6 | -24 | 28 |
| 8 |  |  |  |  |  |
|  | L Angular Gyrus | 2.76 | -58 | -60 | 30 |
| 8 |  |  |  |  |  |
|  | R Lateral Occipital Cortex, superior division | 2.88 | 26 | -72 | 32 |
| 8 |  |  |  |  |  |
|  | R Superior Frontal Gyrus | 2.54 | 6 | 56 | 32 |
| 8 |  |  |  |  |  |
|  | R Superior Frontal Gyrus | 2.72 | 8 | 48 | 34 |
| 8 |  |  |  |  |  |
|  | R Precuneus Cortex | 2.9 | 8 | -42 | 46 |
| 8 |  |  |  |  |  |
|  | L Middle Frontal Gyrus | 2.53 | -40 | 0 | 52 |
| 8 |  |  |  |  |  |
|  | L Precentral Gyrus | 2.67 | -40 | -4 | 56 |
| 8 |  |  |  |  |  |
|  | L Superior Frontal Gyrus | 2.61 | -20 | -8 | 60 |
| 8 |  |  |  |  |  |
|  | R Superior Parietal Lobule | 2.52 | 14 | -52 | 76 |

# Supplementary Table S3. [UT > US] $\boldsymbol{∩}$  [CT > CS]. Descriptive statistics for clusters and local maxima from the conventional ‘boxcar’ analysis showing greater activation for Uncertain-Threat relative to Uncertain-Safety anticipation AND greater activation for Certain-Threat relative to Certain-Safety anticipation (FDR *q*<0.05, whole-brain corrected). 

| ***mm***^***3***^ | **Label** | ***t*** | ***x*** | ***y*** | ***z*** |
| --- | --- | --- | --- | --- | --- |
| 47,064 |  |  |  |  |  |
|  | L Brain-Stem | 3.80 | -4 | -40 | -22 |
|  | R Brain-Stem | 2.89 | 6 | -42 | -26 |
| 24,856 |  |  |  |  |  |
|  | L Paracingulate Gyrus | 7.37 | -8 | 20 | 34 |
|  | R Paracingulate Gyrus | 6.76 | 10 | 18 | 36 |
|  | R Juxtapositional Lobule Cortex (formerly Supplementary Motor Cortex) | 6.99 | 8 | 6 | 64 |
|  | L Superior Frontal Gyrus | 6.64 | -16 | 4 | 68 |
|  | L Juxtapositional Lobule Cortex (formerly Supplementary Motor Cortex) | 6.27 | -6 | 2 | 52 |
|  | R Superior Frontal Gyrus | 7.79 | 14 | 2 | 68 |
|  | R Precentral Gyrus | 6.68 | 48 | 2 | 52 |
|  | R Middle Frontal Gyrus | 4.52 | 38 | 0 | 62 |
| 9,712 |  |  |  |  |  |
|  | R Frontal Pole | 3.82 | 54 | 40 | -2 |
|  | R Inferior Frontal Gyrus, pars triangularis | 4.94 | 50 | 32 | -2 |
|  | R Frontal Orbital Cortex | 6.09 | 40 | 28 | 0 |
|  | R Frontal Operculum Cortex | 8.33 | 48 | 18 | 0 |
| 7,320 |  |  |  |  |  |
|  | L Frontal Pole | 6.78 | -32 | 54 | 24 |
|  | L Middle Frontal Gyrus | 5.39 | -34 | 30 | 38 |
| 6,984 |  |  |  |  |  |
|  | L Frontal Orbital Cortex | 5.52 | -34 | 28 | 0 |
|  | L Insular Cortex | 4.91 | -32 | 26 | 4 |
|  | L Inferior Frontal Gyrus, pars opercularis | 5.49 | -54 | 18 | 0 |
|  | L Frontal Operculum Cortex | 8.85 | -40 | 14 | 2 |
|  | L Precentral Gyrus | 3.06 | -56 | 8 | 22 |
| 5,696 |  |  |  |  |  |
|  | R Frontal Pole | 6.91 | 30 | 50 | 28 |
|  | R Middle Frontal Gyrus | 3.28 | 36 | 34 | 36 |
| 5,264 |  |  |  |  |  |
|  | R Supramarginal Gyrus, posterior division | 6.53 | 64 | -40 | 26 |
|  | R Angular Gyrus | 5.64 | 52 | -46 | 34 |
| 5,152 |  |  |  |  |  |
|  | L Supramarginal Gyrus, posterior division | 6.66 | -56 | -52 | 40 |
| 3,528 |  |  |  |  |  |
|  | R Middle Temporal Gyrus, posterior division | 4.20 | 60 | -24 | -6 |
|  | R Superior Temporal Gyrus, posterior division | 5.96 | 48 | -26 | -4 |
|  | R Middle Temporal Gyrus, temporooccipital part | 4.73 | 52 | -42 | 4 |
|  | R Supramarginal Gyrus, posterior division | 4.19 | 48 | -42 | 12 |
| 2,648 |  |  |  |  |  |
|  | R Brain-Stem | 5.78 | 8 | -34 | -54 |
|  | L Brain-Stem | 4.33 | -6 | -48 | -58 |
| 2,360 |  |  |  |  |  |
|  | R Putamen | 6.59 | 22 | 10 | -4 |
| 1,584 |  |  |  |  |  |
|  | L Putamen | 5.55 | -20 | 14 | -4 |
| 1,424 |  |  |  |  |  |
|  | R Occipital Pole | 4.58 | 20 | -102 | 12 |
| 920 |  |  |  |  |  |
|  | R Caudate | 6.50 | 18 | 8 | 20 |
| 648 |  |  |  |  |  |
|  | L Thalamus | 3.63 | -2 | -18 | -2 |
|  | R Thalamus | 4.65 | 2 | -20 | 0 |
|  | L Brain-Stem | 5.16 | -2 | -30 | -4 |
| 552 |  |  |  |  |  |
|  | L Thalamus | 3.59 | -2 | -4 | -2 |
|  | R Thalamus | 6.04 | 0 | -4 | 6 |
| 536 |  |  |  |  |  |
|  | R Brain-Stem | 5.52 | 12 | -28 | -14 |
|  | L Periaqueductal gray | 3.26 | -2 | -36 | -14 |
|  | R Periaqueductal gray | 2.85 | 0 | -36 | -10 |
| 504 |  |  |  |  |  |
|  | R Postcentral Gyrus | 3.64 | 46 | -28 | 50 |
| 472 |  |  |  |  |  |
|  | L Precentral Gyrus | 4.12 | -48 | 2 | 40 |
| 448 |  |  |  |  |  |
|  | L Caudate | 5.65 | -16 | 2 | 22 |
| 328 |  |  |  |  |  |
|  | L Middle Frontal Gyrus | 3.25 | -26 | -4 | 50 |
|  | L Precentral Gyrus | 3.94 | -28 | -8 | 48 |
| 160 |  |  |  |  |  |
|  | R Superior Parietal Lobule | 3.33 | 34 | -42 | 62 |
| 152 |  |  |  |  |  |
|  | L Thalamus | 4.17 | -10 | -10 | 6 |
| 144 |  |  |  |  |  |
|  | R Amygdala | 3.61 | 30 | -6 | -16 |
|  | R Amygdala | 4.09 | 30 | -10 | -14 |
| 136 |  |  |  |  |  |
|  | L Brain-Stem | 4.24 | -14 | -26 | -14 |
| 104 |  |  |  |  |  |
|  | L Caudate | 3.13 | -8 | 2 | 6 |
|  | L Bed Nucleus of the Stria Terminalis | 3.62 | -6 | 2 | 0 |
| 104 |  |  |  |  |  |
|  | R Cingulate Gyrus, anterior division | 3.53 | 4 | 16 | 26 |
|  | L Cingulate Gyrus, anterior division | 3.54 | -2 | 14 | 26 |
| 96 |  |  |  |  |  |
|  | L Amygdala | 3.34 | -24 | -12 | -10 |
|  | L Amygdala | 2.92 | -26 | -14 | -14 |
|  | L Hippocampus | 2.92 | -26 | -14 | -14 |
| 80 |  |  |  |  |  |
|  | R Putamen | 3.39 | 28 | -2 | 12 |
| 72 |  |  |  |  |  |
|  | R Caudate | 3.04 | 10 | 2 | 12 |
|  | R Thalamus | 2.59 | 10 | -2 | 10 |
| 72 |  |  |  |  |  |
|  | L Precentral Gyrus | 3.33 | -48 | 0 | 54 |
| 56 |  |  |  |  |  |
|  | L Caudate | 2.67 | -18 | -14 | 24 |
| 56 |  |  |  |  |  |
|  | R Superior Parietal Lobule | 3.05 | 26 | -48 | 62 |
| 48 |  |  |  |  |  |
|  | L Brain-Stem | 3.57 | -6 | -38 | -30 |
| 48 |  |  |  |  |  |
|  | L Pallidum | 3.05 | -14 | -2 | -4 |
| 48 |  |  |  |  |  |
|  | R Thalamus | 3.10 | 2 | -14 | 14 |
| 48 |  |  |  |  |  |
|  | R Inferior Frontal Gyrus, pars opercularis | 3.06 | 56 | 20 | 14 |
| 48 |  |  |  |  |  |
|  | R Precentral Gyrus | 3.11 | 38 | -22 | 62 |
| 40 |  |  |  |  |  |
|  | L Brain-Stem | 2.69 | -8 | -18 | -20 |
| 40 |  |  |  |  |  |
|  | R Hippocampus | 3.78 | 36 | -34 | -6 |
| 40 |  |  |  |  |  |
|  | R Thalamus | 3.37 | 12 | -10 | 8 |
| 40 |  |  |  |  |  |
|  | L Caudate | 3.57 | -16 | 14 | 14 |
| 32 |  |  |  |  |  |
|  | R Accumbens | 3.30 | 8 | 6 | -8 |
| 32 |  |  |  |  |  |
|  | R Thalamus | 3.16 | 22 | -34 | 0 |
| 24 |  |  |  |  |  |
|  | R Hippocampus | 2.73 | 34 | -26 | -8 |
| 24 |  |  |  |  |  |
|  | R Frontal Pole | 2.80 | 32 | 58 | -6 |
| 24 |  |  |  |  |  |
|  | L Caudate | 2.74 | -18 | 18 | 6 |
| 24 |  |  |  |  |  |
|  | R Thalamus | 3.08 | 6 | -30 | 10 |
| 24 |  |  |  |  |  |
|  | R Middle Frontal Gyrus | 2.73 | 38 | 16 | 34 |
| 24 |  |  |  |  |  |
|  | R Superior Frontal Gyrus | 2.80 | 2 | 30 | 46 |
| 24 |  |  |  |  |  |
|  | L Angular Gyrus | 2.80 | -42 | -54 | 48 |
| 16 |  |  |  |  |  |
|  | R Brain-Stem | 2.60 | 8 | -38 | -28 |
| 16 |  |  |  |  |  |
|  | R Frontal Orbital Cortex | 2.99 | 38 | 20 | -16 |
| 16 |  |  |  |  |  |
|  | L Brain-Stem | 2.61 | -10 | -32 | -12 |
| 16 |  |  |  |  |  |
|  | L Frontal Pole | 2.39 | -32 | 52 | -14 |
| 16 |  |  |  |  |  |
|  | L Pallidum | 2.86 | -20 | -10 | -4 |
| 16 |  |  |  |  |  |
|  | R Thalamus | 2.64 | 14 | -34 | 8 |
| 16 |  |  |  |  |  |
|  | R Supramarginal Gyrus, posterior division | 2.87 | 58 | -42 | 14 |
| 16 |  |  |  |  |  |
|  | L Inferior Frontal Gyrus, pars triangularis | 2.92 | -50 | 22 | 14 |
| 16 |  |  |  |  |  |
|  | L Cingulate Gyrus, anterior division | 3.05 | -2 | 22 | 20 |
| 16 |  |  |  |  |  |
|  | L Cingulate Gyrus, posterior division | 2.69 | -4 | -20 | 30 |
| 16 |  |  |  |  |  |
|  | L Postcentral Gyrus | 2.39 | -42 | -30 | 46 |
| 16 |  |  |  |  |  |
|  | L Precentral Gyrus | 2.63 | -30 | -12 | 64 |
| 16 |  |  |  |  |  |
|  | L Precentral Gyrus | 2.60 | -34 | -6 | 66 |
| 8 |  |  |  |  |  |
|  | R Brain-Stem | 2.57 | 0 | -44 | -60 |
| 8 |  |  |  |  |  |
|  | R Brain-Stem | 2.58 | 0 | -32 | -60 |
| 8 |  |  |  |  |  |
|  | L Brain-Stem | 2.60 | -2 | -36 | -56 |
| 8 |  |  |  |  |  |
|  | R Brain-Stem | 2.58 | 4 | -38 | -40 |
| 8 |  |  |  |  |  |
|  | R Frontal Pole | 2.46 | 26 | 60 | -12 |
| 8 |  |  |  |  |  |
|  | R Lateral Occipital Cortex, inferior division | 2.47 | 38 | -88 | -10 |
| 8 |  |  |  |  |  |
|  | R Lateral Occipital Cortex, inferior division | 2.56 | 42 | -78 | -10 |
| 8 |  |  |  |  |  |
|  | L Lingual Gyrus | 2.58 | -12 | -84 | -8 |
| 8 |  |  |  |  |  |
|  | R Insular Cortex | 2.54 | 36 | 14 | -8 |
| 8 |  |  |  |  |  |
|  | R Frontal Pole | 2.60 | 28 | 58 | -8 |
| 8 |  |  |  |  |  |
|  | R Insular Cortex | 2.54 | 38 | 16 | -6 |
| 8 |  |  |  |  |  |
|  | L Thalamus | 2.84 | -20 | -36 | 0 |
| 8 |  |  |  |  |  |
|  | R Thalamus | 2.39 | 6 | -10 | 0 |
| 8 |  |  |  |  |  |
|  | L Caudate | 2.67 | -8 | 6 | 0 |
| 8 |  |  |  |  |  |
|  | L Caudate | 2.53 | -8 | 10 | 6 |
| 8 |  |  |  |  |  |
|  | L Inferior Frontal Gyrus, pars triangularis | 2.60 | -54 | 22 | 8 |
| 8 |  |  |  |  |  |
|  | L Occipital Pole | 2.58 | -20 | -102 | 10 |
| 8 |  |  |  |  |  |
|  | R Central Opercular Cortex | 2.64 | 46 | 2 | 12 |
| 8 |  |  |  |  |  |
|  | R Supramarginal Gyrus, posterior division | 2.60 | 66 | -38 | 14 |
| 8 |  |  |  |  |  |
|  | R Cingulate Gyrus, anterior division | 2.66 | 6 | 20 | 22 |
| 8 |  |  |  |  |  |
|  | R Frontal Pole | 2.48 | 6 | 60 | 28 |
| 8 |  |  |  |  |  |
|  | L Postcentral Gyrus | 2.33 | -42 | -34 | 44 |
| 8 |  |  |  |  |  |
|  | L Angular Gyrus | 2.65 | -42 | -56 | 52 |
| 8 |  |  |  |  |  |
|  | L Middle Frontal Gyrus | 2.54 | -40 | 0 | 52 |
| 8 |  |  |  |  |  |
|  | L Precentral Gyrus | 2.68 | -40 | -4 | 56 |

# Supplementary Table S4. [US > UT]. Descriptive statistics for clusters and local maxima from the conventional ‘boxcar’ analysis showing greater activation for Uncertain-Safety relative to Uncertain-Threat anticipation (FDR *q*<0.05, whole-brain corrected). 

| **mm**^**3**^ | **Label** | ***t*** | ***x*** | ***y*** | ***z*** |
| --- | --- | --- | --- | --- | --- |
| 378,128 |  |  |  |  |  |
|  | L Temporal Pole | 5.92 | -46 | 12 | -14 |
|  | R Temporal Pole | 5.59 | 48 | 12 | -12 |
|  | R Superior Temporal Gyrus, anterior division | 7.1 | 62 | 2 | -2 |
|  | R Amygdala | 7.91 | 22 | 0 | -16 |
|  | L Amygdala | 8.5 | -22 | 0 | -18 |
|  | R Temporal Fusiform Cortex, anterior division | 5.29 | 30 | 0 | -38 |
|  | R Amygdala | 6.12 | 28 | -2 | -12 |
|  | L Temporal Fusiform Cortex, anterior division | 4.3 | -30 | -2 | -36 |
|  | R Middle Temporal Gyrus, anterior division | 8.09 | 60 | -2 | -18 |
|  | L Middle Temporal Gyrus, anterior division | 7.22 | -62 | -4 | -16 |
|  | L Superior Temporal Gyrus, anterior division | 5.99 | -60 | -4 | -4 |
|  | R Planum Polare | 9.36 | 52 | -4 | -2 |
|  | R Amygdala | 8.13 | 16 | -6 | -16 |
|  | L Amygdala | 7.13 | -16 | -8 | -16 |
|  | L Insular Cortex | 15.08 | -40 | -8 | 6 |
|  | L Planum Polare | 9.43 | -52 | -8 | 2 |
|  | R Insular Cortex | 15.55 | 38 | -8 | 12 |
|  | R Planum Temporale | 12.17 | 64 | -8 | 4 |
|  | L Precentral Gyrus | 15.16 | -46 | -12 | 32 |
|  | L Putamen | 6.25 | -30 | -12 | -6 |
|  | R Heschls Gyrus (includes H1 and H2) | 11.06 | 54 | -12 | 6 |
|  | R Hippocampus | 10.69 | 18 | -12 | -22 |
|  | R Putamen | 4.54 | 28 | -12 | -6 |
|  | L Amygdala | 5.86 | -20 | -14 | -14 |
|  | L Central Opercular Cortex | 17.56 | -38 | -14 | 16 |
|  | L Juxtapositional Lobule Cortex (formerly Supplementary Motor Cortex) | 8.1 | -8 | -14 | 48 |
|  | L Middle Temporal Gyrus, posterior division | 4.48 | -62 | -14 | -22 |
|  | R Middle Temporal Gyrus, posterior division | 5.04 | 64 | -16 | -22 |
|  | L Planum Temporale | 12.09 | -62 | -20 | 10 |
|  | L Parahippocampal Gyrus, anterior division | 3.97 | -32 | -20 | -28 |
|  | R Temporal Fusiform Cortex, posterior division | 7.15 | 34 | -22 | -24 |
|  | R Supramarginal Gyrus, anterior division | 4.52 | 62 | -22 | 46 |
|  | L Heschls Gyrus (includes H1 and H2) | 9.02 | -46 | -24 | 12 |
|  | R Precentral Gyrus | 15.08 | 4 | -26 | 62 |
|  | R Inferior Temporal Gyrus, posterior division | 4.06 | 44 | -26 | -20 |
|  | L Parietal Operculum Cortex | 15.22 | -34 | -28 | 18 |
|  | R Thalamus | 7.84 | 16 | -28 | 2 |
|  | R Superior Temporal Gyrus, posterior division | 6.09 | 66 | -28 | 8 |
|  | L Thalamus | 8.9 | -14 | -30 | -4 |
|  | L Supramarginal Gyrus, anterior division | 3.86 | -56 | -30 | 42 |
|  | R Parahippocampal Gyrus, posterior division | 12.24 | 28 | -32 | -18 |
|  | R Postcentral Gyrus | 17.12 | 2 | -34 | 62 |
|  | L Postcentral Gyrus | 17.03 | -2 | -36 | 62 |
|  | L Temporal Fusiform Cortex, posterior division | 11.21 | -28 | -38 | -16 |
|  | L Hippocampus | 12.72 | -10 | -42 | 4 |
|  | L Inferior Temporal Gyrus, posterior division | 3.56 | -64 | -42 | -20 |
|  | R Cingulate Gyrus, posterior division | 13.82 | 10 | -42 | 4 |
|  | R Middle Temporal Gyrus, temporooccipital part | 5.89 | 64 | -42 | -12 |
|  | L Inferior Temporal Gyrus, temporooccipital part | 5.93 | -46 | -50 | -18 |
|  | L Middle Temporal Gyrus, temporooccipital part | 6.57 | -58 | -52 | -10 |
|  | L Superior Parietal Lobule | 4.66 | -34 | -52 | 56 |
|  | L Cingulate Gyrus, posterior division | 13.01 | -6 | -54 | 24 |
|  | L Precuneus Cortex | 14.64 | -2 | -56 | 14 |
|  | R Superior Parietal Lobule | 4.72 | 28 | -56 | 62 |
|  | R Temporal Occipital Fusiform Cortex | 3.3 | 38 | -56 | -16 |
|  | L Lingual Gyrus | 13.56 | -12 | -58 | 4 |
|  | L Temporal Occipital Fusiform Cortex | 9.46 | -28 | -58 | -8 |
|  | L Angular Gyrus | 4.56 | -42 | -58 | 26 |
|  | R Angular Gyrus | 7.24 | 48 | -58 | 22 |
|  | R Inferior Temporal Gyrus, temporooccipital part | 5.05 | 60 | -60 | -12 |
|  | R Occipital Fusiform Gyrus | 7.61 | 26 | -66 | -10 |
|  | R Precuneus Cortex | 19.91 | 2 | -68 | 22 |
|  | R Lateral Occipital Cortex, superior division | 12.64 | 52 | -68 | 34 |
|  | R Intracalcarine Cortex | 20.12 | 12 | -70 | 12 |
|  | R Lateral Occipital Cortex, inferior division | 6.75 | 54 | -72 | 4 |
|  | L Occipital Fusiform Gyrus | 5.68 | -26 | -74 | -10 |
|  | R Lingual Gyrus | 18.65 | 0 | -74 | 8 |
|  | L Lateral Occipital Cortex, superior division | 10.88 | -46 | -76 | 30 |
|  | L Lateral Occipital Cortex, inferior division | 7.64 | -50 | -78 | 4 |
|  | L Intracalcarine Cortex | 21 | -14 | -80 | 6 |
|  | R Occipital Pole | 7.27 | 8 | -90 | 32 |
|  | L Occipital Pole | 8.91 | -2 | -96 | 18 |
| 33,528 |  |  |  |  |  |
|  | L Frontal Pole | 7.6 | -6 | 72 | 4 |
|  | R Frontal Pole | 12.12 | 2 | 64 | -4 |
|  | L Frontal Medial Cortex | 7.7 | -4 | 52 | -6 |
|  | L Cingulate Gyrus, anterior division | 3.65 | -6 | 44 | 4 |
|  | R Frontal Medial Cortex | 8.1 | 6 | 40 | -16 |
|  | R Paracingulate Gyrus | 6.68 | 6 | 34 | -10 |
|  | R Cingulate Gyrus, anterior division | 6.28 | 4 | 34 | 8 |
|  | L Paracingulate Gyrus | 5.52 | -6 | 32 | -14 |
|  | R Subcallosal Cortex | 7.06 | 0 | 30 | -24 |
|  | L Subcallosal Cortex | 4.91 | -6 | 24 | -16 |
| 31,912 |  |  |  |  |  |
|  | L Frontal Orbital Cortex | 9.76 | -34 | 36 | -12 |
|  | L Superior Frontal Gyrus | 9.68 | -24 | 24 | 50 |
|  | L Middle Frontal Gyrus | 8.8 | -26 | 16 | 50 |
|  | L Inferior Frontal Gyrus, pars triangularis | 8.18 | -44 | 30 | 20 |
|  | L Frontal Pole | 7.36 | -46 | 36 | 14 |
| 11,928 |  |  |  |  |  |
|  | R Frontal Pole | 7.27 | 52 | 40 | 12 |
|  | R Inferior Frontal Gyrus, pars triangularis | 6.42 | 46 | 34 | 16 |
|  | R Superior Frontal Gyrus | 12.09 | 26 | 30 | 52 |
|  | R Middle Frontal Gyrus | 9.86 | 28 | 20 | 50 |
| 3,896 |  |  |  |  |  |
|  | R Frontal Pole | 10.18 | 36 | 38 | -14 |
|  | R Frontal Orbital Cortex | 6.98 | 24 | 32 | -14 |
| 2,864 |  |  |  |  |  |
|  | R Caudate | 5.7 | 14 | 24 | -2 |
|  | L Caudate | 5.58 | -12 | 22 | 2 |
|  | R Accumbens | 6.62 | 10 | 20 | -4 |
|  | R Subcallosal Cortex | 6.31 | 2 | 10 | -4 |
| 1,688 |  |  |  |  |  |
|  | R Brain-Stem | 3.81 | 2 | -18 | -44 |
|  | L Brain-Stem | 4.38 | -2 | -26 | -44 |
| 616 |  |  |  |  |  |
|  | R Brain-Stem | 4.47 | 10 | -22 | -42 |
| 616 |  |  |  |  |  |
|  | L Cingulate Gyrus, anterior division | 5.59 | -4 | 0 | 28 |
|  | R Cingulate Gyrus, anterior division | 6.05 | 4 | -4 | 30 |
| 600 |  |  |  |  |  |
|  | R Occipital Pole | 3.75 | 18 | -100 | -8 |
| 312 |  |  |  |  |  |
|  | L Occipital Pole | 2.84 | -24 | -100 | -6 |
| 112 |  |  |  |  |  |
|  | L Temporal Pole | 3.52 | -20 | 12 | -36 |
| 104 |  |  |  |  |  |
|  | R Frontal Pole | 2.86 | 38 | 52 | -4 |
| 96 |  |  |  |  |  |
|  | L Paracingulate Gyrus | 2.72 | -10 | 44 | 18 |
| 72 |  |  |  |  |  |
|  | L Frontal Pole | 3.06 | -10 | 44 | -28 |
| 72 |  |  |  |  |  |
|  | L Frontal Pole | 3.12 | -14 | 52 | -22 |
| 64 |  |  |  |  |  |
|  | R Frontal Pole | 3.88 | 46 | 50 | -16 |
| 64 |  |  |  |  |  |
|  | R Postcentral Gyrus | 3.13 | 38 | -30 | 62 |
| 56 |  |  |  |  |  |
|  | L Superior Parietal Lobule | 2.3 | -40 | -46 | 48 |
| 40 |  |  |  |  |  |
|  | L Temporal Pole | 2.56 | -28 | 4 | -40 |
| 40 |  |  |  |  |  |
|  | R Inferior Temporal Gyrus, posterior division | 3.11 | 52 | -32 | -22 |
| 32 |  |  |  |  |  |
|  | R Temporal Pole | 2.77 | 24 | 10 | -36 |
| 32 |  |  |  |  |  |
|  | L Insular Cortex | 3.13 | -32 | 6 | 8 |
| 24 |  |  |  |  |  |
|  | R Frontal Pole | 2.48 | 28 | 62 | 2 |
| 24 |  |  |  |  |  |
|  | L Superior Parietal Lobule | 2.15 | -36 | -44 | 42 |
| 16 |  |  |  |  |  |
|  | L Temporal Pole | 2.41 | -34 | 6 | -36 |
| 16 |  |  |  |  |  |
|  | R Frontal Pole | 2.29 | 24 | 56 | 10 |
| 16 |  |  |  |  |  |
|  | R Precentral Gyrus | 2.23 | 60 | 10 | 34 |
| 16 |  |  |  |  |  |
|  | R Cingulate Gyrus, anterior division | 2.35 | 8 | -8 | 40 |
| 8 |  |  |  |  |  |
|  | R Parahippocampal Gyrus, anterior division | 2.19 | 32 | -16 | -32 |
| 8 |  |  |  |  |  |
|  | L Inferior Temporal Gyrus, posterior division | 2.12 | -54 | -38 | -28 |
| 8 |  |  |  |  |  |
|  | L Frontal Orbital Cortex | 2.35 | -18 | 28 | -22 |
| 8 |  |  |  |  |  |
|  | R Frontal Pole | 2.61 | 18 | 54 | -22 |
| 8 |  |  |  |  |  |
|  | L Frontal Medial Cortex | 2.13 | -8 | 46 | -20 |
| 8 |  |  |  |  |  |
|  | L Frontal Pole | 2.43 | -20 | 44 | -16 |
| 8 |  |  |  |  |  |
|  | R Frontal Pole | 2.15 | 28 | 62 | -16 |
| 8 |  |  |  |  |  |
|  | R Frontal Pole | 2.2 | 46 | 52 | -6 |
| 8 |  |  |  |  |  |
|  | L Brain-Stem | 2.19 | -6 | -36 | -4 |
| 8 |  |  |  |  |  |
|  | L Frontal Pole | 2.27 | -38 | 44 | -2 |
| 8 |  |  |  |  |  |
|  | L Caudate | 2.38 | -6 | 16 | 2 |
| 8 |  |  |  |  |  |
|  | L Putamen | 2.27 | -28 | -12 | 12 |
| 8 |  |  |  |  |  |
|  | R Cingulate Gyrus, anterior division | 2.16 | 4 | -12 | 34 |
| 8 |  |  |  |  |  |
|  | R Supramarginal Gyrus, posterior division | 2.12 | 44 | -38 | 38 |
| 8 |  |  |  |  |  |
|  | L Cingulate Gyrus, posterior division | 2.54 | -10 | -32 | 38 |
| 8 |  |  |  |  |  |
|  | R Middle Frontal Gyrus | 2.13 | 40 | 18 | 38 |
| 8 |  |  |  |  |  |
|  | R Superior Parietal Lobule | 2.14 | 28 | -56 | 44 |
| 8 |  |  |  |  |  |
|  | R Superior Frontal Gyrus | 2.31 | 28 | 2 | 58 |

# Supplementary Table S5. [CS > CT]. Descriptive statistics for clusters and local maxima from the conventional ‘boxcar’ analysis showing greater activation for Certain-Safety relative to Certain-Threat anticipation (FDR *q*<0.05, whole-brain corrected). 

| ***mm***^***3***^ | **Label** | ***t*** | ***x*** | ***y*** | ***z*** |
| --- | --- | --- | --- | --- | --- |
| 244,512 |  |  |  |  |  |
|  | L Frontal Orbital Cortex | 5.13 | -28 | 12 | -24 |
|  | L Temporal Pole | 4.82 | -32 | 10 | -26 |
|  | L Amygdala | 3.89 | -24 | 0 | -14 |
|  | L Superior Temporal Gyrus, anterior division | 4.94 | -60 | 0 | -10 |
|  | L Amygdala | 3.87 | -26 | -2 | -12 |
|  | L Pallidum | 2.89 | -20 | -4 | 0 |
|  | L Middle Temporal Gyrus, anterior division | 6.23 | -60 | -6 | -12 |
|  | R Temporal Fusiform Cortex, anterior division | 2.91 | 38 | -8 | -38 |
|  | L Planum Polare | 6.93 | -48 | -10 | 0 |
|  | L Juxtapositional Lobule Cortex (formerly Supplementary Motor Cortex) | 5.85 | -10 | -10 | 42 |
|  | R Cingulate Gyrus, anterior division | 5.15 | 10 | -10 | 42 |
|  | R Juxtapositional Lobule Cortex (formerly Supplementary Motor Cortex) | 5.08 | 0 | -10 | 48 |
|  | L Left Putamen | 5.91 | -32 | -12 | 2 |
|  | L Postcentral Gyrus | 8.18 | -54 | -16 | 50 |
|  | L Central Opercular Cortex | 6.6 | -52 | -16 | 10 |
|  | L Parahippocampal Gyrus, anterior division | 4.98 | -20 | -16 | -24 |
|  | L Middle Temporal Gyrus, posterior division | 4.19 | -52 | -16 | -10 |
|  | R Inferior Temporal Gyrus, posterior division | 3.44 | 44 | -16 | -32 |
|  | L Planum Temporale | 7.29 | -62 | -18 | 8 |
|  | R Parahippocampal Gyrus, anterior division | 6.58 | 20 | -20 | -22 |
|  | L Heschls Gyrus (includes H1 and H2) | 8.09 | -50 | -22 | 8 |
|  | L Hippocampus | 4.73 | -24 | -24 | -10 |
|  | L Precentral Gyrus | 9.2 | -26 | -26 | 66 |
|  | R Precentral Gyrus | 8.59 | 6 | -26 | 56 |
|  | R Hippocampus | 7.93 | 24 | -26 | -8 |
|  | R Temporal Fusiform Cortex, posterior division | 3.61 | 38 | -26 | -30 |
|  | L Insular Cortex | 8.85 | -32 | -28 | 16 |
|  | L Parietal Operculum Cortex | 3.76 | -42 | -30 | 18 |
|  | R Thalamus | 5.84 | 18 | -30 | -4 |
|  | L Thalamus | 4.76 | -12 | -34 | 0 |
|  | R Parahippocampal Gyrus, posterior division | 10.16 | 22 | -34 | -18 |
|  | L Parahippocampal Gyrus, posterior division | 9.42 | -30 | -40 | -12 |
|  | L Temporal Fusiform Cortex, posterior division | 3.22 | -34 | -42 | -20 |
|  | R Postcentral Gyrus | 7.07 | 10 | -42 | 74 |
|  | L Cingulate Gyrus, posterior division | 9.65 | -10 | -44 | 2 |
|  | L Superior Parietal Lobule | 4.9 | -22 | -44 | 66 |
|  | R Lingual Gyrus | 12 | 14 | -44 | -2 |
|  | L Lingual Gyrus | 12.19 | -20 | -46 | -10 |
|  | R Cingulate Gyrus, posterior division | 9.68 | 10 | -46 | 4 |
|  | R Angular Gyrus | 5.03 | 48 | -54 | 20 |
|  | R Superior Parietal Lobule | 3.47 | 14 | -54 | 68 |
|  | L Angular Gyrus | 3.39 | -60 | -58 | 14 |
|  | R Temporal Occipital Fusiform Cortex | 5.37 | 32 | -58 | -14 |
|  | R Middle Temporal Gyrus, temporooccipital part | 2.75 | 52 | -58 | 10 |
|  | L Precuneus Cortex | 9.3 | -8 | -60 | 14 |
|  | L Inferior Temporal Gyrus, temporooccipital part | 3.77 | -54 | -60 | -12 |
|  | L Temporal Occipital Fusiform Cortex | 3.61 | -38 | -60 | -18 |
|  | L Middle Temporal Gyrus, temporooccipital part | 3.52 | -50 | -60 | 0 |
|  | R Precuneus Cortex | 15.18 | 0 | -68 | 18 |
|  | R Occipital Fusiform Gyrus | 9.69 | 28 | -68 | -6 |
|  | L Occipital Fusiform Gyrus | 8.43 | -28 | -74 | -8 |
|  | R Intracalcarine Cortex | 20.88 | 12 | -74 | 12 |
|  | R Lateral Occipital Cortex, inferior division | 6.65 | 42 | -74 | 10 |
|  | L Intracalcarine Cortex | 21.08 | -10 | -76 | 10 |
|  | L Lateral Occipital Cortex, inferior division | 4.86 | -52 | -76 | 4 |
|  | R Lateral Occipital Cortex, superior division | 7.02 | 50 | -76 | 16 |
|  | L Lateral Occipital Cortex, superior division | 6.96 | -46 | -80 | 18 |
|  | R Cuneal Cortex | 8.42 | 0 | -86 | 34 |
|  | L Occipital Pole | 10.01 | -2 | -96 | 10 |
|  | R Occipital Pole | 8.8 | 8 | -96 | 18 |
| 56,792 |  |  |  |  |  |
|  | R Frontal Pole | 7.85 | 0 | 64 | -10 |
|  | L Frontal Pole | 6.29 | -6 | 58 | -12 |
|  | R Frontal Medial Cortex | 7.04 | 0 | 50 | -12 |
|  | L Paracingulate Gyrus | 5.03 | -6 | 46 | -6 |
|  | R Paracingulate Gyrus | 6.58 | 0 | 42 | -10 |
|  | L Cingulate Gyrus, anterior division | 4.99 | -2 | 40 | -2 |
|  | L Frontal Medial Cortex | 6.29 | -8 | 36 | -12 |
|  | R Cingulate Gyrus, anterior division | 4.97 | 2 | 34 | 14 |
|  | R Frontal Orbital Cortex | 4.54 | 22 | 28 | -20 |
|  | R Right Accumbens | 5.15 | 8 | 18 | -6 |
|  | R Temporal Pole | 4.59 | 36 | 18 | -28 |
|  | L Subcallosal Cortex | 5.65 | -2 | 16 | -6 |
|  | R Subcallosal Cortex | 5.13 | 4 | 14 | -10 |
|  | R Superior Temporal Gyrus, anterior division | 4.59 | 60 | -2 | -12 |
|  | R Middle Temporal Gyrus, anterior division | 4.48 | 56 | -2 | -20 |
|  | R Middle Temporal Gyrus, posterior division | 4.24 | 54 | -10 | -16 |
|  | R Pallidum | 3.68 | 24 | -10 | 6 |
|  | R Postcentral Gyrus | 6.62 | 50 | -12 | 50 |
|  | R Central Opercular Cortex | 7.15 | 38 | -14 | 18 |
|  | R Precentral Gyrus | 6.94 | 36 | -14 | 44 |
|  | R Putamen | 4.35 | 30 | -14 | 2 |
|  | R Planum Polare | 6.5 | 40 | -20 | 0 |
|  | R Insular Cortex | 9.1 | 36 | -22 | 14 |
|  | R Heschls Gyrus (includes H1 and H2) | 6.98 | 38 | -22 | 6 |
|  | R Planum Temporale | 6.2 | 62 | -22 | 12 |
|  | R Superior Temporal Gyrus, posterior division | 3.63 | 70 | -24 | 4 |
| 6,584 |  |  |  |  |  |
|  | R Frontal Pole | 2.79 | 20 | 44 | 46 |
|  | R Superior Frontal Gyrus | 6.03 | 20 | 30 | 42 |
|  | R Middle Frontal Gyrus | 7.68 | 26 | 16 | 46 |
| 6,400 |  |  |  |  |  |
|  | L Middle Frontal Gyrus | 4.59 | -28 | 26 | 46 |
|  | L Superior Frontal Gyrus | 6.29 | -22 | 24 | 40 |
| 1,272 |  |  |  |  |  |
|  | L Inferior Frontal Gyrus, pars triangularis | 4.19 | -48 | 30 | 16 |
|  | L Middle Frontal Gyrus | 3.62 | -46 | 22 | 26 |
|  | L Inferior Frontal Gyrus, pars opercularis | 3.16 | -42 | 14 | 26 |
| 976 |  |  |  |  |  |
|  | R Frontal Pole | 3.01 | 52 | 36 | 12 |
|  | R Inferior Frontal Gyrus, pars triangularis | 2.98 | 46 | 32 | 14 |
|  | R Middle Frontal Gyrus | 3.52 | 52 | 28 | 28 |
| 912 |  |  |  |  |  |
|  | L Frontal Orbital Cortex | 5.05 | -32 | 34 | -12 |
| 704 |  |  |  |  |  |
|  | L Brain-Stem | 3.78 | -14 | -26 | -34 |
| 448 |  |  |  |  |  |
|  | R Frontal Pole | 4.52 | 36 | 34 | -16 |
|  | R Frontal Orbital Cortex | 3.17 | 40 | 28 | -14 |
| 320 |  |  |  |  |  |
|  | R Amygdala | 3.87 | 22 | 2 | -16 |
|  | R Amygdala | 2.72 | 18 | 0 | -24 |
|  | R Amygdala | 4.85 | 26 | 0 | -12 |
| 248 |  |  |  |  |  |
|  | L Middle Frontal Gyrus | 3.49 | -54 | 16 | 34 |
| 232 |  |  |  |  |  |
|  | R Temporal Pole | 3.27 | 30 | 6 | -38 |
|  | R Parahippocampal Gyrus, anterior division | 3.35 | 26 | 2 | -34 |
| 168 |  |  |  |  |  |
|  | L Angular Gyrus | 2.81 | -56 | -52 | 16 |
| 160 |  |  |  |  |  |
|  | R Temporal Fusiform Cortex, anterior division | 3.35 | 26 | 0 | -44 |
| 144 |  |  |  |  |  |
|  | L Frontal Pole | 2.77 | -12 | 46 | 44 |
| 120 |  |  |  |  |  |
|  | R Thalamus | 3.55 | 6 | -14 | 12 |
| 104 |  |  |  |  |  |
|  | R Brain-Stem | 4.12 | 10 | -14 | -34 |
| 104 |  |  |  |  |  |
|  | R Lateral Occipital Cortex, superior division | 3.18 | 34 | -72 | 50 |
| 96 |  |  |  |  |  |
|  | R Brain-Stem | 3.68 | 0 | -18 | -30 |
|  | L Brain-Stem | 2.83 | -2 | -20 | -32 |
| 88 |  |  |  |  |  |
|  | L Left Thalamus | 3.17 | -18 | -24 | 10 |
| 88 |  |  |  |  |  |
|  | R Insular Cortex | 2.56 | 34 | 8 | 8 |
| 80 |  |  |  |  |  |
|  | R Inferior Temporal Gyrus, anterior division | 3.16 | 42 | 2 | -42 |
| 64 |  |  |  |  |  |
|  | L Temporal Fusiform Cortex, anterior division | 2.93 | -34 | -6 | -46 |
| 64 |  |  |  |  |  |
|  | R Temporal Fusiform Cortex, anterior division | 3.33 | 34 | -2 | -42 |
| 64 |  |  |  |  |  |
|  | R Inferior Temporal Gyrus, posterior division | 2.96 | 52 | -18 | -34 |
| 64 |  |  |  |  |  |
|  | R Frontal Pole | 3.83 | 12 | 36 | -20 |
| 64 |  |  |  |  |  |
|  | R Middle Temporal Gyrus, posterior division | 2.93 | 70 | -24 | -14 |
| 64 |  |  |  |  |  |
|  | R Thalamus | 3.1 | 14 | -24 | 18 |
| 56 |  |  |  |  |  |
|  | R Brain-Stem | 3.35 | 8 | -44 | -36 |
| 56 |  |  |  |  |  |
|  | R Frontal Orbital Cortex | 2.95 | 14 | 30 | -18 |
|  | R Subcallosal Cortex | 2.81 | 10 | 28 | -20 |
| 56 |  |  |  |  |  |
|  | R Middle Temporal Gyrus, temporooccipital part | 2.76 | 54 | -56 | 2 |
| 56 |  |  |  |  |  |
|  | R Thalamus | 2.84 | 20 | -26 | 4 |
| 56 |  |  |  |  |  |
|  | R Cingulate Gyrus, anterior division | 3.84 | 4 | 10 | 36 |
| 56 |  |  |  |  |  |
|  | R Cingulate Gyrus, posterior division | 2.41 | 14 | -32 | 38 |
| 48 |  |  |  |  |  |
|  | R Frontal Pole | 2.87 | 14 | 56 | -20 |
| 48 |  |  |  |  |  |
|  | L Left Thalamus | 2.86 | -14 | -24 | 18 |
| 48 |  |  |  |  |  |
|  | R Middle Frontal Gyrus | 2.82 | 42 | 18 | 52 |
| 40 |  |  |  |  |  |
|  | R Frontal Pole | 2.98 | 26 | 66 | 0 |
| 40 |  |  |  |  |  |
|  | L Superior Temporal Gyrus, posterior division | 2.81 | -56 | -36 | 4 |
| 40 |  |  |  |  |  |
|  | L Superior Parietal Lobule | 2.71 | -22 | -56 | 60 |
| 32 |  |  |  |  |  |
|  | L Brain-Stem | 2.91 | -4 | -42 | -36 |
| 32 |  |  |  |  |  |
|  | R Frontal Pole | 2.74 | 14 | 36 | -26 |
| 32 |  |  |  |  |  |
|  | L Frontal Pole | 2.52 | -12 | 36 | -26 |
| 32 |  |  |  |  |  |
|  | R Amygdala | 3.31 | 18 | -6 | -16 |
| 32 |  |  |  |  |  |
|  | L Temporal Pole | 3.04 | -48 | 10 | -10 |
| 32 |  |  |  |  |  |
|  | L Frontal Pole | 2.51 | -18 | 70 | -8 |
| 32 |  |  |  |  |  |
|  | L Frontal Pole | 3.53 | -14 | 62 | 16 |
| 24 |  |  |  |  |  |
|  | R Brain-Stem | 2.53 | 4 | -30 | -46 |
| 24 |  |  |  |  |  |
|  | R Inferior Temporal Gyrus, posterior division | 2.42 | 60 | -16 | -38 |
| 24 |  |  |  |  |  |
|  | L Temporal Fusiform Cortex, anterior division | 2.6 | -36 | -10 | -38 |
| 24 |  |  |  |  |  |
|  | L Temporal Pole | 2.78 | -34 | 10 | -36 |
| 24 |  |  |  |  |  |
|  | L Inferior Temporal Gyrus, posterior division | 2.51 | -58 | -40 | -28 |
| 24 |  |  |  |  |  |
|  | R Temporal Pole | 3.1 | 44 | 20 | -26 |
| 24 |  |  |  |  |  |
|  | L Frontal Orbital Cortex | 2.84 | -22 | 16 | -20 |
| 24 |  |  |  |  |  |
|  | L Frontal Pole | 2.57 | -14 | 50 | -20 |
| 24 |  |  |  |  |  |
|  | L Inferior Temporal Gyrus, temporooccipital part | 2.59 | -58 | -48 | -16 |
| 24 |  |  |  |  |  |
|  | L Frontal Orbital Cortex | 2.41 | -12 | 14 | -18 |
| 24 |  |  |  |  |  |
|  | R Planum Polare | 2.85 | 44 | -6 | -12 |
| 24 |  |  |  |  |  |
|  | R Lateral Occipital Cortex, inferior division | 2.55 | 52 | -76 | -6 |
| 24 |  |  |  |  |  |
|  | L Frontal Pole | 2.78 | -6 | 50 | 48 |
| 16 |  |  |  |  |  |
|  | R Temporal Fusiform Cortex, anterior division | 2.73 | 30 | -6 | -46 |
| 16 |  |  |  |  |  |
|  | L Temporal Fusiform Cortex, anterior division | 2.37 | -26 | -8 | -42 |
| 16 |  |  |  |  |  |
|  | L Temporal Pole | 2.44 | -26 | 2 | -42 |
| 16 |  |  |  |  |  |
|  | R Brain-Stem | 2.94 | 10 | -24 | -40 |
| 16 |  |  |  |  |  |
|  | L Brain-Stem | 2.86 | -2 | -24 | -38 |
| 16 |  |  |  |  |  |
|  | R Temporal Pole | 2.52 | 24 | 12 | -36 |
| 16 |  |  |  |  |  |
|  | L Inferior Temporal Gyrus, posterior division | 2.82 | -50 | -20 | -30 |
| 16 |  |  |  |  |  |
|  | R Temporal Pole | 2.69 | 52 | 20 | -26 |
| 16 |  |  |  |  |  |
|  | L Brain-Stem | 2.54 | -14 | -32 | -26 |
| 16 |  |  |  |  |  |
|  | R Brain-Stem | 2.79 | 10 | -28 | -24 |
| 16 |  |  |  |  |  |
|  | R Frontal Pole | 2.39 | 14 | 48 | -26 |
| 16 |  |  |  |  |  |
|  | R Frontal Pole | 2.44 | 14 | 42 | -24 |
| 16 |  |  |  |  |  |
|  | L Lateral Occipital Cortex, inferior division | 2.59 | -38 | -84 | -20 |
| 16 |  |  |  |  |  |
|  | L Frontal Orbital Cortex | 2.95 | -42 | 22 | -16 |
| 16 |  |  |  |  |  |
|  | R Frontal Pole | 2.49 | 24 | 54 | -16 |
| 16 |  |  |  |  |  |
|  | L Inferior Temporal Gyrus, temporooccipital part | 2.56 | -52 | -50 | -12 |
| 16 |  |  |  |  |  |
|  | L Insular Cortex | 2.55 | -38 | -4 | -12 |
| 16 |  |  |  |  |  |
|  | R Frontal Pole | 2.72 | 40 | 44 | -12 |
| 16 |  |  |  |  |  |
|  | R Pallidum | 2.64 | 24 | -4 | -6 |
| 16 |  |  |  |  |  |
|  | R Middle Temporal Gyrus, temporooccipital part | 2.62 | 66 | -54 | -2 |
| 16 |  |  |  |  |  |
|  | L Caudate | 2.74 | -14 | 24 | -2 |
| 16 |  |  |  |  |  |
|  | R Pallidum | 2.79 | 20 | -6 | 4 |
| 16 |  |  |  |  |  |
|  | R Thalamus | 2.58 | 16 | -22 | 12 |
| 16 |  |  |  |  |  |
|  | R Caudate | 2.45 | 14 | 18 | 14 |
| 16 |  |  |  |  |  |
|  | R Cingulate Gyrus, anterior division | 2.41 | 4 | -8 | 30 |
| 16 |  |  |  |  |  |
|  | R Cingulate Gyrus, anterior division | 2.79 | 0 | -2 | 30 |
| 16 |  |  |  |  |  |
|  | L Frontal Pole | 3.03 | -14 | 52 | 30 |
| 16 |  |  |  |  |  |
|  | R Superior Frontal Gyrus | 2.53 | 0 | 50 | 36 |
| 16 |  |  |  |  |  |
|  | R Middle Frontal Gyrus | 2.71 | 42 | 18 | 40 |
| 8 |  |  |  |  |  |
|  | L Temporal Pole | 2.28 | -34 | 8 | -48 |
| 8 |  |  |  |  |  |
|  | R Inferior Temporal Gyrus, posterior division | 2.41 | 44 | -14 | -40 |
| 8 |  |  |  |  |  |
|  | L Parahippocampal Gyrus, anterior division | 2.71 | -22 | -2 | -38 |
| 8 |  |  |  |  |  |
|  | R Inferior Temporal Gyrus, anterior division | 2.46 | 38 | 2 | -38 |
| 8 |  |  |  |  |  |
|  | L Temporal Pole | 2.42 | -32 | 12 | -38 |
| 8 |  |  |  |  |  |
|  | R Brain-Stem | 2.44 | 12 | -24 | -36 |
| 8 |  |  |  |  |  |
|  | L Parahippocampal Gyrus, anterior division | 2.42 | -30 | -16 | -34 |
| 8 |  |  |  |  |  |
|  | R Brain-Stem | 2.55 | 12 | -30 | -32 |
| 8 |  |  |  |  |  |
|  | L Brain-Stem | 2.29 | -2 | -26 | -28 |
| 8 |  |  |  |  |  |
|  | L Temporal Fusiform Cortex, posterior division | 2.5 | -40 | -22 | -28 |
| 8 |  |  |  |  |  |
|  | L Temporal Fusiform Cortex, posterior division | 2.75 | -42 | -40 | -24 |
| 8 |  |  |  |  |  |
|  | L Temporal Fusiform Cortex, posterior division | 2.38 | -46 | -40 | -24 |
| 8 |  |  |  |  |  |
|  | R Inferior Temporal Gyrus, posterior division | 2.38 | 60 | -30 | -24 |
| 8 |  |  |  |  |  |
|  | L Frontal Orbital Cortex | 2.3 | -28 | 26 | -22 |
| 8 |  |  |  |  |  |
|  | R Temporal Fusiform Cortex, posterior division | 2.51 | 42 | -24 | -20 |
| 8 |  |  |  |  |  |
|  | L Frontal Orbital Cortex | 2.43 | -24 | 24 | -20 |
| 8 |  |  |  |  |  |
|  | R Frontal Pole | 2.63 | 16 | 40 | -20 |
| 8 |  |  |  |  |  |
|  | L Inferior Temporal Gyrus, temporooccipital part | 2.39 | -48 | -46 | -18 |
| 8 |  |  |  |  |  |
|  | R Temporal Occipital Fusiform Cortex | 2.68 | 42 | -44 | -16 |
| 8 |  |  |  |  |  |
|  | R Inferior Temporal Gyrus, temporooccipital part | 2.28 | 46 | -42 | -16 |
| 8 |  |  |  |  |  |
|  | L Amygdala | 2.37 | -14 | -8 | -16 |
| 8 |  |  |  |  |  |
|  | L Frontal Orbital Cortex | 2.6 | -14 | 20 | -16 |
| 8 |  |  |  |  |  |
|  | L Brain-Stem | 2.29 | -8 | -34 | -8 |
| 8 |  |  |  |  |  |
|  | L Inferior Temporal Gyrus, temporooccipital part | 2.34 | -42 | -58 | -6 |
| 8 |  |  |  |  |  |
|  | L Superior Temporal Gyrus, posterior division | 2.42 | -60 | -22 | -2 |
| 8 |  |  |  |  |  |
|  | R Middle Temporal Gyrus, posterior division | 2.37 | 70 | -38 | 0 |
| 8 |  |  |  |  |  |
|  | R Paracingulate Gyrus | 2.3 | 12 | 52 | 2 |
| 8 |  |  |  |  |  |
|  | R Thalamus | 2.39 | 8 | -14 | 4 |
| 8 |  |  |  |  |  |
|  | R Thalamus | 2.36 | 6 | -20 | 8 |
| 8 |  |  |  |  |  |
|  | L Caudate | 2.53 | -12 | 20 | 8 |
| 8 |  |  |  |  |  |
|  | R Middle Temporal Gyrus, temporooccipital part | 2.34 | 68 | -46 | 10 |
| 8 |  |  |  |  |  |
|  | L Cingulate Gyrus, anterior division | 2.93 | -4 | 26 | 16 |
| 8 |  |  |  |  |  |
|  | R Angular Gyrus | 2.28 | 58 | -48 | 18 |
| 8 |  |  |  |  |  |
|  | L Thalamus | 2.3 | -6 | -14 | 18 |
| 8 |  |  |  |  |  |
|  | L Cingulate Gyrus, anterior division | 2.66 | -4 | 0 | 28 |
| 8 |  |  |  |  |  |
|  | R Cingulate Gyrus, anterior division | 2.56 | 2 | 2 | 28 |
| 8 |  |  |  |  |  |
|  | L Supramarginal Gyrus, anterior division | 2.27 | -60 | -28 | 32 |
| 8 |  |  |  |  |  |
|  | R Cingulate Gyrus, anterior division | 2.4 | 4 | -14 | 34 |
| 8 |  |  |  |  |  |
|  | L Lateral Occipital Cortex, superior division | 2.33 | -34 | -68 | 38 |
| 8 |  |  |  |  |  |
|  | R Supramarginal Gyrus, anterior division | 2.33 | 58 | -24 | 40 |
| 8 |  |  |  |  |  |
|  | L Superior Frontal Gyrus | 2.51 | -2 | 42 | 42 |
| 8 |  |  |  |  |  |
|  | L Superior Frontal Gyrus | 2.45 | -2 | 48 | 46 |
| 8 |  |  |  |  |  |
|  | R Frontal Pole | 2.38 | 12 | 50 | 48 |
| 8 |  |  |  |  |  |
|  | L Superior Frontal Gyrus | 2.46 | -6 | 38 | 56 |

# Supplementary Table S6. [UT_sustained _> US_sustained_]. Descriptive statistics for clusters and local maxima showing greater activation for Uncertain-Threat relative to Uncertain-Safety anticipation for the OSP Sustained regressor (FDR *q*<0.05, whole-brain corrected). 

| ***mm***^***3***^ | **Label** | ***t*** | ***x*** | ***y*** | ***z*** |
| --- | --- | --- | --- | --- | --- |
| 125,152 |  |  |  |  |  |
|  | R Frontal Operculum Cortex | 13.66 | 38 | 22 | 8 |
|  | R Frontal Orbital Cortex | 9.86 | 34 | 20 | -10 |
|  | R Central Opercular Cortex | 11.39 | 42 | 10 | 4 |
|  | R Inferior Frontal Gyrus, pars opercularis | 9.27 | 54 | 10 | 4 |
|  | R Precentral Gyrus | 6.78 | 64 | 8 | 10 |
|  | R Putamen | 9.99 | 24 | 6 | -8 |
|  | L Caudate | 7.01 | -16 | 4 | 22 |
|  | R Pallidum | 5.59 | 14 | 0 | -4 |
|  | R Caudate | 8.51 | 18 | -2 | 22 |
|  | R Thalamus | 7.42 | 10 | -2 | 10 |
|  | R Thalamus | 5.13 | 0 | -4 | 6 |
|  | L Thalamus | 6.51 | -10 | -10 | 16 |
|  | R Amygdala | 5.84 | 26 | -12 | -12 |
|  | R Brain-Stem | 7.68 | 2 | -30 | -2 |
|  | R Periaqueductal gray | 4.29 | 2 | -34 | -14 |
|  | L Brain-Stem | 6.38 | -10 | -38 | -48 |
|  | R Temporal Occipital Fusiform Cortex | 9.94 | 34 | -60 | -20 |
|  | R Lingual Gyrus | 2.96 | 6 | -86 | -10 |
| 60,160 |  |  |  |  |  |
|  | R Paracingulate Gyrus | 12.26 | 10 | 20 | 34 |
|  | L Paracingulate Gyrus | 10.42 | -8 | 12 | 38 |
|  | R Cingulate Gyrus, anterior division | 12.74 | 10 | 12 | 38 |
|  | L Cingulate Gyrus, anterior division | 10.45 | -6 | 10 | 40 |
|  | R Juxtapositional Lobule Cortex (formerly Supplementary Motor Cortex) | 11.99 | 6 | 6 | 54 |
|  | R Superior Frontal Gyrus | 10.76 | 16 | 6 | 68 |
|  | L Superior Frontal Gyrus | 10.47 | -14 | 4 | 70 |
|  | L Juxtapositional Lobule Cortex (formerly Supplementary Motor Cortex) | 10.02 | -4 | 4 | 46 |
|  | L Middle Frontal Gyrus | 5.62 | -42 | 0 | 56 |
|  | R Precentral Gyrus | 11.4 | 46 | 0 | 50 |
|  | L Precentral Gyrus | 8.03 | -40 | -4 | 48 |
|  | R Cingulate Gyrus, posterior division | 5.95 | 8 | -20 | 42 |
| 19,400 |  |  |  |  |  |
|  | L Frontal Orbital Cortex | 8.31 | -34 | 24 | -8 |
|  | L Caudate | 3.79 | -16 | 18 | 4 |
|  | L Frontal Operculum Cortex | 12.89 | -40 | 10 | 4 |
|  | L Inferior Frontal Gyrus, pars opercularis | 5.48 | -56 | 10 | 14 |
|  | L Putamen | 8.25 | -22 | 6 | -4 |
|  | L Central Opercular Cortex | 8.1 | -52 | 4 | 4 |
| 18,064 |  |  |  |  |  |
|  | R Parietal Operculum Cortex | 5.87 | 46 | -22 | 18 |
|  | R Middle Temporal Gyrus, posterior division | 8.33 | 52 | -30 | -4 |
|  | R Middle Temporal Gyrus, temporooccipital part | 5.75 | 48 | -40 | 4 |
|  | R Supramarginal Gyrus, posterior division | 10.15 | 66 | -42 | 28 |
|  | R Angular Gyrus | 8.98 | 52 | -46 | 34 |
| 8,912 |  |  |  |  |  |
|  | L Frontal Pole | 9.63 | -28 | 48 | 26 |
|  | L Middle Frontal Gyrus | 6.29 | -36 | 34 | 40 |
| 8,688 |  |  |  |  |  |
|  | L Postcentral Gyrus | 4.34 | -56 | -22 | 24 |
|  | L Parietal Operculum Cortex | 5.45 | -56 | -32 | 26 |
|  | L Supramarginal Gyrus, anterior division | 5.61 | -66 | -38 | 26 |
|  | L Supramarginal Gyrus, posterior division | 7.83 | -60 | -48 | 34 |
| 6,440 |  |  |  |  |  |
|  | R Frontal Pole | 7.43 | 32 | 44 | 28 |
|  | R Middle Frontal Gyrus | 4.74 | 36 | 34 | 38 |
| 4,112 |  |  |  |  |  |
|  | R Postcentral Gyrus | 2.49 | 36 | -36 | 52 |
|  | R Superior Parietal Lobule | 6.92 | 28 | -44 | 70 |
| 2,552 |  |  |  |  |  |
|  | R Occipital Pole | 5.73 | 24 | -96 | 14 |
| 2,088 |  |  |  |  |  |
|  | R Temporal Pole | 4.61 | 44 | 6 | -36 |
| 1,000 |  |  |  |  |  |
|  | L Temporal Pole | 4.48 | -44 | 6 | -44 |
|  | L Inferior Temporal Gyrus, anterior division | 3.66 | -44 | 0 | -36 |
|  | L Middle Temporal Gyrus, anterior division | 3.52 | -48 | -2 | -32 |
| 944 |  |  |  |  |  |
|  | L Superior Parietal Lobule | 5.06 | -22 | -50 | 64 |
| 760 |  |  |  |  |  |
|  | R Postcentral Gyrus | 4.01 | 48 | -26 | 50 |
| 448 |  |  |  |  |  |
|  | L Occipital Pole | 3.63 | -20 | -100 | 8 |
| 328 |  |  |  |  |  |
|  | R Frontal Pole | 3.67 | 28 | 58 | -8 |
| 288 |  |  |  |  |  |
|  | L Pallidum | 4.05 | -18 | -8 | -2 |
| 240 |  |  |  |  |  |
|  | L Hippocampus | 3.61 | -34 | -28 | -8 |
| 232 |  |  |  |  |  |
|  | R Occipital Fusiform Gyrus | 3.31 | 14 | -86 | -12 |
| 224 |  |  |  |  |  |
|  | R Hippocampus | 3.6 | 36 | -34 | -6 |
| 208 |  |  |  |  |  |
|  | L Cingulate Gyrus, posterior division | 3.66 | -10 | -22 | 40 |
| 184 |  |  |  |  |  |
|  | R Inferior Frontal Gyrus, pars opercularis | 2.75 | 38 | 14 | 26 |
| 128 |  |  |  |  |  |
|  | R Occipital Pole | 3.17 | 8 | -100 | -6 |
| 112 |  |  |  |  |  |
|  | L Lingual Gyrus | 3.05 | -10 | -86 | -10 |
| 72 |  |  |  |  |  |
|  | L Frontal Pole | 3.43 | -32 | 58 | -12 |
| 72 |  |  |  |  |  |
|  | L Caudate | 2.84 | -18 | -16 | 24 |
| 64 |  |  |  |  |  |
|  | R Inferior Temporal Gyrus, posterior division | 2.9 | 50 | -30 | -28 |
| 64 |  |  |  |  |  |
|  | L Amygdala | 2.8 | -24 | -10 | -12 |
| 56 |  |  |  |  |  |
|  | L Temporal Pole | 2.94 | -30 | 22 | -34 |
| 56 |  |  |  |  |  |
|  | L Subcallosal Cortex | 3.88 | -10 | 16 | -22 |
| 48 |  |  |  |  |  |
|  | L Frontal Orbital Cortex | 3.34 | -22 | 18 | -24 |
| 48 |  |  |  |  |  |
|  | R Accumbens | 3.55 | 10 | 6 | -8 |
| 40 |  |  |  |  |  |
|  | R Inferior Temporal Gyrus, posterior division | 2.96 | 56 | -10 | -40 |
| 40 |  |  |  |  |  |
|  | L Hippocampus | 3.43 | -28 | -18 | -12 |
| 40 |  |  |  |  |  |
|  | L Thalamus | 4.05 | -6 | -26 | 14 |
| 40 |  |  |  |  |  |
|  | L Precuneus Cortex | 2.71 | -12 | -46 | 50 |
| 32 |  |  |  |  |  |
|  | L Parahippocampal Gyrus, anterior division | 2.96 | -20 | 0 | -38 |
| 32 |  |  |  |  |  |
|  | R Brain-Stem | 2.73 | 6 | -22 | -20 |
| 32 |  |  |  |  |  |
|  | L Frontal Pole | 2.68 | -30 | 52 | -14 |
| 32 |  |  |  |  |  |
|  | L Frontal Pole | 2.55 | -30 | 48 | 8 |
| 32 |  |  |  |  |  |
|  | R Thalamus | 3.66 | 8 | -30 | 12 |
| 32 |  |  |  |  |  |
|  | L Superior Frontal Gyrus | 2.71 | -6 | 28 | 52 |
| 32 |  |  |  |  |  |
|  | L Superior Parietal Lobule | 3.21 | -14 | -54 | 72 |
| 24 |  |  |  |  |  |
|  | L Temporal Pole | 2.54 | -36 | 22 | -38 |
| 24 |  |  |  |  |  |
|  | R Middle Temporal Gyrus, anterior division | 2.71 | 58 | 0 | -36 |
| 24 |  |  |  |  |  |
|  | L Frontal Orbital Cortex | 2.81 | -46 | 32 | -10 |
| 24 |  |  |  |  |  |
|  | R Hippocampus | 4.15 | 34 | -28 | -6 |
| 24 |  |  |  |  |  |
|  | L Frontal Pole | 2.71 | -26 | 68 | -6 |
| 24 |  |  |  |  |  |
|  | L Middle Temporal Gyrus, temporooccipital part | 2.73 | -50 | -50 | 4 |
| 24 |  |  |  |  |  |
|  | L Postcentral Gyrus | 2.6 | -44 | -28 | 40 |
| 24 |  |  |  |  |  |
|  | R Postcentral Gyrus | 2.72 | 32 | -34 | 46 |
| 24 |  |  |  |  |  |
|  | L Superior Parietal Lobule | 2.61 | -42 | -52 | 58 |
| 16 |  |  |  |  |  |
|  | R Inferior Temporal Gyrus, anterior division | 2.87 | 48 | -4 | -42 |
| 16 |  |  |  |  |  |
|  | R Temporal Fusiform Cortex, anterior division | 2.87 | 26 | -4 | -42 |
| 16 |  |  |  |  |  |
|  | R Parahippocampal Gyrus, anterior division | 3.06 | 24 | -10 | -32 |
| 16 |  |  |  |  |  |
|  | R Putamen | 3.94 | 32 | -18 | -2 |
| 16 |  |  |  |  |  |
|  | R Thalamus | 2.47 | 8 | -8 | -2 |
| 16 |  |  |  |  |  |
|  | R Putamen | 2.57 | 30 | -18 | 8 |
| 16 |  |  |  |  |  |
|  | R Thalamus | 2.89 | 12 | -34 | 8 |
| 16 |  |  |  |  |  |
|  | L Precentral Gyrus | 3 | -50 | 2 | 28 |
| 8 |  |  |  |  |  |
|  | R Parahippocampal Gyrus, anterior division | 2.69 | 22 | -8 | -36 |
| 8 |  |  |  |  |  |
|  | R Inferior Temporal Gyrus, anterior division | 2.4 | 54 | -6 | -34 |
| 8 |  |  |  |  |  |
|  | L Temporal Pole | 3.32 | -32 | 12 | -34 |
| 8 |  |  |  |  |  |
|  | R Inferior Temporal Gyrus, temporooccipital part | 2.62 | 58 | -42 | -20 |
| 8 |  |  |  |  |  |
|  | R Lateral Occipital Cortex, inferior division | 2.4 | 38 | -88 | -12 |
| 8 |  |  |  |  |  |
|  | R Thalamus | 2.36 | 22 | -34 | 0 |
| 8 |  |  |  |  |  |
|  | L Pallidum | 2.46 | -12 | 2 | 0 |
| 8 |  |  |  |  |  |
|  | L Paracingulate Gyrus | 2.38 | -10 | 54 | 12 |
| 8 |  |  |  |  |  |
|  | R Supramarginal Gyrus, anterior division | 2.58 | 52 | -22 | 32 |
| 8 |  |  |  |  |  |
|  | R Superior Frontal Gyrus | 2.36 | 2 | 54 | 36 |
| 8 |  |  |  |  |  |
|  | L Superior Parietal Lobule | 2.36 | -30 | -46 | 50 |
| 8 |  |  |  |  |  |
|  | L Bed Nucleus of the Stria Terminalis | 5.68 | -6 | 2 | -4 |

# Supplementary Table S7. [CT_sustained _> CS_sustained_]. Descriptive statistics for clusters and local maxima showing greater activation for Certain-Threat relative to Certain-Safety anticipation for the OSP Sustained regressor (FDR *q*<0.05, whole-brain corrected). 

| ***mm***^***3***^ | **Label** | ***t*** | ***x*** | ***y*** | ***z*** |
| --- | --- | --- | --- | --- | --- |
| 14,880 |  |  |  |  |  |
|  | L Cingulate Gyrus, anterior division | 4.82 | -8 | 24 | 30 |
|  | L Paracingulate Gyrus | 5.78 | -8 | 20 | 34 |
|  | R Paracingulate Gyrus | 5.18 | 10 | 18 | 36 |
|  | L Juxtapositional Lobule Cortex (formerly Supplementary Motor Cortex) | 5.37 | -6 | 6 | 58 |
|  | R Juxtapositional Lobule Cortex (formerly Supplementary Motor Cortex) | 6.01 | 6 | 6 | 70 |
|  | L Superior Frontal Gyrus | 6.07 | -16 | 4 | 68 |
|  | R Superior Frontal Gyrus | 7.39 | 14 | 4 | 72 |
|  | L Middle Frontal Gyrus | 3.21 | -36 | -4 | 62 |
|  | L Precentral Gyrus | 3.34 | -32 | -6 | 68 |
|  | R Precentral Gyrus | 4.62 | 32 | -8 | 66 |
| 5,264 |  |  |  |  |  |
|  | L Postcentral Gyrus | 3.43 | -40 | -32 | 48 |
|  | L Superior Parietal Lobule | 3.38 | -34 | -46 | 46 |
|  | L Supramarginal Gyrus, posterior division | 5.58 | -56 | -52 | 38 |
|  | L Angular Gyrus | 4.27 | -44 | -54 | 56 |
|  | L Lateral Occipital Cortex, superior division | 3.99 | -34 | -60 | 54 |
| 4,864 |  |  |  |  |  |
|  | R Frontal Pole | 4.65 | 50 | 34 | -4 |
|  | R Frontal Orbital Cortex | 4.50 | 38 | 30 | -2 |
|  | R Inferior Frontal Gyrus, pars triangularis | 3.73 | 54 | 24 | 6 |
|  | R Frontal Operculum Cortex | 6.78 | 48 | 18 | 0 |
|  | R Inferior Frontal Gyrus, pars opercularis | 3.60 | 56 | 16 | 6 |
|  | R Central Opercular Cortex | 4.54 | 50 | 8 | 2 |
| 4,336 |  |  |  |  |  |
|  | L Frontal Pole | 6.63 | -40 | 54 | 14 |
|  | L Middle Frontal Gyrus | 4.10 | -34 | 28 | 38 |
| 3,968 |  |  |  |  |  |
|  | R Postcentral Gyrus | 3.68 | 44 | -28 | 48 |
|  | R Supramarginal Gyrus, anterior division | 4.10 | 46 | -32 | 44 |
|  | R Supramarginal Gyrus, posterior division | 5.01 | 64 | -42 | 28 |
|  | R Superior Parietal Lobule | 4.14 | 44 | -46 | 58 |
|  | R Angular Gyrus | 3.82 | 50 | -48 | 34 |
| 3,168 |  |  |  |  |  |
|  | R Frontal Pole | 5.36 | 32 | 52 | 28 |
| 2,408 |  |  |  |  |  |
|  | L Inferior Frontal Gyrus, pars triangularis | 3.45 | -52 | 20 | -2 |
|  | L Frontal Operculum Cortex | 6.77 | -38 | 14 | 6 |
|  | L Inferior Frontal Gyrus, pars opercularis | 3.67 | -52 | 14 | 2 |
| 1,376 |  |  |  |  |  |
|  | R Middle Frontal Gyrus | 3.33 | 46 | 8 | 46 |
|  | R Precentral Gyrus | 4.95 | 50 | 2 | 52 |
| 1,288 |  |  |  |  |  |
|  | R Superior Temporal Gyrus, posterior division | 4.86 | 48 | -26 | -4 |
|  | R Middle Temporal Gyrus, posterior division | 4.15 | 50 | -28 | -8 |
|  | R Supramarginal Gyrus, posterior division | 3.40 | 46 | -38 | 8 |
|  | R Middle Temporal Gyrus, temporooccipital part | 4.59 | 52 | -42 | 4 |
| 968 |  |  |  |  |  |
|  | R Brain-Stem | 5.75 | 6 | -46 | -60 |
|  | L Brain-Stem | 3.54 | -6 | -48 | -58 |
| 544 |  |  |  |  |  |
|  | L Precentral Gyrus | 3.98 | -54 | 0 | 42 |
| 432 |  |  |  |  |  |
|  | R Caudate | 6.01 | 18 | -6 | 24 |
| 248 |  |  |  |  |  |
|  | R Putamen | 3.61 | 18 | 8 | -8 |
| 240 |  |  |  |  |  |
|  | R Superior Frontal Gyrus | 3.86 | 12 | 28 | 58 |
| 216 |  |  |  |  |  |
|  | L Frontal Pole | 3.74 | -44 | 56 | -8 |
| 192 |  |  |  |  |  |
|  | L Brain-Stem | 3.87 | -4 | -30 | -54 |
| 192 |  |  |  |  |  |
|  | L Caudate | 4.86 | -16 | 2 | 22 |
| 176 |  |  |  |  |  |
|  | R Frontal Pole | 4.01 | 32 | 60 | -6 |
| 176 |  |  |  |  |  |
|  | R Thalamus | 4.40 | 2 | -18 | 0 |
| 160 |  |  |  |  |  |
|  | R Brain-Stem | 4.73 | 10 | -32 | -16 |
| 160 |  |  |  |  |  |
|  | L Putamen | 3.84 | -22 | 14 | -4 |
| 160 |  |  |  |  |  |
|  | R Occipital Pole | 4.23 | 22 | -102 | 12 |
| 152 |  |  |  |  |  |
|  | R Amygdala | 3.53 | 30 | -4 | -20 |
|  | R Amygdala | 4.26 | 30 | -8 | -16 |
| 144 |  |  |  |  |  |
|  | L Frontal Orbital Cortex | 4.13 | -34 | 30 | -2 |
| 144 |  |  |  |  |  |
|  | R Frontal Pole | 3.82 | 52 | 46 | 0 |
| 136 |  |  |  |  |  |
|  | L Frontal Pole | 4.10 | -50 | 42 | -2 |
| 104 |  |  |  |  |  |
|  | L Periaqueductal Grey | 4.38 | -2 | -36 | -16 |
| 104 |  |  |  |  |  |
|  | R Frontal Pole | 3.83 | 18 | 54 | 30 |
| 88 |  |  |  |  |  |
|  | L Brain-Stem | 4.18 | -6 | -44 | -64 |
| 88 |  |  |  |  |  |
|  | R Brain-Stem | 3.77 | 0 | -28 | -4 |
| 88 |  |  |  |  |  |
|  | L Precentral Gyrus | 3.48 | -28 | -8 | 48 |
| 80 |  |  |  |  |  |
|  | L Brain-Stem | 3.36 | -6 | -38 | -62 |
| 80 |  |  |  |  |  |
|  | R Thalamus | 3.30 | 22 | -34 | -2 |
|  | R Hippocampus | 4.46 | 20 | -36 | 6 |
| 72 |  |  |  |  |  |
|  | R Superior Temporal Gyrus, posterior division | 3.66 | 56 | -20 | -4 |
| 64 |  |  |  |  |  |
|  | L Frontal Pole | 3.72 | -32 | 48 | -16 |
| 64 |  |  |  |  |  |
|  | R Accumbens | 3.05 | 6 | 6 | -6 |
| 64 |  |  |  |  |  |
|  | R Putamen | 3.20 | 24 | 16 | -6 |
| 64 |  |  |  |  |  |
|  | R Putamen | 3.81 | 24 | 14 | 4 |
| 64 |  |  |  |  |  |
|  | L Caudate | 4.26 | -18 | 16 | 12 |
| 56 |  |  |  |  |  |
|  | L Temporal Fusiform Cortex, anterior division | 4.38 | -32 | 0 | -50 |
| 56 |  |  |  |  |  |
|  | R Thalamus | 4.12 | 0 | -4 | 6 |
| 56 |  |  |  |  |  |
|  | R Thalamus | 4.14 | 6 | -30 | 8 |
| 56 |  |  |  |  |  |
|  | R Frontal Pole | 3.52 | 34 | 36 | 40 |
| 48 |  |  |  |  |  |
|  | L Amygdala | 4.05 | -24 | -12 | -10 |
| 48 |  |  |  |  |  |
|  | R Putamen | 3.64 | 20 | 6 | 6 |
| 48 |  |  |  |  |  |
|  | L Frontal Operculum Cortex | 3.68 | -36 | 26 | 8 |
| 48 |  |  |  |  |  |
|  | L Superior Frontal Gyrus | 3.31 | -2 | 18 | 62 |
| 40 |  |  |  |  |  |
|  | L Brain-Stem | 3.33 | -6 | -38 | -30 |
| 40 |  |  |  |  |  |
|  | L Putamen | 3.04 | -16 | 8 | -10 |
| 40 |  |  |  |  |  |
|  | R Thalamus | 4.87 | 12 | -34 | 8 |
| 40 |  |  |  |  |  |
|  | R Superior Parietal Lobule | 3.61 | 38 | -48 | 68 |
| 40 |  |  |  |  |  |
|  | L Superior Parietal Lobule | 3.20 | -34 | -46 | 68 |
| 32 |  |  |  |  |  |
|  | L Brain-Stem | 3.17 | -14 | -26 | -14 |
| 32 |  |  |  |  |  |
|  | R Temporal Pole | 3.25 | 50 | 20 | -16 |
| 32 |  |  |  |  |  |
|  | R Lateral Occipital Cortex, inferior division | 3.40 | 48 | -72 | -12 |
| 32 |  |  |  |  |  |
|  | L Frontal Pole | 3.27 | -50 | 46 | -12 |
| 32 |  |  |  |  |  |
|  | R Hippocampus | 3.71 | 36 | -34 | -6 |
| 32 |  |  |  |  |  |
|  | L Postcentral Gyrus | 2.89 | -48 | -28 | 40 |
|  | L Supramarginal Gyrus, anterior division | 2.89 | -50 | -30 | 38 |
| 24 |  |  |  |  |  |
|  | R Frontal Pole | 3.30 | 24 | 60 | -12 |
| 24 |  |  |  |  |  |
|  | R Middle Temporal Gyrus, posterior division | 2.97 | 60 | -24 | -8 |
| 24 |  |  |  |  |  |
|  | R Thalamus | 3.95 | 0 | -20 | 10 |
| 24 |  |  |  |  |  |
|  | L Cingulate Gyrus, anterior division | 3.22 | -4 | 24 | 20 |
| 24 |  |  |  |  |  |
|  | R Angular Gyrus | 2.84 | 52 | -52 | 48 |
| 16 |  |  |  |  |  |
|  | L Brain-Stem | 3.08 | -2 | -32 | -60 |
| 16 |  |  |  |  |  |
|  | R Brain-Stem | 3.35 | 2 | -30 | -54 |
| 16 |  |  |  |  |  |
|  | R Temporal Pole | 3.21 | 30 | 24 | -36 |
| 16 |  |  |  |  |  |
|  | R Inferior Temporal Gyrus, posterior division | 2.94 | 58 | -36 | -20 |
| 16 |  |  |  |  |  |
|  | L Inferior Temporal Gyrus, posterior division | 3.59 | -54 | -36 | -18 |
| 16 |  |  |  |  |  |
|  | L Brain-Stem | 3.39 | -10 | -34 | -14 |
| 16 |  |  |  |  |  |
|  | R Frontal Orbital Cortex | 2.86 | 30 | 22 | -14 |
| 16 |  |  |  |  |  |
|  | L Frontal Pole | 3.23 | -42 | 54 | -14 |
| 16 |  |  |  |  |  |
|  | L Pallidum | 3.17 | -14 | -2 | -4 |
| 16 |  |  |  |  |  |
|  | L Caudate | 2.82 | -18 | 20 | -2 |
| 16 |  |  |  |  |  |
|  | R Caudate | 3.02 | 18 | 14 | 12 |
| 16 |  |  |  |  |  |
|  | R Caudate | 2.98 | 12 | 0 | 20 |
| 16 |  |  |  |  |  |
|  | R Supramarginal Gyrus, posterior division | 2.89 | 40 | -40 | 38 |
| 8 |  |  |  |  |  |
|  | L Temporal Pole | 2.91 | -38 | 22 | -40 |
| 8 |  |  |  |  |  |
|  | L Temporal Pole | 2.79 | -34 | 14 | -34 |
| 8 |  |  |  |  |  |
|  | R Brain-Stem | 2.78 | 6 | -42 | -26 |
| 8 |  |  |  |  |  |
|  | L Brain-Stem | 2.83 | -10 | -38 | -26 |
| 8 |  |  |  |  |  |
|  | L Subcallosal Cortex | 2.88 | -8 | 28 | -24 |
| 8 |  |  |  |  |  |
|  | L Brain-Stem | 2.85 | -12 | -26 | -20 |
| 8 |  |  |  |  |  |
|  | R Inferior Temporal Gyrus, temporooccipital part | 2.88 | 62 | -46 | -16 |
| 8 |  |  |  |  |  |
|  | L Frontal Orbital Cortex | 2.91 | -36 | 22 | -16 |
| 8 |  |  |  |  |  |
|  | L Hippocampus | 2.86 | -28 | -14 | -14 |
| 8 |  |  |  |  |  |
|  | R Frontal Pole | 2.81 | 42 | 56 | -10 |
| 8 |  |  |  |  |  |
|  | R Hippocampus | 2.86 | 34 | -26 | -8 |
| 8 |  |  |  |  |  |
|  | L Middle Temporal Gyrus, posterior division | 3.03 | -54 | -32 | -6 |
| 8 |  |  |  |  |  |
|  | R Insular Cortex | 2.80 | 38 | 10 | -6 |
| 8 |  |  |  |  |  |
|  | L Frontal Orbital Cortex | 2.89 | -32 | 26 | -6 |
| 8 |  |  |  |  |  |
|  | L Lateral Occipital Cortex, inferior division | 3.09 | -42 | -80 | -4 |
| 8 |  |  |  |  |  |
|  | R Hippocampus | 3.23 | 24 | -32 | -4 |
| 8 |  |  |  |  |  |
|  | L Pallidum | 3.14 | -20 | -10 | -4 |
| 8 |  |  |  |  |  |
|  | L Middle Temporal Gyrus, posterior division | 2.97 | -52 | -42 | -2 |
| 8 |  |  |  |  |  |
|  | L Pallidum | 2.92 | -16 | -6 | -2 |
| 8 |  |  |  |  |  |
|  | L Thalamus | 2.85 | -2 | -2 | 0 |
| 8 |  |  |  |  |  |
|  | L Thalamus | 3.12 | -20 | -36 | 2 |
| 8 |  |  |  |  |  |
|  | R Occipital Pole | 2.84 | 32 | -96 | 6 |
| 8 |  |  |  |  |  |
|  | L Thalamus | 2.82 | -12 | -10 | 6 |
| 8 |  |  |  |  |  |
|  | L Frontal Pole | 2.78 | -32 | 64 | 6 |
| 8 |  |  |  |  |  |
|  | L Supramarginal Gyrus, posterior division | 3.33 | -54 | -44 | 20 |
| 8 |  |  |  |  |  |
|  | R Precentral Gyrus | 3.36 | 54 | 4 | 20 |
| 8 |  |  |  |  |  |
|  | R Caudate | 2.89 | 18 | -20 | 22 |
| 8 |  |  |  |  |  |
|  | L Middle Frontal Gyrus | 3.29 | -40 | 30 | 26 |
| 8 |  |  |  |  |  |
|  | L Paracingulate Gyrus | 3.01 | -6 | 20 | 46 |
| 8 |  |  |  |  |  |
|  | L Superior Frontal Gyrus | 2.99 | -14 | 30 | 52 |
| 8 |  |  |  |  |  |
|  | R Frontal Pole | 2.80 | 10 | 40 | 56 |
| 8 |  |  |  |  |  |
|  | R Precentral Gyrus | 2.79 | 28 | -12 | 60 |
| 8 |  |  |  |  |  |
|  | R Superior Parietal Lobule | 3.06 | 28 | -48 | 62 |
| 8 |  |  |  |  |  |
|  | R Precentral Gyrus | 2.87 | 40 | -20 | 62 |
| 8 |  |  |  |  |  |
|  | R Superior Frontal Gyrus | 3.01 | 24 | 8 | 64 |
| 8 |  |  |  |  |  |
|  | R Lateral Occipital Cortex, superior division | 2.83 | 8 | -66 | 66 |
| 8 |  |  |  |  |  |
|  | R Superior Parietal Lobule | 2.83 | 14 | -52 | 76 |

# Supplementary Table S8. [UT_sustained _> US_sustained_] $\boldsymbol{∩}$ [CT_sustained _> CS_sustained_]. Descriptive statistics for clusters and local maxima greater activation for Uncertain-Threat relative to Uncertain-Safety anticipation for the OSP Sustained regressor AND greater sustained activation for Certain-Threat relative to Certain-Safety anticipation for the OSP Sustained regressor (FDR *q*<0.05, whole-brain corrected). 

| **mm**^**3**^ | **Label** | ***t*** | ***x*** | ***y*** | ***z*** |
| --- | --- | --- | --- | --- | --- |
| 14,616 |  |  |  |  |  |
|  | L Cingulate Gyrus, anterior division | 4.82 | -8 | 24 | 30 |
|  | L Paracingulate Gyrus | 5.78 | -8 | 20 | 34 |
|  | R Paracingulate Gyrus | 5.18 | 10 | 18 | 36 |
|  | L Juxtapositional Lobule Cortex (formerly Supplementary Motor Cortex) | 5.28 | -4 | 6 | 58 |
|  | R Juxtapositional Lobule Cortex (formerly Supplementary Motor Cortex) | 6.01 | 6 | 6 | 70 |
|  | L Superior Frontal Gyrus | 6.07 | -16 | 4 | 68 |
|  | R Superior Frontal Gyrus | 7.39 | 14 | 4 | 72 |
|  | L Middle Frontal Gyrus | 3.21 | -36 | -4 | 62 |
|  | L Precentral Gyrus | 3.34 | -32 | -6 | 68 |
|  | R Precentral Gyrus | 4.62 | 32 | -8 | 66 |
| 4,856 |  |  |  |  |  |
|  | R Frontal Pole | 4.63 | 50 | 34 | -4 |
|  | R Frontal Orbital Cortex | 4.50 | 38 | 30 | -2 |
|  | R Inferior Frontal Gyrus, pars triangularis | 3.73 | 54 | 24 | 6 |
|  | R Frontal Operculum Cortex | 6.78 | 48 | 18 | 0 |
|  | R Inferior Frontal Gyrus, pars opercularis | 3.60 | 56 | 16 | 6 |
|  | R Central Opercular Cortex | 4.54 | 50 | 8 | 2 |
| 3,616 |  |  |  |  |  |
|  | L Frontal Pole | 5.60 | -34 | 54 | 24 |
|  | L Middle Frontal Gyrus | 4.10 | -34 | 28 | 38 |
| 2,624 |  |  |  |  |  |
|  | R Frontal Pole | 5.36 | 32 | 52 | 28 |
| 2,384 |  |  |  |  |  |
|  | L Inferior Frontal Gyrus, pars triangularis | 3.45 | -52 | 20 | -2 |
|  | L Frontal Operculum Cortex | 6.77 | -38 | 14 | 6 |
|  | L Inferior Frontal Gyrus, pars opercularis | 3.67 | -52 | 14 | 2 |
| 2,168 |  |  |  |  |  |
|  | L Supramarginal Gyrus, posterior division | 5.36 | -56 | -52 | 38 |
|  | L Angular Gyrus | 3.25 | -52 | -56 | 50 |
| 1,448 |  |  |  |  |  |
|  | R Supramarginal Gyrus, posterior division | 5.01 | 64 | -42 | 28 |
|  | R Angular Gyrus | 3.82 | 50 | -48 | 34 |
| 1,376 |  |  |  |  |  |
|  | R Middle Frontal Gyrus | 3.33 | 46 | 8 | 46 |
|  | R Precentral Gyrus | 4.95 | 50 | 2 | 52 |
| 1,248 |  |  |  |  |  |
|  | R Superior Temporal Gyrus, posterior division | 4.86 | 48 | -26 | -4 |
|  | R Middle Temporal Gyrus, posterior division | 4.15 | 50 | -28 | -8 |
|  | R Supramarginal Gyrus, posterior division | 3.38 | 48 | -38 | 8 |
| 816 |  |  |  |  |  |
|  | R Brain-Stem | 5.20 | 6 | -48 | -60 |
| 432 |  |  |  |  |  |
|  | R Caudate | 6.01 | 18 | -6 | 24 |
| 328 |  |  |  |  |  |
|  | R Postcentral Gyrus | 3.28 | 48 | -24 | 42 |
| 272 |  |  |  |  |  |
|  | L Precentral Gyrus | 3.72 | -48 | 2 | 40 |
| 232 |  |  |  |  |  |
|  | R Putamen | 3.61 | 18 | 8 | -8 |
| 216 |  |  |  |  |  |
|  | R Postcentral Gyrus | 3.93 | 38 | -38 | 66 |
|  | R Superior Parietal Lobule | 2.52 | 38 | -44 | 62 |
| 184 |  |  |  |  |  |
|  | L Brain-Stem | 3.87 | -4 | -30 | -54 |
| 176 |  |  |  |  |  |
|  | L Caudate | 4.86 | -16 | 2 | 22 |
| 160 |  |  |  |  |  |
|  | R Occipital Pole | 4.03 | 22 | -102 | 12 |
| 152 |  |  |  |  |  |
|  | L Putamen | 3.84 | -22 | 14 | -4 |
| 144 |  |  |  |  |  |
|  | L Frontal Orbital Cortex | 4.13 | -34 | 30 | -2 |
| 136 |  |  |  |  |  |
|  | R Superior Frontal Gyrus | 3.61 | 10 | 30 | 56 |
| 104 |  |  |  |  |  |
|  | L Brain-Stem | 3.54 | -6 | -48 | -58 |
| 104 |  |  |  |  |  |
|  | R Brain-Stem | 4.58 | 12 | -28 | -14 |
| 88 |  |  |  |  |  |
|  | L Brain-Stem | 4.18 | -6 | -44 | -64 |
| 88 |  |  |  |  |  |
|  | R Brain-Stem | 3.77 | 0 | -28 | -4 |
| 80 |  |  |  |  |  |
|  | L Brain-Stem | 3.36 | -6 | -38 | -62 |
| 80 |  |  |  |  |  |
|  | L Precentral Gyrus | 3.48 | -28 | -8 | 48 |
| 64 |  |  |  |  |  |
|  | R Superior Temporal Gyrus, posterior division | 3.66 | 56 | -20 | -4 |
| 64 |  |  |  |  |  |
|  | R Thalamus | 4.24 | 2 | -20 | 0 |
| 64 |  |  |  |  |  |
|  | R Putamen | 3.81 | 24 | 14 | 4 |
| 56 |  |  |  |  |  |
|  | R Thalamus | 4.12 | 0 | -4 | 6 |
| 48 |  |  |  |  |  |
|  | R Brain-Stem | 3.24 | 0 | -36 | -14 |
| 48 |  |  |  |  |  |
|  | R Putamen | 2.99 | 20 | 14 | -4 |
| 48 |  |  |  |  |  |
|  | R Putamen | 3.64 | 20 | 6 | 6 |
| 48 |  |  |  |  |  |
|  | L Frontal Operculum Cortex | 3.68 | -36 | 26 | 8 |
| 40 |  |  |  |  |  |
|  | R Brain-Stem | 3.97 | 12 | -16 | -22 |
| 40 |  |  |  |  |  |
|  | R Frontal Pole | 3.52 | 34 | 36 | 40 |
| 40 |  |  |  |  |  |
|  | R Superior Parietal Lobule | 3.50 | 38 | -46 | 68 |
| 32 |  |  |  |  |  |
|  | L Brain-Stem | 3.17 | -14 | -26 | -14 |
| 32 |  |  |  |  |  |
|  | L Amygdala | 2.80 | -24 | -10 | -12 |
| 32 |  |  |  |  |  |
|  | L Caudate | 3.56 | -18 | 16 | 12 |
| 32 |  |  |  |  |  |
|  | L Superior Frontal Gyrus | 2.86 | -4 | 20 | 64 |
| 24 |  |  |  |  |  |
|  | R Amygdala | 3.21 | 28 | -12 | -14 |
| 24 |  |  |  |  |  |
|  | L Putamen | 2.94 | -16 | 10 | -8 |
| 24 |  |  |  |  |  |
|  | R Middle Temporal Gyrus, posterior division | 2.97 | 60 | -24 | -8 |
| 16 |  |  |  |  |  |
|  | L Brain-Stem | 3.08 | -2 | -32 | -60 |
| 16 |  |  |  |  |  |
|  | R Brain-Stem | 3.35 | 2 | -30 | -54 |
| 16 |  |  |  |  |  |
|  | L Brain-Stem | 3.09 | -4 | -42 | -26 |
| 16 |  |  |  |  |  |
|  | R Frontal Orbital Cortex | 2.86 | 30 | 22 | -14 |
| 16 |  |  |  |  |  |
|  | R Accumbens | 2.96 | 8 | 6 | -8 |
| 16 |  |  |  |  |  |
|  | R Hippocampus | 3.60 | 36 | -34 | -6 |
| 16 |  |  |  |  |  |
|  | R Frontal Pole | 2.84 | 32 | 58 | -6 |
| 16 |  |  |  |  |  |
|  | L Pallidum | 2.81 | -14 | -4 | -4 |
| 16 |  |  |  |  |  |
|  | R Thalamus | 2.89 | 12 | -34 | 8 |
| 16 |  |  |  |  |  |
|  | R Caudate | 3.02 | 18 | 14 | 12 |
| 16 |  |  |  |  |  |
|  | R Caudate | 2.98 | 12 | 0 | 20 |
| 16 |  |  |  |  |  |
|  | L Superior Parietal Lobule | 2.61 | -42 | -52 | 58 |
| 8 |  |  |  |  |  |
|  | L Brain-Stem | 3.33 | -6 | -38 | -30 |
| 8 |  |  |  |  |  |
|  | R Brain-Stem | 2.78 | 6 | -42 | -26 |
| 8 |  |  |  |  |  |
|  | L Brain-Stem | 2.85 | -12 | -26 | -20 |
| 8 |  |  |  |  |  |
|  | L Frontal Orbital Cortex | 2.89 | -32 | 26 | -6 |
| 8 |  |  |  |  |  |
|  | L Pallidum | 3.14 | -20 | -10 | -4 |
| 8 |  |  |  |  |  |
|  | L Pallidum | 2.92 | -16 | -6 | -2 |
| 8 |  |  |  |  |  |
|  | L Caudate | 2.68 | -18 | 18 | -2 |
| 8 |  |  |  |  |  |
|  | R Thalamus | 2.36 | 22 | -34 | 0 |
| 8 |  |  |  |  |  |
|  | L Thalamus | 2.82 | -12 | -10 | 6 |
| 8 |  |  |  |  |  |
|  | R Thalamus | 2.83 | 6 | -30 | 10 |
| 8 |  |  |  |  |  |
|  | R Caudate | 2.86 | 18 | -20 | 22 |
| 8 |  |  |  |  |  |
|  | R Precentral Gyrus | 2.79 | 28 | -12 | 60 |
| 8 |  |  |  |  |  |
|  | R Superior Parietal Lobule | 2.92 | 28 | -48 | 62 |
| 8 |  |  |  |  |  |
|  | R Superior Frontal Gyrus | 3.01 | 24 | 8 | 64 |
| 8 |  |  |  |  |  |
|  | L Superior Parietal Lobule | 2.78 | -34 | -48 | 68 |

# Supplementary Table S9. [UT_sustained _> CT_sustained_]. Descriptive statistics for clusters and local maxima showing greater sustained activation for Uncertain-Threat relative to Certain-Threat anticipation for the OSP Sustained regressor (FDR *q*<0.05, whole-brain corrected). 

| ***mm***^***3***^ | **Label** | ***t*** | ***x*** | ***y*** | ***z*** |
| --- | --- | --- | --- | --- | --- |
| 62,312 |  |  |  |  |  |
|  | L Paracingulate Gyrus | 7.49 | -4 | 34 | 26 |
|  | R Paracingulate Gyrus | 11.43 | 2 | 18 | 38 |
|  | R Cingulate Gyrus, anterior division | 11.87 | 0 | 10 | 42 |
|  | R Middle Frontal Gyrus | 6.99 | 48 | 10 | 50 |
|  | L Cingulate Gyrus, anterior division | 9.79 | -8 | 6 | 40 |
|  | R Juxtapositional Lobule Cortex (formerly Supplementary Motor Cortex) | 12.01 | 6 | 6 | 48 |
|  | R Precentral Gyrus | 11.49 | 42 | -2 | 54 |
|  | L Precentral Gyrus | 5.67 | -36 | -4 | 48 |
|  | L Superior Frontal Gyrus | 10.40 | -12 | -8 | 68 |
|  | R Superior Frontal Gyrus | 11.88 | 18 | -10 | 70 |
|  | L Cingulate Gyrus, posterior division | 5.64 | -2 | -18 | 28 |
|  | R Cingulate Gyrus, posterior division | 7.64 | 8 | -22 | 46 |
| 34,376 |  |  |  |  |  |
|  | R Inferior Temporal Gyrus, temporooccipital part | 3.81 | 46 | -44 | -14 |
|  | R Temporal Occipital Fusiform Cortex | 5.21 | 36 | -48 | -16 |
|  | R Middle Temporal Gyrus, temporooccipital part | 2.66 | 54 | -52 | 4 |
|  | R Lateral Occipital Cortex, inferior division | 10.89 | 42 | -82 | -14 |
|  | R Occipital Fusiform Gyrus | 10.86 | 30 | -82 | -14 |
|  | R Occipital Pole | 11.63 | 26 | -96 | 2 |
| 25,008 |  |  |  |  |  |
|  | R Frontal Pole | 4.30 | 54 | 38 | -6 |
|  | R Frontal Orbital Cortex | 14.02 | 34 | 28 | 2 |
|  | R Frontal Operculum Cortex | 12.74 | 42 | 24 | 4 |
|  | R Inferior Frontal Gyrus, pars triangularis | 9.28 | 54 | 22 | 2 |
|  | R Inferior Frontal Gyrus, pars opercularis | 8.87 | 56 | 14 | 0 |
|  | R Central Opercular Cortex | 9.74 | 54 | 4 | 4 |
|  | R Precentral Gyrus | 9.37 | 58 | 4 | 10 |
|  | R Putamen | 10.99 | 26 | 2 | -6 |
|  | R Pallidum | 3.66 | 16 | 2 | 0 |
| 22,848 |  |  |  |  |  |
|  | L Accumbens | 2.93 | -6 | 8 | -10 |
|  | L Caudate | 5.10 | -8 | 0 | 10 |
|  | R Thalamus | 6.96 | 12 | -4 | 14 |
|  | L Thalamus | 5.12 | -6 | -6 | 8 |
|  | R Caudate | 5.01 | 16 | -8 | 20 |
|  | L Brain-Stem | 5.31 | -6 | -30 | -8 |
|  | R Brain-Stem | 7.03 | 6 | -30 | -8 |
|  | R Lingual Gyrus | 2.57 | 2 | -74 | -8 |
| 20,160 |  |  |  |  |  |
|  | L Frontal Orbital Cortex | 9.12 | -30 | 22 | -8 |
|  | L Frontal Operculum Cortex | 13.62 | -36 | 18 | 8 |
|  | L Inferior Frontal Gyrus, pars opercularis | 4.24 | -58 | 12 | 16 |
|  | L Precentral Gyrus | 7.34 | -56 | 8 | 8 |
|  | L Putamen | 9.11 | -24 | 6 | -6 |
|  | L Insular Cortex | 7.20 | -40 | 4 | 4 |
|  | L Central Opercular Cortex | 8.21 | -46 | 0 | 8 |
| 12,160 |  |  |  |  |  |
|  | R Parietal Operculum Cortex | 8.36 | 46 | -20 | 18 |
|  | R Supramarginal Gyrus, posterior division | 8.44 | 66 | -44 | 30 |
|  | R Angular Gyrus | 8.51 | 64 | -48 | 32 |
| 11,912 |  |  |  |  |  |
|  | L Occipital Fusiform Gyrus | 6.46 | -20 | -86 | -10 |
|  | L Lateral Occipital Cortex, inferior division | 5.39 | -32 | -88 | -12 |
|  | L Occipital Pole | 8.80 | -12 | -94 | -8 |
| 7,744 |  |  |  |  |  |
|  | L Parietal Operculum Cortex | 5.66 | -56 | -32 | 26 |
|  | L Supramarginal Gyrus, anterior division | 5.29 | -58 | -36 | 30 |
|  | L Supramarginal Gyrus, posterior division | 7.09 | -64 | -44 | 36 |
|  | L Angular Gyrus | 2.67 | -46 | -54 | 46 |
| 5,816 |  |  |  |  |  |
|  | L Frontal Pole | 7.25 | -36 | 46 | 26 |
|  | L Middle Frontal Gyrus | 5.20 | -40 | 36 | 36 |
| 4,200 |  |  |  |  |  |
|  | R Postcentral Gyrus | 6.58 | 34 | -38 | 62 |
|  | R Superior Parietal Lobule | 7.70 | 24 | -44 | 64 |
| 2,736 |  |  |  |  |  |
|  | R Frontal Pole | 4.57 | 34 | 52 | 30 |
|  | R Middle Frontal Gyrus | 4.00 | 32 | 34 | 28 |
| 1,472 |  |  |  |  |  |
|  | R Middle Temporal Gyrus, posterior division | 4.95 | 54 | -28 | -4 |
| 1,112 |  |  |  |  |  |
|  | R Temporal Pole | 4.39 | 44 | 6 | -36 |
|  | R Middle Temporal Gyrus, anterior division | 3.61 | 48 | 2 | -32 |
|  | R Inferior Temporal Gyrus, anterior division | 3.11 | 40 | 2 | -42 |
| 720 |  |  |  |  |  |
|  | L Temporal Pole | 4.53 | -44 | 6 | -42 |
|  | L Inferior Temporal Gyrus, anterior division | 3.06 | -50 | -2 | -34 |
| 480 |  |  |  |  |  |
|  | L Superior Parietal Lobule | 4.68 | -20 | -50 | 66 |
| 328 |  |  |  |  |  |
|  | R Postcentral Gyrus | 3.88 | 46 | -26 | 50 |
| 200 |  |  |  |  |  |
|  | L Cingulate Gyrus, posterior division | 4.22 | -12 | -26 | 40 |
| 184 |  |  |  |  |  |
|  | R Frontal Pole | 4.12 | 12 | 36 | -20 |
| 136 |  |  |  |  |  |
|  | L Frontal Orbital Cortex | 4.05 | -22 | 18 | -26 |
| 112 |  |  |  |  |  |
|  | L Precuneus Cortex | 3.41 | -12 | -68 | 40 |
| 72 |  |  |  |  |  |
|  | L Frontal Pole | 3.35 | -30 | 58 | -12 |
| 56 |  |  |  |  |  |
|  | R Amygdala | 3.40 | 26 | -10 | -12 |
| 56 |  |  |  |  |  |
|  | L Frontal Pole | 3.04 | -22 | 68 | -2 |
| 48 |  |  |  |  |  |
|  | R Frontal Pole | 2.77 | 16 | 34 | -26 |
| 48 |  |  |  |  |  |
|  | L Pallidum | 3.93 | -12 | 2 | 0 |
| 48 |  |  |  |  |  |
|  | L Frontal Pole | 2.96 | -32 | 66 | -2 |
| 40 |  |  |  |  |  |
|  | L Frontal Orbital Cortex | 2.98 | -18 | 10 | -22 |
| 32 |  |  |  |  |  |
|  | L Temporal Fusiform Cortex, anterior division | 2.81 | -36 | -2 | -46 |
| 32 |  |  |  |  |  |
|  | R Brain-Stem | 2.79 | 12 | -14 | -28 |
| 32 |  |  |  |  |  |
|  | R Frontal Pole | 2.70 | 26 | 60 | -8 |
| 32 |  |  |  |  |  |
|  | L Precentral Gyrus | 3.06 | -40 | -12 | 54 |
| 24 |  |  |  |  |  |
|  | R Inferior Temporal Gyrus, posterior division | 3.24 | 56 | -10 | -40 |
| 24 |  |  |  |  |  |
|  | R Brain-Stem | 2.95 | 12 | -42 | -38 |
| 24 |  |  |  |  |  |
|  | L Parahippocampal Gyrus, anterior division | 3.09 | -20 | 0 | -38 |
| 24 |  |  |  |  |  |
|  | L Frontal Pole | 2.94 | -18 | 60 | -20 |
| 24 |  |  |  |  |  |
|  | L Frontal Orbital Cortex | 3.01 | -22 | 22 | -18 |
| 16 |  |  |  |  |  |
|  | R Inferior Temporal Gyrus, posterior division | 2.70 | 44 | -14 | -42 |
| 16 |  |  |  |  |  |
|  | R Subcallosal Cortex | 2.79 | 8 | 26 | -26 |
| 16 |  |  |  |  |  |
|  | L Brain-Stem | 2.73 | -14 | -18 | -22 |
| 16 |  |  |  |  |  |
|  | R Temporal Pole | 2.82 | 36 | 14 | -22 |
| 16 |  |  |  |  |  |
|  | R Frontal Orbital Cortex | 2.69 | 20 | 24 | -22 |
| 16 |  |  |  |  |  |
|  | R Subcallosal Cortex | 2.67 | 12 | 18 | -14 |
| 16 |  |  |  |  |  |
|  | L Pallidum | 3.23 | -20 | -12 | 0 |
| 16 |  |  |  |  |  |
|  | R Insular Cortex | 3.02 | 38 | -16 | 6 |
| 16 |  |  |  |  |  |
|  | R Thalamus | 2.94 | 4 | -20 | 14 |
| 16 |  |  |  |  |  |
|  | R Caudate | 2.49 | 18 | 10 | 14 |
| 16 |  |  |  |  |  |
|  | L Caudate | 3.14 | -16 | 2 | 24 |
| 16 |  |  |  |  |  |
|  | R Lateral Occipital Cortex, superior division | 2.48 | 44 | -56 | 56 |
| 8 |  |  |  |  |  |
|  | L Temporal Pole | 2.51 | -28 | 12 | -48 |
| 8 |  |  |  |  |  |
|  | R Inferior Temporal Gyrus, anterior division | 2.63 | 48 | -6 | -42 |
| 8 |  |  |  |  |  |
|  | R Inferior Temporal Gyrus, posterior division | 2.55 | 52 | -16 | -38 |
| 8 |  |  |  |  |  |
|  | L Frontal Orbital Cortex | 2.76 | -22 | 30 | -20 |
| 8 |  |  |  |  |  |
|  | L Occipital Fusiform Gyrus | 2.58 | -38 | -68 | -18 |
| 8 |  |  |  |  |  |
|  | R Frontal Pole | 2.52 | 24 | 52 | -18 |
| 8 |  |  |  |  |  |
|  | R Frontal Pole | 2.54 | 26 | 56 | -14 |
| 8 |  |  |  |  |  |
|  | R Middle Temporal Gyrus, posterior division | 2.44 | 68 | -24 | -10 |
| 8 |  |  |  |  |  |
|  | L Accumbens | 2.55 | -6 | 14 | -6 |
| 8 |  |  |  |  |  |
|  | L Occipital Pole | 2.53 | -20 | -104 | 12 |
| 8 |  |  |  |  |  |
|  | L Frontal Pole | 2.50 | -40 | 48 | 14 |
| 8 |  |  |  |  |  |
|  | L Caudate | 2.65 | -16 | 12 | 16 |
| 8 |  |  |  |  |  |
|  | L Caudate | 2.44 | -16 | 4 | 18 |
| 8 |  |  |  |  |  |
|  | L Caudate | 2.50 | -12 | -8 | 20 |
| 8 |  |  |  |  |  |
|  | R Caudate | 2.98 | 18 | -2 | 20 |
| 8 |  |  |  |  |  |
|  | L Frontal Pole | 2.50 | -42 | 40 | 20 |
| 8 |  |  |  |  |  |
|  | L Middle Frontal Gyrus | 2.43 | -36 | 6 | 46 |
| 8 |  |  |  |  |  |
|  | L Middle Frontal Gyrus | 2.53 | -32 | 6 | 54 |

# Supplementary Table S10. [US_sustained _> UT_sustained_]. Descriptive statistics for clusters and local maxima showing greater activation for Uncertain-Safety relative to Uncertain-Threat anticipation for the OSP Sustained regressor (FDR *q*<0.05, whole-brain corrected). 

| ***mm***^***3***^ | **Label** | ***t*** | ***x*** | ***y*** | ***z*** |
| --- | --- | --- | --- | --- | --- |
| 382,568 |  |  |  |  |  |
|  | L Frontal Orbital Cortex | 2.57 | -24 | 14 | -18 |
|  | L Temporal Pole | 5.83 | -46 | 12 | -14 |
|  | R Temporal Pole | 5.53 | 30 | 6 | -24 |
|  | L Amygdala | 7.60 | -24 | 0 | -18 |
|  | R Amygdala | 7.55 | 22 | 0 | -18 |
|  | L Temporal Fusiform Cortex, anterior division | 4.66 | -30 | -2 | -36 |
|  | R Middle Temporal Gyrus, anterior division | 8.39 | 60 | -2 | -18 |
|  | L Middle Temporal Gyrus, anterior division | 5.94 | -60 | -4 | -16 |
|  | L Superior Temporal Gyrus, anterior division | 5.71 | -60 | -4 | -4 |
|  | R Planum Polare | 9.04 | 52 | -4 | -2 |
|  | R Temporal Fusiform Cortex, anterior division | 3.94 | 30 | -4 | -38 |
|  | R Superior Temporal Gyrus, anterior division | 6.80 | 62 | -6 | -4 |
|  | L Insular Cortex | 13.64 | -38 | -8 | 6 |
|  | L Planum Polare | 8.77 | -52 | -8 | 2 |
|  | R Insular Cortex | 14.53 | 38 | -8 | 12 |
|  | R Planum Temporale | 11.25 | 64 | -8 | 4 |
|  | R Putamen | 4.60 | 32 | -8 | -8 |
|  | L Juxtapositional Lobule Cortex (formerly Supplementary Motor Cortex) | 7.49 | -10 | -10 | 42 |
|  | L Pallidum | 4.83 | -26 | -10 | -4 |
|  | R Central Opercular Cortex | 14.41 | 38 | -10 | 16 |
|  | R Middle Temporal Gyrus, posterior division | 5.19 | 62 | -10 | -26 |
|  | L Precentral Gyrus | 15.58 | -46 | -12 | 32 |
|  | L Middle Temporal Gyrus, posterior division | 4.05 | -64 | -12 | -18 |
|  | R Hippocampus | 9.61 | 18 | -12 | -22 |
|  | L Central Opercular Cortex | 16.39 | -38 | -14 | 16 |
|  | R Heschls Gyrus (includes H1 and H2) | 10.47 | 54 | -14 | 6 |
|  | R Superior Temporal Gyrus, posterior division | 7.48 | 68 | -18 | 8 |
|  | L Planum Temporale | 10.16 | -62 | -20 | 10 |
|  | L Parahippocampal Gyrus, anterior division | 3.88 | -32 | -22 | -28 |
|  | R Supramarginal Gyrus, anterior division | 4.82 | 62 | -22 | 46 |
|  | L Thalamus | 8.18 | -14 | -24 | -2 |
|  | L Heschls Gyrus (includes H1 and H2) | 8.43 | -48 | -26 | 8 |
|  | L Superior Temporal Gyrus, posterior division | 6.29 | -66 | -26 | 8 |
|  | R Precentral Gyrus | 14.47 | 4 | -26 | 62 |
|  | R Inferior Temporal Gyrus, posterior division | 4.07 | 44 | -26 | -20 |
|  | L Parietal Operculum Cortex | 14.99 | -34 | -28 | 18 |
|  | L Supramarginal Gyrus, anterior division | 3.04 | -56 | -30 | 42 |
|  | R Thalamus | 7.42 | 16 | -30 | -4 |
|  | R Parahippocampal Gyrus, posterior division | 11.26 | 28 | -32 | -18 |
|  | L Temporal Fusiform Cortex, posterior division | 10.45 | -30 | -34 | -18 |
|  | R Postcentral Gyrus | 16.04 | 2 | -34 | 64 |
|  | L Postcentral Gyrus | 17.03 | -2 | -36 | 62 |
|  | R Supramarginal Gyrus, posterior division | 2.89 | 52 | -36 | 52 |
|  | L Parahippocampal Gyrus, posterior division | 10.97 | -26 | -38 | -16 |
|  | R Temporal Fusiform Cortex, posterior division | 10.96 | 24 | -38 | -16 |
|  | L Hippocampus | 12.00 | -10 | -42 | 4 |
|  | L Inferior Temporal Gyrus, posterior division | 3.92 | -64 | -42 | -20 |
|  | R Cingulate Gyrus, posterior division | 13.07 | 10 | -42 | 4 |
|  | R Middle Temporal Gyrus, temporooccipital part | 6.67 | 64 | -42 | -12 |
|  | L Cingulate Gyrus, posterior division | 12.12 | -12 | -48 | 0 |
|  | L Middle Temporal Gyrus, temporooccipital part | 6.75 | -56 | -50 | -10 |
|  | R Lingual Gyrus | 13.66 | 12 | -50 | 2 |
|  | L Superior Parietal Lobule | 3.73 | -34 | -52 | 56 |
|  | L Inferior Temporal Gyrus, temporooccipital part | 6.33 | -46 | -54 | -20 |
|  | R Temporal Occipital Fusiform Cortex | 10.23 | 30 | -54 | -6 |
|  | R Superior Parietal Lobule | 4.26 | 26 | -54 | 62 |
|  | L Temporal Occipital Fusiform Cortex | 8.93 | -28 | -58 | -8 |
|  | L Angular Gyrus | 3.76 | -42 | -58 | 26 |
|  | R Angular Gyrus | 5.80 | 54 | -58 | 16 |
|  | R Inferior Temporal Gyrus, temporooccipital part | 5.22 | 60 | -58 | -12 |
|  | L Lingual Gyrus | 13.60 | -12 | -60 | 4 |
|  | L Precuneus Cortex | 15.60 | -6 | -68 | 20 |
|  | R Precuneus Cortex | 18.89 | 2 | -68 | 22 |
|  | R Supracalcarine Cortex | 18.19 | 2 | -68 | 18 |
|  | R Lateral Occipital Cortex, superior division | 10.57 | 52 | -68 | 34 |
|  | R Occipital Fusiform Gyrus | 7.45 | 26 | -68 | -10 |
|  | R Intracalcarine Cortex | 20.10 | 12 | -70 | 12 |
|  | L Occipital Fusiform Gyrus | 5.48 | -26 | -74 | -10 |
|  | L Lateral Occipital Cortex, superior division | 10.06 | -46 | -76 | 30 |
|  | L Lateral Occipital Cortex, inferior division | 8.31 | -50 | -78 | 4 |
|  | R Lateral Occipital Cortex, inferior division | 7.70 | 44 | -78 | 14 |
|  | L Intracalcarine Cortex | 20.46 | -14 | -80 | 6 |
|  | R Cuneal Cortex | 4.27 | 16 | -80 | 30 |
|  | R Occipital Pole | 5.26 | 24 | -88 | 36 |
|  | L Occipital Pole | 9.29 | -2 | -96 | 16 |
| 33,072 |  |  |  |  |  |
|  | L Frontal Pole | 6.17 | -6 | 72 | 4 |
|  | R Frontal Pole | 11.06 | 2 | 64 | -4 |
|  | R Superior Frontal Gyrus | 3.65 | 6 | 54 | 20 |
|  | L Frontal Medial Cortex | 6.93 | -4 | 52 | -6 |
|  | L Paracingulate Gyrus | 6.15 | -8 | 48 | -4 |
|  | R Frontal Medial Cortex | 8.01 | 6 | 40 | -16 |
|  | L Cingulate Gyrus, anterior division | 4.86 | -2 | 34 | 0 |
|  | R Paracingulate Gyrus | 6.71 | 6 | 34 | -10 |
|  | R Cingulate Gyrus, anterior division | 5.52 | 4 | 32 | 8 |
|  | R Subcallosal Cortex | 6.46 | 4 | 30 | -18 |
|  | L Subcallosal Cortex | 5.41 | -6 | 24 | -16 |
|  | R Caudate | 6.32 | 14 | 24 | -2 |
|  | L Caudate | 4.78 | -12 | 22 | 2 |
|  | R Accumbens | 5.58 | 10 | 20 | -4 |
| 30,608 |  |  |  |  |  |
|  | L Frontal Pole | 7.97 | -50 | 40 | 12 |
|  | L Frontal Orbital Cortex | 8.82 | -34 | 34 | -12 |
|  | L Inferior Frontal Gyrus, pars triangularis | 7.77 | -44 | 32 | 18 |
|  | L Superior Frontal Gyrus | 8.29 | -24 | 24 | 50 |
|  | L Middle Frontal Gyrus | 7.79 | -52 | 24 | 30 |
| 24,696 |  |  |  |  |  |
|  | R Frontal Pole | 10.72 | 32 | 36 | -14 |
|  | R Superior Frontal Gyrus | 11.16 | 24 | 34 | 50 |
|  | R Inferior Frontal Gyrus, pars triangularis | 6.93 | 46 | 34 | 16 |
|  | R Middle Frontal Gyrus | 6.59 | 36 | 20 | 50 |
| 2,480 |  |  |  |  |  |
|  | R Brain-Stem | 4.37 | 10 | -22 | -40 |
|  | L Brain-Stem | 4.44 | -2 | -26 | -44 |
| 656 |  |  |  |  |  |
|  | L Cingulate Gyrus, anterior division | 5.36 | -4 | 0 | 28 |
|  | R Cingulate Gyrus, anterior division | 6.81 | 4 | -4 | 30 |
| 632 |  |  |  |  |  |
|  | R Occipital Pole | 4.27 | 18 | -100 | -8 |
| 288 |  |  |  |  |  |
|  | L Superior Parietal Lobule | 2.63 | -40 | -46 | 48 |
| 232 |  |  |  |  |  |
|  | L Paracingulate Gyrus | 3.45 | -10 | 44 | 18 |
| 224 |  |  |  |  |  |
|  | R Frontal Pole | 2.81 | 16 | 50 | 34 |
| 176 |  |  |  |  |  |
|  | L Occipital Pole | 3.06 | -24 | -100 | -6 |
| 128 |  |  |  |  |  |
|  | R Caudate | 4.47 | 12 | 18 | 12 |
| 112 |  |  |  |  |  |
|  | L Occipital Pole | 2.93 | -12 | -104 | -8 |
| 80 |  |  |  |  |  |
|  | L Temporal Pole | 2.32 | -20 | 8 | -36 |
| 80 |  |  |  |  |  |
|  | R Temporal Pole | 3.36 | 24 | 10 | -36 |
| 80 |  |  |  |  |  |
|  | R Temporal Pole | 2.82 | 46 | 10 | -24 |
| 80 |  |  |  |  |  |
|  | R Paracingulate Gyrus | 2.94 | 2 | 46 | 12 |
| 64 |  |  |  |  |  |
|  | R Frontal Pole | 3.00 | 24 | 54 | 6 |
| 56 |  |  |  |  |  |
|  | L Frontal Pole | 2.82 | -16 | 64 | -18 |
| 48 |  |  |  |  |  |
|  | R Temporal Pole | 2.80 | 30 | 14 | -44 |
| 48 |  |  |  |  |  |
|  | R Precentral Gyrus | 2.96 | 60 | 10 | 34 |
| 40 |  |  |  |  |  |
|  | L Inferior Temporal Gyrus, posterior division | 2.71 | -56 | -20 | -22 |
| 40 |  |  |  |  |  |
|  | R Accumbens | 2.82 | 12 | 14 | -10 |
| 40 |  |  |  |  |  |
|  | L Brain-Stem | 3.21 | -6 | -36 | -4 |
| 40 |  |  |  |  |  |
|  | L Superior Frontal Gyrus | 2.46 | -4 | 56 | 30 |
| 32 |  |  |  |  |  |
|  | R Inferior Temporal Gyrus, temporooccipital part | 2.66 | 60 | -54 | -22 |
| 32 |  |  |  |  |  |
|  | L Frontal Pole | 2.47 | -14 | 52 | -24 |
| 24 |  |  |  |  |  |
|  | R Parahippocampal Gyrus, anterior division | 2.40 | 30 | -16 | -34 |
| 24 |  |  |  |  |  |
|  | L Temporal Pole | 2.30 | -48 | 20 | -28 |
| 24 |  |  |  |  |  |
|  | L Frontal Pole | 2.82 | -10 | 44 | -28 |
| 24 |  |  |  |  |  |
|  | R Brain-Stem | 2.63 | 6 | -36 | -10 |
| 24 |  |  |  |  |  |
|  | R Cingulate Gyrus, anterior division | 3.07 | 4 | -12 | 34 |
| 24 |  |  |  |  |  |
|  | R Postcentral Gyrus | 2.79 | 38 | -30 | 60 |
| 16 |  |  |  |  |  |
|  | R Temporal Pole | 2.24 | 26 | 6 | -42 |
| 16 |  |  |  |  |  |
|  | R Frontal Pole | 2.40 | 6 | 60 | -26 |
| 16 |  |  |  |  |  |
|  | R Lateral Occipital Cortex, inferior division | 2.27 | 32 | -86 | -16 |
| 16 |  |  |  |  |  |
|  | L Insular Cortex | 2.58 | -32 | 6 | 8 |
| 16 |  |  |  |  |  |
|  | R Cingulate Gyrus, anterior division | 2.22 | 8 | 42 | 10 |
| 8 |  |  |  |  |  |
|  | L Brain-Stem | 2.21 | -8 | -44 | -38 |
| 8 |  |  |  |  |  |
|  | R Temporal Pole | 2.11 | 28 | 14 | -32 |
| 8 |  |  |  |  |  |
|  | R Temporal Fusiform Cortex, posterior division | 2.13 | 34 | -18 | -30 |
| 8 |  |  |  |  |  |
|  | L Temporal Pole | 2.49 | -38 | 8 | -28 |
| 8 |  |  |  |  |  |
|  | R Inferior Temporal Gyrus, posterior division | 2.28 | 56 | -24 | -26 |
| 8 |  |  |  |  |  |
|  | R Frontal Orbital Cortex | 2.17 | 12 | 28 | -26 |
| 8 |  |  |  |  |  |
|  | L Frontal Orbital Cortex | 2.24 | -18 | 28 | -22 |
| 8 |  |  |  |  |  |
|  | R Frontal Orbital Cortex | 2.12 | 26 | 12 | -16 |
| 8 |  |  |  |  |  |
|  | L Occipital Pole | 2.27 | -24 | -104 | 0 |
| 8 |  |  |  |  |  |
|  | R Frontal Pole | 2.26 | 40 | 62 | 0 |
| 8 |  |  |  |  |  |
|  | L Frontal Pole | 2.25 | -26 | 54 | 4 |
| 8 |  |  |  |  |  |
|  | L Cingulate Gyrus, anterior division | 2.69 | -4 | 30 | 12 |
| 8 |  |  |  |  |  |
|  | R Paracingulate Gyrus | 2.39 | 14 | 42 | 18 |
| 8 |  |  |  |  |  |
|  | L Cingulate Gyrus, posterior division | 2.14 | -10 | -32 | 38 |
| 8 |  |  |  |  |  |
|  | L Lateral Occipital Cortex, superior division | 2.38 | -14 | -66 | 56 |

# Supplementary Table S11. [CS_sustained _> CT_sustained_]. Descriptive statistics for clusters and local maxima showing greater activation for Certain-Safety relative to Certain-Threat anticipation for the OSP Sustained regressor (FDR *q*<0.05, whole-brain corrected). 

| ***mm***^***3***^ | **Label** | ***t*** | ***x*** | ***y*** | ***z*** |
| --- | --- | --- | --- | --- | --- |
| 273,936 |  |  |  |  |  |
|  | R Frontal Pole | 4.95 | 36 | 34 | -14 |
|  | R Frontal Orbital Cortex | 3.79 | 22 | 28 | -20 |
|  | L Frontal Orbital Cortex | 4.49 | -28 | 12 | -24 |
|  | R Temporal Pole | 4.85 | 34 | 12 | -24 |
|  | L Temporal Pole | 4.74 | -34 | 10 | -24 |
|  | R Planum Polare | 4.06 | 50 | 4 | -8 |
|  | L Amygdala | 4.32 | -24 | 0 | -14 |
|  | R Superior Temporal Gyrus, anterior division | 4.80 | 58 | 0 | -12 |
|  | R Middle Temporal Gyrus, anterior division | 3.68 | 58 | 0 | -26 |
|  | L Amygdala | 4.84 | -26 | -2 | -12 |
|  | L Amygdala | 4.84 | -26 | -2 | -12 |
|  | L Pallidum | 4.34 | -20 | -4 | 0 |
|  | R Temporal Fusiform Cortex, anterior division | 3.62 | 38 | -6 | -38 |
|  | L Middle Temporal Gyrus, anterior division | 5.83 | -60 | -8 | -10 |
|  | R Pallidum | 3.59 | 26 | -8 | 0 |
|  | L Postcentral Gyrus | 6.60 | -54 | -10 | 26 |
|  | L Juxtapositional Lobule Cortex (formerly Supplementary Motor Cortex) | 5.61 | -10 | -10 | 42 |
|  | L Putamen | 5.14 | -28 | -10 | -4 |
|  | L Cingulate Gyrus, anterior division | 5.13 | -6 | -10 | 42 |
|  | R Cingulate Gyrus, anterior division | 5.49 | 10 | -10 | 42 |
|  | R Middle Temporal Gyrus, posterior division | 4.72 | 54 | -10 | -16 |
|  | R Postcentral Gyrus | 6.64 | 50 | -12 | 50 |
|  | R Putamen | 4.09 | 30 | -12 | 4 |
|  | L Central Opercular Cortex | 4.86 | -42 | -14 | 20 |
|  | R Juxtapositional Lobule Cortex (formerly Supplementary Motor Cortex) | 4.30 | 8 | -14 | 48 |
|  | L Thalamus | 4.90 | -4 | -16 | 12 |
|  | L Middle Temporal Gyrus, posterior division | 3.96 | -68 | -16 | -16 |
|  | R Central Opercular Cortex | 6.82 | 38 | -16 | 18 |
|  | R Heschls Gyrus (includes H1 and H2) | 6.81 | 50 | -16 | 8 |
|  | R Inferior Temporal Gyrus, posterior division | 3.22 | 46 | -18 | -32 |
|  | R Parahippocampal Gyrus, anterior division | 6.61 | 20 | -20 | -22 |
|  | L Planum Polare | 6.94 | -40 | -22 | 0 |
|  | L Parahippocampal Gyrus, anterior division | 3.39 | -18 | -22 | -20 |
|  | R Insular Cortex | 8.43 | 36 | -22 | 14 |
|  | R Supramarginal Gyrus, anterior division | 3.42 | 64 | -22 | 20 |
|  | L Insular Cortex | 7.94 | -34 | -24 | 16 |
|  | L Hippocampus | 4.95 | -26 | -24 | -10 |
|  | L Precentral Gyrus | 8.24 | -26 | -26 | 66 |
|  | L Heschls Gyrus (includes H1 and H2) | 8.18 | -34 | -26 | 12 |
|  | R Precentral Gyrus | 7.54 | 6 | -26 | 56 |
|  | R Hippocampus | 6.31 | 24 | -26 | -8 |
|  | R Parietal Operculum Cortex | 2.67 | 54 | -28 | 24 |
|  | L Planum Temporale | 7.73 | -34 | -30 | 16 |
|  | R Thalamus | 5.57 | 18 | -30 | -4 |
|  | R Planum Temporale | 6.64 | 40 | -32 | 16 |
|  | L Parietal Operculum Cortex | 5.00 | -44 | -34 | 22 |
|  | L Brain-Stem | 3.96 | -8 | -34 | -8 |
|  | L Temporal Fusiform Cortex, posterior division | 3.27 | -34 | -34 | -28 |
|  | R Parahippocampal Gyrus, posterior division | 8.64 | 22 | -34 | -18 |
|  | R Temporal Fusiform Cortex, posterior division | 6.67 | 30 | -34 | -18 |
|  | R Brain-Stem | 3.18 | 6 | -34 | -8 |
|  | L Parahippocampal Gyrus, posterior division | 8.86 | -30 | -38 | -12 |
|  | R Lingual Gyrus | 10.41 | 18 | -42 | -10 |
|  | R Cingulate Gyrus, posterior division | 9.33 | 10 | -42 | 4 |
|  | L Lingual Gyrus | 11.68 | -12 | -52 | -2 |
|  | L Cingulate Gyrus, posterior division | 8.05 | -2 | -52 | 22 |
|  | L Angular Gyrus | 3.32 | -56 | -52 | 18 |
|  | R Temporal Occipital Fusiform Cortex | 9.15 | 30 | -52 | -6 |
|  | L Superior Parietal Lobule | 3.20 | -14 | -54 | 68 |
|  | R Angular Gyrus | 4.88 | 48 | -54 | 20 |
|  | L Precuneus Cortex | 8.42 | -18 | -56 | 4 |
|  | R Middle Temporal Gyrus, temporooccipital part | 2.99 | 52 | -58 | 10 |
|  | L Middle Temporal Gyrus, temporooccipital part | 4.05 | -50 | -60 | 0 |
|  | L Inferior Temporal Gyrus, temporooccipital part | 3.75 | -54 | -60 | -14 |
|  | L Temporal Occipital Fusiform Cortex | 5.36 | -34 | -62 | -14 |
|  | R Occipital Fusiform Gyrus | 9.20 | 28 | -66 | -12 |
|  | R Cuneal Cortex | 14.49 | 2 | -70 | 20 |
|  | L Occipital Fusiform Gyrus | 7.22 | -28 | -74 | -8 |
|  | L Lateral Occipital Cortex, inferior division | 4.43 | -52 | -74 | 8 |
|  | R Intracalcarine Cortex | 20.22 | 12 | -74 | 12 |
|  | L Intracalcarine Cortex | 20.48 | -10 | -76 | 10 |
|  | R Lateral Occipital Cortex, superior division | 6.39 | 46 | -76 | 16 |
|  | R Lateral Occipital Cortex, inferior division | 6.28 | 42 | -76 | 10 |
|  | L Lateral Occipital Cortex, superior division | 6.51 | -42 | -78 | 20 |
|  | R Precuneus Cortex | 9.62 | 2 | -78 | 38 |
|  | L Cuneal Cortex | 7.80 | -2 | -86 | 34 |
|  | L Occipital Pole | 10.28 | -2 | -96 | 10 |
|  | R Occipital Pole | 8.67 | 8 | -96 | 18 |
| 23,496 |  |  |  |  |  |
|  | L Frontal Pole | 5.71 | -2 | 66 | 0 |
|  | R Frontal Pole | 7.66 | 0 | 64 | -10 |
|  | R Frontal Medial Cortex | 6.78 | 2 | 50 | -12 |
|  | L Paracingulate Gyrus | 4.68 | -6 | 46 | -6 |
|  | R Paracingulate Gyrus | 5.95 | 2 | 40 | -10 |
|  | L Frontal Medial Cortex | 5.47 | -8 | 36 | -12 |
|  | R Cingulate Gyrus, anterior division | 5.33 | 0 | 36 | 4 |
|  | L Cingulate Gyrus, anterior division | 4.64 | -2 | 32 | -6 |
|  | R Subcallosal Cortex | 4.86 | 2 | 30 | -14 |
|  | L Caudate | 4.18 | -14 | 24 | -2 |
|  | L Subcallosal Cortex | 4.65 | -4 | 20 | -10 |
|  | R Accumbens | 3.77 | 8 | 18 | -6 |
|  | L Accumbens | 4.43 | -6 | 16 | -4 |
| 4,872 |  |  |  |  |  |
|  | R Superior Frontal Gyrus | 5.39 | 22 | 28 | 42 |
|  | R Middle Frontal Gyrus | 6.87 | 26 | 18 | 44 |
| 4,808 |  |  |  |  |  |
|  | L Frontal Pole | 2.56 | -20 | 46 | 44 |
|  | L Superior Frontal Gyrus | 6.24 | -22 | 24 | 40 |
|  | L Middle Frontal Gyrus | 3.36 | -32 | 24 | 50 |
| 1,080 |  |  |  |  |  |
|  | L Frontal Orbital Cortex | 5.18 | -24 | 30 | -20 |
| 928 |  |  |  |  |  |
|  | L Frontal Pole | 2.55 | -48 | 38 | 18 |
|  | L Inferior Frontal Gyrus, pars triangularis | 3.98 | -48 | 30 | 16 |
| 712 |  |  |  |  |  |
|  | R Frontal Pole | 2.80 | 52 | 36 | 12 |
|  | R Inferior Frontal Gyrus, pars triangularis | 2.73 | 48 | 34 | 14 |
|  | R Middle Frontal Gyrus | 3.74 | 46 | 30 | 20 |
| 632 |  |  |  |  |  |
|  | L Brain-Stem | 3.71 | -12 | -28 | -38 |
| 560 |  |  |  |  |  |
|  | R Amygdala | 4.40 | 22 | 2 | -16 |
|  | R Amygdala | 5.40 | 28 | 0 | -12 |
|  | R Amygdala | 3.20 | 18 | 0 | -24 |
| 248 |  |  |  |  |  |
|  | R Subcallosal Cortex | 3.66 | 12 | 18 | -14 |
|  | R Frontal Orbital Cortex | 3.71 | 16 | 12 | -18 |
| 240 |  |  |  |  |  |
|  | R Superior Parietal Lobule | 3.13 | 12 | -54 | 66 |
| 224 |  |  |  |  |  |
|  | L Supramarginal Gyrus, anterior division | 3.11 | -62 | -28 | 32 |
| 208 |  |  |  |  |  |
|  | R Brain-Stem | 4.40 | 0 | -18 | -30 |
|  | L Brain-Stem | 3.25 | -2 | -24 | -40 |
| 168 |  |  |  |  |  |
|  | R Middle Frontal Gyrus | 3.35 | 38 | 10 | 56 |
| 160 |  |  |  |  |  |
|  | R Frontal Pole | 3.13 | 14 | 72 | 14 |
| 144 |  |  |  |  |  |
|  | R Cingulate Gyrus, anterior division | 4.11 | 4 | 10 | 36 |
| 136 |  |  |  |  |  |
|  | R Brain-Stem | 3.14 | 4 | -30 | -40 |
| 128 |  |  |  |  |  |
|  | L Cingulate Gyrus, anterior division | 3.26 | -4 | 0 | 28 |
|  | R Cingulate Gyrus, anterior division | 3.71 | 0 | -2 | 30 |
| 120 |  |  |  |  |  |
|  | L Brain-Stem | 3.34 | -8 | -28 | -18 |
| 120 |  |  |  |  |  |
|  | L Middle Frontal Gyrus | 2.96 | -52 | 16 | 34 |
| 112 |  |  |  |  |  |
|  | R Temporal Pole | 2.79 | 44 | 4 | -44 |
|  | R Inferior Temporal Gyrus, anterior division | 3.09 | 42 | 2 | -42 |
| 104 |  |  |  |  |  |
|  | R Temporal Fusiform Cortex, anterior division | 2.79 | 30 | -6 | -46 |
| 104 |  |  |  |  |  |
|  | R Frontal Orbital Cortex | 3.59 | 14 | 30 | -18 |
|  | R Subcallosal Cortex | 3.24 | 10 | 28 | -20 |
| 96 |  |  |  |  |  |
|  | L Temporal Pole | 4.02 | -28 | 12 | -48 |
| 96 |  |  |  |  |  |
|  | L Temporal Fusiform Cortex, posterior division | 2.56 | -46 | -40 | -24 |
|  | L Inferior Temporal Gyrus, posterior division | 2.56 | -48 | -42 | -22 |
|  | L Inferior Temporal Gyrus, temporooccipital part | 3.01 | -48 | -46 | -18 |
| 96 |  |  |  |  |  |
|  | L Inferior Temporal Gyrus, temporooccipital part | 3.02 | -58 | -46 | -18 |
|  | L Middle Temporal Gyrus, temporooccipital part | 2.52 | -62 | -46 | -12 |
| 96 |  |  |  |  |  |
|  | R Middle Temporal Gyrus, temporooccipital part | 3.19 | 66 | -54 | -2 |
| 96 |  |  |  |  |  |
|  | R Thalamus | 3.39 | 10 | -20 | 18 |
| 88 |  |  |  |  |  |
|  | R Temporal Occipital Fusiform Cortex | 2.91 | 42 | -40 | -26 |
| 88 |  |  |  |  |  |
|  | R Brain-Stem | 3.84 | 10 | -28 | -26 |
| 80 |  |  |  |  |  |
|  | L Thalamus | 3.52 | -10 | -22 | 18 |
| 72 |  |  |  |  |  |
|  | R Brain-Stem | 3.64 | 8 | -44 | -36 |
| 72 |  |  |  |  |  |
|  | L Inferior Temporal Gyrus, posterior division | 3.39 | -64 | -38 | -24 |
| 72 |  |  |  |  |  |
|  | R Middle Temporal Gyrus, temporooccipital part | 3.00 | 54 | -52 | 2 |
| 64 |  |  |  |  |  |
|  | R Inferior Temporal Gyrus, posterior division | 3.09 | 52 | -16 | -38 |
| 64 |  |  |  |  |  |
|  | R Brain-Stem | 2.95 | 14 | -24 | -36 |
| 64 |  |  |  |  |  |
|  | R Brain-Stem | 2.76 | 2 | -16 | -22 |
| 64 |  |  |  |  |  |
|  | R Amygdala | 3.67 | 18 | -6 | -16 |
| 64 |  |  |  |  |  |
|  | L Middle Frontal Gyrus | 3.03 | -52 | 16 | 42 |
| 56 |  |  |  |  |  |
|  | L Parahippocampal Gyrus, anterior division | 3.27 | -22 | -2 | -38 |
| 56 |  |  |  |  |  |
|  | R Brain-Stem | 3.57 | 10 | -14 | -34 |
| 56 |  |  |  |  |  |
|  | L Temporal Pole | 3.22 | -34 | 8 | -36 |
| 56 |  |  |  |  |  |
|  | R Middle Temporal Gyrus, anterior division | 2.52 | 54 | -2 | -28 |
| 56 |  |  |  |  |  |
|  | R Frontal Pole | 3.54 | 12 | 36 | -20 |
| 56 |  |  |  |  |  |
|  | R Middle Frontal Gyrus | 3.07 | 52 | 24 | 36 |
| 48 |  |  |  |  |  |
|  | R Temporal Fusiform Cortex, anterior division | 2.85 | 26 | 0 | -44 |
| 48 |  |  |  |  |  |
|  | L Temporal Pole | 2.71 | -30 | 14 | -38 |
| 48 |  |  |  |  |  |
|  | L Brain-Stem | 3.35 | -4 | -42 | -36 |
| 48 |  |  |  |  |  |
|  | R Temporal Pole | 3.17 | 46 | 14 | -26 |
| 48 |  |  |  |  |  |
|  | R Middle Frontal Gyrus | 2.70 | 42 | 24 | 36 |
| 32 |  |  |  |  |  |
|  | R Brain-Stem | 3.14 | 10 | -24 | -40 |
| 32 |  |  |  |  |  |
|  | L Inferior Temporal Gyrus, posterior division | 2.51 | -52 | -18 | -36 |
| 32 |  |  |  |  |  |
|  | R Temporal Pole | 3.21 | 52 | 20 | -26 |
| 32 |  |  |  |  |  |
|  | R Pallidum | 2.98 | 16 | 2 | 0 |
| 32 |  |  |  |  |  |
|  | R Lateral Occipital Cortex, superior division | 2.44 | 34 | -72 | 50 |
| 24 |  |  |  |  |  |
|  | R Inferior Temporal Gyrus, posterior division | 2.79 | 60 | -14 | -38 |
| 24 |  |  |  |  |  |
|  | R Inferior Temporal Gyrus, posterior division | 2.85 | 58 | -30 | -24 |
| 24 |  |  |  |  |  |
|  | L Amygdala | 2.85 | -14 | -6 | -16 |
| 24 |  |  |  |  |  |
|  | L Middle Temporal Gyrus, posterior division | 2.63 | -62 | -30 | -12 |
| 24 |  |  |  |  |  |
|  | L Temporal Pole | 2.68 | -48 | 10 | -10 |
| 24 |  |  |  |  |  |
|  | L Hippocampus | 2.49 | -28 | -36 | -6 |
| 24 |  |  |  |  |  |
|  | R Pallidum | 2.69 | 22 | -4 | -6 |
| 24 |  |  |  |  |  |
|  | R Caudate | 2.65 | 14 | 16 | 14 |
| 24 |  |  |  |  |  |
|  | R Middle Frontal Gyrus | 2.69 | 42 | 18 | 40 |
| 24 |  |  |  |  |  |
|  | R Superior Frontal Gyrus | 3.09 | 22 | -2 | 54 |
| 16 |  |  |  |  |  |
|  | L Brain-Stem | 3.08 | -10 | -38 | -40 |
| 16 |  |  |  |  |  |
|  | L Temporal Fusiform Cortex, anterior division | 2.39 | -36 | -10 | -36 |
| 16 |  |  |  |  |  |
|  | R Brain-Stem | 2.54 | 12 | -30 | -32 |
| 16 |  |  |  |  |  |
|  | L Inferior Temporal Gyrus, posterior division | 2.54 | -50 | -20 | -30 |
| 16 |  |  |  |  |  |
|  | R Brain-Stem | 2.55 | 12 | -38 | -30 |
| 16 |  |  |  |  |  |
|  | R Brain-Stem | 2.96 | 14 | -30 | -28 |
| 16 |  |  |  |  |  |
|  | R Brain-Stem | 2.61 | 12 | -16 | -28 |
| 16 |  |  |  |  |  |
|  | L Inferior Temporal Gyrus, posterior division | 2.61 | -56 | -44 | -24 |
| 16 |  |  |  |  |  |
|  | L Brain-Stem | 2.67 | -14 | -32 | -26 |
| 16 |  |  |  |  |  |
|  | R Inferior Temporal Gyrus, temporooccipital part | 2.39 | 64 | -50 | -18 |
| 16 |  |  |  |  |  |
|  | L Planum Polare | 2.87 | -40 | -4 | -18 |
| 16 |  |  |  |  |  |
|  | L Frontal Orbital Cortex | 2.70 | -12 | 14 | -18 |
| 16 |  |  |  |  |  |
|  | L Frontal Orbital Cortex | 2.61 | -44 | 32 | -18 |
| 16 |  |  |  |  |  |
|  | L Inferior Temporal Gyrus, temporooccipital part | 2.75 | -52 | -50 | -12 |
| 16 |  |  |  |  |  |
|  | L Inferior Temporal Gyrus, temporooccipital part | 2.50 | -42 | -58 | -6 |
| 16 |  |  |  |  |  |
|  | R Frontal Pole | 2.73 | 24 | 54 | 8 |
| 16 |  |  |  |  |  |
|  | L Frontal Pole | 3.65 | -14 | 62 | 16 |
| 16 |  |  |  |  |  |
|  | L Thalamus | 3.09 | -6 | -14 | 18 |
| 16 |  |  |  |  |  |
|  | L Cingulate Gyrus, anterior division | 2.78 | -2 | 8 | 26 |
| 16 |  |  |  |  |  |
|  | L Frontal Pole | 3.29 | -14 | 52 | 30 |
| 16 |  |  |  |  |  |
|  | R Postcentral Gyrus | 2.37 | 46 | -18 | 64 |
| 8 |  |  |  |  |  |
|  | L Brain-Stem | 2.30 | -4 | -40 | -48 |
| 8 |  |  |  |  |  |
|  | R Inferior Temporal Gyrus, anterior division | 2.31 | 48 | 0 | -46 |
| 8 |  |  |  |  |  |
|  | L Temporal Fusiform Cortex, anterior division | 2.30 | -30 | -6 | -44 |
| 8 |  |  |  |  |  |
|  | L Temporal Pole | 2.38 | -38 | 14 | -40 |
| 8 |  |  |  |  |  |
|  | R Middle Temporal Gyrus, anterior division | 2.62 | 54 | 2 | -36 |
| 8 |  |  |  |  |  |
|  | R Brain-Stem | 2.36 | 8 | -26 | -34 |
| 8 |  |  |  |  |  |
|  | L Brain-Stem | 2.35 | -2 | -26 | -28 |
| 8 |  |  |  |  |  |
|  | R Middle Temporal Gyrus, posterior division | 2.34 | 58 | -12 | -28 |
| 8 |  |  |  |  |  |
|  | L Inferior Temporal Gyrus, posterior division | 2.48 | -56 | -38 | -24 |
| 8 |  |  |  |  |  |
|  | R Parahippocampal Gyrus, anterior division | 2.28 | 16 | -8 | -24 |
| 8 |  |  |  |  |  |
|  | L Brain-Stem | 2.36 | -2 | -22 | -22 |
| 8 |  |  |  |  |  |
|  | R Hippocampus | 2.40 | 12 | -10 | -22 |
| 8 |  |  |  |  |  |
|  | R Temporal Fusiform Cortex, posterior division | 2.29 | 42 | -24 | -20 |
| 8 |  |  |  |  |  |
|  | L Frontal Orbital Cortex | 2.35 | -22 | 16 | -20 |
| 8 |  |  |  |  |  |
|  | R Frontal Pole | 2.39 | 16 | 40 | -20 |
| 8 |  |  |  |  |  |
|  | R Frontal Pole | 2.32 | 48 | 36 | -18 |
| 8 |  |  |  |  |  |
|  | R Inferior Temporal Gyrus, temporooccipital part | 2.48 | 46 | -42 | -16 |
| 8 |  |  |  |  |  |
|  | L Frontal Orbital Cortex | 2.52 | -14 | 20 | -16 |
| 8 |  |  |  |  |  |
|  | R Hippocampus | 2.43 | 34 | -24 | -14 |
| 8 |  |  |  |  |  |
|  | L Amygdala | 2.32 | -18 | -12 | -14 |
| 8 |  |  |  |  |  |
|  | R Amygdala | 2.33 | 22 | -14 | -12 |
| 8 |  |  |  |  |  |
|  | R Middle Temporal Gyrus, temporooccipital part | 2.37 | 58 | -44 | -8 |
| 8 |  |  |  |  |  |
|  | L Middle Temporal Gyrus, posterior division | 2.45 | -54 | -26 | -8 |
| 8 |  |  |  |  |  |
|  | L Frontal Pole | 2.32 | -12 | 64 | -6 |
| 8 |  |  |  |  |  |
|  | R Putamen | 2.30 | 32 | -2 | -4 |
| 8 |  |  |  |  |  |
|  | L Superior Temporal Gyrus, posterior division | 2.40 | -60 | -22 | -2 |
| 8 |  |  |  |  |  |
|  | R Insular Cortex | 2.39 | 34 | 20 | 0 |
| 8 |  |  |  |  |  |
|  | R Caudate | 2.31 | 10 | 20 | 6 |
| 8 |  |  |  |  |  |
|  | L Caudate | 2.85 | -12 | 20 | 8 |
| 8 |  |  |  |  |  |
|  | R Middle Temporal Gyrus, temporooccipital part | 2.29 | 68 | -46 | 10 |
| 8 |  |  |  |  |  |
|  | L Caudate | 2.83 | -8 | 12 | 12 |
| 8 |  |  |  |  |  |
|  | L Cingulate Gyrus, anterior division | 2.59 | -4 | 26 | 16 |
| 8 |  |  |  |  |  |
|  | R Frontal Pole | 2.30 | 16 | 62 | 16 |
| 8 |  |  |  |  |  |
|  | R Precentral Gyrus | 2.31 | 60 | 8 | 22 |
| 8 |  |  |  |  |  |
|  | L Cingulate Gyrus, posterior division | 2.86 | -4 | -24 | 26 |
| 8 |  |  |  |  |  |
|  | L Inferior Frontal Gyrus, pars opercularis | 2.37 | -42 | 14 | 26 |
| 8 |  |  |  |  |  |
|  | L Precentral Gyrus | 2.59 | -40 | -2 | 32 |
| 8 |  |  |  |  |  |
|  | L Cingulate Gyrus, anterior division | 2.81 | -8 | 12 | 32 |
| 8 |  |  |  |  |  |
|  | R Superior Frontal Gyrus | 2.45 | 0 | 48 | 36 |
| 8 |  |  |  |  |  |
|  | R Precuneus Cortex | 2.84 | 12 | -64 | 38 |
| 8 |  |  |  |  |  |
|  | R Frontal Pole | 2.48 | 20 | 36 | 38 |
| 8 |  |  |  |  |  |
|  | R Cingulate Gyrus, anterior division | 2.33 | 4 | 2 | 40 |
| 8 |  |  |  |  |  |
|  | L Frontal Pole | 2.40 | -14 | 50 | 40 |
| 8 |  |  |  |  |  |
|  | R Precuneus Cortex | 2.44 | 14 | -42 | 42 |
| 8 |  |  |  |  |  |
|  | L Frontal Pole | 2.28 | -12 | 46 | 42 |
| 8 |  |  |  |  |  |
|  | L Postcentral Gyrus | 2.56 | -42 | -28 | 52 |
| 8 |  |  |  |  |  |
|  | L Lateral Occipital Cortex, superior division | 2.42 | -16 | -76 | 54 |

# Supplementary Table S12. [CT_sustained _> UT_sustained_]. Descriptive statistics for clusters and local maxima showing greater activation for Certain-Threat relative to Uncertain-Threat anticipation for the OSP Sustained regressor (FDR *q*<0.05, whole-brain corrected). 

| ***mm***^***3***^ | **Label** | ***t*** | ***x*** | ***y*** | ***z*** |
| --- | --- | --- | --- | --- | --- |
| 384,256 |  |  |  |  |  |
|  | L Frontal Pole | 3.75 | -50 | 40 | 10 |
|  | L Inferior Frontal Gyrus, pars triangularis | 4.77 | -44 | 32 | 18 |
|  | L Middle Frontal Gyrus | 5.83 | -40 | 24 | 24 |
|  | L Temporal Pole | 4.36 | -58 | 8 | -22 |
|  | R Temporal Pole | 4.53 | 52 | 8 | -10 |
|  | R Amygdala | 3.94 | 28 | 4 | -26 |
|  | L Amygdala | 4.85 | -32 | 2 | -18 |
|  | R Superior Temporal Gyrus, anterior division | 6.25 | 60 | 2 | -10 |
|  | L Amygdala | 4.48 | -24 | 0 | -20 |
|  | L Temporal Fusiform Cortex, anterior division | 3.86 | -30 | 0 | -36 |
|  | R Middle Temporal Gyrus, anterior division | 8.28 | 58 | 0 | -20 |
|  | R Amygdala | 5.10 | 26 | -2 | -18 |
|  | L Amygdala | 4.22 | -22 | -2 | -18 |
|  | R Planum Polare | 4.76 | 50 | -2 | -6 |
|  | R Amygdala | 4.65 | 18 | -4 | -18 |
|  | R Amygdala | 4.62 | 14 | -4 | -16 |
|  | L Middle Temporal Gyrus, anterior division | 5.86 | -58 | -4 | -14 |
|  | L Superior Temporal Gyrus, anterior division | 3.75 | -52 | -4 | -10 |
|  | R Precentral Gyrus | 12.47 | 52 | -6 | 28 |
|  | L Amygdala | 6.50 | -16 | -8 | -20 |
|  | L Amygdala | 4.18 | -22 | -8 | -22 |
|  | R Postcentral Gyrus | 13.05 | 66 | -8 | 32 |
|  | R Insular Cortex | 11.89 | 38 | -8 | 12 |
|  | L Insular Cortex | 13.21 | -36 | -10 | 14 |
|  | R Central Opercular Cortex | 7.75 | 44 | -10 | 16 |
|  | R Planum Temporale | 6.46 | 66 | -10 | 6 |
|  | L Central Opercular Cortex | 13.50 | -38 | -12 | 16 |
|  | L Precentral Gyrus | 12.10 | -48 | -12 | 36 |
|  | L Parahippocampal Gyrus, anterior division | 5.92 | -18 | -12 | -26 |
|  | L Planum Polare | 4.41 | -48 | -12 | -2 |
|  | R Middle Temporal Gyrus, posterior division | 5.93 | 60 | -12 | -8 |
|  | L Putamen | 4.82 | -32 | -14 | -8 |
|  | L Hippocampus | 8.19 | -22 | -20 | -18 |
|  | L Middle Temporal Gyrus, posterior division | 4.51 | -62 | -20 | -12 |
|  | R Hippocampus | 9.41 | 22 | -20 | -18 |
|  | R Superior Temporal Gyrus, posterior division | 6.71 | 66 | -20 | 4 |
|  | R Parahippocampal Gyrus, anterior division | 4.28 | 28 | -20 | -28 |
|  | R Parietal Operculum Cortex | 6.59 | 36 | -22 | 18 |
|  | L Thalamus | 7.30 | -16 | -24 | -2 |
|  | R Supramarginal Gyrus, anterior division | 6.81 | 60 | -24 | 44 |
|  | R Temporal Fusiform Cortex, posterior division | 6.62 | 34 | -24 | -22 |
|  | R Heschls Gyrus (includes H1 and H2) | 4.40 | 48 | -24 | 8 |
|  | L Heschls Gyrus (includes H1 and H2) | 5.86 | -48 | -26 | 6 |
|  | R Inferior Temporal Gyrus, posterior division | 3.28 | 60 | -26 | -22 |
|  | R Parahippocampal Gyrus, posterior division | 7.90 | 28 | -28 | -22 |
|  | L Parietal Operculum Cortex | 9.43 | -36 | -30 | 18 |
|  | L Superior Temporal Gyrus, posterior division | 6.67 | -68 | -30 | 6 |
|  | L Inferior Temporal Gyrus, posterior division | 5.43 | -46 | -30 | -28 |
|  | L Supramarginal Gyrus, anterior division | 4.53 | -56 | -30 | 42 |
|  | L Temporal Fusiform Cortex, posterior division | 9.15 | -30 | -34 | -18 |
|  | L Brain-Stem | 2.56 | -20 | -34 | -32 |
|  | R Thalamus | 6.75 | 14 | -34 | 4 |
|  | L Postcentral Gyrus | 13.03 | -2 | -36 | 62 |
|  | L Planum Temporale | 8.69 | -62 | -36 | 16 |
|  | L Parahippocampal Gyrus, posterior division | 10.23 | -24 | -38 | -16 |
|  | R Lingual Gyrus | 12.21 | 32 | -40 | -10 |
|  | R Supramarginal Gyrus, posterior division | 5.20 | 38 | -40 | 44 |
|  | L Supramarginal Gyrus, posterior division | 4.48 | -44 | -42 | 46 |
|  | L Cingulate Gyrus, posterior division | 8.70 | -8 | -44 | 4 |
|  | R Superior Parietal Lobule | 4.42 | 38 | -44 | 54 |
|  | L Lingual Gyrus | 11.44 | -28 | -46 | -8 |
|  | R Cingulate Gyrus, posterior division | 9.97 | 2 | -48 | 18 |
|  | L Superior Parietal Lobule | 8.23 | -34 | -52 | 58 |
|  | L Inferior Temporal Gyrus, temporooccipital part | 4.48 | -48 | -54 | -20 |
|  | L Middle Temporal Gyrus, temporooccipital part | 7.28 | -60 | -56 | -10 |
|  | L Temporal Occipital Fusiform Cortex | 3.43 | -42 | -56 | -14 |
|  | L Angular Gyrus | 5.23 | -44 | -58 | 22 |
|  | R Angular Gyrus | 2.55 | 46 | -58 | 44 |
|  | R Intracalcarine Cortex | 10.36 | 24 | -60 | 6 |
|  | L Intracalcarine Cortex | 10.72 | -14 | -64 | 10 |
|  | R Precuneus Cortex | 15.56 | 10 | -64 | 18 |
|  | L Lateral Occipital Cortex, inferior division | 8.51 | -56 | -66 | -8 |
|  | L Precuneus Cortex | 12.26 | -6 | -68 | 20 |
|  | R Cuneal Cortex | 13.46 | 18 | -68 | 22 |
|  | R Supracalcarine Cortex | 8.90 | 0 | -78 | 14 |
|  | L Lateral Occipital Cortex, superior division | 11.21 | -24 | -82 | 32 |
|  | L Cuneal Cortex | 8.78 | -4 | -82 | 30 |
|  | R Lateral Occipital Cortex, superior division | 12.64 | 38 | -82 | 32 |
|  | L Supracalcarine Cortex | 7.68 | -2 | -84 | 10 |
|  | R Occipital Pole | 8.24 | 14 | -88 | 34 |
|  | L Occipital Pole | 7.18 | -2 | -90 | 24 |
| 66,152 |  |  |  |  |  |
|  | R Frontal Pole | 8.59 | 2 | 58 | -8 |
|  | R Paracingulate Gyrus | 4.67 | 6 | 54 | 18 |
|  | R Frontal Medial Cortex | 8.08 | 6 | 50 | -10 |
|  | L Frontal Medial Cortex | 5.84 | -2 | 48 | -16 |
|  | L Paracingulate Gyrus | 5.69 | -8 | 46 | -8 |
|  | L Frontal Pole | 5.91 | -18 | 40 | 50 |
|  | L Cingulate Gyrus, anterior division | 3.69 | -2 | 36 | -6 |
|  | R Superior Frontal Gyrus | 10.97 | 26 | 32 | 50 |
|  | R Cingulate Gyrus, anterior division | 3.47 | 6 | 32 | 10 |
|  | L Superior Frontal Gyrus | 8.16 | -24 | 24 | 46 |
|  | L Subcallosal Cortex | 5.83 | -4 | 24 | -18 |
|  | R Caudate | 3.88 | 14 | 24 | -2 |
|  | R Middle Frontal Gyrus | 8.25 | 28 | 20 | 54 |
|  | R Accumbens | 2.57 | 12 | 16 | -6 |
|  | R Subcallosal Cortex | 4.85 | 2 | 14 | -6 |
|  | L Middle Frontal Gyrus | 2.44 | -32 | 8 | 64 |
| 10,376 |  |  |  |  |  |
|  | L Lingual Gyrus | 2.75 | -8 | -86 | -16 |
| 2,912 |  |  |  |  |  |
|  | R Inferior Temporal Gyrus, posterior division | 3.09 | 58 | -34 | -16 |
|  | R Middle Temporal Gyrus, temporooccipital part | 4.67 | 58 | -50 | -10 |
|  | R Inferior Temporal Gyrus, temporooccipital part | 5.04 | 60 | -58 | -12 |
|  | R Lateral Occipital Cortex, inferior division | 3.45 | 58 | -60 | -4 |
| 2,896 |  |  |  |  |  |
|  | L Frontal Pole | 4.05 | -48 | 46 | -12 |
|  | L Frontal Orbital Cortex | 6.69 | -34 | 36 | -12 |
| 456 |  |  |  |  |  |
|  | L Juxtapositional Lobule Cortex (formerly Supplementary Motor Cortex) | 6.39 | -4 | 0 | 64 |
| 376 |  |  |  |  |  |
|  | R Temporal Fusiform Cortex, anterior division | 3.19 | 32 | -4 | -40 |
|  | R Parahippocampal Gyrus, anterior division | 2.88 | 26 | -6 | -36 |
| 360 |  |  |  |  |  |
|  | R Temporal Pole | 3.67 | 24 | 16 | -44 |
| 296 |  |  |  |  |  |
|  | L Brain-Stem | 2.47 | -2 | -36 | -6 |
|  | R Brain-Stem | 3.91 | 6 | -38 | -12 |
| 208 |  |  |  |  |  |
|  | L Cingulate Gyrus, anterior division | 3.62 | -4 | -2 | 30 |
|  | R Cingulate Gyrus, anterior division | 4.24 | 4 | -4 | 32 |
| 184 |  |  |  |  |  |
|  | R Temporal Fusiform Cortex, posterior division | 3.72 | 40 | -22 | -32 |
| 168 |  |  |  |  |  |
|  | L Brain-Stem | 3.21 | -6 | -26 | -44 |
| 160 |  |  |  |  |  |
|  | L Caudate | 3.24 | -18 | 18 | 12 |
| 96 |  |  |  |  |  |
|  | L Temporal Pole | 2.35 | -32 | 6 | -28 |
| 96 |  |  |  |  |  |
|  | L Middle Frontal Gyrus | 2.93 | -40 | 14 | 46 |
| 80 |  |  |  |  |  |
|  | L Middle Temporal Gyrus, temporooccipital part | 2.70 | -48 | -52 | 6 |
| 72 |  |  |  |  |  |
|  | R Brain-Stem | 3.06 | 0 | -18 | -40 |
| 64 |  |  |  |  |  |
|  | L Temporal Pole | 2.72 | -44 | 22 | -32 |
| 64 |  |  |  |  |  |
|  | L Inferior Temporal Gyrus, posterior division | 2.48 | -60 | -24 | -26 |
| 56 |  |  |  |  |  |
|  | L Brain-Stem | 3.20 | -12 | -24 | -32 |
| 48 |  |  |  |  |  |
|  | R Inferior Temporal Gyrus, temporooccipital part | 2.38 | 58 | -56 | -22 |
| 48 |  |  |  |  |  |
|  | L Frontal Orbital Cortex | 2.56 | -34 | 30 | -22 |
| 48 |  |  |  |  |  |
|  | L Frontal Pole | 2.44 | -10 | 54 | -24 |
| 40 |  |  |  |  |  |
|  | L Temporal Pole | 2.85 | -28 | 18 | -40 |
| 40 |  |  |  |  |  |
|  | R Brain-Stem | 2.82 | 0 | -20 | -24 |
| 40 |  |  |  |  |  |
|  | L Frontal Pole | 2.97 | -12 | 64 | -18 |
| 32 |  |  |  |  |  |
|  | R Temporal Pole | 2.45 | 44 | 14 | -20 |
| 32 |  |  |  |  |  |
|  | R Paracingulate Gyrus | 2.81 | 12 | 44 | 18 |
| 32 |  |  |  |  |  |
|  | R Caudate | 2.76 | 16 | 2 | 22 |
| 24 |  |  |  |  |  |
|  | R Temporal Fusiform Cortex, posterior division | 2.24 | 36 | -16 | -28 |
| 24 | 24 |  |  |  |  |
|  | L Temporal Pole | 2.51 | -48 | 12 | -28 |
| 24 | 24 |  |  |  |  |
|  | L Caudate | 2.27 | -12 | 20 | -4 |
| 24 | 24 |  |  |  |  |
|  | L Frontal Pole | 2.59 | -36 | 48 | 4 |
| 24 | 24 |  |  |  |  |
|  | R Angular Gyrus | 2.32 | 42 | -52 | 48 |
| 16 | 16 |  |  |  |  |
|  | L Temporal Fusiform Cortex, posterior division | 2.21 | -36 | -14 | -38 |
| 16 | 16 |  |  |  |  |
|  | R Temporal Pole | 2.20 | 24 | 4 | -40 |
| 16 | 16 |  |  |  |  |
|  | L Brain-Stem | 2.84 | -6 | -14 | -38 |
| 16 |  |  |  |  |  |
|  | L Brain-Stem | 2.45 | -10 | -32 | -36 |
| 16 |  |  |  |  |  |
|  | R Brain-Stem | 2.63 | 4 | -18 | -32 |
| 16 |  |  |  |  |  |
|  | R Temporal Pole | 2.26 | 24 | 10 | -32 |
| 16 |  |  |  |  |  |
|  | R Brain-Stem | 3.11 | 6 | -42 | -28 |
| 16 |  |  |  |  |  |
|  | L Brain-Stem | 2.20 | -8 | -28 | -28 |
| 16 |  |  |  |  |  |
|  | R Brain-Stem | 2.44 | 2 | -24 | -26 |
| 16 |  |  |  |  |  |
|  | R Parahippocampal Gyrus, anterior division | 2.34 | 20 | 2 | -28 |
| 16 |  |  |  |  |  |
|  | L Temporal Pole | 2.72 | -38 | 10 | -28 |
| 16 |  |  |  |  |  |
|  | R Frontal Orbital Cortex | 2.95 | 10 | 18 | -26 |
| 16 |  |  |  |  |  |
|  | L Temporal Occipital Fusiform Cortex | 2.37 | -42 | -48 | -24 |
| 16 |  |  |  |  |  |
|  | L Frontal Medial Cortex | 2.48 | -6 | 48 | -24 |
| 16 |  |  |  |  |  |
|  | R Frontal Orbital Cortex | 2.39 | 26 | 12 | -18 |
| 16 |  |  |  |  |  |
|  | R Frontal Pole | 2.79 | 38 | 60 | -10 |
| 16 |  |  |  |  |  |
|  | L Insular Cortex | 3.01 | -32 | 6 | 8 |
| 16 |  |  |  |  |  |
|  | L Frontal Pole | 3.17 | -18 | 48 | 30 |
| 8 |  |  |  |  |  |
|  | R Brain-Stem | 2.23 | 10 | -20 | -44 |
| 8 |  |  |  |  |  |
|  | R Brain-Stem | 2.31 | 6 | -44 | -42 |
| 8 |  |  |  |  |  |
|  | R Brain-Stem | 2.23 | 8 | -26 | -42 |
| 8 |  |  |  |  |  |
|  | L Temporal Fusiform Cortex, posterior division | 2.37 | -28 | -12 | -42 |
| 8 |  |  |  |  |  |
|  | L Brain-Stem | 2.49 | -10 | -18 | -40 |
| 8 |  |  |  |  |  |
|  | R Brain-Stem | 2.16 | 12 | -26 | -38 |
| 8 |  |  |  |  |  |
|  | L Temporal Pole | 2.24 | -20 | 8 | -36 |
| 8 |  |  |  |  |  |
|  | R Temporal Pole | 2.24 | 24 | 20 | -36 |
| 8 |  |  |  |  |  |
|  | L Temporal Pole | 2.31 | -42 | 14 | -34 |
| 8 |  |  |  |  |  |
|  | L Temporal Fusiform Cortex, posterior division | 2.35 | -40 | -30 | -30 |
| 8 |  |  |  |  |  |
|  | R Inferior Temporal Gyrus, posterior division | 2.16 | 54 | -24 | -26 |
| 8 |  |  |  |  |  |
|  | R Inferior Temporal Gyrus, posterior division | 2.18 | 52 | -38 | -18 |
| 8 |  |  |  |  |  |
|  | L Middle Temporal Gyrus, posterior division | 2.21 | -64 | -30 | -18 |
| 8 | 8 |  |  |  |  |
|  | L Middle Temporal Gyrus, posterior division | 2.13 | -66 | -28 | -16 |
| 8 | 8 |  |  |  |  |
|  | R Frontal Orbital Cortex | 2.28 | 22 | 14 | -16 |
| 8 | 8 |  |  |  |  |
|  | L Frontal Pole | 2.68 | -36 | 58 | -16 |
| 8 | 8 |  |  |  |  |
|  | R Frontal Pole | 2.20 | 26 | 48 | -12 |
| 8 | 8 |  |  |  |  |
|  | L Frontal Pole | 2.13 | -36 | 54 | -10 |
| 8 | 8 |  |  |  |  |
|  | R Frontal Pole | 2.13 | 14 | 62 | -10 |
| 8 | 8 |  |  |  |  |
|  | L Middle Temporal Gyrus, posterior division | 2.18 | -68 | -24 | -8 |
| 8 | 8 |  |  |  |  |
|  | R Putamen | 2.21 | 32 | -14 | -8 |
| 8 | 8 |  |  |  |  |
|  | L Frontal Pole | 2.68 | -18 | 60 | -8 |
| 8 | 8 |  |  |  |  |
|  | R Cingulate Gyrus, anterior division | 2.15 | 2 | 32 | -6 |
| 8 | 8 |  |  |  |  |
|  | L Brain-Stem | 2.20 | -6 | -36 | -4 |
| 8 | 8 |  |  |  |  |
|  | L Cingulate Gyrus, anterior division | 2.21 | -8 | 36 | -4 |
| 8 | 8 |  |  |  |  |
|  | L Caudate | 2.57 | -6 | 16 | 2 |
| 8 | 8 |  |  |  |  |
|  | R Heschls Gyrus (includes H1 and H2) | 2.30 | 44 | -20 | 8 |
| 8 | 8 |  |  |  |  |
|  | L Thalamus | 2.20 | -4 | -28 | 10 |
| 8 | 8 |  |  |  |  |
|  | R Middle Frontal Gyrus | 2.14 | 42 | 28 | 36 |
| 8 | 8 |  |  |  |  |
|  | L Frontal Pole | 2.39 | -4 | 44 | 52 |

# Supplementary Table S13. [CT_phasic _> CS_phasic_]. Descriptive statistics for clusters and local maxima showing greater activation for Certain-Threat relative to Certain-Safety anticipation for the OSP Phasic regressor (FDR *q*<0.05, whole-brain corrected). 

| ***mm***^***3***^ | **Label** | ***t*** | ***x*** | ***y*** | ***z*** |
| --- | --- | --- | --- | --- | --- |
| 80,016 |  |  |  |  |  |
|  | R Insular Cortex | 7.02 | 34 | 24 | 2 |
|  | L Caudate | 6.60 | -14 | 22 | -2 |
|  | L Frontal Orbital Cortex | 6.44 | -30 | 22 | -8 |
|  | L Inferior Frontal Gyrus, pars triangularis | 3.92 | -50 | 22 | 12 |
|  | R Caudate | 7.34 | 14 | 20 | -2 |
|  | R Frontal Operculum Cortex | 8.62 | 36 | 18 | 8 |
|  | R Frontal Orbital Cortex | 4.93 | 44 | 18 | -8 |
|  | L Frontal Operculum Cortex | 8.12 | -42 | 16 | -2 |
|  | L Insular Cortex | 7.89 | -36 | 14 | 4 |
|  | R Inferior Frontal Gyrus, pars opercularis | 5.09 | 60 | 14 | 8 |
|  | R Accumbens | 4.38 | 6 | 14 | -2 |
|  | L Inferior Frontal Gyrus, pars opercularis | 6.44 | -58 | 12 | 4 |
|  | L Accumbens | 6.19 | -6 | 10 | -4 |
|  | L Putamen | 6.01 | -20 | 8 | 0 |
|  | R Central Opercular Cortex | 7.32 | 48 | 8 | -2 |
|  | R Temporal Pole | 3.37 | 38 | 8 | -22 |
|  | L Temporal Pole | 3.88 | -30 | 6 | -20 |
|  | R Putamen | 8.94 | 22 | 4 | -10 |
|  | R Precentral Gyrus | 4.96 | 60 | 2 | 12 |
|  | L Central Opercular Cortex | 5.87 | -50 | 0 | 2 |
|  | L Amygdala | 3.83 | -32 | -2 | -18 |
|  | L Pallidum | 5.58 | -20 | -2 | 0 |
|  | R Thalamus | 4.32 | 0 | -8 | 2 |
|  | L Planum Polare | 4.80 | -42 | -10 | -8 |
|  | L Postcentral Gyrus | 4.73 | -46 | -10 | 30 |
|  | L Precentral Gyrus | 5.94 | -36 | -16 | 50 |
|  | R Planum Polare | 2.95 | 40 | -16 | -4 |
|  | L Thalamus | 7.43 | -4 | -26 | 0 |
|  | R Thalamus | 6.28 | 8 | -26 | 4 |
|  | L Brain-Stem | 7.20 | -6 | -34 | -8 |
|  | L Parahippocampal Gyrus, posterior division | 2.97 | -14 | -34 | -10 |
|  | R Brain-Stem | 5.44 | 8 | -34 | -8 |
|  | L Temporal Occipital Fusiform Cortex | 3.75 | -34 | -54 | -22 |
|  | R Temporal Occipital Fusiform Cortex | 3.48 | 36 | -60 | -20 |
|  | L Occipital Fusiform Gyrus | 3.95 | -24 | -68 | -16 |
|  | R Occipital Fusiform Gyrus | 3.33 | 26 | -70 | -16 |
|  | L Lingual Gyrus | 3.62 | -10 | -76 | -14 |
| 21,328 |  |  |  |  |  |
|  | L Paracingulate Gyrus | 4.46 | -6 | 36 | 22 |
|  | L Cingulate Gyrus, anterior division | 5.79 | -8 | 14 | 36 |
|  | R Superior Frontal Gyrus | 3.60 | 4 | 14 | 60 |
|  | R Paracingulate Gyrus | 6.22 | 0 | 12 | 46 |
|  | R Cingulate Gyrus, anterior division | 7.24 | 10 | 8 | 40 |
|  | L Juxtapositional Lobule Cortex (formerly Supplementary Motor Cortex) | 6.32 | -10 | 2 | 42 |
|  | R Juxtapositional Lobule Cortex (formerly Supplementary Motor Cortex) | 6.89 | 4 | -2 | 50 |
|  | L Superior Frontal Gyrus | 5.59 | -12 | -8 | 66 |
|  | L Precentral Gyrus | 3.86 | -14 | -20 | 72 |
|  | R Precentral Gyrus | 4.22 | 8 | -28 | 50 |
|  | R Cingulate Gyrus, posterior division | 4.64 | 10 | -32 | 46 |
| 10,120 |  |  |  |  |  |
|  | R Supramarginal Gyrus, anterior division | 4.92 | 62 | -28 | 26 |
|  | R Parietal Operculum Cortex | 5.58 | 52 | -32 | 28 |
|  | R Angular Gyrus | 5.48 | 56 | -46 | 32 |
|  | R Supramarginal Gyrus, posterior division | 5.39 | 58 | -46 | 38 |
| 7,712 |  |  |  |  |  |
|  | L Postcentral Gyrus | 3.80 | -60 | -20 | 20 |
|  | L Planum Temporale | 3.52 | -60 | -30 | 10 |
|  | L Parietal Operculum Cortex | 7.12 | -44 | -34 | 22 |
|  | L Supramarginal Gyrus, anterior division | 5.69 | -64 | -36 | 26 |
|  | L Supramarginal Gyrus, posterior division | 4.89 | -64 | -48 | 34 |
|  | L Angular Gyrus | 3.35 | -58 | -54 | 34 |
| 7,120 |  |  |  |  |  |
|  | R Middle Frontal Gyrus | 6.09 | 46 | 2 | 56 |
|  | R Precentral Gyrus | 6.24 | 36 | -18 | 46 |
|  | R Postcentral Gyrus | 5.19 | 30 | -28 | 60 |
| 4,624 |  |  |  |  |  |
|  | R Frontal Pole | 5.94 | 26 | 46 | 26 |
| 4,320 |  |  |  |  |  |
|  | R Lateral Occipital Cortex, inferior division | 4.62 | 38 | -78 | -12 |
|  | R Occipital Fusiform Gyrus | 3.35 | 32 | -80 | -10 |
|  | R Lateral Occipital Cortex, superior division | 3.37 | 32 | -88 | 16 |
|  | R Occipital Pole | 4.45 | 36 | -90 | 8 |
| 4,280 |  |  |  |  |  |
|  | L Frontal Pole | 6.22 | -36 | 48 | 30 |
|  | L Middle Frontal Gyrus | 4.18 | -32 | 30 | 42 |
| 3,128 |  |  |  |  |  |
|  | R Lingual Gyrus | 3.70 | 26 | -58 | 4 |
|  | R Intracalcarine Cortex | 5.98 | 12 | -72 | 14 |
|  | R Cuneal Cortex | 3.84 | 4 | -80 | 20 |
| 2,272 |  |  |  |  |  |
|  | L Lingual Gyrus | 3.40 | -10 | -62 | 2 |
|  | L Intracalcarine Cortex | 6.45 | -14 | -72 | 8 |
| 1,768 |  |  |  |  |  |
|  | R Superior Temporal Gyrus, posterior division | 5.01 | 62 | -22 | -2 |
|  | R Middle Temporal Gyrus, posterior division | 4.72 | 56 | -28 | -4 |
| 1,528 |  |  |  |  |  |
|  | R Superior Frontal Gyrus | 4.66 | 14 | -10 | 68 |
|  | R Precentral Gyrus | 3.30 | 14 | -16 | 72 |
| 1,264 |  |  |  |  |  |
|  | L Precuneus Cortex | 5.77 | -6 | -72 | 46 |
|  | L Cuneal Cortex | 3.23 | -18 | -72 | 26 |
| 1,032 |  |  |  |  |  |
|  | R Precuneus Cortex | 4.78 | 18 | -60 | 28 |
|  | R Lateral Occipital Cortex, superior division | 3.36 | 20 | -70 | 48 |
| 920 |  |  |  |  |  |
|  | L Precentral Gyrus | 4.94 | -14 | -30 | 62 |
| 816 |  |  |  |  |  |
|  | L Occipital Fusiform Gyrus | 3.73 | -20 | -86 | -8 |
|  | L Lateral Occipital Cortex, inferior division | 2.96 | -36 | -86 | -14 |
|  | L Occipital Pole | 3.99 | -32 | -92 | -14 |
| 728 |  |  |  |  |  |
|  | L Precentral Gyrus | 3.69 | -10 | -22 | 46 |
|  | L Cingulate Gyrus, posterior division | 4.55 | -10 | -26 | 40 |
| 504 |  |  |  |  |  |
|  | L Middle Temporal Gyrus, temporooccipital part | 4.56 | -58 | -60 | 10 |
| 440 |  |  |  |  |  |
|  | R Lateral Occipital Cortex, superior division | 4.42 | 22 | -86 | 30 |
|  | R Occipital Pole | 3.49 | 12 | -90 | 26 |
| 360 |  |  |  |  |  |
|  | L Precuneus Cortex | 3.65 | -6 | -50 | 48 |
| 344 |  |  |  |  |  |
|  | L Thalamus | 4.08 | -16 | -26 | 8 |
| 328 |  |  |  |  |  |
|  | L Supramarginal Gyrus, posterior division | 3.47 | -52 | -50 | 14 |
|  | L Angular Gyrus | 3.69 | -56 | -52 | 18 |
| 320 |  |  |  |  |  |
|  | R Parahippocampal Gyrus, anterior division | 4.16 | 24 | -8 | -32 |
|  | R Temporal Fusiform Cortex, posterior division | 4.20 | 34 | -14 | -32 |
| 304 |  |  |  |  |  |
|  | R Temporal Pole | 3.95 | 46 | 8 | -40 |
| 280 |  |  |  |  |  |
|  | L Lateral Occipital Cortex, superior division | 2.93 | -26 | -88 | 18 |
|  | L Occipital Pole | 3.26 | -30 | -92 | 18 |
| 232 |  |  |  |  |  |
|  | R Insular Cortex | 4.18 | 36 | -20 | 2 |
| 216 |  |  |  |  |  |
|  | L Insular Cortex | 3.84 | -40 | -18 | -2 |
|  | L Putamen | 3.32 | -30 | -18 | -2 |
| 208 |  |  |  |  |  |
|  | R Hippocampus | 4.10 | 26 | -40 | 2 |
| 208 |  |  |  |  |  |
|  | L Precentral Gyrus | 3.53 | -22 | -14 | 58 |
| 200 |  |  |  |  |  |
|  | L Thalamus | 5.19 | -22 | -36 | 0 |
| 200 |  |  |  |  |  |
|  | L Frontal Pole | 3.99 | -34 | 42 | 18 |
| 192 |  |  |  |  |  |
|  | R Cingulate Gyrus, anterior division | 3.74 | 0 | 34 | 4 |
|  | L Subcallosal Cortex | 4.13 | -2 | 28 | -2 |
| 176 |  |  |  |  |  |
|  | R Precentral Gyrus | 4.40 | 32 | -14 | 64 |
| 168 |  |  |  |  |  |
|  | R Frontal Medial Cortex | 3.48 | 2 | 36 | -28 |
| 168 |  |  |  |  |  |
|  | L Lateral Occipital Cortex, inferior division | 3.32 | -30 | -90 | -4 |
|  | L Occipital Pole | 3.75 | -34 | -92 | -6 |
| 168 |  |  |  |  |  |
|  | L Caudate | 3.41 | -12 | 4 | 12 |
| 160 |  |  |  |  |  |
|  | L Middle Temporal Gyrus, posterior division | 3.18 | -60 | -22 | -14 |
| 160 |  |  |  |  |  |
|  | L Supramarginal Gyrus, posterior division | 3.34 | -56 | -48 | 50 |
| 144 |  |  |  |  |  |
|  | L Frontal Pole | 4.15 | -26 | 64 | -12 |
| 144 |  |  |  |  |  |
|  | R Middle Temporal Gyrus, temporooccipital part | 3.63 | 68 | -40 | 4 |
| 144 |  |  |  |  |  |
|  | R Superior Parietal Lobule | 3.46 | 24 | -42 | 60 |
| 136 |  |  |  |  |  |
|  | L Hippocampus | 3.76 | -26 | -34 | -8 |
| 136 |  |  |  |  |  |
|  | L Occipital Pole | 2.78 | -32 | -96 | 2 |
| 136 |  |  |  |  |  |
|  | L Frontal Pole | 3.38 | -26 | 50 | 16 |
| 136 |  |  |  |  |  |
|  | R Cingulate Gyrus, posterior division | 3.53 | 4 | -20 | 30 |
| 136 |  |  |  |  |  |
|  | L Postcentral Gyrus | 3.10 | -36 | -28 | 50 |
| 128 |  |  |  |  |  |
|  | R Temporal Occipital Fusiform Cortex | 3.32 | 28 | -56 | -18 |
| 128 |  |  |  |  |  |
|  | R Frontal Pole | 3.38 | 26 | 68 | -4 |
| 120 |  |  |  |  |  |
|  | R Middle Temporal Gyrus, anterior division | 3.11 | 54 | 2 | -36 |
|  | R Inferior Temporal Gyrus, anterior division | 3.54 | 48 | 0 | -34 |
| 120 |  |  |  |  |  |
|  | L Brain-Stem | 4.11 | -12 | -30 | -30 |
| 120 |  |  |  |  |  |
|  | L Lateral Occipital Cortex, superior division | 3.29 | -32 | -60 | 40 |
| 104 |  |  |  |  |  |
|  | R Brain-Stem | 3.15 | 14 | -20 | -28 |
| 104 |  |  |  |  |  |
|  | R Occipital Fusiform Gyrus | 3.12 | 28 | -72 | -8 |
| 104 |  |  |  |  |  |
|  | L Insular Cortex | 3.66 | -36 | -20 | 12 |
| 96 |  |  |  |  |  |
|  | R Superior Temporal Gyrus, anterior division | 3.06 | 56 | 0 | -14 |
| 96 |  |  |  |  |  |
|  | R Putamen | 3.14 | 28 | -16 | 8 |
| 96 |  |  |  |  |  |
|  | R Inferior Frontal Gyrus, pars opercularis | 3.54 | 48 | 18 | 22 |
| 96 |  |  |  |  |  |
|  | L Precentral Gyrus | 3.51 | -6 | -26 | 50 |
| 88 |  |  |  |  |  |
|  | L Occipital Pole | 3.31 | -12 | -98 | 16 |
| 88 |  |  |  |  |  |
|  | R Precentral Gyrus | 3.05 | 62 | 4 | 32 |
| 80 |  |  |  |  |  |
|  | R Frontal Orbital Cortex | 3.58 | 36 | 26 | -16 |
| 80 |  |  |  |  |  |
|  | R Amygdala | 3.70 | 22 | -14 | -12 |
| 80 |  |  |  |  |  |
|  | R Frontal Pole | 3.32 | 28 | 62 | -10 |
| 80 |  |  |  |  |  |
|  | R Lateral Occipital Cortex, superior division | 2.77 | 42 | -76 | 20 |
|  | R Lateral Occipital Cortex, inferior division | 2.77 | 42 | -78 | 14 |
| 72 |  |  |  |  |  |
|  | L Occipital Fusiform Gyrus | 3.10 | -36 | -78 | -14 |
| 72 |  |  |  |  |  |
|  | R Precuneus Cortex | 3.47 | 2 | -44 | 48 |
| 64 |  |  |  |  |  |
|  | R Brain-Stem | 3.24 | 16 | -32 | -42 |
| 64 |  |  |  |  |  |
|  | R Inferior Temporal Gyrus, posterior division | 3.59 | 58 | -20 | -28 |
| 64 |  |  |  |  |  |
|  | L Amygdala | 4.88 | -18 | -12 | -14 |
| 64 |  |  |  |  |  |
|  | R Precentral Gyrus | 2.56 | 40 | 2 | 32 |
| 56 |  |  |  |  |  |
|  | L Middle Temporal Gyrus, posterior division | 2.81 | -62 | -30 | -12 |
| 56 |  |  |  |  |  |
|  | R Paracingulate Gyrus | 3.27 | 2 | 46 | -2 |
| 56 |  |  |  |  |  |
|  | L Thalamus | 3.40 | -10 | -10 | 6 |
| 56 |  |  |  |  |  |
|  | R Frontal Pole | 3.53 | 26 | 50 | 42 |
| 48 |  |  |  |  |  |
|  | R Brain-Stem | 3.47 | 6 | -28 | -42 |
| 48 |  |  |  |  |  |
|  | R Middle Temporal Gyrus, anterior division | 3.03 | 58 | -4 | -34 |
| 48 |  |  |  |  |  |
|  | L Hippocampus | 3.36 | -30 | -10 | -26 |
| 48 |  |  |  |  |  |
|  | L Lateral Occipital Cortex, inferior division | 2.94 | -48 | -78 | -16 |
| 48 |  |  |  |  |  |
|  | L Frontal Pole | 2.99 | -24 | 38 | -16 |
| 48 |  |  |  |  |  |
|  | L Frontal Pole | 3.42 | -22 | 66 | -2 |
| 48 |  |  |  |  |  |
|  | R Superior Temporal Gyrus, posterior division | 3.36 | 70 | -20 | 6 |
| 48 |  |  |  |  |  |
|  | R Angular Gyrus | 2.99 | 60 | -56 | 18 |
| 48 |  |  |  |  |  |
|  | L Lateral Occipital Cortex, superior division | 2.85 | -40 | -66 | 20 |
| 48 |  |  |  |  |  |
|  | R Caudate | 3.39 | 18 | -12 | 22 |
| 48 |  |  |  |  |  |
|  | R Cuneal Cortex | 3.39 | 10 | -78 | 30 |
| 48 |  |  |  |  |  |
|  | R Lateral Occipital Cortex, superior division | 3.58 | 26 | -78 | 32 |
| 48 |  |  |  |  |  |
|  | R Precentral Gyrus | 2.82 | 50 | -8 | 32 |
| 48 |  |  |  |  |  |
|  | L Precentral Gyrus | 3.08 | -8 | -22 | 58 |
| 48 |  |  |  |  |  |
|  | R Superior Frontal Gyrus | 2.79 | 26 | 2 | 62 |
| 48 |  |  |  |  |  |
|  | L Superior Frontal Gyrus | 3.14 | -14 | 2 | 68 |
| 40 |  |  |  |  |  |
|  | L Temporal Pole | 2.95 | -52 | 4 | -32 |
| 40 |  |  |  |  |  |
|  | L Caudate | 3.16 | -14 | -4 | 20 |
| 40 |  |  |  |  |  |
|  | R Precentral Gyrus | 3.09 | 56 | 6 | 28 |
| 40 |  |  |  |  |  |
|  | R Lateral Occipital Cortex, superior division | 2.80 | 16 | -84 | 30 |
|  | R Occipital Pole | 2.94 | 14 | -88 | 32 |
| 40 |  |  |  |  |  |
|  | L Middle Frontal Gyrus | 3.09 | -42 | 20 | 34 |
| 40 |  |  |  |  |  |
|  | R Superior Parietal Lobule | 3.17 | 16 | -48 | 64 |
| 32 |  |  |  |  |  |
|  | L Inferior Temporal Gyrus, anterior division | 3.25 | -50 | -2 | -34 |
| 32 |  |  |  |  |  |
|  | R Temporal Pole | 2.94 | 54 | 10 | -34 |
| 32 |  |  |  |  |  |
|  | L Inferior Temporal Gyrus, posterior division | 3.25 | -60 | -30 | -20 |
| 32 |  |  |  |  |  |
|  | R Hippocampus | 2.66 | 36 | -30 | -12 |
| 32 |  |  |  |  |  |
|  | L Lateral Occipital Cortex, inferior division | 3.14 | -40 | -70 | -10 |
| 32 |  |  |  |  |  |
|  | R Middle Temporal Gyrus, temporooccipital part | 2.76 | 66 | -46 | 2 |
| 32 |  |  |  |  |  |
|  | L Caudate | 3.18 | -8 | 12 | 6 |
| 32 |  |  |  |  |  |
|  | R Supramarginal Gyrus, posterior division | 3.04 | 66 | -42 | 10 |
| 32 |  |  |  |  |  |
|  | L Lateral Occipital Cortex, superior division | 2.83 | -36 | -84 | 14 |
| 32 |  |  |  |  |  |
|  | L Lateral Occipital Cortex, superior division | 2.72 | -56 | -66 | 22 |
| 32 |  |  |  |  |  |
|  | L Caudate | 3.36 | -14 | -10 | 20 |
| 32 |  |  |  |  |  |
|  | L Frontal Pole | 3.56 | -46 | 46 | 20 |
| 32 |  |  |  |  |  |
|  | L Cingulate Gyrus, posterior division | 3.59 | -6 | -38 | 26 |
| 32 |  |  |  |  |  |
|  | R Cingulate Gyrus, anterior division | 3.86 | 4 | 6 | 28 |
| 32 |  |  |  |  |  |
|  | R Cingulate Gyrus, posterior division | 3.13 | 2 | -42 | 40 |
| 32 |  |  |  |  |  |
|  | R Postcentral Gyrus | 2.94 | 20 | -36 | 62 |
| 32 |  |  |  |  |  |
|  | L Precentral Gyrus | 2.76 | -28 | -14 | 66 |
| 24 |  |  |  |  |  |
|  | L Brain-Stem | 3.11 | -10 | -30 | -40 |
| 24 |  |  |  |  |  |
|  | L Parahippocampal Gyrus, anterior division | 3.32 | -22 | -6 | -36 |
| 24 |  |  |  |  |  |
|  | R Brain-Stem | 3.31 | 8 | -24 | -32 |
| 24 |  |  |  |  |  |
|  | R Inferior Temporal Gyrus, posterior division | 2.84 | 62 | -32 | -26 |
| 24 |  |  |  |  |  |
|  | R Frontal Orbital Cortex | 2.84 | 16 | 24 | -26 |
| 24 |  |  |  |  |  |
|  | L Hippocampus | 2.99 | -26 | -18 | -20 |
| 24 |  |  |  |  |  |
|  | R Middle Temporal Gyrus, posterior division | 3.47 | 48 | -14 | -16 |
| 24 |  |  |  |  |  |
|  | R Frontal Pole | 2.83 | 34 | 34 | -14 |
| 24 |  |  |  |  |  |
|  | L Occipital Pole | 3.00 | -16 | -92 | -10 |
| 24 |  |  |  |  |  |
|  | L Lateral Occipital Cortex, inferior division | 2.94 | -50 | -80 | -10 |
| 24 |  |  |  |  |  |
|  | L Planum Polare | 3.24 | -46 | -10 | -2 |
| 24 |  |  |  |  |  |
|  | R Cingulate Gyrus, anterior division | 2.76 | 4 | 34 | 10 |
| 24 |  |  |  |  |  |
|  | L Frontal Pole | 2.82 | -42 | 50 | 14 |
| 24 |  |  |  |  |  |
|  | L Supramarginal Gyrus, posterior division | 2.88 | -64 | -50 | 18 |
| 24 |  |  |  |  |  |
|  | L Occipital Pole | 3.26 | -14 | -92 | 22 |
| 24 |  |  |  |  |  |
|  | L Lateral Occipital Cortex, superior division | 2.97 | -34 | -78 | 28 |
| 24 |  |  |  |  |  |
|  | R Precentral Gyrus | 2.64 | 44 | -14 | 38 |
| 24 |  |  |  |  |  |
|  | R Postcentral Gyrus | 3.79 | 46 | -28 | 54 |
| 16 |  |  |  |  |  |
|  | R Temporal Fusiform Cortex, anterior division | 2.59 | 30 | -6 | -44 |
| 16 |  |  |  |  |  |
|  | R Middle Temporal Gyrus, posterior division | 2.91 | 60 | -10 | -30 |
| 16 |  |  |  |  |  |
|  | L Temporal Pole | 3.40 | -34 | 10 | -30 |
| 16 |  |  |  |  |  |
|  | R Temporal Fusiform Cortex, posterior division | 2.61 | 42 | -32 | -26 |
| 16 |  |  |  |  |  |
|  | R Hippocampus | 2.81 | 32 | -8 | -26 |
| 16 |  |  |  |  |  |
|  | L Middle Temporal Gyrus, anterior division | 2.90 | -56 | -8 | -26 |
| 16 |  |  |  |  |  |
|  | L Inferior Temporal Gyrus, posterior division | 2.66 | -50 | -42 | -24 |
| 16 |  |  |  |  |  |
|  | L Temporal Pole | 3.21 | -42 | 14 | -24 |
| 16 |  |  |  |  |  |
|  | L Middle Temporal Gyrus, posterior division | 2.71 | -64 | -16 | -22 |
| 16 |  |  |  |  |  |
|  | R Hippocampus | 2.68 | 26 | -20 | -14 |
| 16 |  |  |  |  |  |
|  | R Hippocampus | 2.64 | 28 | -28 | -12 |
| 16 |  |  |  |  |  |
|  | L Frontal Pole | 2.65 | -16 | 62 | -12 |
| 16 |  |  |  |  |  |
|  | L Middle Temporal Gyrus, posterior division | 2.78 | -66 | -24 | -10 |
| 16 |  |  |  |  |  |
|  | L Frontal Pole | 2.96 | -14 | 68 | -6 |
| 16 |  |  |  |  |  |
|  | R Lateral Occipital Cortex, inferior division | 3.13 | 36 | -80 | 2 |
| 16 |  |  |  |  |  |
|  | L Lingual Gyrus | 2.87 | -20 | -60 | 2 |
| 16 |  |  |  |  |  |
|  | R Insular Cortex | 2.63 | 42 | -14 | 2 |
| 16 |  |  |  |  |  |
|  | R Paracingulate Gyrus | 3.14 | 8 | 46 | 4 |
| 16 |  |  |  |  |  |
|  | L Occipital Pole | 2.68 | -34 | -94 | 6 |
| 16 |  |  |  |  |  |
|  | R Middle Temporal Gyrus, temporooccipital part | 2.76 | 62 | -52 | 6 |
| 16 |  |  |  |  |  |
|  | L Central Opercular Cortex | 3.18 | -46 | -10 | 6 |
| 16 |  |  |  |  |  |
|  | L Insular Cortex | 2.72 | -32 | -26 | 8 |
| 16 |  |  |  |  |  |
|  | R Cingulate Gyrus, anterior division | 3.07 | 12 | 40 | 8 |
| 16 |  |  |  |  |  |
|  | L Middle Temporal Gyrus, temporooccipital part | 2.64 | -46 | -54 | 10 |
| 16 |  |  |  |  |  |
|  | L Thalamus | 2.87 | -8 | -2 | 10 |
| 16 |  |  |  |  |  |
|  | R Occipital Pole | 2.89 | 10 | -90 | 14 |
| 16 |  |  |  |  |  |
|  | R Frontal Pole | 2.65 | 38 | 42 | 18 |
| 16 |  |  |  |  |  |
|  | R Parietal Operculum Cortex | 2.92 | 44 | -20 | 18 |
| 16 |  |  |  |  |  |
|  | R Parietal Operculum Cortex | 2.67 | 38 | -30 | 20 |
| 16 |  |  |  |  |  |
|  | R Precentral Gyrus | 2.82 | 62 | 2 | 22 |
| 16 |  |  |  |  |  |
|  | R Lateral Occipital Cortex, superior division | 3.22 | 40 | -74 | 24 |
| 16 |  |  |  |  |  |
|  | R Cingulate Gyrus, posterior division | 2.64 | 6 | -44 | 34 |
| 16 |  |  |  |  |  |
|  | L Cingulate Gyrus, posterior division | 2.59 | -12 | -46 | 34 |
| 16 |  |  |  |  |  |
|  | L Middle Frontal Gyrus | 2.74 | -38 | 20 | 40 |
| 16 |  |  |  |  |  |
|  | L Precentral Gyrus | 3.15 | -8 | -34 | 48 |
| 16 |  |  |  |  |  |
|  | R Superior Frontal Gyrus | 3.37 | 6 | 36 | 48 |
| 16 |  |  |  |  |  |
|  | R Postcentral Gyrus | 2.81 | 54 | -14 | 50 |
| 16 |  |  |  |  |  |
|  | R Superior Frontal Gyrus | 2.63 | 22 | 0 | 56 |
| 16 |  |  |  |  |  |
|  | R Precuneus Cortex | 2.53 | 12 | -56 | 56 |
| 8 |  |  |  |  |  |
|  | L Brain-Stem | 2.67 | -4 | -44 | -60 |
| 8 |  |  |  |  |  |
|  | L Temporal Pole | 2.77 | -36 | 14 | -44 |
| 8 |  |  |  |  |  |
|  | L Brain-Stem | 2.69 | -12 | -34 | -42 |
| 8 |  |  |  |  |  |
|  | L Brain-Stem | 2.87 | -2 | -26 | -42 |
| 8 |  |  |  |  |  |
|  | L Brain-Stem | 2.54 | -6 | -22 | -40 |
| 8 |  |  |  |  |  |
|  | R Temporal Fusiform Cortex, posterior division | 2.68 | 42 | -16 | -38 |
| 8 |  |  |  |  |  |
|  | L Brain-Stem | 2.65 | -20 | -30 | -34 |
| 8 |  |  |  |  |  |
|  | L Brain-Stem | 2.59 | -20 | -26 | -34 |
| 8 |  |  |  |  |  |
|  | R Inferior Temporal Gyrus, posterior division | 2.56 | 46 | -20 | -34 |
| 8 |  |  |  |  |  |
|  | R Inferior Temporal Gyrus, posterior division | 2.67 | 46 | -22 | -28 |
| 8 |  |  |  |  |  |
|  | L Brain-Stem | 2.57 | -6 | -20 | -28 |
| 8 |  |  |  |  |  |
|  | L Inferior Temporal Gyrus, posterior division | 2.58 | -56 | -44 | -24 |
| 8 |  |  |  |  |  |
|  | R Amygdala | 2.97 | 30 | 4 | -22 |
|  | R Amygdala | 2.97 | 30 | 4 | -22 |
| 8 |  |  |  |  |  |
|  | R Inferior Temporal Gyrus, temporooccipital part | 2.55 | 60 | -50 | -20 |
| 8 |  |  |  |  |  |
|  | R Inferior Temporal Gyrus, posterior division | 2.60 | 58 | -28 | -20 |
| 8 |  |  |  |  |  |
|  | L Middle Temporal Gyrus, posterior division | 2.54 | -66 | -24 | -18 |
| 8 |  |  |  |  |  |
|  | L Hippocampus | 2.81 | -34 | -16 | -18 |
| 8 |  |  |  |  |  |
|  | R Middle Temporal Gyrus, posterior division | 2.54 | 52 | -12 | -18 |
| 8 |  |  |  |  |  |
|  | R Hippocampus | 2.62 | 24 | -12 | -18 |
| 8 |  |  |  |  |  |
|  | L Inferior Temporal Gyrus, temporooccipital part | 2.81 | -52 | -60 | -16 |
| 8 |  |  |  |  |  |
|  | R Lateral Occipital Cortex, inferior division | 2.65 | 50 | -76 | -14 |
| 8 |  |  |  |  |  |
|  | R Occipital Fusiform Gyrus | 2.71 | 32 | -72 | -14 |
| 8 |  |  |  |  |  |
|  | R Parahippocampal Gyrus, posterior division | 2.71 | 18 | -30 | -14 |
| 8 |  |  |  |  |  |
|  | L Frontal Pole | 2.56 | -28 | 50 | -14 |
| 8 |  |  |  |  |  |
|  | L Frontal Pole | 3.37 | -18 | 54 | -14 |
| 8 |  |  |  |  |  |
|  | L Frontal Orbital Cortex | 2.82 | -26 | 36 | -12 |
| 8 |  |  |  |  |  |
|  | R Middle Temporal Gyrus, posterior division | 2.62 | 60 | -12 | -10 |
| 8 |  |  |  |  |  |
|  | L Superior Temporal Gyrus, anterior division | 2.54 | -56 | -4 | -10 |
| 8 |  |  |  |  |  |
|  | R Planum Polare | 2.57 | 50 | 4 | -8 |
| 8 |  |  |  |  |  |
|  | L Subcallosal Cortex | 2.54 | -6 | 26 | -8 |
| 8 |  |  |  |  |  |
|  | L Planum Polare | 2.61 | -48 | -8 | -6 |
| 8 |  |  |  |  |  |
|  | L Middle Temporal Gyrus, temporooccipital part | 2.73 | -52 | -60 | -4 |
| 8 |  |  |  |  |  |
|  | R Middle Temporal Gyrus, temporooccipital part | 2.54 | 60 | -54 | -2 |
| 8 |  |  |  |  |  |
|  | L Lingual Gyrus | 2.73 | -18 | -50 | -2 |
| 8 |  |  |  |  |  |
|  | L Frontal Pole | 2.61 | -14 | 70 | -2 |
| 8 |  |  |  |  |  |
|  | L Pallidum | 2.74 | -24 | -10 | 0 |
| 8 |  |  |  |  |  |
|  | L Lateral Occipital Cortex, inferior division | 2.52 | -40 | -74 | 4 |
| 8 |  |  |  |  |  |
|  | L Lingual Gyrus | 2.72 | -4 | -70 | 4 |
| 8 |  |  |  |  |  |
|  | R Inferior Frontal Gyrus, pars triangularis | 2.56 | 50 | 32 | 4 |
| 8 |  |  |  |  |  |
|  | R Superior Temporal Gyrus, posterior division | 2.52 | 70 | -30 | 8 |
| 8 |  |  |  |  |  |
|  | L Heschls Gyrus (includes H1 and H2) | 2.51 | -42 | -18 | 10 |
| 8 |  |  |  |  |  |
|  | L Putamen | 2.57 | -24 | 4 | 12 |
| 8 |  |  |  |  |  |
|  | L Occipital Pole | 2.76 | -20 | -92 | 14 |
| 8 |  |  |  |  |  |
|  | L Lateral Occipital Cortex, superior division | 2.53 | -44 | -70 | 16 |
| 8 |  |  |  |  |  |
|  | R Cingulate Gyrus, anterior division | 2.56 | 4 | 24 | 16 |
| 8 |  |  |  |  |  |
|  | L Frontal Pole | 2.54 | -26 | 56 | 16 |
| 8 |  |  |  |  |  |
|  | L Thalamus | 2.56 | -8 | -14 | 18 |
| 8 |  |  |  |  |  |
|  | L Central Opercular Cortex | 2.82 | -40 | -8 | 18 |
| 8 |  |  |  |  |  |
|  | L Caudate | 2.59 | -18 | 6 | 18 |
| 8 |  |  |  |  |  |
|  | R Precentral Gyrus | 2.65 | 58 | 4 | 20 |
| 8 |  |  |  |  |  |
|  | R Frontal Pole | 2.62 | 42 | 42 | 20 |
| 8 |  |  |  |  |  |
|  | L Occipital Pole | 2.62 | -20 | -96 | 22 |
| 8 |  |  |  |  |  |
|  | R Cingulate Gyrus, posterior division | 2.60 | 4 | -40 | 24 |
| 8 |  |  |  |  |  |
|  | L Postcentral Gyrus | 3.02 | -56 | -14 | 26 |
| 8 |  |  |  |  |  |
|  | L Frontal Pole | 2.53 | -26 | 44 | 26 |
| 8 |  |  |  |  |  |
|  | R Frontal Pole | 3.12 | 10 | 58 | 26 |
| 8 |  |  |  |  |  |
|  | R Frontal Pole | 2.56 | 46 | 44 | 28 |
| 8 |  |  |  |  |  |
|  | L Frontal Pole | 2.64 | -24 | 46 | 28 |
| 8 |  |  |  |  |  |
|  | R Frontal Pole | 2.65 | 24 | 38 | 32 |
| 8 |  |  |  |  |  |
|  | R Middle Frontal Gyrus | 2.72 | 38 | 26 | 34 |
| 8 |  |  |  |  |  |
|  | L Cingulate Gyrus, posterior division | 2.51 | -10 | -38 | 36 |
| 8 |  |  |  |  |  |
|  | R Precentral Gyrus | 2.60 | 56 | 8 | 36 |
| 8 |  |  |  |  |  |
|  | R Superior Frontal Gyrus | 2.62 | 8 | 40 | 38 |
| 8 |  |  |  |  |  |
|  | L Precuneus Cortex | 2.65 | -10 | -48 | 42 |
| 8 |  |  |  |  |  |
|  | L Cingulate Gyrus, posterior division | 2.69 | -4 | -18 | 42 |
| 8 |  |  |  |  |  |
|  | L Frontal Pole | 2.54 | -26 | 44 | 44 |
| 8 |  |  |  |  |  |
|  | L Lateral Occipital Cortex, superior division | 2.53 | -26 | -60 | 46 |
| 8 |  |  |  |  |  |
|  | L Postcentral Gyrus | 2.63 | -38 | -24 | 46 |
| 8 |  |  |  |  |  |
|  | R Middle Frontal Gyrus | 2.59 | 34 | 28 | 46 |
| 8 |  |  |  |  |  |
|  | L Precentral Gyrus | 2.53 | -32 | -10 | 58 |
| 8 |  |  |  |  |  |
|  | R Superior Frontal Gyrus | 2.62 | 20 | 0 | 60 |
| 8 |  |  |  |  |  |
|  | L Precentral Gyrus | 2.60 | -24 | -20 | 68 |
| 8 |  |  |  |  |  |
|  | L Precentral Gyrus | 2.64 | -18 | -24 | 70 |

# Supplementary Table S14. [UT_phasic _> US_phasic_]. Descriptive statistics for clusters and local maxima showing greater activation for Uncertain-Threat relative to Uncertain-Safety anticipation for the OSP Phasic regressor (FDR *q*<0.05, whole-brain corrected). 

| ***mm***^***3***^ | **Label** | ***t*** | ***x*** | ***y*** | ***z*** |
| --- | --- | --- | --- | --- | --- |
| 4,088 |  |  |  |  |  |
|  | R Inferior Frontal Gyrus, pars triangularis | 3.47 | 50 | 24 | 8 |
|  | R Insular Cortex | 6.17 | 38 | 20 | -4 |
|  | R Temporal Pole | 4.40 | 50 | 20 | -14 |
|  | R Frontal Orbital Cortex | 3.82 | 38 | 20 | -16 |
|  | R Frontal Operculum Cortex | 5.87 | 46 | 14 | -2 |
|  | R Inferior Frontal Gyrus, pars opercularis | 3.43 | 56 | 12 | 4 |
|  | R Central Opercular Cortex | 4.52 | 38 | 10 | 8 |
|  | R Precentral Gyrus | 3.56 | 60 | 6 | 6 |
| 3,944 |  |  |  |  |  |
|  | R Postcentral Gyrus | 3.82 | 64 | -18 | 22 |
|  | R Supramarginal Gyrus, anterior division | 4.77 | 64 | -26 | 26 |
|  | R Parietal Operculum Cortex | 4.87 | 56 | -28 | 24 |
|  | R Supramarginal Gyrus, posterior division | 4.01 | 56 | -40 | 20 |
|  | R Middle Temporal Gyrus, temporooccipital part | 5.93 | 64 | -44 | 8 |
|  | R Angular Gyrus | 4.40 | 54 | -48 | 16 |
| 3,776 |  |  |  |  |  |
|  | R Thalamus | 6.64 | 6 | -22 | 6 |
|  | L Thalamus | 5.01 | -6 | -24 | 10 |
|  | L Brain-Stem | 6.22 | -4 | -34 | -6 |
|  | R Brain-Stem | 5.78 | 4 | -36 | -8 |
| 3,008 |  |  |  |  |  |
|  | L Postcentral Gyrus | 3.70 | -62 | -18 | 20 |
|  | L Supramarginal Gyrus, anterior division | 5.60 | -64 | -28 | 20 |
|  | L Supramarginal Gyrus, posterior division | 5.64 | -60 | -44 | 18 |
|  | L Angular Gyrus | 4.17 | -54 | -54 | 44 |
| 2,744 |  |  |  |  |  |
|  | R Frontal Pole | 5.60 | 32 | 50 | 28 |
|  | R Middle Frontal Gyrus | 3.68 | 42 | 34 | 36 |
| 2,640 |  |  |  |  |  |
|  | L Frontal Orbital Cortex | 4.80 | -32 | 24 | -6 |
|  | L Frontal Operculum Cortex | 5.14 | -40 | 14 | 0 |
|  | L Insular Cortex | 4.40 | -44 | 10 | -6 |
|  | L Central Opercular Cortex | 4.59 | -38 | 8 | 6 |
|  | L Putamen | 5.17 | -30 | 6 | 0 |
|  | L Pallidum | 3.80 | -16 | 4 | -2 |
|  | L Planum Polare | 3.55 | -54 | -2 | -2 |
| 2,176 |  |  |  |  |  |
|  | R Postcentral Gyrus | 4.44 | 60 | -8 | 30 |
|  | R Precentral Gyrus | 5.65 | 40 | -12 | 48 |
| 2,144 |  |  |  |  |  |
|  | L Precentral Gyrus | 6.15 | -46 | -12 | 32 |
|  | L Postcentral Gyrus | 4.39 | -40 | -18 | 42 |
| 1,824 |  |  |  |  |  |
|  | L Precentral Gyrus | 6.53 | -20 | -30 | 58 |
|  | L Postcentral Gyrus | 4.27 | -20 | -34 | 68 |
| 1,624 |  |  |  |  |  |
|  | R Precentral Gyrus | 7.06 | 16 | -26 | 70 |
|  | R Postcentral Gyrus | 4.42 | 24 | -28 | 58 |
| 1,360 |  |  |  |  |  |
|  | R Intracalcarine Cortex | 4.97 | 10 | -76 | 14 |
| 1,184 |  |  |  |  |  |
|  | R Frontal Pole | 4.69 | 42 | 44 | 4 |
| 1,136 |  |  |  |  |  |
|  | L Intracalcarine Cortex | 5.15 | -8 | -70 | 8 |
| 1,104 |  |  |  |  |  |
|  | L Insular Cortex | 4.82 | -42 | -10 | -4 |
|  | L Putamen | 4.53 | -32 | -10 | -4 |
|  | L Planum Polare | 4.93 | -44 | -14 | -2 |
| 1,000 |  |  |  |  |  |
|  | R Putamen | 4.20 | 22 | 10 | 0 |
|  | R Pallidum | 4.33 | 20 | 2 | -2 |
| 992 |  |  |  |  |  |
|  | R Supramarginal Gyrus, anterior division | 3.79 | 58 | -34 | 48 |
|  | R Supramarginal Gyrus, posterior division | 5.04 | 60 | -46 | 42 |
|  | R Angular Gyrus | 3.54 | 50 | -48 | 50 |
| 464 |  |  |  |  |  |
|  | L Central Opercular Cortex | 3.62 | -38 | -20 | 20 |
|  | L Insular Cortex | 4.88 | -32 | -28 | 16 |
|  | L Parietal Operculum Cortex | 4.21 | -36 | -30 | 20 |
| 408 |  |  |  |  |  |
|  | L Cingulate Gyrus, posterior division | 4.67 | -6 | -20 | 46 |
|  | L Precentral Gyrus | 4.23 | -12 | -24 | 44 |
| 400 |  |  |  |  |  |
|  | R Insular Cortex | 3.43 | 42 | -10 | 4 |
|  | R Planum Polare | 4.14 | 42 | -14 | -4 |
| 352 |  |  |  |  |  |
|  | R Paracingulate Gyrus | 4.58 | 8 | 18 | 36 |
| 344 |  |  |  |  |  |
|  | L Precentral Gyrus | 5.35 | -8 | -26 | 58 |
| 312 |  |  |  |  |  |
|  | R Precentral Gyrus | 4.99 | 6 | -30 | 60 |
| 272 |  |  |  |  |  |
|  | R Cingulate Gyrus, anterior division | 5.21 | 0 | 32 | 0 |
|  | R Subcallosal Cortex | 3.90 | 0 | 26 | -4 |
| 264 |  |  |  |  |  |
|  | L Middle Temporal Gyrus, temporooccipital part | 4.28 | -54 | -54 | 6 |
| 264 |  |  |  |  |  |
|  | L Paracingulate Gyrus | 4.79 | -4 | 30 | 32 |
| 248 |  |  |  |  |  |
|  | R Lateral Occipital Cortex, superior division | 3.79 | 50 | -58 | 46 |
|  | R Angular Gyrus | 3.19 | 52 | -58 | 32 |
| 224 |  |  |  |  |  |
|  | L Postcentral Gyrus | 4.93 | -60 | -6 | 16 |
| 200 |  |  |  |  |  |
|  | L Inferior Temporal Gyrus, posterior division | 3.88 | -52 | -42 | -24 |
| 200 |  |  |  |  |  |
|  | R Thalamus | 5.73 | 6 | -4 | -2 |
| 160 |  |  |  |  |  |
|  | L Frontal Pole | 4.76 | -26 | 52 | 10 |
| 152 |  |  |  |  |  |
|  | R Middle Temporal Gyrus, posterior division | 3.88 | 64 | -30 | 0 |
| 152 |  |  |  |  |  |
|  | R Caudate | 5.06 | 14 | 24 | -2 |
| 144 |  |  |  |  |  |
|  | L Cingulate Gyrus, anterior division | 4.22 | -8 | 26 | 26 |
| 136 |  |  |  |  |  |
|  | R Frontal Pole | 4.12 | 26 | 34 | -10 |
| 112 |  |  |  |  |  |
|  | L Insular Cortex | 4.33 | -40 | 10 | -12 |
| 112 |  |  |  |  |  |
|  | L Parietal Operculum Cortex | 4.20 | -48 | -38 | 22 |
| 104 |  |  |  |  |  |
|  | R Frontal Pole | 3.61 | 26 | 64 | -2 |
| 104 |  |  |  |  |  |
|  | R Angular Gyrus | 3.86 | 60 | -52 | 24 |
| 96 |  |  |  |  |  |
|  | R Brain-Stem | 3.64 | 4 | -20 | -24 |
| 96 |  |  |  |  |  |
|  | L Inferior Temporal Gyrus, temporooccipital part | 3.44 | -60 | -48 | -14 |
| 96 |  |  |  |  |  |
|  | L Angular Gyrus | 3.76 | -48 | -54 | 28 |
|  | L Lateral Occipital Cortex, superior division | 3.61 | -46 | -62 | 26 |
| 96 |  |  |  |  |  |
|  | L Lateral Occipital Cortex, superior division | 3.96 | -30 | -80 | 32 |
| 96 |  |  |  |  |  |
|  | R Cingulate Gyrus, posterior division | 3.53 | 6 | -36 | 34 |
| 88 |  |  |  |  |  |
|  | R Cingulate Gyrus, posterior division | 3.94 | 0 | -22 | 28 |
| 88 |  |  |  |  |  |
|  | R Paracingulate Gyrus | 3.28 | 2 | 16 | 46 |
|  | L Paracingulate Gyrus | 3.54 | -2 | 12 | 46 |
| 80 |  |  |  |  |  |
|  | R Frontal Pole | 3.79 | 22 | 44 | -14 |
| 80 |  |  |  |  |  |
|  | L Frontal Pole | 3.51 | -28 | 52 | 30 |
| 72 |  |  |  |  |  |
|  | L Brain-Stem | 3.42 | -8 | -38 | -26 |
| 72 |  |  |  |  |  |
|  | R Putamen | 4.47 | 24 | -4 | 6 |
| 64 |  |  |  |  |  |
|  | R Insular Cortex | 3.85 | 38 | -18 | 2 |
| 64 |  |  |  |  |  |
|  | L Frontal Pole | 3.16 | -50 | 38 | 10 |
|  | L Inferior Frontal Gyrus, pars triangularis | 3.34 | -50 | 34 | 10 |
| 56 |  |  |  |  |  |
|  | L Brain-Stem | 3.83 | -8 | -28 | -12 |
| 56 |  |  |  |  |  |
|  | R Middle Temporal Gyrus, posterior division | 3.67 | 64 | -22 | -6 |
| 56 |  |  |  |  |  |
|  | R Frontal Pole | 3.32 | 48 | 48 | -4 |
| 56 |  |  |  |  |  |
|  | L Frontal Pole | 4.18 | -26 | 52 | 4 |
| 56 |  |  |  |  |  |
|  | R Middle Frontal Gyrus | 3.65 | 52 | 28 | 26 |
| 56 |  |  |  |  |  |
|  | R Cingulate Gyrus, anterior division | 3.34 | 0 | 16 | 32 |
| 56 |  |  |  |  |  |
|  | R Superior Frontal Gyrus | 4.21 | 4 | 34 | 58 |
| 48 |  |  |  |  |  |
|  | L Brain-Stem | 3.45 | -6 | -40 | -52 |
|  | R Brain-Stem | 3.54 | 0 | -40 | -52 |
| 48 |  |  |  |  |  |
|  | R Middle Temporal Gyrus, posterior division | 3.59 | 58 | -28 | -2 |
| 48 |  |  |  |  |  |
|  | L Insular Cortex | 4.22 | -34 | -24 | 6 |
| 48 |  |  |  |  |  |
|  | L Precentral Gyrus | 3.53 | -58 | 4 | 10 |
| 48 |  |  |  |  |  |
|  | R Insular Cortex | 3.39 | 36 | -12 | 14 |
| 48 |  |  |  |  |  |
|  | L Postcentral Gyrus | 3.86 | -60 | -20 | 34 |
| 48 |  |  |  |  |  |
|  | L Precuneus Cortex | 3.18 | -8 | -68 | 34 |
| 48 |  |  |  |  |  |
|  | R Frontal Pole | 4.19 | 24 | 44 | 46 |
| 40 |  |  |  |  |  |
|  | R Frontal Orbital Cortex | 3.92 | 28 | 26 | -16 |
| 40 |  |  |  |  |  |
|  | L Frontal Orbital Cortex | 3.29 | -40 | 20 | -14 |
| 40 |  |  |  |  |  |
|  | L Parahippocampal Gyrus, posterior division | 3.72 | -20 | -38 | -12 |
| 40 |  |  |  |  |  |
|  | L Putamen | 3.67 | -20 | 2 | -8 |
| 40 |  |  |  |  |  |
|  | R Frontal Pole | 4.30 | 26 | 52 | -2 |
| 40 |  |  |  |  |  |
|  | L Frontal Pole | 3.10 | -48 | 46 | -2 |
| 40 |  |  |  |  |  |
|  | R Frontal Pole | 3.56 | 12 | 58 | 26 |
| 40 |  |  |  |  |  |
|  | L Middle Frontal Gyrus | 3.55 | -46 | 12 | 36 |
| 40 |  |  |  |  |  |
|  | L Lateral Occipital Cortex, superior division | 3.17 | -42 | -70 | 42 |
| 40 |  |  |  |  |  |
|  | R Precentral Gyrus | 3.82 | 24 | -14 | 64 |
| 40 |  |  |  |  |  |
|  | L Precentral Gyrus | 3.19 | -16 | -16 | 66 |
| 32 |  |  |  |  |  |
|  | R Temporal Pole | 3.60 | 34 | 24 | -42 |
| 32 |  |  |  |  |  |
|  | L Brain-Stem | 3.33 | -4 | -16 | -22 |
| 32 |  |  |  |  |  |
|  | R Lateral Occipital Cortex, inferior division | 3.40 | 58 | -62 | -4 |
| 32 |  |  |  |  |  |
|  | L Insular Cortex | 3.46 | -42 | -4 | 2 |
| 32 |  |  |  |  |  |
|  | R Occipital Pole | 3.60 | 0 | -98 | 12 |
| 32 |  |  |  |  |  |
|  | L Lateral Occipital Cortex, superior division | 3.36 | -34 | -84 | 8 |
| 32 |  |  |  |  |  |
|  | L Central Opercular Cortex | 3.86 | -44 | -14 | 10 |
| 32 |  |  |  |  |  |
|  | R Cingulate Gyrus, anterior division | 3.68 | 8 | 34 | 20 |
| 32 |  |  |  |  |  |
|  | L Inferior Frontal Gyrus, pars opercularis | 3.23 | -58 | 14 | 22 |
| 32 |  |  |  |  |  |
|  | R Paracingulate Gyrus | 3.60 | 0 | 38 | 22 |
| 32 |  |  |  |  |  |
|  | R Middle Frontal Gyrus | 3.59 | 50 | 34 | 28 |
| 32 |  |  |  |  |  |
|  | L Paracingulate Gyrus | 3.36 | -4 | 42 | 28 |
| 32 |  |  |  |  |  |
|  | R Lateral Occipital Cortex, superior division | 3.97 | 28 | -76 | 32 |
| 32 |  |  |  |  |  |
|  | R Frontal Pole | 3.27 | 4 | 58 | 34 |
| 32 |  |  |  |  |  |
|  | L Frontal Pole | 3.60 | -36 | 40 | 38 |
| 32 |  |  |  |  |  |
|  | L Cingulate Gyrus, anterior division | 3.12 | -6 | -8 | 44 |
| 32 |  |  |  |  |  |
|  | R Precentral Gyrus | 3.60 | 6 | -20 | 48 |
| 32 |  |  |  |  |  |
|  | R Supramarginal Gyrus, posterior division | 3.02 | 52 | -38 | 50 |
| 32 |  |  |  |  |  |
|  | R Precentral Gyrus | 3.71 | 44 | -8 | 56 |
| 32 |  |  |  |  |  |
|  | L Precentral Gyrus | 4.18 | -4 | -36 | 58 |
| 32 |  |  |  |  |  |
|  | R Precentral Gyrus | 3.45 | 6 | -22 | 66 |
| 32 |  |  |  |  |  |
|  | R Postcentral Gyrus | 3.49 | 24 | -32 | 70 |
| 24 |  |  |  |  |  |
|  | L Brain-Stem | 3.56 | -8 | -44 | -38 |
| 24 |  |  |  |  |  |
|  | L Lateral Occipital Cortex, inferior division | 3.45 | -36 | -82 | -18 |
| 24 |  |  |  |  |  |
|  | R Superior Temporal Gyrus, anterior division | 3.84 | 60 | 4 | -14 |
| 24 |  |  |  |  |  |
|  | L Frontal Orbital Cortex | 3.74 | -26 | 34 | -14 |
| 24 |  |  |  |  |  |
|  | L Superior Temporal Gyrus, anterior division | 3.26 | -50 | -4 | -12 |
| 24 |  |  |  |  |  |
|  | R Parahippocampal Gyrus, posterior division | 3.59 | 18 | -34 | -10 |
| 24 |  |  |  |  |  |
|  | R Lateral Occipital Cortex, inferior division | 3.83 | 48 | -78 | -8 |
| 24 |  |  |  |  |  |
|  | L Insular Cortex | 3.30 | -36 | 0 | -6 |
| 24 |  |  |  |  |  |
|  | L Temporal Pole | 3.41 | -56 | 4 | -6 |
| 24 |  |  |  |  |  |
|  | R Lateral Occipital Cortex, inferior division | 3.27 | 38 | -90 | -4 |
| 24 |  |  |  |  |  |
|  | R Frontal Pole | 3.43 | 38 | 56 | -2 |
| 24 |  |  |  |  |  |
|  | L Lateral Occipital Cortex, inferior division | 3.21 | -46 | -86 | -2 |
| 24 |  |  |  |  |  |
|  | R Hippocampus | 3.89 | 26 | -36 | 0 |
| 24 |  |  |  |  |  |
|  | L Lateral Occipital Cortex, inferior division | 3.48 | -56 | -70 | 6 |
| 24 |  |  |  |  |  |
|  | L Thalamus | 3.26 | -14 | -22 | 8 |
| 24 |  |  |  |  |  |
|  | R Frontal Pole | 3.20 | 24 | 60 | 6 |
| 24 |  |  |  |  |  |
|  | R Frontal Pole | 3.42 | 34 | 64 | 10 |
| 24 |  |  |  |  |  |
|  | L Thalamus | 3.08 | -18 | -26 | 12 |
| 24 |  |  |  |  |  |
|  | R Putamen | 3.58 | 26 | 8 | 10 |
| 24 |  |  |  |  |  |
|  | R Planum Temporale | 3.41 | 46 | -28 | 14 |
| 24 |  |  |  |  |  |
|  | R Cingulate Gyrus, anterior division | 3.43 | 0 | 14 | 24 |
| 24 |  |  |  |  |  |
|  | R Inferior Frontal Gyrus, pars opercularis | 3.55 | 52 | 20 | 24 |
| 24 |  |  |  |  |  |
|  | L Lateral Occipital Cortex, superior division | 3.16 | -44 | -60 | 36 |
| 24 |  |  |  |  |  |
|  | R Cingulate Gyrus, posterior division | 3.08 | 0 | -28 | 34 |
|  | L Cingulate Gyrus, posterior division | 3.16 | -2 | -30 | 36 |
| 24 |  |  |  |  |  |
|  | L Superior Frontal Gyrus | 3.44 | -6 | 36 | 42 |
| 24 |  |  |  |  |  |
|  | R Superior Frontal Gyrus | 3.24 | 4 | 48 | 42 |
| 24 |  |  |  |  |  |
|  | L Postcentral Gyrus | 3.38 | -54 | -20 | 50 |
| 24 |  |  |  |  |  |
|  | L Juxtapositional Lobule Cortex (formerly Supplementary Motor Cortex) | 3.62 | -6 | -14 | 52 |
| 16 |  |  |  |  |  |
|  | R Brain-Stem | 3.23 | 4 | -44 | -56 |
| 16 |  |  |  |  |  |
|  | L Brain-Stem | 3.69 | -4 | -38 | -46 |
| 16 |  |  |  |  |  |
|  | R Brain-Stem | 3.00 | 6 | -14 | -38 |
| 16 |  |  |  |  |  |
|  | R Brain-Stem | 3.04 | 6 | -14 | -28 |
| 16 |  |  |  |  |  |
|  | L Brain-Stem | 3.23 | -4 | -36 | -16 |
| 16 |  |  |  |  |  |
|  | L Lateral Occipital Cortex, inferior division | 3.22 | -42 | -78 | -14 |
| 16 |  |  |  |  |  |
|  | R Frontal Orbital Cortex | 3.20 | 38 | 26 | -14 |
| 16 |  |  |  |  |  |
|  | R Lateral Occipital Cortex, inferior division | 3.38 | 46 | -76 | -12 |
| 16 |  |  |  |  |  |
|  | L Hippocampus | 4.08 | -28 | -34 | -8 |
| 16 |  |  |  |  |  |
|  | L Frontal Orbital Cortex | 3.33 | -42 | 32 | -8 |
| 16 |  |  |  |  |  |
|  | R Middle Temporal Gyrus, posterior division | 3.34 | 54 | -24 | -6 |
| 16 |  |  |  |  |  |
|  | L Hippocampus | 3.23 | -24 | -40 | 0 |
| 16 |  |  |  |  |  |
|  | R Hippocampus | 3.42 | 18 | -38 | 2 |
| 16 |  |  |  |  |  |
|  | R Putamen | 3.18 | 30 | -14 | 0 |
| 16 |  |  |  |  |  |
|  | L Central Opercular Cortex | 3.39 | -54 | -4 | 8 |
| 16 |  |  |  |  |  |
|  | L Planum Temporale | 3.42 | -64 | -28 | 10 |
| 16 |  |  |  |  |  |
|  | R Planum Temporale | 3.02 | 56 | -28 | 12 |
| 16 |  |  |  |  |  |
|  | R Precentral Gyrus | 3.26 | 64 | -2 | 12 |
| 16 |  |  |  |  |  |
|  | L Frontal Pole | 3.04 | -44 | 52 | 14 |
| 16 |  |  |  |  |  |
|  | R Frontal Pole | 3.15 | 40 | 60 | 12 |
| 16 |  |  |  |  |  |
|  | R Postcentral Gyrus | 3.53 | 66 | -8 | 18 |
| 16 |  |  |  |  |  |
|  | L Lateral Occipital Cortex, superior division | 3.10 | -40 | -82 | 18 |
| 16 |  |  |  |  |  |
|  | L Angular Gyrus | 3.24 | -40 | -54 | 20 |
| 16 |  |  |  |  |  |
|  | R Inferior Frontal Gyrus, pars opercularis | 3.27 | 58 | 12 | 20 |
| 16 |  |  |  |  |  |
|  | L Middle Frontal Gyrus | 3.44 | -42 | 22 | 24 |
| 16 |  |  |  |  |  |
|  | R Cingulate Gyrus, anterior division | 3.70 | 4 | 24 | 26 |
| 16 |  |  |  |  |  |
|  | R Frontal Pole | 3.02 | 46 | 42 | 28 |
| 16 |  |  |  |  |  |
|  | R Paracingulate Gyrus | 3.28 | 6 | 32 | 30 |
| 16 |  |  |  |  |  |
|  | R Middle Frontal Gyrus | 3.09 | 46 | 22 | 32 |
| 16 |  |  |  |  |  |
|  | R Paracingulate Gyrus | 3.33 | 8 | 42 | 34 |
| 16 |  |  |  |  |  |
|  | L Paracingulate Gyrus | 3.15 | -2 | 22 | 36 |
| 16 |  |  |  |  |  |
|  | R Paracingulate Gyrus | 3.25 | 2 | 28 | 42 |
| 16 |  |  |  |  |  |
|  | L Middle Frontal Gyrus | 3.13 | -40 | 22 | 42 |
| 16 |  |  |  |  |  |
|  | L Precuneus Cortex | 3.09 | -12 | -42 | 44 |
| 8 |  |  |  |  |  |
|  | R Brain-Stem | 3.21 | 2 | -28 | -46 |
| 8 |  |  |  |  |  |
|  | R Brain-Stem | 3.23 | 6 | -42 | -44 |
| 8 |  |  |  |  |  |
|  | R Brain-Stem | 3.21 | 12 | -38 | -40 |
| 8 |  |  |  |  |  |
|  | R Brain-Stem | 3.09 | 0 | -20 | -38 |
| 8 |  |  |  |  |  |
|  | L Temporal Pole | 3.10 | -22 | 16 | -38 |
| 8 |  |  |  |  |  |
|  | R Temporal Pole | 3.12 | 48 | 6 | -34 |
| 8 |  |  |  |  |  |
|  | R Brain-Stem | 3.15 | 8 | -34 | -24 |
| 8 |  |  |  |  |  |
|  | L Temporal Pole | 3.08 | -52 | 8 | -22 |
| 8 |  |  |  |  |  |
|  | R Amygdala | 2.96 | 26 | -4 | -20 |
|  | R Amygdala | 2.96 | 26 | -4 | -20 |
| 8 |  |  |  |  |  |
|  | R Middle Temporal Gyrus, anterior division | 3.07 | 58 | 2 | -20 |
| 8 |  |  |  |  |  |
|  | R Parahippocampal Gyrus, posterior division | 2.98 | 22 | -36 | -18 |
| 8 |  |  |  |  |  |
|  | R Planum Polare | 3.11 | 42 | 4 | -18 |
| 8 |  |  |  |  |  |
|  | R Inferior Temporal Gyrus, temporooccipital part | 3.25 | 64 | -40 | -16 |
| 8 |  |  |  |  |  |
|  | L Temporal Pole | 3.03 | -50 | 16 | -16 |
| 8 |  |  |  |  |  |
|  | R Frontal Orbital Cortex | 3.07 | 42 | 20 | -16 |
| 8 |  |  |  |  |  |
|  | L Frontal Pole | 2.99 | -24 | 42 | -14 |
| 8 |  |  |  |  |  |
|  | L Amygdala | 3.10 | -18 | -6 | -12 |
|  | L Amygdala | 3.10 | -18 | -6 | -12 |
| 8 |  |  |  |  |  |
|  | R Temporal Pole | 3.03 | 56 | 10 | -10 |
| 8 |  |  |  |  |  |
|  | R Hippocampus | 3.02 | 26 | -32 | -6 |
| 8 |  |  |  |  |  |
|  | R Middle Temporal Gyrus, posterior division | 3.08 | 50 | -26 | -6 |
| 8 |  |  |  |  |  |
|  | L Superior Temporal Gyrus, anterior division | 3.09 | -56 | -6 | -6 |
| 8 |  |  |  |  |  |
|  | R Insular Cortex | 3.09 | 42 | 6 | -6 |
| 8 |  |  |  |  |  |
|  | R Frontal Orbital Cortex | 2.98 | 48 | 28 | -6 |
| 8 |  |  |  |  |  |
|  | L Paracingulate Gyrus | 3.38 | -6 | 46 | -4 |
| 8 |  |  |  |  |  |
|  | R Frontal Pole | 2.96 | 26 | 60 | -4 |
| 8 |  |  |  |  |  |
|  | L Superior Temporal Gyrus, posterior division | 3.39 | -62 | -22 | -2 |
| 8 |  |  |  |  |  |
|  | L Precentral Gyrus | 3.47 | -56 | 6 | 2 |
| 8 |  |  |  |  |  |
|  | L Thalamus | 2.97 | -20 | -26 | 6 |
| 8 |  |  |  |  |  |
|  | R Insular Cortex | 2.96 | 34 | -24 | 8 |
| 8 |  |  |  |  |  |
|  | R Central Opercular Cortex | 3.46 | 56 | -2 | 8 |
| 8 |  |  |  |  |  |
|  | R Frontal Pole | 3.37 | 10 | 64 | 8 |
| 8 |  |  |  |  |  |
|  | L Planum Temporale | 3.42 | -50 | -30 | 10 |
| 8 |  |  |  |  |  |
|  | R Precentral Gyrus | 3.16 | 60 | 2 | 10 |
| 8 |  |  |  |  |  |
|  | L Precentral Gyrus | 3.00 | -60 | 10 | 10 |
| 8 |  |  |  |  |  |
|  | L Central Opercular Cortex | 2.98 | -46 | -18 | 12 |
| 8 |  |  |  |  |  |
|  | R Central Opercular Cortex | 2.99 | 50 | -14 | 12 |
| 8 |  |  |  |  |  |
|  | R Caudate | 3.10 | 16 | 10 | 12 |
| 8 |  |  |  |  |  |
|  | L Inferior Frontal Gyrus, pars opercularis | 3.16 | -54 | 10 | 12 |
| 8 |  |  |  |  |  |
|  | R Frontal Pole | 3.02 | 6 | 66 | 12 |
| 8 |  |  |  |  |  |
|  | L Planum Temporale | 3.33 | -36 | -34 | 14 |
| 8 |  |  |  |  |  |
|  | L Inferior Frontal Gyrus, pars triangularis | 3.04 | -48 | 34 | 14 |
| 8 |  |  |  |  |  |
|  | L Parietal Operculum Cortex | 2.97 | -44 | -26 | 16 |
| 8 |  |  |  |  |  |
|  | L Frontal Pole | 3.20 | -48 | 42 | 16 |
| 8 |  |  |  |  |  |
|  | R Caudate | 3.26 | 16 | -4 | 18 |
| 8 |  |  |  |  |  |
|  | R Parietal Operculum Cortex | 2.97 | 40 | -30 | 20 |
| 8 |  |  |  |  |  |
|  | L Precentral Gyrus | 3.43 | -58 | 0 | 24 |
| 8 |  |  |  |  |  |
|  | L Cingulate Gyrus, anterior division | 3.13 | -2 | 10 | 26 |
| 8 |  |  |  |  |  |
|  | R Postcentral Gyrus | 3.00 | 64 | -18 | 30 |
| 8 |  |  |  |  |  |
|  | R Precentral Gyrus | 3.03 | 62 | 6 | 30 |
| 8 |  |  |  |  |  |
|  | R Superior Frontal Gyrus | 3.03 | 6 | 50 | 30 |
| 8 |  |  |  |  |  |
|  | L Frontal Pole | 2.97 | -14 | 52 | 30 |
| 8 |  |  |  |  |  |
|  | R Lateral Occipital Cortex, superior division | 3.14 | 24 | -82 | 34 |
| 8 |  |  |  |  |  |
|  | L Precuneus Cortex | 2.96 | -14 | -76 | 34 |
| 8 |  |  |  |  |  |
|  | L Cingulate Gyrus, anterior division | 2.96 | -8 | 12 | 34 |
| 8 |  |  |  |  |  |
|  | L Supramarginal Gyrus, anterior division | 3.04 | -60 | -28 | 36 |
| 8 |  |  |  |  |  |
|  | R Paracingulate Gyrus | 3.15 | 0 | 38 | 36 |
| 8 |  |  |  |  |  |
|  | L Middle Frontal Gyrus | 2.97 | -30 | 26 | 38 |
| 8 |  |  |  |  |  |
|  | L Supramarginal Gyrus, posterior division | 3.02 | -60 | -42 | 40 |
| 8 |  |  |  |  |  |
|  | L Supramarginal Gyrus, anterior division | 2.97 | -62 | -38 | 40 |
| 8 |  |  |  |  |  |
|  | L Precuneus Cortex | 2.99 | -6 | -50 | 42 |
| 8 |  |  |  |  |  |
|  | L Supramarginal Gyrus, posterior division | 3.40 | -50 | -48 | 42 |
| 8 |  |  |  |  |  |
|  | L Middle Frontal Gyrus | 3.22 | -40 | 20 | 50 |
| 8 |  |  |  |  |  |
|  | R Frontal Pole | 3.13 | 12 | 44 | 50 |
| 8 |  |  |  |  |  |
|  | R Frontal Pole | 2.98 | 12 | 40 | 52 |
| 8 |  |  |  |  |  |
|  | L Precuneus Cortex | 3.18 | -10 | -50 | 54 |
| 8 |  |  |  |  |  |
|  | R Postcentral Gyrus | 3.09 | 22 | -38 | 58 |
| 8 |  |  |  |  |  |
|  | L Precentral Gyrus | 3.00 | -36 | -12 | 60 |
| 8 |  |  |  |  |  |
|  | L Postcentral Gyrus | 3.19 | -20 | -46 | 66 |
| 8 |  |  |  |  |  |
|  | R Superior Parietal Lobule | 3.27 | 22 | -42 | 66 |
| 8 |  |  |  |  |  |
|  | L Precentral Gyrus | 3.37 | -6 | -16 | 72 |

# Supplementary Table S15. [CT_phasic _> UT_phasic_]. Descriptive statistics for clusters and local maxima showing greater activation for Certain-Threat relative to Uncertain-Threat anticipation for the OSP Phasic regressor (FDR *q*<0.05, whole-brain corrected). 

| ***mm***^***3***^ | **Label** | ***t*** | ***x*** | ***y*** | ***z*** |
| --- | --- | --- | --- | --- | --- |
| 73,416 |  |  |  |  |  |
|  | R Frontal Pole | 4.02 | 2 | 58 | -2 |
|  | L Paracingulate Gyrus | 7.53 | -8 | 22 | 32 |
|  | L Insular Cortex | 6.46 | -32 | 20 | 8 |
|  | R Paracingulate Gyrus | 5.26 | 10 | 20 | 34 |
|  | L Frontal Operculum Cortex | 6.72 | -34 | 16 | 8 |
|  | L Cingulate Gyrus, anterior division | 7.97 | -8 | 14 | 36 |
|  | L Inferior Frontal Gyrus, pars opercularis | 4.60 | -58 | 10 | 4 |
|  | R Cingulate Gyrus, anterior division | 9.81 | 10 | 10 | 40 |
|  | L Central Opercular Cortex | 3.07 | -52 | 2 | 4 |
|  | R Juxtapositional Lobule Cortex (formerly Supplementary Motor Cortex) | 9.66 | 0 | 0 |  |
|  | L Middle Frontal Gyrus | 4.86 | -38 | -2 | 56 |
|  | L Precentral Gyrus | 7.27 | -48 | -4 | 54 |
|  | L Juxtapositional Lobule Cortex (formerly Supplementary Motor Cortex) | 9.77 | -2 | -6 | 64 |
|  | L Superior Frontal Gyrus | 8.30 | -14 | -6 | 68 |
|  | R Superior Frontal Gyrus | 7.18 | 16 | -10 | 72 |
|  | R Precentral Gyrus | 8.80 | 32 | -26 | 52 |
|  | R Postcentral Gyrus | 7.75 | 36 | -26 | 62 |
|  | R Cingulate Gyrus, posterior division | 5.26 | 4 | -32 | 26 |
|  | L Cingulate Gyrus, posterior division | 5.75 | -4 | -36 | 26 |
|  | R Superior Parietal Lobule | 5.29 | 26 | -42 | 62 |
|  | R Lateral Occipital Cortex, superior division | 4.34 | 32 | -62 | 64 |
| 27,752 |  |  |  |  |  |
|  | R Frontal Pole | 4.05 | 12 | 34 | -20 |
|  | R Frontal Orbital Cortex | 5.09 | 22 | 28 | -20 |
|  | L Accumbens | 5.22 | -8 | 18 | -2 |
|  | R Subcallosal Cortex | 3.82 | 12 | 18 | -16 |
|  | L Subcallosal Cortex | 3.63 | -6 | 16 | -14 |
|  | L Putamen | 7.48 | -16 | 12 | -12 |
|  | R Putamen | 8.32 | 20 | 12 | 0 |
|  | L Frontal Orbital Cortex | 7.17 | -18 | 10 | -16 |
|  | R Accumbens | 7.92 | 6 | 10 | -4 |
|  | R Caudate | 10.69 | 8 | 6 | 2 |
|  | L Caudate | 9.46 | -8 | 4 | 4 |
|  | R Amygdala | 4.47 | 16 | 0 | -20 |
|  | R Thalamus | 6.30 | 8 | -2 | 6 |
|  | R Amygdala | 4.17 | 12 | -4 | -18 |
|  | R Amygdala | 2.81 | 18 | -4 | -24 |
|  | R Amygdala | 4.50 | 28 | -8 | -12 |
|  | R Amygdala | 4.20 | 30 | -8 | -18 |
|  | L Amygdala | 5.54 | -28 | -10 | -16 |
|  | L Hippocampus | 5.54 | -22 | -14 | -18 |
|  | L Amygdala | 5.46 | -20 | -16 | -14 |
|  | L Thalamus | 5.44 | -2 | -16 | 12 |
|  | R Hippocampus | 4.58 | 22 | -20 | -16 |
| 26,104 |  |  |  |  |  |
|  | R Middle Temporal Gyrus, temporooccipital part | 3.95 | 54 | -52 | -6 |
|  | R Temporal Occipital Fusiform Cortex | 2.91 | 40 | -52 | -16 |
|  | R Lingual Gyrus | 2.75 | 12 | -70 | -12 |
|  | R Occipital Fusiform Gyrus | 3.19 | 20 | -84 | -6 |
|  | R Lateral Occipital Cortex, inferior division | 9.84 | 32 | -88 | -2 |
|  | R Occipital Pole | 13.54 | 24 | -98 | 0 |
| 7,992 |  |  |  |  |  |
|  | L Postcentral Gyrus | 3.68 | -48 | -30 | 46 |
|  | L Supramarginal Gyrus, anterior division | 5.73 | -42 | -38 | 40 |
|  | L Supramarginal Gyrus, posterior division | 4.64 | -58 | -46 | 50 |
|  | L Superior Parietal Lobule | 5.44 | -34 | -54 | 50 |
|  | L Angular Gyrus | 3.09 | -42 | -58 | 46 |
|  | L Lateral Occipital Cortex, superior division | 4.00 | -30 | -62 | 40 |
| 6,144 |  |  |  |  |  |
|  | L Frontal Pole | 6.33 | -36 | 48 | 30 |
|  | L Middle Frontal Gyrus | 5.58 | -38 | 36 | 40 |
| 5,992 |  |  |  |  |  |
|  | L Lateral Occipital Cortex, inferior division | 4.64 | -40 | -72 | 2 |
|  | L Occipital Pole | 7.77 | -26 | -96 | -6 |
| 3,856 |  |  |  |  |  |
|  | L Frontal Pole | 4.61 | -8 | 68 | -16 |
|  | R Frontal Pole | 4.83 | 2 | 66 | -10 |
| 3,448 |  |  |  |  |  |
|  | R Precuneus Cortex | 3.90 | 4 | -60 | 44 |
|  | L Precuneus Cortex | 6.75 | -10 | -70 | 40 |
| 2,664 |  |  |  |  |  |
|  | R Precuneus Cortex | 6.46 | 14 | -64 | 34 |
|  | R Lateral Occipital Cortex, superior division | 3.55 | 12 | -74 | 52 |
| 2,248 |  |  |  |  |  |
|  | R Frontal Orbital Cortex | 4.93 | 34 | 28 | 2 |
|  | R Frontal Operculum Cortex | 6.10 | 36 | 20 | 8 |
|  | R Central Opercular Cortex | 3.11 | 48 | 4 | 2 |
| 1,880 |  |  |  |  |  |
|  | R Frontal Pole | 5.08 | 30 | 38 | 36 |
| 1,384 |  |  |  |  |  |
|  | R Temporal Pole | 4.27 | 42 | 6 | -40 |
|  | R Inferior Temporal Gyrus, anterior division | 4.14 | 52 | -4 | -34 |
| 1,136 |  |  |  |  |  |
|  | L Frontal Pole | 4.30 | -26 | 40 | -18 |
|  | L Frontal Orbital Cortex | 6.91 | -24 | 28 | -22 |
| 888 |  |  |  |  |  |
|  | L Temporal Pole | 4.01 | -46 | 6 | -40 |
|  | L Inferior Temporal Gyrus, anterior division | 5.33 | -46 | 0 | -38 |
|  | L Temporal Fusiform Cortex, anterior division | 3.29 | -38 | -2 | -40 |
| 824 |  |  |  |  |  |
|  | R Angular Gyrus | 4.13 | 56 | -46 | 26 |
| 592 |  |  |  |  |  |
|  | R Precentral Gyrus | 4.24 | 60 | 4 | 14 |
| 576 |  |  |  |  |  |
|  | R Brain-Stem | 3.27 | 8 | -48 | -54 |
| 392 |  |  |  |  |  |
|  | L Brain-Stem | 4.11 | -8 | -46 | -52 |
| 344 |  |  |  |  |  |
|  | R Precuneus Cortex | 4.60 | 2 | -44 | 42 |
|  | R Cingulate Gyrus, posterior division | 3.13 | 4 | -44 | 36 |
| 304 |  |  |  |  |  |
|  | R Temporal Pole | 4.80 | 38 | 18 | -32 |
| 296 |  |  |  |  |  |
|  | L Brain-Stem | 4.45 | -10 | -24 | -42 |
| 288 |  |  |  |  |  |
|  | L Cingulate Gyrus, posterior division | 4.57 | -12 | -28 | 40 |
| 280 |  |  |  |  |  |
|  | R Central Opercular Cortex | 3.49 | 50 | -18 | 18 |
|  | R Parietal Operculum Cortex | 4.15 | 46 | -22 | 18 |
| 272 |  |  |  |  |  |
|  | L Hippocampus | 4.27 | -22 | -38 | 0 |
| 240 |  |  |  |  |  |
|  | L Lateral Occipital Cortex, superior division | 3.80 | -28 | -80 | 50 |
| 216 |  |  |  |  |  |
|  | R Insular Cortex | 4.14 | 36 | 16 | -10 |
| 192 |  |  |  |  |  |
|  | R Lateral Occipital Cortex, superior division | 3.37 | 26 | -64 | 42 |
| 192 |  |  |  |  |  |
|  | L Precentral Gyrus | 3.44 | -16 | -30 | 68 |
| 168 |  |  |  |  |  |
|  | L Postcentral Gyrus | 3.77 | -62 | -22 | 28 |
| 152 |  |  |  |  |  |
|  | R Brain-Stem | 3.26 | 0 | -36 | -40 |
| 144 |  |  |  |  |  |
|  | R Temporal Fusiform Cortex, anterior division | 3.28 | 32 | -8 | -42 |
|  | R Temporal Fusiform Cortex, posterior division | 3.04 | 32 | -10 | -38 |
| 144 |  |  |  |  |  |
|  | R Brain-Stem | 3.03 | 14 | -20 | -38 |
| 144 |  |  |  |  |  |
|  | R Brain-Stem | 3.74 | 8 | -24 | -22 |
| 136 |  |  |  |  |  |
|  | L Hippocampus | 3.81 | -32 | -18 | -14 |
| 128 |  |  |  |  |  |
|  | R Temporal Pole | 3.03 | 26 | 6 | -34 |
|  | R Parahippocampal Gyrus, anterior division | 3.37 | 26 | 2 | -38 |
| 128 |  |  |  |  |  |
|  | R Lateral Occipital Cortex, superior division | 3.20 | 30 | -70 | 26 |
| 120 |  |  |  |  |  |
|  | L Temporal Pole | 3.45 | -32 | 4 | -22 |
|  | L Amygdala | 4.22 | -32 | -2 | -20 |
| 120 |  |  |  |  |  |
|  | R Supramarginal Gyrus, posterior division | 2.77 | 66 | -40 | 34 |
| 112 |  |  |  |  |  |
|  | L Frontal Pole | 3.37 | -16 | 50 | -20 |
| 104 |  |  |  |  |  |
|  | R Hippocampus | 4.24 | 26 | -36 | 4 |
| 96 |  |  |  |  |  |
|  | L Bed Nucleus of the Stria Terminalis | 9.84 | -6 | 4 | 0 |
|  | R Brain-Stem | 3.18 | 4 | -32 | -48 |
| 96 |  |  |  |  |  |
|  | L Brain-Stem | 2.87 | -2 | -32 | -28 |
|  | R Brain-Stem | 3.29 | 0 | -38 | -28 |
| 96 |  |  |  |  |  |
|  | L Lateral Occipital Cortex, superior division | 3.00 | -26 | -70 | 60 |
| 88 |  |  |  |  |  |
|  | R Temporal Fusiform Cortex, posterior division | 3.48 | 34 | -14 | -34 |
| 88 |  |  |  |  |  |
|  | L Brain-Stem | 3.51 | -4 | -38 | -22 |
| 88 |  |  |  |  |  |
|  | R Middle Temporal Gyrus, posterior division | 3.77 | 64 | -20 | -14 |
| 88 |  |  |  |  |  |
|  | L Insular Cortex | 3.21 | -32 | 18 | -8 |
| 88 |  |  |  |  |  |
|  | L Frontal Pole | 2.80 | -10 | 58 | 4 |
|  | L Paracingulate Gyrus | 3.30 | -8 | 54 | 2 |
| 88 |  |  |  |  |  |
|  | L Supramarginal Gyrus, posterior division | 3.79 | -50 | -50 | 38 |
|  | L Angular Gyrus | 2.69 | -48 | -52 | 34 |
| 80 |  |  |  |  |  |
|  | R Brain-Stem | 3.85 | 8 | -24 | -34 |
| 80 |  |  |  |  |  |
|  | L Frontal Pole | 3.38 | -26 | 64 | -12 |
| 80 |  |  |  |  |  |
|  | R Paracingulate Gyrus | 3.14 | 2 | 36 | -10 |
|  | R Frontal Medial Cortex | 3.08 | 8 | 36 | -12 |
| 72 |  |  |  |  |  |
|  | L Inferior Temporal Gyrus, anterior division | 2.97 | -52 | -8 | -34 |
| 72 |  |  |  |  |  |
|  | L Middle Temporal Gyrus, posterior division | 2.69 | -66 | -22 | -20 |
| 72 |  |  |  |  |  |
|  | L Paracingulate Gyrus | 3.25 | -6 | 46 | 6 |
| 72 |  |  |  |  |  |
|  | L Frontal Pole | 3.33 | -32 | 62 | 16 |
| 72 |  |  |  |  |  |
|  | L Lateral Occipital Cortex, superior division | 3.18 | -36 | -66 | 56 |
| 64 |  |  |  |  |  |
|  | R Temporal Pole | 2.88 | 36 | 14 | -22 |
| 64 |  |  |  |  |  |
|  | L Middle Temporal Gyrus, posterior division | 3.36 | -66 | -32 | -20 |
| 64 |  |  |  |  |  |
|  | L Hippocampus | 3.31 | -24 | -26 | -12 |
| 64 |  |  |  |  |  |
|  | R Lateral Occipital Cortex, superior division | 3.51 | 46 | -74 | 42 |
| 56 |  |  |  |  |  |
|  | R Parahippocampal Gyrus, anterior division | 3.46 | 22 | -10 | -32 |
| 56 |  |  |  |  |  |
|  | L Cingulate Gyrus, anterior division | 3.58 | -4 | 40 | -4 |
| 56 |  |  |  |  |  |
|  | L Pallidum | 3.28 | -20 | -10 | 0 |
| 56 |  |  |  |  |  |
|  | R Angular Gyrus | 2.95 | 62 | -50 | 42 |
| 56 |  |  |  |  |  |
|  | L Precentral Gyrus | 3.51 | -10 | -18 | 42 |
| 48 |  |  |  |  |  |
|  | L Brain-Stem | 3.01 | -6 | -20 | -28 |
| 48 |  |  |  |  |  |
|  | L Frontal Orbital Cortex | 3.00 | -22 | 18 | -26 |
| 48 |  |  |  |  |  |
|  | R Brain-Stem | 3.16 | 2 | -18 | -20 |
|  | L Brain-Stem | 2.84 | -4 | -22 | -18 |
| 48 |  |  |  |  |  |
|  | L Middle Temporal Gyrus, posterior division | 3.43 | -70 | -20 | -14 |
| 48 |  |  |  |  |  |
|  | L Lateral Occipital Cortex, inferior division | 3.15 | -46 | -70 | -8 |
| 48 |  |  |  |  |  |
|  | R Middle Temporal Gyrus, posterior division | 3.32 | 62 | -32 | -6 |
| 48 |  |  |  |  |  |
|  | L Lateral Occipital Cortex, superior division | 3.54 | -36 | -82 | 44 |
| 40 |  |  |  |  |  |
|  | L Parahippocampal Gyrus, anterior division | 3.05 | -26 | 0 | -32 |
| 40 |  |  |  |  |  |
|  | R Hippocampus | 3.50 | 32 | -8 | -26 |
| 40 |  |  |  |  |  |
|  | L Inferior Temporal Gyrus, temporooccipital part | 3.01 | -46 | -58 | -6 |
| 40 |  |  |  |  |  |
|  | R Precuneus Cortex | 2.73 | 6 | -56 | 24 |
| 40 |  |  |  |  |  |
|  | R Frontal Pole | 3.37 | 38 | 52 | 26 |
| 40 |  |  |  |  |  |
|  | R Lateral Occipital Cortex, superior division | 2.89 | 52 | -68 | 40 |
| 40 |  |  |  |  |  |
|  | R Angular Gyrus | 2.84 | 50 | -52 | 40 |
| 40 |  |  |  |  |  |
|  | L Precuneus Cortex | 3.09 | -8 | -54 | 58 |
| 32 |  |  |  |  |  |
|  | R Brain-Stem | 3.17 | 8 | -40 | -52 |
| 32 |  |  |  |  |  |
|  | L Temporal Pole | 3.42 | -38 | 12 | -36 |
| 32 |  |  |  |  |  |
|  | L Brain-Stem | 2.56 | -18 | -24 | -34 |
| 32 |  |  |  |  |  |
|  | L Frontal Orbital Cortex | 3.30 | -14 | 32 | -22 |
| 32 |  |  |  |  |  |
|  | L Frontal Pole | 3.00 | -14 | 38 | -24 |
| 32 |  |  |  |  |  |
|  | L Middle Temporal Gyrus, posterior division | 3.19 | -60 | -26 | -12 |
| 32 |  |  |  |  |  |
|  | R Middle Temporal Gyrus, posterior division | 2.97 | 66 | -16 | -8 |
| 32 |  |  |  |  |  |
|  | L Thalamus | 2.77 | -12 | -32 | 8 |
| 32 |  |  |  |  |  |
|  | R Paracingulate Gyrus | 2.71 | 14 | 48 | 8 |
| 32 |  |  |  |  |  |
|  | R Caudate | 3.18 | 16 | -14 | 22 |
| 32 |  |  |  |  |  |
|  | R Frontal Pole | 2.67 | 28 | 42 | 22 |
| 32 |  |  |  |  |  |
|  | L Middle Frontal Gyrus | 2.69 | -44 | 20 | 36 |
| 32 |  |  |  |  |  |
|  | R Precuneus Cortex | 2.65 | 8 | -56 | 52 |
| 32 |  |  |  |  |  |
|  | R Middle Frontal Gyrus | 2.69 | 32 | 2 | 54 |
| 32 |  |  |  |  |  |
|  | L Lateral Occipital Cortex, superior division | 2.84 | -18 | -68 | 66 |
| 24 |  |  |  |  |  |
|  | L Temporal Pole | 2.64 | -40 | 8 | -44 |
| 24 |  |  |  |  |  |
|  | L Temporal Pole | 2.59 | -34 | 10 | -30 |
| 24 |  |  |  |  |  |
|  | R Inferior Temporal Gyrus, posterior division | 2.97 | 56 | -18 | -26 |
| 24 |  |  |  |  |  |
|  | R Temporal Fusiform Cortex, posterior division | 2.76 | 38 | -18 | -26 |
| 24 |  |  |  |  |  |
|  | R Subcallosal Cortex | 3.23 | 2 | 20 | -18 |
| 24 |  |  |  |  |  |
|  | R Middle Temporal Gyrus, posterior division | 2.74 | 62 | -34 | -14 |
| 24 |  |  |  |  |  |
|  | R Temporal Pole | 2.79 | 52 | 10 | -14 |
| 24 |  |  |  |  |  |
|  | L Frontal Pole | 2.79 | -22 | 52 | -14 |
| 24 |  |  |  |  |  |
|  | R Hippocampus | 3.22 | 36 | -30 | -12 |
| 24 |  |  |  |  |  |
|  | R Thalamus | 2.97 | 14 | -36 | 4 |
| 24 |  |  |  |  |  |
|  | R Thalamus | 2.58 | 6 | -16 | 6 |
| 24 |  |  |  |  |  |
|  | R Paracingulate Gyrus | 2.72 | 4 | 50 | 12 |
| 24 |  |  |  |  |  |
|  | L Parietal Operculum Cortex | 3.00 | -42 | -30 | 22 |
| 24 |  |  |  |  |  |
|  | L Parietal Operculum Cortex | 2.61 | -44 | -34 | 24 |
| 24 |  |  |  |  |  |
|  | R Precentral Gyrus | 2.77 | 46 | 6 | 30 |
| 24 |  |  |  |  |  |
|  | R Superior Frontal Gyrus | 2.98 | 6 | 42 | 38 |
| 24 |  |  |  |  |  |
|  | R Cingulate Gyrus, posterior division | 2.82 | 10 | -38 | 42 |
| 24 |  |  |  |  |  |
|  | L Lateral Occipital Cortex, superior division | 2.63 | -12 | -76 | 58 |
| 24 |  |  |  |  |  |
|  | R Lateral Occipital Cortex, superior division | 2.71 | 16 | -66 | 64 |
| 24 |  |  |  |  |  |
|  | L Lateral Occipital Cortex, superior division | 2.75 | -14 | -64 | 64 |
| 16 |  |  |  |  |  |
|  | R Brain-Stem | 2.66 | 12 | -24 | -42 |
| 16 |  |  |  |  |  |
|  | R Inferior Temporal Gyrus, posterior division | 2.68 | 60 | -16 | -38 |
| 16 |  |  |  |  |  |
|  | L Inferior Temporal Gyrus, posterior division | 2.61 | -48 | -16 | -38 |
| 16 |  |  |  |  |  |
|  | L Temporal Fusiform Cortex, posterior division | 2.81 | -34 | -18 | -36 |
| 16 |  |  |  |  |  |
|  | L Temporal Pole | 2.88 | -36 | 6 | -36 |
| 16 |  |  |  |  |  |
|  | L Temporal Pole | 2.72 | -52 | 4 | -34 |
| 16 |  |  |  |  |  |
|  | L Temporal Pole | 2.70 | -50 | 14 | -34 |
| 16 |  |  |  |  |  |
|  | L Temporal Fusiform Cortex, posterior division | 2.66 | -36 | -34 | -28 |
| 16 |  |  |  |  |  |
|  | L Inferior Temporal Gyrus, posterior division | 2.79 | -50 | -34 | -28 |
| 16 |  |  |  |  |  |
|  | L Amygdala | 3.25 | -14 | -2 | -20 |
| 16 |  |  |  |  |  |
|  | R Frontal Pole | 2.76 | 0 | 54 | -20 |
| 16 |  |  |  |  |  |
|  | L Frontal Pole | 2.77 | -32 | 58 | -14 |
| 16 |  |  |  |  |  |
|  | R Frontal Medial Cortex | 2.87 | 2 | 50 | -12 |
| 16 |  |  |  |  |  |
|  | L Parahippocampal Gyrus, posterior division | 3.33 | -14 | -32 | -10 |
| 16 |  |  |  |  |  |
|  | L Hippocampus | 3.25 | -28 | -38 | -8 |
| 16 |  |  |  |  |  |
|  | R Insular Cortex | 2.87 | 36 | 4 | -8 |
| 16 |  |  |  |  |  |
|  | L Frontal Orbital Cortex | 3.25 | -28 | 28 | -4 |
| 16 |  |  |  |  |  |
|  | R Hippocampus | 3.19 | 26 | -38 | -2 |
| 16 |  |  |  |  |  |
|  | R Thalamus | 2.77 | 4 | -22 | -2 |
| 16 |  |  |  |  |  |
|  | R Thalamus | 2.60 | 6 | -30 | 4 |
| 16 |  |  |  |  |  |
|  | L Lateral Occipital Cortex, inferior division | 2.77 | -42 | -66 | 6 |
| 16 |  |  |  |  |  |
|  | L Insular Cortex | 2.68 | -36 | -16 | 10 |
| 16 |  |  |  |  |  |
|  | R Thalamus | 3.17 | 20 | -20 | 14 |
| 16 |  |  |  |  |  |
|  | R Cingulate Gyrus, anterior division | 2.75 | 10 | 38 | 16 |
| 16 |  |  |  |  |  |
|  | R Angular Gyrus | 2.97 | 44 | -48 | 20 |
| 16 |  |  |  |  |  |
|  | L Frontal Pole | 2.66 | -20 | 56 | 18 |
| 16 |  |  |  |  |  |
|  | L Caudate | 3.38 | -14 | -10 | 20 |
| 16 |  |  |  |  |  |
|  | L Caudate | 2.71 | -14 | -6 | 20 |
| 16 |  |  |  |  |  |
|  | L Frontal Pole | 2.86 | -26 | 46 | 20 |
| 16 |  |  |  |  |  |
|  | R Precentral Gyrus | 2.93 | 52 | 4 | 22 |
| 16 |  |  |  |  |  |
|  | R Precentral Gyrus | 3.17 | 58 | 6 | 26 |
| 16 |  |  |  |  |  |
|  | L Supramarginal Gyrus, posterior division | 2.87 | -54 | -44 | 28 |
| 16 |  |  |  |  |  |
|  | R Precentral Gyrus | 2.70 | 42 | 2 | 30 |
| 16 |  |  |  |  |  |
|  | R Supramarginal Gyrus, posterior division | 2.73 | 58 | -40 | 38 |
| 16 |  |  |  |  |  |
|  | R Angular Gyrus | 2.56 | 44 | -48 | 42 |
| 16 |  |  |  |  |  |
|  | R Supramarginal Gyrus, anterior division | 3.10 | 54 | -28 | 40 |
| 16 |  |  |  |  |  |
|  | R Postcentral Gyrus | 2.87 | 54 | -10 | 50 |
| 16 |  |  |  |  |  |
|  | L Superior Parietal Lobule | 2.87 | -36 | -42 | 60 |
| 16 |  |  |  |  |  |
|  | R Lateral Occipital Cortex, superior division | 2.66 | 16 | -74 | 60 |
| 16 |  |  |  |  |  |
|  | L Lateral Occipital Cortex, superior division | 2.83 | -10 | -66 | 64 |
| 8 |  |  |  |  |  |
|  | L Brain-Stem | 2.50 | -2 | -36 | -48 |
| 8 |  |  |  |  |  |
|  | R Inferior Temporal Gyrus, anterior division | 2.48 | 50 | -2 | -46 |
| 8 |  |  |  |  |  |
|  | R Brain-Stem | 2.71 | 14 | -26 | -40 |
| 8 |  |  |  |  |  |
|  | L Temporal Pole | 2.64 | -46 | 12 | -40 |
| 8 |  |  |  |  |  |
|  | R Temporal Pole | 2.58 | 32 | 4 | -38 |
| 8 |  |  |  |  |  |
|  | L Brain-Stem | 2.55 | -12 | -30 | -30 |
| 8 |  |  |  |  |  |
|  | R Inferior Temporal Gyrus, posterior division | 2.58 | 54 | -12 | -30 |
| 8 |  |  |  |  |  |
|  | R Temporal Pole | 2.80 | 28 | 10 | -30 |
| 8 |  |  |  |  |  |
|  | L Brain-Stem | 2.88 | -16 | -22 | -28 |
| 8 |  |  |  |  |  |
|  | L Inferior Temporal Gyrus, posterior division | 2.59 | -52 | -36 | -26 |
| 8 |  |  |  |  |  |
|  | R Temporal Pole | 2.76 | 32 | 8 | -26 |
| 8 |  |  |  |  |  |
|  | R Brain-Stem | 2.61 | 12 | -30 | -22 |
| 8 |  |  |  |  |  |
|  | L Temporal Fusiform Cortex, posterior division | 3.24 | -38 | -24 | -20 |
| 8 |  |  |  |  |  |
|  | L Frontal Orbital Cortex | 2.58 | -14 | 20 | -20 |
| 8 |  |  |  |  |  |
|  | R Middle Temporal Gyrus, posterior division | 2.49 | 64 | -8 | -18 |
| 8 |  |  |  |  |  |
|  | R Frontal Orbital Cortex | 2.56 | 18 | 22 | -18 |
| 8 |  |  |  |  |  |
|  | R Occipital Fusiform Gyrus | 2.49 | 30 | -80 | -16 |
| 8 |  |  |  |  |  |
|  | R Temporal Occipital Fusiform Cortex | 2.49 | 26 | -58 | -16 |
| 8 |  |  |  |  |  |
|  | R Frontal Medial Cortex | 2.85 | 4 | 34 | -16 |
| 8 |  |  |  |  |  |
|  | L Frontal Pole | 2.57 | -30 | 50 | -14 |
| 8 |  |  |  |  |  |
|  | L Hippocampus | 2.61 | -34 | -32 | -12 |
| 8 |  |  |  |  |  |
|  | L Middle Temporal Gyrus, posterior division | 2.51 | -56 | -30 | -12 |
| 8 |  |  |  |  |  |
|  | L Subcallosal Cortex | 2.50 | -4 | 24 | -12 |
| 8 |  |  |  |  |  |
|  | R Subcallosal Cortex | 2.77 | 4 | 28 | -12 |
| 8 |  |  |  |  |  |
|  | R Frontal Pole | 2.60 | 24 | 66 | -12 |
| 8 |  |  |  |  |  |
|  | L Hippocampus | 2.48 | -32 | -28 | -10 |
| 8 |  |  |  |  |  |
|  | R Middle Temporal Gyrus, posterior division | 2.58 | 70 | -22 | -10 |
| 8 |  |  |  |  |  |
|  | L Subcallosal Cortex | 2.89 | -6 | 28 | -6 |
| 8 |  |  |  |  |  |
|  | R Middle Temporal Gyrus, posterior division | 2.55 | 56 | -36 | -4 |
| 8 |  |  |  |  |  |
|  | R Inferior Frontal Gyrus, pars triangularis | 2.69 | 50 | 20 | -4 |
| 8 |  |  |  |  |  |
|  | R Middle Temporal Gyrus, temporooccipital part | 2.55 | 60 | -46 | -2 |
| 8 |  |  |  |  |  |
|  | R Middle Temporal Gyrus, temporooccipital part | 2.53 | 54 | -48 | 4 |
| 8 |  |  |  |  |  |
|  | R Frontal Pole | 2.58 | 0 | 64 | 4 |
| 8 |  |  |  |  |  |
|  | L Frontal Pole | 2.56 | -28 | 66 | 6 |
| 8 |  |  |  |  |  |
|  | L Cingulate Gyrus, posterior division | 2.54 | -8 | -44 | 8 |
| 8 |  |  |  |  |  |
|  | L Central Opercular Cortex | 2.83 | -52 | 0 | 8 |
| 8 |  |  |  |  |  |
|  | R Cingulate Gyrus, posterior division | 2.56 | 8 | -44 | 10 |
| 8 |  |  |  |  |  |
|  | R Middle Temporal Gyrus, temporooccipital part | 2.87 | 48 | -48 | 14 |
| 8 |  |  |  |  |  |
|  | R Central Opercular Cortex | 2.50 | 46 | -4 | 14 |
| 8 |  |  |  |  |  |
|  | L Central Opercular Cortex | 2.67 | -42 | -4 | 14 |
| 8 |  |  |  |  |  |
|  | R Paracingulate Gyrus | 2.56 | 6 | 48 | 14 |
| 8 |  |  |  |  |  |
|  | R Lateral Occipital Cortex, superior division | 2.67 | 44 | -60 | 20 |
| 8 |  |  |  |  |  |
|  | R Paracingulate Gyrus | 2.62 | 10 | 40 | 20 |
| 8 |  |  |  |  |  |
|  | L Precuneus Cortex | 2.67 | -8 | -54 | 22 |
| 8 |  |  |  |  |  |
|  | R Inferior Frontal Gyrus, pars opercularis | 2.52 | 38 | 14 | 24 |
| 8 |  |  |  |  |  |
|  | L Supramarginal Gyrus, anterior division | 2.56 | -62 | -34 | 30 |
| 8 |  |  |  |  |  |
|  | R Precuneus Cortex | 2.67 | 12 | -54 | 36 |
| 8 |  |  |  |  |  |
|  | L Precuneus Cortex | 2.51 | -8 | -56 | 38 |
| 8 |  |  |  |  |  |
|  | R Angular Gyrus | 2.64 | 58 | -48 | 38 |
| 8 |  |  |  |  |  |
|  | L Precuneus Cortex | 3.37 | -10 | -60 | 40 |
| 8 |  |  |  |  |  |
|  | R Precuneus Cortex | 2.58 | 4 | -52 | 44 |
| 8 |  |  |  |  |  |
|  | R Precuneus Cortex | 2.49 | 8 | -40 | 46 |
| 8 |  |  |  |  |  |
|  | R Superior Frontal Gyrus | 2.72 | 6 | 36 | 50 |
| 8 |  |  |  |  |  |
|  | L Middle Frontal Gyrus | 2.69 | -30 | 6 | 56 |
| 8 |  |  |  |  |  |
|  | R Superior Frontal Gyrus | 2.68 | 22 | 10 | 58 |
| 8 |  |  |  |  |  |
|  | L Precentral Gyrus | 2.58 | -20 | -26 | 74 |

# Supplementary Table S16. [UT_sustained _> US_sustained_] $\boldsymbol{∩}$ [CT_phasic _> CS_phasic_]. Descriptive statistics for clusters and local maxima showing greater activation for Uncertain-Threat relative to Uncertain-Safety anticipation for the OSP Sustained regressor AND greater activation for Certain-Threat relative to Certain-Safety anticipation for the OSP Phasic regressor (FDR *q*<0.05, whole-brain corrected). 

| ***mm***^***3***^ | **Label** | ***t*** | ***x*** | ***y*** | ***z*** |
| --- | --- | --- | --- | --- | --- |
| 18,304 |  |  |  |  |  |
|  | L Left Caudate | 4.00 | -8 | 4 | 4 |
|  | R Right Thalamus | 4.22 | 8 | -2 | 6 |
|  | R Right Caudate | 5.32 | 12 | -4 | 16 |
|  | R Left Thalamus | 4.05 | 0 | -6 | 8 |
|  | L Left Thalamus | 3.79 | -2 | -14 | -2 |
|  | R Brain-Stem | 4.05 | 10 | -28 | -26 |
|  | L Brain-Stem | 6.24 | -2 | -30 | -2 |
|  | L Temporal Occipital Fusiform Cortex | 3.75 | -34 | -54 | -22 |
|  | L Occipital Fusiform Gyrus | 3.95 | -24 | -68 | -16 |
|  | L Lingual Gyrus | 3.62 | -10 | -76 | -14 |
| 15,736 |  |  |  |  |  |
|  | L Paracingulate Gyrus | 3.99 | -8 | 30 | 26 |
|  | L Cingulate Gyrus, anterior division | 5.79 | -8 | 14 | 36 |
|  | R Superior Frontal Gyrus | 3.60 | 4 | 14 | 60 |
|  | R Paracingulate Gyrus | 6.22 | 0 | 12 | 46 |
|  | R Cingulate Gyrus, anterior division | 7.24 | 10 | 8 | 40 |
|  | L Juxtapositional Lobule Cortex (formerly Supplementary Motor Cortex) | 5.74 | -6 | 4 | 46 |
|  | L Bed Nucleus of the Stria Terminalis | 4.24 | -6 | 4 | -2 |
|  | R Juxtapositional Lobule Cortex (formerly Supplementary Motor Cortex) | 6.89 | 4 | -2 | 50 |
|  | L Superior Frontal Gyrus | 5.59 | -12 | -8 | 66 |
| 13,920 |  |  |  |  |  |
|  | R Insular Cortex | 6.93 | 34 | 24 | 2 |
|  | R Frontal Orbital Cortex | 5.41 | 36 | 20 | -10 |
|  | R Frontal Operculum Cortex | 8.51 | 36 | 18 | 8 |
|  | R Inferior Frontal Gyrus, pars opercularis | 4.95 | 60 | 14 | 8 |
|  | R Right Putamen | 8.65 | 22 | 10 | -4 |
|  | R Central Opercular Cortex | 7.32 | 48 | 8 | -2 |
|  | R Precentral Gyrus | 4.58 | 60 | 4 | 14 |
|  | R Right Pallidum | 3.40 | 16 | 2 | -4 |
| 9,016 |  |  |  |  |  |
|  | L Frontal Orbital Cortex | 6.44 | -30 | 22 | -8 |
|  | L Inferior Frontal Gyrus, pars triangularis | 3.92 | -50 | 22 | 12 |
|  | L Frontal Operculum Cortex | 8.12 | -42 | 16 | -2 |
|  | L Insular Cortex | 7.89 | -36 | 14 | 4 |
|  | L Inferior Frontal Gyrus, pars opercularis | 6.44 | -58 | 12 | 4 |
|  | L Precentral Gyrus | 4.64 | -56 | 6 | 2 |
|  | L Central Opercular Cortex | 5.40 | -50 | 2 | 2 |
| 7,440 |  |  |  |  |  |
|  | R Supramarginal Gyrus, anterior division | 3.55 | 64 | -28 | 30 |
|  | R Parietal Operculum Cortex | 5.12 | 54 | -30 | 26 |
|  | R Angular Gyrus | 5.48 | 56 | -46 | 32 |
|  | R Supramarginal Gyrus, posterior division | 5.39 | 58 | -46 | 38 |
| 4,712 |  |  |  |  |  |
|  | L Postcentral Gyrus | 2.94 | -58 | -22 | 22 |
|  | L Parietal Operculum Cortex | 4.14 | -54 | -34 | 24 |
|  | L Supramarginal Gyrus, anterior division | 5.09 | -66 | -38 | 26 |
|  | L Supramarginal Gyrus, posterior division | 4.89 | -64 | -48 | 34 |
|  | L Angular Gyrus | 3.35 | -58 | -54 | 34 |
| 4,088 |  |  |  |  |  |
|  | R Frontal Pole | 5.87 | 26 | 46 | 26 |
| 3,608 |  |  |  |  |  |
|  | L Frontal Pole | 6.22 | -36 | 48 | 30 |
|  | L Middle Frontal Gyrus | 4.12 | -32 | 30 | 40 |
| 3,040 |  |  |  |  |  |
|  | L Putamen | 6.01 | -20 | 8 | 0 |
| 2,664 |  |  |  |  |  |
|  | R Middle Frontal Gyrus | 6.09 | 46 | 2 | 56 |
|  | R Precentral Gyrus | 4.46 | 40 | -14 | 54 |
| 2,640 |  |  |  |  |  |
|  | R Temporal Occipital Fusiform Cortex | 3.48 | 36 | -60 | -20 |
| 1,448 |  |  |  |  |  |
|  | R Superior Frontal Gyrus | 4.66 | 14 | -10 | 68 |
| 1,416 |  |  |  |  |  |
|  | R Middle Temporal Gyrus, posterior division | 4.72 | 56 | -28 | -4 |
|  | R Middle Temporal Gyrus, temporooccipital part | 2.70 | 64 | -38 | 0 |
| 1,040 |  |  |  |  |  |
|  | R Brain-Stem | 4.69 | 2 | -34 | -50 |
|  | L Brain-Stem | 3.78 | -8 | -38 | -48 |
| 840 |  |  |  |  |  |
|  | L Precentral Gyrus | 4.26 | -40 | -2 | 52 |
| 640 |  |  |  |  |  |
|  | R Occipital Pole | 3.89 | 24 | -94 | 16 |
| 408 |  |  |  |  |  |
|  | R Cingulate Gyrus, posterior division | 4.18 | 10 | -24 | 42 |
| 296 |  |  |  |  |  |
|  | R Temporal Pole | 3.63 | 48 | 10 | -40 |
| 280 |  |  |  |  |  |
|  | R Precentral Gyrus | 3.38 | 34 | -22 | 52 |
| 176 |  |  |  |  |  |
|  | R Precentral Gyrus | 4.40 | 32 | -14 | 64 |
| 136 |  |  |  |  |  |
|  | L Cingulate Gyrus, posterior division | 3.58 | -10 | -24 | 40 |
| 136 |  |  |  |  |  |
|  | L Supramarginal Gyrus, posterior division | 3.34 | -56 | -48 | 50 |
| 128 |  |  |  |  |  |
|  | R Temporal Occipital Fusiform Cortex | 3.32 | 28 | -56 | -18 |
| 128 |  |  |  |  |  |
|  | R Superior Parietal Lobule | 3.46 | 24 | -42 | 60 |
| 112 |  |  |  |  |  |
|  | L Precentral Gyrus | 3.34 | -26 | -10 | 58 |
| 104 |  |  |  |  |  |
|  | L Frontal Pole | 3.26 | -26 | 48 | 16 |
| 72 |  |  |  |  |  |
|  | L Caudate | 3.79 | -16 | 18 | 4 |
| 64 |  |  |  |  |  |
|  | R Inferior Temporal Gyrus, anterior division | 3.54 | 48 | 0 | -34 |
| 56 |  |  |  |  |  |
|  | L Thalamus | 3.40 | -10 | -10 | 6 |
| 56 |  |  |  |  |  |
|  | R Thalamus | 3.85 | 4 | -14 | 16 |
| 48 |  |  |  |  |  |
|  | R Caudate | 3.02 | 16 | -14 | 24 |
| 48 |  |  |  |  |  |
|  | L Superior Frontal Gyrus | 3.14 | -14 | 2 | 68 |
| 40 |  |  |  |  |  |
|  | L Caudate | 3.16 | -14 | -4 | 20 |
| 40 |  |  |  |  |  |
|  | L Precentral Gyrus | 2.82 | -50 | -2 | 42 |
| 32 |  |  |  |  |  |
|  | R Frontal Pole | 2.97 | 28 | 62 | -12 |
| 32 |  |  |  |  |  |
|  | R Supramarginal Gyrus, posterior division | 2.87 | 66 | -42 | 10 |
| 32 |  |  |  |  |  |
|  | L Caudate | 2.81 | -14 | 8 | 18 |
| 32 |  |  |  |  |  |
|  | R Thalamus | 3.19 | 14 | -20 | 16 |
| 32 |  |  |  |  |  |
|  | L Thalamus | 3.26 | -14 | -18 | 18 |
| 24 |  |  |  |  |  |
|  | L Inferior Temporal Gyrus, anterior division | 3.25 | -50 | -2 | -34 |
| 24 |  |  |  |  |  |
|  | R Amygdala | 3.07 | 24 | -14 | -12 |
| 24 |  |  |  |  |  |
|  | R Amygdala | 3.29 | 22 | -6 | -12 |
| 24 |  |  |  |  |  |
|  | R Middle Temporal Gyrus, temporooccipital part | 2.84 | 64 | -38 | 4 |
| 24 |  |  |  |  |  |
|  | L Frontal Pole | 2.70 | -42 | 52 | 16 |
| 24 |  |  |  |  |  |
|  | L Caudate | 2.91 | -14 | -10 | 20 |
| 24 |  |  |  |  |  |
|  | L Precuneus Cortex | 2.59 | -10 | -46 | 50 |
| 16 |  |  |  |  |  |
|  | R Parahippocampal Gyrus, anterior division | 3.06 | 24 | -10 | -32 |
| 16 |  |  |  |  |  |
|  | R Brain-Stem | 2.75 | 4 | -34 | -22 |
| 16 |  |  |  |  |  |
|  | R Brain-Stem | 2.89 | 8 | -12 | -24 |
| 16 |  |  |  |  |  |
|  | R Brain-Stem | 2.73 | 6 | -22 | -20 |
| 16 |  |  |  |  |  |
|  | L Thalamus | 2.87 | -8 | -2 | 10 |
| 16 |  |  |  |  |  |
|  | L Thalamus | 3.48 | -6 | -26 | 14 |
| 16 |  |  |  |  |  |
|  | R Precentral Gyrus | 2.43 | 60 | 10 | 24 |
| 16 |  |  |  |  |  |
|  | R Precentral Gyrus | 2.56 | 40 | 2 | 32 |
| 16 |  |  |  |  |  |
|  | R Cingulate Gyrus, anterior division | 2.83 | 2 | -10 | 42 |
| 16 |  |  |  |  |  |
|  | R Postcentral Gyrus | 2.70 | 46 | -28 | 52 |
| 16 |  |  |  |  |  |
|  | R Superior Parietal Lobule | 2.69 | 16 | -50 | 64 |
| 8 |  |  |  |  |  |
|  | L Brain-Stem | 2.67 | -4 | -44 | -60 |
| 8 |  |  |  |  |  |
|  | R Brain-Stem | 2.60 | 4 | -46 | -58 |
| 8 |  |  |  |  |  |
|  | L Brain-Stem | 2.54 | -2 | -40 | -56 |
| 8 |  |  |  |  |  |
|  | R Brain-Stem | 2.76 | 8 | -44 | -48 |
| 8 |  |  |  |  |  |
|  | R Parahippocampal Gyrus, anterior division | 2.69 | 22 | -8 | -36 |
| 8 |  |  |  |  |  |
|  | R Temporal Pole | 2.62 | 54 | 10 | -36 |
| 8 |  |  |  |  |  |
|  | R Brain-Stem | 2.58 | 12 | -16 | -28 |
| 8 |  |  |  |  |  |
|  | L Brain-Stem | 2.43 | -4 | -16 | -20 |
| 8 |  |  |  |  |  |
|  | R Frontal Orbital Cortex | 2.46 | 34 | 26 | -14 |
| 8 |  |  |  |  |  |
|  | R Amygdala | 2.72 | 28 | -8 | -12 |
| 8 |  |  |  |  |  |
|  | R Thalamus | 2.44 | 8 | -6 | -2 |
| 8 |  |  |  |  |  |
|  | L Caudate | 2.38 | -16 | 18 | -2 |
| 8 |  |  |  |  |  |
|  | R Thalamus | 2.82 | 0 | -8 | 0 |
| 8 |  |  |  |  |  |
|  | L Pallidum | 2.46 | -12 | 2 | 0 |
| 8 |  |  |  |  |  |
|  | R Occipital Pole | 2.52 | 32 | -90 | 4 |
| 8 |  |  |  |  |  |
|  | R Thalamus | 2.72 | 14 | -12 | 6 |
| 8 |  |  |  |  |  |
|  | L Caudate | 2.73 | -8 | 10 | 6 |
| 8 |  |  |  |  |  |
|  | R Putamen | 2.62 | 28 | -12 | 8 |
| 8 |  |  |  |  |  |
|  | R Thalamus | 2.65 | 12 | -10 | 8 |
| 8 |  |  |  |  |  |
|  | R Thalamus | 2.97 | 4 | -18 | 14 |
| 8 |  |  |  |  |  |
|  | R Parietal Operculum Cortex | 2.92 | 44 | -20 | 18 |
| 8 |  |  |  |  |  |
|  | L Frontal Pole | 2.50 | -34 | 44 | 20 |
| 8 |  |  |  |  |  |
|  | R Precentral Gyrus | 2.97 | 56 | 6 | 26 |
| 8 |  |  |  |  |  |
|  | L Frontal Pole | 2.53 | -26 | 44 | 26 |
| 8 |  |  |  |  |  |
|  | R Cingulate Gyrus, anterior division | 2.61 | 4 | 8 | 28 |
| 8 |  |  |  |  |  |
|  | L Frontal Pole | 2.64 | -24 | 46 | 28 |
| 8 |  |  |  |  |  |
|  | L Precentral Gyrus | 2.53 | -32 | -10 | 58 |
| 8 |  |  |  |  |  |
|  | R Superior Frontal Gyrus | 2.54 | 26 | 2 | 64 |
| 8 |  |  |  |  |  |
|  | R Precentral Gyrus | 2.88 | 28 | -20 | 68 |

**Supplementary Table S17. [CS**_**phasic**_ **> CT**_**phasic**_**].** Descriptive statistics for clusters and local maxima showing greater activation for Certain-Safety relative to Certain-Threat anticipation for the OSP Phasic regressor (FDR *q*<0.05, whole-brain corrected). 

| ***mm***^***3***^ | **Label** | ***t*** | ***x*** | ***y*** | ***z*** |
| --- | --- | --- | --- | --- | --- |
| 5,896 |  |  |  |  |  |
|  | L Precentral Gyrus | 6.35 | -8 | -20 | 80 |
|  | L Superior Parietal Lobule | 4.92 | -30 | -42 | 68 |
|  | L Postcentral Gyrus | 6.59 | -10 | -44 | 72 |
| 2,456 |  |  |  |  |  |
|  | R Precentral Gyrus | 6.66 | 12 | -24 | 80 |
|  | R Postcentral Gyrus | 5.13 | 10 | -42 | 72 |
|  | R Precuneus Cortex | 4.96 | 6 | -44 | 68 |
| 632 |  |  |  |  |  |
|  | R Frontal Pole | 4.18 | 26 | 38 | 50 |
|  | R Superior Frontal Gyrus | 5.19 | 24 | 30 | 48 |
| 432 |  |  |  |  |  |
|  | R Cingulate Gyrus, posterior division | 4.69 | 0 | -46 | 14 |
| 312 |  |  |  |  |  |
|  | R Postcentral Gyrus | 4.78 | 66 | -12 | 32 |
| 288 |  |  |  |  |  |
|  | R Superior Parietal Lobule | 5.58 | 30 | -56 | 60 |
| 280 |  |  |  |  |  |
|  | R Superior Parietal Lobule | 4.79 | 44 | -44 | 62 |
| 272 |  |  |  |  |  |
|  | L Postcentral Gyrus | 4.96 | -58 | -12 | 46 |
| 152 |  |  |  |  |  |
|  | L Lateral Occipital Cortex, superior division | 4.21 | -32 | -64 | 58 |
| 120 |  |  |  |  |  |
|  | L Temporal Pole | 4.58 | -36 | 22 | -34 |
| 96 |  |  |  |  |  |
|  | R Precuneus Cortex | 4.76 | 2 | -60 | 16 |
| 96 |  |  |  |  |  |
|  | R Lateral Occipital Cortex, superior division | 3.94 | 30 | -70 | 60 |
| 88 |  |  |  |  |  |
|  | R Middle Frontal Gyrus | 4.13 | 30 | 18 | 60 |
| 72 |  |  |  |  |  |
|  | R Lateral Occipital Cortex, superior division | 4.23 | 18 | -82 | 48 |
| 72 |  |  |  |  |  |
|  | R Lateral Occipital Cortex, superior division | 4.04 | 26 | -78 | 50 |
| 64 |  |  |  |  |  |
|  | R Lateral Occipital Cortex, superior division | 3.67 | 50 | -72 | 34 |
| 64 |  |  |  |  |  |
|  | L Lateral Occipital Cortex, superior division | 4.01 | -26 | -82 | 44 |
| 56 |  |  |  |  |  |
|  | L Frontal Pole | 4.11 | -48 | 44 | -12 |
| 56 |  |  |  |  |  |
|  | R Superior Frontal Gyrus | 4.35 | 12 | 32 | 58 |
| 48 |  |  |  |  |  |
|  | R Temporal Pole | 3.84 | 40 | 24 | -26 |
| 48 |  |  |  |  |  |
|  | L Superior Frontal Gyrus | 3.51 | -24 | 16 | 52 |
| 40 |  |  |  |  |  |
|  | L Subcallosal Cortex | 3.67 | -2 | 14 | -10 |
| 40 |  |  |  |  |  |
|  | R Frontal Pole | 4.40 | 46 | 54 | -8 |
| 40 |  |  |  |  |  |
|  | R Postcentral Gyrus | 3.97 | 58 | -18 | 36 |
| 40 |  |  |  |  |  |
|  | L Superior Frontal Gyrus | 3.89 | -4 | 16 | 66 |
| 32 |  |  |  |  |  |
|  | R Frontal Pole | 3.96 | 12 | 42 | 48 |
| 32 |  |  |  |  |  |
|  | L Postcentral Gyrus | 3.89 | -54 | -28 | 52 |
| 24 |  |  |  |  |  |
|  | R Temporal Pole | 3.95 | 36 | 22 | -36 |
| 24 |  |  |  |  |  |
|  | L Temporal Pole | 3.98 | -30 | 12 | -34 |
| 24 |  |  |  |  |  |
|  | R Frontal Pole | 3.86 | 14 | 58 | -22 |
| 24 |  |  |  |  |  |
|  | R Frontal Medial Cortex | 4.51 | 4 | 34 | -20 |
| 24 |  |  |  |  |  |
|  | R Parahippocampal Gyrus, posterior division | 3.84 | 28 | -34 | -14 |
| 24 |  |  |  |  |  |
|  | R Lingual Gyrus | 3.78 | 4 | -86 | -4 |
| 24 |  |  |  |  |  |
|  | L Frontal Pole | 3.80 | -44 | 48 | -6 |
| 24 |  |  |  |  |  |
|  | L Frontal Pole | 3.79 | -22 | 62 | -6 |
| 24 |  |  |  |  |  |
|  | R Lingual Gyrus | 3.66 | 12 | -44 | -4 |
| 24 |  |  |  |  |  |
|  | R Postcentral Gyrus | 3.67 | 58 | -14 | 30 |
| 24 |  |  |  |  |  |
|  | R Cuneal Cortex | 3.56 | 4 | -82 | 42 |
| 24 |  |  |  |  |  |
|  | R Lateral Occipital Cortex, superior division | 3.46 | 32 | -76 | 42 |
| 24 |  |  |  |  |  |
|  | R Lateral Occipital Cortex, superior division | 3.93 | 36 | -70 | 52 |
| 24 |  |  |  |  |  |
|  | L Superior Parietal Lobule | 3.75 | -34 | -54 | 58 |
| 24 |  |  |  |  |  |
|  | L Lateral Occipital Cortex, superior division | 3.49 | -22 | -64 | 62 |
| 16 |  |  |  |  |  |
|  | L Frontal Orbital Cortex | 3.78 | -36 | 20 | -24 |
| 16 |  |  |  |  |  |
|  | R Parahippocampal Gyrus, posterior division | 3.66 | 22 | -30 | -16 |
| 16 |  |  |  |  |  |
|  | L Frontal Pole | 3.69 | -42 | 58 | -8 |
| 16 |  |  |  |  |  |
|  | L Paracingulate Gyrus | 3.45 | -10 | 46 | -6 |
| 16 |  |  |  |  |  |
|  | R Precentral Gyrus | 3.49 | 60 | -6 | 44 |
| 16 |  |  |  |  |  |
|  | R Lateral Occipital Cortex, superior division | 3.59 | 40 | -62 | 54 |
| 16 |  |  |  |  |  |
|  | L Superior Frontal Gyrus | 3.92 | -6 | 38 | 56 |
| 16 |  |  |  |  |  |
|  | L Lateral Occipital Cortex, superior division | 3.55 | -24 | -66 | 58 |
| 16 |  |  |  |  |  |
|  | R Lateral Occipital Cortex, superior division | 3.53 | 8 | -66 | 66 |
| 16 |  |  |  |  |  |
|  | L Superior Frontal Gyrus | 3.59 | -6 | 20 | 68 |
| 8 |  |  |  |  |  |
|  | R Parahippocampal Gyrus, posterior division | 3.56 | 26 | -28 | -22 |
| 8 |  |  |  |  |  |
|  | L Frontal Pole | 3.50 | -16 | 38 | -22 |
| 8 |  |  |  |  |  |
|  | R Subcallosal Cortex | 3.44 | 4 | 26 | -20 |
| 8 |  |  |  |  |  |
|  | R Temporal Fusiform Cortex, posterior division | 3.72 | 40 | -26 | -18 |
| 8 |  |  |  |  |  |
|  | L Lingual Gyrus | 3.41 | -4 | -86 | -14 |
| 8 |  |  |  |  |  |
|  | R Parahippocampal Gyrus, posterior division | 3.62 | 30 | -36 | -12 |
| 8 |  |  |  |  |  |
|  | R Frontal Pole | 4.00 | 46 | 48 | -12 |
| 8 |  |  |  |  |  |
|  | L Lingual Gyrus | 3.53 | -2 | -78 | -10 |
| 8 |  |  |  |  |  |
|  | R Occipital Pole | 3.54 | 10 | -96 | -4 |
| 8 |  |  |  |  |  |
|  | R Frontal Pole | 3.40 | 40 | 60 | 2 |
| 8 |  |  |  |  |  |
|  | L Heschls Gyrus (includes H1 and H2) | 3.43 | -50 | -16 | 10 |
| 8 |  |  |  |  |  |
|  | L Frontal Pole | 3.49 | -10 | 70 | 10 |
| 8 |  |  |  |  |  |
|  | R Lateral Occipital Cortex, superior division | 3.43 | 50 | -78 | 26 |
| 8 |  |  |  |  |  |
|  | L Lateral Occipital Cortex, superior division | 3.52 | -42 | -80 | 38 |
| 8 |  |  |  |  |  |
|  | R Lateral Occipital Cortex, superior division | 3.38 | 34 | -80 | 42 |
| 8 |  |  |  |  |  |
|  | R Lateral Occipital Cortex, superior division | 3.57 | 40 | -76 | 44 |
| 8 |  |  |  |  |  |
|  | R Frontal Pole | 3.37 | 14 | 50 | 44 |
| 8 |  |  |  |  |  |
|  | L Superior Frontal Gyrus | 3.40 | -24 | 18 | 46 |
| 8 |  |  |  |  |  |
|  | R Frontal Pole | 3.61 | 18 | 46 | 46 |
| 8 |  |  |  |  |  |
|  | L Frontal Pole | 3.39 | -18 | 42 | 50 |
| 8 |  |  |  |  |  |
|  | L Superior Frontal Gyrus | 3.41 | -16 | 18 | 58 |
| 8 |  |  |  |  |  |
|  | R Lateral Occipital Cortex, superior division | 3.36 | 22 | -64 | 64 |
| 8 |  |  |  |  |  |
|  | R Lateral Occipital Cortex, superior division | 3.42 | 26 | -62 | 66 |
| 8 |  |  |  |  |  |
|  | L Superior Parietal Lobule | 3.36 | -28 | -56 | 66 |

# Supplementary Table S18. [US_phasic _> UT_phasic_]. Descriptive statistics for clusters and local maxima showing greater activation for Uncertain-Safety relative to Uncertain-Threat anticipation for the OSP Phasic regressor (FDR *q*<0.05, whole-brain corrected). 

| ***mm***^***3***^ | **Label** | ***t*** | ***x*** | ***y*** | ***z*** |
| --- | --- | --- | --- | --- | --- |
| 2,776 |  |  |  |  |  |
|  | L Postcentral Gyrus | 6.74 | -46 | -16 | 62 |
|  | L Precentral Gyrus | 5.91 | -42 | -18 | 66 |
|  | L Superior Parietal Lobule | 4.94 | -38 | -42 | 62 |
| 1,432 |  |  |  |  |  |
|  | R Occipital Pole | 6.22 | 14 | -96 | -2 |
| 1,304 |  |  |  |  |  |
|  | L Frontal Pole | 4.98 | -12 | 68 | -16 |
|  | R Frontal Pole | 5.39 | 2 | 68 | -14 |
| 888 |  |  |  |  |  |
|  | L Lateral Occipital Cortex, superior division | 5.69 | -18 | -68 | 64 |
| 856 |  |  |  |  |  |
|  | R Postcentral Gyrus | 5.85 | 46 | -20 | 64 |
| 784 |  |  |  |  |  |
|  | L Occipital Pole | 5.78 | -20 | -98 | -10 |
| 760 |  |  |  |  |  |
|  | R Frontal Orbital Cortex | 5.56 | 14 | 16 | -16 |
| 528 |  |  |  |  |  |
|  | L Middle Frontal Gyrus | 4.46 | -32 | 0 | 66 |
|  | L Precentral Gyrus | 4.45 | -36 | -6 | 66 |
| 432 |  |  |  |  |  |
|  | R Postcentral Gyrus | 4.39 | 42 | -36 | 60 |
| 344 |  |  |  |  |  |
|  | R Lingual Gyrus | 4.68 | 10 | -70 | -10 |
| 344 |  |  |  |  |  |
|  | L Occipital Pole | 5.25 | -20 | -102 | 6 |
| 280 |  |  |  |  |  |
|  | L Frontal Orbital Cortex | 4.99 | -26 | 30 | -20 |
| 224 |  |  |  |  |  |
|  | L Frontal Orbital Cortex | 4.15 | -18 | 10 | -22 |
| 200 |  |  |  |  |  |
|  | R Subcallosal Cortex | 5.15 | 2 | 18 | -6 |
|  | L Subcallosal Cortex | 5.28 | -2 | 16 | -6 |
| 192 |  |  |  |  |  |
|  | R Frontal Pole | 4.76 | 6 | 74 | 2 |
| 192 |  |  |  |  |  |
|  | R Precentral Gyrus | 4.39 | 38 | -6 | 66 |
| 152 |  |  |  |  |  |
|  | L Brain-Stem | 4.27 | -2 | -34 | -58 |
|  | R Brain-Stem | 3.91 | 8 | -36 | -60 |
| 144 |  |  |  |  |  |
|  | L Temporal Pole | 4.57 | -46 | 2 | -38 |
| 128 |  |  |  |  |  |
|  | L Hippocampus | 5.13 | -20 | -22 | -16 |
| 128 |  |  |  |  |  |
|  | R Occipital Pole | 3.85 | 28 | -98 | 6 |
| 120 |  |  |  |  |  |
|  | L Frontal Pole | 4.80 | -12 | 40 | -24 |
| 120 |  |  |  |  |  |
|  | R Hippocampus | 4.66 | 20 | -20 | -16 |
| 120 |  |  |  |  |  |
|  | L Lateral Occipital Cortex, superior division | 3.68 | -32 | -88 | 36 |
| 120 |  |  |  |  |  |
|  | R Precentral Gyrus | 3.47 | 4 | -16 | 76 |
| 104 |  |  |  |  |  |
|  | L Frontal Pole | 4.17 | -14 | 72 | 0 |
| 96 |  |  |  |  |  |
|  | L Occipital Fusiform Gyrus | 3.93 | -20 | -82 | -14 |
| 88 |  |  |  |  |  |
|  | R Temporal Pole | 4.67 | 40 | 20 | -30 |
| 88 |  |  |  |  |  |
|  | L Frontal Pole | 3.57 | -10 | 48 | -22 |
| 88 |  |  |  |  |  |
|  | R Lateral Occipital Cortex, superior division | 4.19 | 46 | -74 | 42 |
| 88 |  |  |  |  |  |
|  | R Postcentral Gyrus | 4.16 | 56 | -22 | 54 |
| 72 |  |  |  |  |  |
|  | L Frontal Pole | 4.48 | -10 | 52 | -28 |
| 72 |  |  |  |  |  |
|  | R Lateral Occipital Cortex, superior division | 4.15 | 14 | -78 | 56 |
| 64 |  |  |  |  |  |
|  | L Lingual Gyrus | 4.43 | -2 | -74 | -8 |
| 64 |  |  |  |  |  |
|  | L Precentral Gyrus | 4.15 | -32 | -10 | 50 |
| 64 |  |  |  |  |  |
|  | L Superior Parietal Lobule | 4.40 | -30 | -48 | 48 |
| 56 |  |  |  |  |  |
|  | L Lateral Occipital Cortex, superior division | 3.80 | -22 | -86 | 44 |
| 56 |  |  |  |  |  |
|  | L Superior Parietal Lobule | 3.57 | -22 | -50 | 72 |
| 56 |  |  |  |  |  |
|  | L Superior Parietal Lobule | 3.74 | -10 | -54 | 72 |
| 56 |  |  |  |  |  |
|  | R Superior Frontal Gyrus | 3.50 | 22 | -8 | 72 |
| 56 |  |  |  |  |  |
|  | L Juxtapositional Lobule Cortex (formerly Supplementary Motor Cortex) | 3.89 | -4 | 2 | 72 |
| 56 |  |  |  |  |  |
|  | R Superior Parietal Lobule | 3.92 | 22 | -50 | 74 |
| 48 |  |  |  |  |  |
|  | R Temporal Pole | 3.74 | 26 | 6 | -34 |
| 48 |  |  |  |  |  |
|  | L Lingual Gyrus | 3.67 | -12 | -78 | -12 |
|  | L Occipital Fusiform Gyrus | 3.67 | -16 | -78 | -16 |
| 48 |  |  |  |  |  |
|  | L Cingulate Gyrus, posterior division | 4.70 | -6 | -44 | 4 |
| 48 |  |  |  |  |  |
|  | L Caudate | 4.33 | -8 | 0 | 8 |
| 48 |  |  |  |  |  |
|  | L Postcentral Gyrus | 3.83 | -58 | -10 | 46 |
| 40 |  |  |  |  |  |
|  | L Frontal Orbital Cortex | 3.78 | -48 | 28 | -12 |
| 32 |  |  |  |  |  |
|  | R Inferior Temporal Gyrus, anterior division | 3.68 | 42 | -2 | -40 |
| 32 |  |  |  |  |  |
|  | L Subcallosal Cortex | 3.98 | -4 | 14 | -12 |
| 32 |  |  |  |  |  |
|  | R Lingual Gyrus | 4.57 | 12 | -58 | -4 |
| 32 |  |  |  |  |  |
|  | R Paracingulate Gyrus | 4.14 | 14 | 48 | 2 |
| 32 |  |  |  |  |  |
|  | L Frontal Pole | 3.99 | -10 | 74 | 6 |
| 32 |  |  |  |  |  |
|  | L Postcentral Gyrus | 3.66 | -56 | -26 | 52 |
| 32 |  |  |  |  |  |
|  | L Postcentral Gyrus | 3.75 | -48 | -36 | 58 |
| 32 |  |  |  |  |  |
|  | R Lateral Occipital Cortex, superior division | 3.70 | 14 | -64 | 66 |
| 32 |  |  |  |  |  |
|  | R Superior Parietal Lobule | 3.99 | 38 | -44 | 68 |
| 24 |  |  |  |  |  |
|  | L Temporal Pole | 3.56 | -34 | 16 | -30 |
| 24 |  |  |  |  |  |
|  | R Frontal Pole | 3.66 | 28 | 34 | -20 |
| 24 |  |  |  |  |  |
|  | R Lingual Gyrus | 3.62 | 22 | -58 | -10 |
| 24 |  |  |  |  |  |
|  | R Lingual Gyrus | 3.78 | 2 | -72 | -6 |
| 24 |  |  |  |  |  |
|  | R Lingual Gyrus | 3.65 | 2 | -64 | 2 |
| 24 |  |  |  |  |  |
|  | R Occipital Pole | 3.45 | 24 | -96 | 20 |
| 24 |  |  |  |  |  |
|  | L Superior Frontal Gyrus | 3.69 | -24 | -4 | 56 |
| 16 |  |  |  |  |  |
|  | L Brain-Stem | 3.66 | -8 | -48 | -52 |
| 16 |  |  |  |  |  |
|  | R Brain-Stem | 4.17 | 8 | -30 | -36 |
| 16 |  |  |  |  |  |
|  | R Temporal Pole | 3.73 | 36 | 14 | -32 |
| 16 |  |  |  |  |  |
|  | R Frontal Pole | 3.52 | 18 | 36 | -20 |
| 16 |  |  |  |  |  |
|  | L Frontal Orbital Cortex | 3.61 | -14 | 20 | -16 |
| 16 |  |  |  |  |  |
|  | R Occipital Fusiform Gyrus | 3.45 | 16 | -88 | -12 |
| 16 |  |  |  |  |  |
|  | R Hippocampus | 4.21 | 28 | -18 | -12 |
| 16 |  |  |  |  |  |
|  | R Lingual Gyrus | 4.12 | 30 | -46 | -4 |
| 16 |  |  |  |  |  |
|  | R Hippocampus | 3.93 | 32 | -34 | -2 |
| 16 |  |  |  |  |  |
|  | Bed Nucleus of the Stria Terminalis | 3.37 | 8 | 4 | 2 |
|  | R Occipital Pole | 3.43 | 38 | -94 | 8 |
| 16 |  |  |  |  |  |
|  | R Lateral Occipital Cortex, inferior division | 3.50 | 56 | -72 | 8 |
| 16 |  |  |  |  |  |
|  | L Precentral Gyrus | 3.57 | -52 | 0 | 24 |
| 16 |  |  |  |  |  |
|  | R Lateral Occipital Cortex, superior division | 3.40 | 22 | -62 | 64 |
| 16 |  |  |  |  |  |
|  | R Postcentral Gyrus | 3.54 | 12 | -42 | 74 |
| 16 |  |  |  |  |  |
|  | R Postcentral Gyrus | 3.43 | 10 | -38 | 80 |
| 16 |  |  |  |  |  |
|  | R Precentral Gyrus | 3.71 | 10 | -26 | 80 |
| 8 |  |  |  |  |  |
|  | L Brain-Stem | 3.29 | -8 | -40 | -60 |
| 8 |  |  |  |  |  |
|  | R Temporal Fusiform Cortex, anterior division | 3.42 | 24 | -6 | -42 |
| 8 |  |  |  |  |  |
|  | R Inferior Temporal Gyrus, anterior division | 3.38 | 42 | 2 | -40 |
| 8 |  |  |  |  |  |
|  | R Temporal Pole | 3.35 | 30 | 10 | -34 |
| 8 |  |  |  |  |  |
|  | L Temporal Pole | 3.56 | -50 | 14 | -32 |
| 8 |  |  |  |  |  |
|  | L Temporal Pole | 3.47 | -24 | 4 | -26 |
| 8 |  |  |  |  |  |
|  | R Frontal Pole | 3.39 | 14 | 34 | -26 |
| 8 |  |  |  |  |  |
|  | R Parahippocampal Gyrus, anterior division | 3.79 | 16 | -6 | -24 |
| 8 |  |  |  |  |  |
|  | L Frontal Pole | 3.47 | -4 | 62 | -24 |
| 8 |  |  |  |  |  |
|  | R Amygdala | 3.61 | 12 | -4 | -18 |
| 8 |  |  |  |  |  |
|  | L Hippocampus | 3.81 | -36 | -26 | -12 |
| 8 |  |  |  |  |  |
|  | L Lingual Gyrus | 3.75 | -12 | -56 | -6 |
| 8 |  |  |  |  |  |
|  | L Frontal Medial Cortex | 3.31 | -8 | 54 | -6 |
| 8 |  |  |  |  |  |
|  | Bed Nucleus of the Stria Terminalis | 3.46 | -6 | 2 | -4 |
| 8 |  |  |  |  |  |
|  | L Frontal Pole | 3.59 | -2 | 66 | 0 |
| 8 |  |  |  |  |  |
|  | R Lateral Occipital Cortex, inferior division | 3.29 | 44 | -64 | 8 |
| 8 |  |  |  |  |  |
|  | L Caudate | 3.66 | -18 | 14 | 14 |
| 8 |  |  |  |  |  |
|  | R Postcentral Gyrus | 3.35 | 64 | -12 | 40 |
| 8 |  |  |  |  |  |
|  | L Postcentral Gyrus | 3.77 | -40 | -34 | 56 |
| 8 |  |  |  |  |  |
|  | R Lateral Occipital Cortex, superior division | 3.45 | 26 | -58 | 60 |

# Supplementary Table S19. [UT_phasic _> CT_phasic_]. Descriptive statistics for clusters and local maxima showing greater activation for Uncertain-Threat relative to Certain-Threat anticipation for the OSP Phasic regressor (FDR *q*<0.05, whole-brain corrected). 

| ***mm***^***3***^ | **Label** | ***t*** | ***x*** | ***y*** | ***z*** |
| --- | --- | --- | --- | --- | --- |
| 43,448 |  |  |  |  |  |
|  | R Thalamus | 4.15 | 14 | -24 | -4 |
|  | R Temporal Fusiform Cortex, posterior division | 3.42 | 34 | -30 | -18 |
|  | R Brain-Stem | 3.28 | 8 | -30 | -14 |
|  | L Temporal Fusiform Cortex, posterior division | 2.85 | -30 | -34 | -20 |
|  | R Parahippocampal Gyrus, posterior division | 3.1 | 20 | -36 | -18 |
|  | L Parahippocampal Gyrus, posterior division | 5.41 | -20 | -40 | -12 |
|  | R Lingual Gyrus | 6.72 | 32 | -40 | -10 |
|  | L Lingual Gyrus | 6.96 | -24 | -46 | -6 |
|  | L Temporal Occipital Fusiform Cortex | 8.24 | -30 | -48 | -8 |
|  | R Temporal Occipital Fusiform Cortex | 6.63 | 28 | -52 | -8 |
|  | L Precuneus Cortex | 4.36 | -18 | -58 | 8 |
|  | R Precuneus Cortex | 3.52 | 16 | -60 | 12 |
|  | L Supracalcarine Cortex | 4.52 | -18 | -64 | 14 |
|  | R Occipital Fusiform Gyrus | 4.93 | 24 | -66 | -10 |
|  | L Occipital Fusiform Gyrus | 5.84 | -22 | -68 | -10 |
|  | R Supracalcarine Cortex | 4.73 | 2 | -76 | 14 |
|  | R Intracalcarine Cortex | 5.52 | 16 | -80 | 8 |
|  | R Lateral Occipital Cortex, superior division | 5.09 | 18 | -80 | 40 |
|  | L Lateral Occipital Cortex, superior division | 5.63 | -42 | -82 | 18 |
|  | L Cuneal Cortex | 6.42 | -2 | -86 | 34 |
|  | R Cuneal Cortex | 6.14 | 6 | -86 | 36 |
|  | L Intracalcarine Cortex | 6.32 | -8 | -88 | 6 |
|  | L Occipital Pole | 6.51 | -8 | -90 | 36 |
|  | R Occipital Pole | 7.96 | 2 | -96 | -2 |
| 4,432 |  |  |  |  |  |
|  | R Frontal Pole | 5.4 | 48 | 48 | -4 |
|  | R Frontal Orbital Cortex | 5.21 | 44 | 30 | -12 |
|  | R Temporal Pole | 4.19 | 56 | 16 | -12 |
| 3,888 |  |  |  |  |  |
|  | R Superior Temporal Gyrus, anterior division | 3.25 | 62 | 0 | -12 |
|  | R Planum Polare | 5.21 | 42 | -12 | -6 |
|  | R Superior Temporal Gyrus, posterior division | 3.88 | 68 | -14 | 2 |
|  | R Central Opercular Cortex | 4.84 | 58 | -16 | 10 |
|  | R Planum Temporale | 4.66 | 54 | -22 | 8 |
|  | R Heschls Gyrus (includes H1 and H2) | 5.78 | 44 | -26 | 10 |
| 2,528 |  |  |  |  |  |
|  | L Superior Temporal Gyrus, anterior division | 3.13 | -62 | -8 | -2 |
|  | L Central Opercular Cortex | 4.68 | -40 | -18 | 20 |
|  | L Heschls Gyrus (includes H1 and H2) | 4.61 | -48 | -26 | 6 |
|  | L Insular Cortex | 4.5 | -32 | -28 | 16 |
|  | L Parietal Operculum Cortex | 4.92 | -34 | -30 | 18 |
|  | L Planum Temporale | 4.78 | -38 | -34 | 12 |
| 2,224 |  |  |  |  |  |
|  | L Frontal Pole | 5.29 | -46 | 46 | -12 |
|  | L Inferior Frontal Gyrus, pars triangularis | 4.46 | -48 | 32 | -4 |
|  | L Frontal Orbital Cortex | 4.23 | -46 | 28 | -6 |
| 1,816 |  |  |  |  |  |
|  | R Frontal Pole | 4.97 | 10 | 50 | 46 |
| 1,368 |  |  |  |  |  |
|  | R Lateral Occipital Cortex, inferior division | 5.06 | 48 | -74 | 12 |
|  | R Lateral Occipital Cortex, superior division | 4.5 | 50 | -78 | 22 |
| 1,176 |  |  |  |  |  |
|  | R Precentral Gyrus | 4.07 | 52 | -6 | 28 |
|  | R Postcentral Gyrus | 5.05 | 64 | -8 | 32 |
| 1,168 |  |  |  |  |  |
|  | L Inferior Frontal Gyrus, pars triangularis | 3.99 | -56 | 28 | 16 |
|  | L Inferior Frontal Gyrus, pars opercularis | 3.68 | -58 | 20 | 16 |
| 1,144 |  |  |  |  |  |
|  | L Frontal Pole | 5.66 | -14 | 58 | 28 |
|  | L Superior Frontal Gyrus | 5.71 | -4 | 56 | 30 |
| 936 |  |  |  |  |  |
|  | L Temporal Pole | 3.65 | -58 | 4 | -4 |
|  | L Planum Polare | 4.99 | -48 | -12 | -2 |
|  | L Insular Cortex | 4.19 | -40 | -14 | -6 |
| 856 |  |  |  |  |  |
|  | L Superior Temporal Gyrus, posterior division | 3.62 | -56 | -28 | -2 |
|  | L Middle Temporal Gyrus, posterior division | 4.71 | -50 | -38 | 0 |
| 680 |  |  |  |  |  |
|  | L Superior Frontal Gyrus | 4.56 | -2 | 48 | 46 |
|  | L Frontal Pole | 4.16 | -12 | 44 | 48 |
| 448 |  |  |  |  |  |
|  | R Frontal Pole | 3.35 | 50 | 36 | 22 |
|  | R Middle Frontal Gyrus | 4.15 | 52 | 28 | 26 |
| 376 |  |  |  |  |  |
|  | R Superior Frontal Gyrus | 3.93 | 22 | 30 | 48 |
|  | R Middle Frontal Gyrus | 3.22 | 28 | 20 | 54 |
| 312 |  |  |  |  |  |
|  | L Superior Parietal Lobule | 5.83 | -26 | -56 | 64 |
| 256 |  |  |  |  |  |
|  | L Frontal Orbital Cortex | 4.67 | -40 | 22 | -14 |
| 256 |  |  |  |  |  |
|  | L Postcentral Gyrus | 3.89 | -54 | -18 | 50 |
| 200 |  |  |  |  |  |
|  | L Temporal Pole | 4.38 | -52 | 8 | -20 |
| 192 |  |  |  |  |  |
|  | L Precentral Gyrus | 4.38 | -52 | -8 | 32 |
| 192 |  |  |  |  |  |
|  | R Supramarginal Gyrus, anterior division | 3.98 | 62 | -24 | 46 |
| 192 |  |  |  |  |  |
|  | L Middle Frontal Gyrus | 4.06 | -38 | 16 | 48 |
| 176 |  |  |  |  |  |
|  | R Frontal Pole | 4.55 | 10 | 68 | 20 |
| 176 |  |  |  |  |  |
|  | R Precentral Gyrus | 3.81 | 38 | -12 | 44 |
| 168 |  |  |  |  |  |
|  | L Precentral Gyrus | 4.8 | -34 | -22 | 44 |
|  | L Postcentral Gyrus | 3.31 | -42 | -26 | 48 |
| 152 |  |  |  |  |  |
|  | L Superior Temporal Gyrus, posterior division | 3.38 | -68 | -32 | 12 |
|  | L Planum Temporale | 3.64 | -62 | -36 | 14 |
| 144 |  |  |  |  |  |
|  | R Temporal Pole | 3.72 | 40 | 26 | -34 |
| 144 |  |  |  |  |  |
|  | L Postcentral Gyrus | 4.36 | -20 | -32 | 72 |
| 136 |  |  |  |  |  |
|  | L Frontal Pole | 3.86 | -24 | 60 | -8 |
| 128 |  |  |  |  |  |
|  | L Temporal Pole | 4.03 | -38 | 24 | -36 |
| 128 |  |  |  |  |  |
|  | R Inferior Frontal Gyrus, pars opercularis | 4.09 | 60 | 20 | 14 |
| 120 |  |  |  |  |  |
|  | L Angular Gyrus | 3.45 | -46 | -54 | 26 |
| 120 |  |  |  |  |  |
|  | L Precentral Gyrus | 3.36 | -26 | -26 | 52 |
| 120 |  |  |  |  |  |
|  | L Superior Frontal Gyrus | 4.25 | -4 | 16 | 62 |
| 112 |  |  |  |  |  |
|  | L Supramarginal Gyrus, posterior division | 3.99 | -60 | -46 | 12 |
| 104 |  |  |  |  |  |
|  | L Cingulate Gyrus, posterior division | 3.8 | -2 | -46 | 12 |
| 104 |  |  |  |  |  |
|  | L Postcentral Gyrus | 3.71 | -42 | -26 | 58 |
| 104 |  |  |  |  |  |
|  | R Precentral Gyrus | 3.91 | 12 | -28 | 70 |
| 96 |  |  |  |  |  |
|  | R Temporal Pole | 3.65 | 52 | 8 | -20 |
|  | R Superior Temporal Gyrus, anterior division | 4.13 | 50 | 2 | -20 |
| 96 |  |  |  |  |  |
|  | R Frontal Pole | 3.41 | 38 | 62 | -10 |
| 96 |  |  |  |  |  |
|  | R Supracalcarine Cortex | 3.78 | 22 | -64 | 16 |
| 96 |  |  |  |  |  |
|  | L Postcentral Gyrus | 3.85 | -6 | -42 | 64 |
| 88 |  |  |  |  |  |
|  | R Cingulate Gyrus, posterior division | 3.77 | 18 | -46 | 2 |
|  | R Lingual Gyrus | 3.25 | 16 | -50 | 0 |
| 88 |  |  |  |  |  |
|  | L Postcentral Gyrus | 3.65 | -66 | -8 | 26 |
| 80 |  |  |  |  |  |
|  | L Superior Temporal Gyrus, anterior division | 3.85 | -52 | 0 | -16 |
| 80 |  |  |  |  |  |
|  | R Superior Temporal Gyrus, posterior division | 3.11 | 68 | -32 | 4 |
| 80 |  |  |  |  |  |
|  | R Precuneus Cortex | 4.51 | 22 | -54 | 8 |
| 80 |  |  |  |  |  |
|  | R Middle Temporal Gyrus, temporooccipital part | 3.33 | 68 | -40 | 6 |
| 80 |  |  |  |  |  |
|  | R Inferior Frontal Gyrus, pars opercularis | 3.36 | 52 | 16 | 28 |
| 72 |  |  |  |  |  |
|  | R Periaqueductal gray | 3.15 | 0 | -32 | -10 |
|  | R Brain-Stem | 3.24 | 4 | -36 | -8 |
| 72 |  |  |  |  |  |
|  | L Putamen | 4.8 | -32 | -12 | -6 |
| 64 |  |  |  |  |  |
|  | R Inferior Temporal Gyrus, temporooccipital part | 4.31 | 56 | -54 | -24 |
| 64 |  |  |  |  |  |
|  | L Temporal Fusiform Cortex, posterior division | 3.61 | -34 | -26 | -22 |
| 64 |  |  |  |  |  |
|  | L Superior Frontal Gyrus | 3.5 | -4 | 28 | 62 |
| 64 |  |  |  |  |  |
|  | L Postcentral Gyrus | 3.19 | -4 | -40 | 72 |
| 56 |  |  |  |  |  |
|  | L Temporal Pole | 3.07 | -36 | 20 | -26 |
| 56 |  |  |  |  |  |
|  | R Middle Temporal Gyrus, anterior division | 3.37 | 58 | 4 | -22 |
| 56 |  |  |  |  |  |
|  | L Frontal Pole | 3.28 | -40 | 56 | -4 |
| 56 |  |  |  |  |  |
|  | R Lingual Gyrus | 3.92 | 12 | -56 | 2 |
| 56 |  |  |  |  |  |
|  | R Central Opercular Cortex | 3.63 | 42 | -8 | 12 |
| 56 |  |  |  |  |  |
|  | L Lateral Occipital Cortex, superior division | 3.31 | -50 | -70 | 26 |
| 56 |  |  |  |  |  |
|  | R Superior Frontal Gyrus | 3.77 | 4 | 36 | 58 |
| 56 |  |  |  |  |  |
|  | L Postcentral Gyrus | 3.3 | -26 | -40 | 68 |
| 48 |  |  |  |  |  |
|  | L Brain-Stem | 3.05 | -6 | -36 | -60 |
| 48 |  |  |  |  |  |
|  | L Temporal Pole | 3.16 | -48 | 12 | -12 |
| 48 |  |  |  |  |  |
|  | L Precentral Gyrus | 3.56 | -14 | -24 | 72 |
| 40 |  |  |  |  |  |
|  | L Temporal Pole | 3.45 | -50 | 20 | -18 |
| 40 |  |  |  |  |  |
|  | R Supramarginal Gyrus, posterior division | 3.7 | 56 | -38 | 14 |
| 40 |  |  |  |  |  |
|  | R Cingulate Gyrus, posterior division | 3.16 | 0 | -44 | 20 |
| 40 |  |  |  |  |  |
|  | L Precentral Gyrus | 3.86 | -36 | -14 | 46 |
| 40 |  |  |  |  |  |
|  | R Superior Frontal Gyrus | 2.98 | 24 | 26 | 58 |
| 32 |  |  |  |  |  |
|  | R Temporal Pole | 4.19 | 48 | 24 | -26 |
| 32 |  |  |  |  |  |
|  | L Middle Temporal Gyrus, posterior division | 3.1 | -64 | -36 | -4 |
| 32 |  |  |  |  |  |
|  | L Central Opercular Cortex | 3.54 | -48 | -12 | 16 |
| 32 |  |  |  |  |  |
|  | L Angular Gyrus | 3.22 | -60 | -56 | 24 |
| 32 |  |  |  |  |  |
|  | L Superior Frontal Gyrus | 3.22 | -26 | 22 | 50 |
| 32 |  |  |  |  |  |
|  | R Middle Frontal Gyrus | 3.26 | 38 | 24 | 52 |
| 32 |  |  |  |  |  |
|  | L Middle Frontal Gyrus | 3.16 | -38 | 18 | 56 |
| 32 |  |  |  |  |  |
|  | L Superior Parietal Lobule | 3.27 | -32 | -50 | 60 |
| 24 |  |  |  |  |  |
|  | R Brain-Stem | 2.88 | 14 | -22 | -24 |
| 24 |  |  |  |  |  |
|  | R Parahippocampal Gyrus, posterior division | 3.44 | 20 | -28 | -18 |
| 24 |  |  |  |  |  |
|  | L Temporal Occipital Fusiform Cortex | 3.24 | -34 | -58 | -16 |
| 24 |  |  |  |  |  |
|  | R Temporal Pole | 3.28 | 60 | 6 | -16 |
| 24 |  |  |  |  |  |
|  | R Frontal Pole | 3.16 | 20 | 44 | -16 |
| 24 |  |  |  |  |  |
|  | L Thalamus | 4.28 | -16 | -32 | -4 |
| 24 |  |  |  |  |  |
|  | R Temporal Pole | 3.7 | 60 | 12 | -6 |
| 24 |  |  |  |  |  |
|  | L Cingulate Gyrus, posterior division | 3.03 | -14 | -48 | 2 |
| 24 |  |  |  |  |  |
|  | R Frontal Pole | 2.91 | 36 | 54 | 4 |
| 24 |  |  |  |  |  |
|  | L Frontal Pole | 3.64 | -2 | 64 | 12 |
| 24 |  |  |  |  |  |
|  | R Inferior Frontal Gyrus, pars opercularis | 3.02 | 48 | 14 | 30 |
| 24 |  |  |  |  |  |
|  | L Superior Frontal Gyrus | 2.98 | -22 | 20 | 44 |
| 24 |  |  |  |  |  |
|  | L Superior Frontal Gyrus | 3.7 | -4 | 36 | 42 |
| 24 |  |  |  |  |  |
|  | L Superior Frontal Gyrus | 3.06 | -2 | 38 | 48 |
| 24 |  |  |  |  |  |
|  | L Superior Parietal Lobule | 3.23 | -18 | -48 | 64 |
| 24 |  |  |  |  |  |
|  | L Postcentral Gyrus | 3.46 | -32 | -38 | 64 |
| 16 |  |  |  |  |  |
|  | R Brain-Stem | 3.29 | 10 | -38 | -58 |
| 16 |  |  |  |  |  |
|  | L Frontal Medial Cortex | 2.94 | -6 | 48 | -26 |
| 16 |  |  |  |  |  |
|  | R Inferior Temporal Gyrus, temporooccipital part | 3.01 | 48 | -42 | -22 |
| 16 |  |  |  |  |  |
|  | R Temporal Pole | 3.3 | 48 | 20 | -22 |
| 16 |  |  |  |  |  |
|  | R Occipital Fusiform Gyrus | 2.88 | 20 | -86 | -18 |
| 16 |  |  |  |  |  |
|  | R Frontal Orbital Cortex | 3.35 | 38 | 22 | -16 |
| 16 |  |  |  |  |  |
|  | L Planum Polare | 3.04 | -44 | -6 | -14 |
| 16 |  |  |  |  |  |
|  | R Frontal Pole | 2.91 | 30 | 64 | -8 |
| 16 |  |  |  |  |  |
|  | L Middle Temporal Gyrus, temporooccipital part | 2.9 | -58 | -54 | -4 |
| 16 |  |  |  |  |  |
|  | R Thalamus | 2.95 | 16 | -20 | 0 |
| 16 |  |  |  |  |  |
|  | R Cingulate Gyrus, anterior division | 3.43 | 0 | 32 | 0 |
| 16 |  |  |  |  |  |
|  | L Lateral Occipital Cortex, inferior division | 2.83 | -46 | -84 | 10 |
| 16 |  |  |  |  |  |
|  | L Frontal Pole | 3.46 | -14 | 68 | 12 |
| 16 |  |  |  |  |  |
|  | R Central Opercular Cortex | 3.1 | 38 | -14 | 22 |
| 16 |  |  |  |  |  |
|  | L Caudate | 3.21 | -16 | -12 | 24 |
| 16 |  |  |  |  |  |
|  | L Middle Frontal Gyrus | 2.93 | -42 | 22 | 24 |
| 16 |  |  |  |  |  |
|  | L Frontal Pole | 2.95 | -20 | 54 | 32 |
| 16 |  |  |  |  |  |
|  | L Postcentral Gyrus | 3.4 | -56 | -14 | 36 |
| 16 |  |  |  |  |  |
|  | R Postcentral Gyrus | 2.97 | 60 | -12 | 38 |
| 16 |  |  |  |  |  |
|  | R Frontal Pole | 3.04 | 22 | 44 | 46 |
| 16 |  |  |  |  |  |
|  | R Superior Frontal Gyrus | 2.99 | 14 | 28 | 56 |
| 16 |  |  |  |  |  |
|  | R Lateral Occipital Cortex, superior division | 3.07 | 18 | -58 | 60 |
| 16 |  |  |  |  |  |
|  | R Superior Parietal Lobule | 3.03 | 26 | -54 | 62 |
| 16 |  |  |  |  |  |
|  | L Postcentral Gyrus | 3.17 | -12 | -40 | 70 |
| 16 |  |  |  |  |  |
|  | R Postcentral Gyrus | 3.15 | 6 | -32 | 80 |
| 8 |  |  |  |  |  |
|  | R Brain-Stem | 2.9 | 8 | -32 | -56 |
| 8 |  |  |  |  |  |
|  | R Brain-Stem | 2.98 | 4 | -30 | -54 |
| 8 |  |  |  |  |  |
|  | R Temporal Pole | 2.92 | 34 | 24 | -42 |
| 8 |  |  |  |  |  |
|  | R Inferior Temporal Gyrus, temporooccipital part | 2.84 | 54 | -46 | -24 |
| 8 |  |  |  |  |  |
|  | R Inferior Temporal Gyrus, temporooccipital part | 2.81 | 52 | -40 | -22 |
| 8 |  |  |  |  |  |
|  | R Parahippocampal Gyrus, posterior division | 2.91 | 26 | -28 | -22 |
| 8 |  |  |  |  |  |
|  | R Frontal Orbital Cortex | 3 | 12 | 16 | -22 |
| 8 |  |  |  |  |  |
|  | L Lateral Occipital Cortex, inferior division | 2.94 | -36 | -82 | -18 |
| 8 |  |  |  |  |  |
|  | L Parahippocampal Gyrus, posterior division | 2.8 | -16 | -30 | -18 |
| 8 |  |  |  |  |  |
|  | R Planum Polare | 2.86 | 42 | -2 | -16 |
| 8 |  |  |  |  |  |
|  | L Frontal Pole | 3.16 | -4 | 60 | -14 |
| 8 |  |  |  |  |  |
|  | L Frontal Pole | 3.17 | -12 | 60 | -14 |
| 8 |  |  |  |  |  |
|  | L Occipital Fusiform Gyrus | 3.39 | -32 | -68 | -12 |
| 8 |  |  |  |  |  |
|  | L Frontal Pole | 2.82 | -38 | 52 | -12 |
| 8 |  |  |  |  |  |
|  | L Paracingulate Gyrus | 2.88 | -12 | 44 | -4 |
| 8 |  |  |  |  |  |
|  | R Insular Cortex | 3.38 | 44 | 2 | -2 |
| 8 |  |  |  |  |  |
|  | L Superior Temporal Gyrus, anterior division | 2.92 | -62 | -2 | 2 |
| 8 |  |  |  |  |  |
|  | R Insular Cortex | 2.8 | 40 | -10 | 4 |
| 8 |  |  |  |  |  |
|  | L Insular Cortex | 2.81 | -38 | -8 | 4 |
| 8 |  |  |  |  |  |
|  | L Intracalcarine Cortex | 2.97 | -16 | -76 | 10 |
| 8 |  |  |  |  |  |
|  | R Central Opercular Cortex | 2.82 | 50 | -12 | 10 |
| 8 |  |  |  |  |  |
|  | L Intracalcarine Cortex | 3.08 | -2 | -66 | 12 |
| 8 |  |  |  |  |  |
|  | L Precuneus Cortex | 2.8 | -2 | -62 | 14 |
| 8 |  |  |  |  |  |
|  | L Central Opercular Cortex | 2.94 | -38 | -10 | 14 |
| 8 |  |  |  |  |  |
|  | L Postcentral Gyrus | 3.05 | -62 | -8 | 14 |
| 8 |  |  |  |  |  |
|  | R Central Opercular Cortex | 2.84 | 50 | -8 | 16 |
| 8 |  |  |  |  |  |
|  | R Lateral Occipital Cortex, superior division | 3.08 | 24 | -86 | 20 |
| 8 |  |  |  |  |  |
|  | L Angular Gyrus | 2.81 | -54 | -60 | 22 |
| 8 |  |  |  |  |  |
|  | L Lateral Occipital Cortex, superior division | 2.85 | -50 | -62 | 24 |
| 8 |  |  |  |  |  |
|  | L Inferior Frontal Gyrus, pars opercularis | 2.84 | -54 | 16 | 24 |
| 8 |  |  |  |  |  |
|  | L Inferior Frontal Gyrus, pars triangularis | 2.81 | -52 | 24 | 24 |
| 8 |  |  |  |  |  |
|  | L Cuneal Cortex | 2.93 | -10 | -80 | 26 |
| 8 |  |  |  |  |  |
|  | R Superior Frontal Gyrus | 2.84 | 6 | 56 | 26 |
| 8 |  |  |  |  |  |
|  | R Cuneal Cortex | 2.95 | 14 | -76 | 30 |
| 8 |  |  |  |  |  |
|  | L Frontal Pole | 3.18 | -20 | 54 | 38 |
| 8 |  |  |  |  |  |
|  | L Superior Frontal Gyrus | 2.83 | -18 | 36 | 44 |
| 8 |  |  |  |  |  |
|  | L Middle Frontal Gyrus | 2.83 | -28 | 28 | 48 |
| 8 |  |  |  |  |  |
|  | R Precuneus Cortex | 2.83 | 2 | -76 | 50 |
| 8 |  |  |  |  |  |
|  | L Postcentral Gyrus | 3.08 | -44 | -20 | 50 |
| 8 |  |  |  |  |  |
|  | L Precentral Gyrus | 2.88 | -36 | -20 | 52 |
| 8 |  |  |  |  |  |
|  | L Postcentral Gyrus | 2.81 | -28 | -36 | 54 |
| 8 |  |  |  |  |  |
|  | L Postcentral Gyrus | 2.98 | -42 | -24 | 54 |
| 8 |  |  |  |  |  |
|  | R Middle Frontal Gyrus | 3 | 46 | 14 | 54 |
| 8 |  |  |  |  |  |
|  | L Superior Frontal Gyrus | 2.9 | -14 | 30 | 56 |
| 8 |  |  |  |  |  |
|  | R Middle Frontal Gyrus | 2.96 | 38 | 12 | 58 |
| 8 |  |  |  |  |  |
|  | R Postcentral Gyrus | 2.82 | 28 | -32 | 68 |
| 8 |  |  |  |  |  |
|  | R Postcentral Gyrus | 2.86 | 24 | -32 | 70 |

# Supplementary Table S20. [UT_onset _> US_onset_]. Descriptive statistics for clusters and local maxima showing greater activation for Uncertain-Threat relative to Uncertain-Safety anticipation for the OSP Onset regressor (FDR *q*<0.05, whole-brain corrected). 

| ***mm***^***3***^ | **Label** | ***t*** | ***x*** | ***y*** | ***z*** |
| --- | --- | --- | --- | --- | --- |
| 286,080 |  |  |  |  |  |
|  | L Frontal Pole | 6.60 | -6 | 68 | -10 |
|  | R Frontal Pole | 6.10 | 2 | 66 | -10 |
|  | L Cingulate Gyrus, anterior division | 3.96 | -4 | 44 | 4 |
|  | R Superior Frontal Gyrus | 7.45 | 4 | 44 | 44 |
|  | L Frontal Medial Cortex | 4.41 | -4 | 38 | -22 |
|  | R Frontal Medial Cortex | 5.02 | 6 | 36 | -18 |
|  | R Inferior Frontal Gyrus, pars triangularis | 6.74 | 56 | 34 | 10 |
|  | L Inferior Frontal Gyrus, pars triangularis | 3.19 | -40 | 26 | 20 |
|  | L Frontal Operculum Cortex | 4.67 | -34 | 24 | 6 |
|  | R Frontal Operculum Cortex | 4.17 | 36 | 24 | 10 |
|  | R Insular Cortex | 7.37 | 32 | 22 | -6 |
|  | L Insular Cortex | 3.86 | -36 | 18 | -6 |
|  | L Frontal Orbital Cortex | 7.73 | -24 | 16 | -20 |
|  | L Temporal Pole | 6.00 | -36 | 16 | -32 |
|  | L Subcallosal Cortex | 4.78 | -4 | 16 | -8 |
|  | R Frontal Orbital Cortex | 9.05 | 22 | 16 | -20 |
|  | L Paracingulate Gyrus | 5.48 | -4 | 12 | 52 |
|  | L Inferior Frontal Gyrus, pars opercularis | 5.22 | -50 | 12 | 28 |
|  | R Subcallosal Cortex | 5.26 | 12 | 12 | -16 |
|  | L Caudate | 4.87 | -6 | 10 | 0 |
|  | L Middle Frontal Gyrus | 4.70 | -44 | 10 | 36 |
|  | R Inferior Frontal Gyrus, pars opercularis | 7.41 | 44 | 10 | 24 |
|  | R Paracingulate Gyrus | 4.69 | 6 | 10 | 52 |
|  | R Caudate | 5.44 | 10 | 8 | 8 |
|  | R Temporal Pole | 8.97 | 54 | 6 | -18 |
|  | R Juxtapositional Lobule Cortex (formerly Supplementary Motor Cortex) | 7.88 | 6 | 6 | 66 |
|  | R Middle Frontal Gyrus | 7.84 | 42 | 6 | 38 |
|  | L Juxtapositional Lobule Cortex (formerly Supplementary Motor Cortex) | 6.72 | -4 | 4 | 66 |
|  | L Superior Frontal Gyrus | 5.46 | -12 | 2 | 70 |
|  | R Precentral Gyrus | 6.98 | 50 | 2 | 50 |
|  | L Precentral Gyrus | 6.82 | -46 | 0 | 48 |
|  | L Amygdala | 4.25 | -24 | 0 | -26 |
|  | L Inferior Temporal Gyrus, anterior division | 4.25 | -42 | 0 | -38 |
|  | R Superior Temporal Gyrus, anterior division | 6.24 | 58 | 0 | -10 |
|  | R Inferior Temporal Gyrus, anterior division | 5.06 | 40 | 0 | -40 |
|  | R Temporal Fusiform Cortex, anterior division | 3.98 | 36 | 0 | -44 |
|  | L Middle Temporal Gyrus, anterior division | 4.34 | -52 | -2 | -18 |
|  | L Temporal Fusiform Cortex, anterior division | 4.93 | -34 | -4 | -40 |
|  | R Amygdala | 4.92 | 16 | -6 | -16 |
|  | L Parahippocampal Gyrus, anterior division | 5.27 | -30 | -10 | -34 |
|  | L Superior Temporal Gyrus, anterior division | 2.77 | -54 | -10 | -8 |
|  | R Middle Temporal Gyrus, posterior division | 6.90 | 58 | -10 | -12 |
|  | R Temporal Fusiform Cortex, posterior division | 5.53 | 34 | -10 | -42 |
|  | R Parahippocampal Gyrus, anterior division | 4.84 | 30 | -10 | -32 |
|  | R Inferior Temporal Gyrus, posterior division | 3.83 | 44 | -24 | -22 |
|  | R Parahippocampal Gyrus, posterior division | 4.31 | 32 | -26 | -22 |
|  | R Superior Temporal Gyrus, posterior division | 7.55 | 50 | -28 | 0 |
|  | L Parahippocampal Gyrus, posterior division | 3.41 | -28 | -30 | -24 |
|  | R Supramarginal Gyrus, anterior division | 2.90 | 54 | -32 | 48 |
|  | R Postcentral Gyrus | 2.81 | 6 | -32 | 80 |
|  | L Thalamus | 3.23 | -16 | -34 | 8 |
|  | R Hippocampus | 8.40 | 28 | -34 | -4 |
|  | R Thalamus | 4.50 | 14 | -34 | 4 |
|  | L Hippocampus | 7.24 | -24 | -38 | 0 |
|  | R Supramarginal Gyrus, posterior division | 4.45 | 42 | -40 | 40 |
|  | L Temporal Fusiform Cortex, posterior division | 4.18 | -40 | -42 | -20 |
|  | L Inferior Temporal Gyrus, posterior division | 3.76 | -46 | -42 | -18 |
|  | R Cingulate Gyrus, posterior division | 6.25 | 10 | -42 | 4 |
|  | L Cingulate Gyrus, posterior division | 5.86 | -6 | -44 | 10 |
|  | R Superior Parietal Lobule | 5.97 | 34 | -44 | 48 |
|  | L Inferior Temporal Gyrus, temporooccipital part | 5.08 | -52 | -50 | -20 |
|  | R Inferior Temporal Gyrus, temporooccipital part | 9.31 | 50 | -50 | -22 |
|  | R Precuneus Cortex | 6.87 | 2 | -54 | 60 |
|  | R Angular Gyrus | 5.53 | 38 | -54 | 50 |
|  | R Temporal Occipital Fusiform Cortex | 6.64 | 46 | -58 | -18 |
|  | L Temporal Occipital Fusiform Cortex | 5.00 | -42 | -60 | -18 |
|  | L Middle Temporal Gyrus, temporooccipital part | 4.68 | -50 | -60 | -2 |
|  | R Middle Temporal Gyrus, temporooccipital part | 4.89 | 54 | -60 | -2 |
|  | L Lateral Occipital Cortex, inferior division | 6.21 | -50 | -64 | -18 |
|  | L Intracalcarine Cortex | 3.96 | -14 | -64 | 10 |
|  | R Lateral Occipital Cortex, superior division | 7.48 | 32 | -64 | 32 |
|  | L Lateral Occipital Cortex, superior division | 7.65 | -8 | -70 | 58 |
|  | L Precuneus Cortex | 8.79 | -2 | -72 | 54 |
|  | L Occipital Fusiform Gyrus | 9.52 | -20 | -82 | -14 |
|  | R Lateral Occipital Cortex, inferior division | 7.82 | 46 | -82 | -8 |
|  | L Cuneal Cortex | 3.64 | -4 | -84 | 18 |
|  | L Lingual Gyrus | 9.21 | -8 | -86 | -12 |
|  | R Occipital Fusiform Gyrus | 11.24 | 22 | -86 | -10 |
|  | R Lingual Gyrus | 9.50 | 4 | -86 | -6 |
|  | R Cuneal Cortex | 2.95 | 0 | -86 | 24 |
|  | L Occipital Pole | 10.14 | -16 | -94 | -12 |
|  | R Occipital Pole | 10.51 | 12 | -94 | -8 |
| 3,432 |  |  |  |  |  |
|  | L Supramarginal Gyrus, anterior division | 3.70 | -44 | -34 | 38 |
|  | L Superior Parietal Lobule | 4.61 | -40 | -44 | 46 |
|  | L Supramarginal Gyrus, posterior division | 5.08 | -36 | -48 | 40 |
|  | L Lateral Occipital Cortex, superior division | 3.04 | -32 | -62 | 46 |
| 2,760 |  |  |  |  |  |
|  | R Precentral Gyrus | 5.07 | 40 | -22 | 66 |
|  | R Postcentral Gyrus | 3.26 | 44 | -22 | 56 |
| 1,232 |  |  |  |  |  |
|  | L Thalamus | 3.20 | -2 | -22 | -2 |
|  | R Thalamus | 5.55 | 4 | -22 | -2 |
|  | R Brain-Stem | 4.99 | 6 | -30 | -6 |
| 1,056 |  |  |  |  |  |
|  | R Brain-Stem | 5.80 | 0 | -18 | -22 |
|  | L Brain-Stem | 3.60 | -6 | -34 | -24 |
| 760 |  |  |  |  |  |
|  | L Cingulate Gyrus, anterior division | 4.81 | -4 | 6 | 28 |
|  | R Cingulate Gyrus, anterior division | 4.58 | 0 | -4 | 36 |
| 704 |  |  |  |  |  |
|  | L Superior Temporal Gyrus, posterior division | 4.31 | -54 | -20 | -4 |
| 192 |  |  |  |  |  |
|  | R Cingulate Gyrus, anterior division | 4.14 | 4 | 12 | 26 |
| 168 |  |  |  |  |  |
|  | R Cingulate Gyrus, anterior division | 4.44 | 6 | 24 | 20 |
| 160 |  |  |  |  |  |
|  | R Brain-Stem | 3.32 | 4 | -20 | -40 |
| 136 |  |  |  |  |  |
|  | L Cingulate Gyrus, posterior division | 3.79 | -2 | -20 | 32 |
| 128 |  |  |  |  |  |
|  | R Supramarginal Gyrus, anterior division | 2.89 | 60 | -22 | 46 |
| 120 |  |  |  |  |  |
|  | L Frontal Pole | 2.86 | -26 | 66 | 8 |
| 112 |  |  |  |  |  |
|  | L Brain-Stem | 3.51 | -2 | -30 | -36 |
|  | R Brain-Stem | 3.41 | 0 | -32 | -38 |
| 104 |  |  |  |  |  |
|  | R Cingulate Gyrus, posterior division | 3.23 | 6 | -24 | 28 |
| 104 |  |  |  |  |  |
|  | L Cingulate Gyrus, posterior division | 3.81 | -10 | -42 | 36 |
| 104 |  |  |  |  |  |
|  | L Precuneus Cortex | 2.57 | -12 | -44 | 40 |
| 96 |  |  |  |  |  |
|  | R Brain-Stem | 3.08 | 10 | -20 | -28 |
| 96 |  |  |  |  |  |
|  | L Brain-Stem | 4.81 | -8 | -28 | -12 |
| 96 |  |  |  |  |  |
|  | L Supramarginal Gyrus, anterior division | 3.01 | -66 | -36 | 34 |
| 88 |  |  |  |  |  |
|  | R Brain-Stem | 3.57 | 2 | -30 | -52 |
| 80 |  |  |  |  |  |
|  | L Angular Gyrus | 3.09 | -50 | -56 | 14 |
| 64 |  |  |  |  |  |
|  | R Brain-Stem | 3.19 | 2 | -22 | -34 |
| 64 |  |  |  |  |  |
|  | L Amygdala | 4.09 | -28 | 2 | -20 |
| 64 |  |  |  |  |  |
|  | R Amygdala | 3.43 | 30 | 2 | -18 |
| 64 |  |  |  |  |  |
|  | R Thalamus | 2.71 | 10 | -6 | 4 |
| 64 |  |  |  |  |  |
|  | R Supramarginal Gyrus, anterior division | 3.27 | 54 | -24 | 42 |
| 56 |  |  |  |  |  |
|  | R Brain-Stem | 2.67 | 10 | -32 | -32 |
| 56 |  |  |  |  |  |
|  | L Parahippocampal Gyrus, posterior division | 3.10 | -24 | -40 | -16 |
| 56 |  |  |  |  |  |
|  | L Temporal Fusiform Cortex, posterior division | 2.87 | -32 | -40 | -14 |
| 56 |  |  |  |  |  |
|  | L Inferior Frontal Gyrus, pars opercularis | 3.79 | -48 | 12 | 10 |
| 48 |  |  |  |  |  |
|  | L Inferior Temporal Gyrus, posterior division | 3.38 | -46 | -24 | -28 |
| 48 |  |  |  |  |  |
|  | R Frontal Orbital Cortex | 2.68 | 40 | 30 | -14 |
| 48 |  |  |  |  |  |
|  | L Middle Temporal Gyrus, posterior division | 2.92 | -62 | -22 | -10 |
| 48 |  |  |  |  |  |
|  | L Postcentral Gyrus | 3.01 | -64 | -22 | 36 |
| 48 |  |  |  |  |  |
|  | R Paracingulate Gyrus | 2.97 | 12 | 14 | 36 |
| 40 |  |  |  |  |  |
|  | L Brain-Stem | 2.69 | -4 | -38 | -40 |
| 40 |  |  |  |  |  |
|  | L Middle Frontal Gyrus | 2.79 | -38 | 34 | 20 |
| 40 |  |  |  |  |  |
|  | L Cingulate Gyrus, anterior division | 3.16 | -4 | 26 | 18 |
| 40 |  |  |  |  |  |
|  | L Cingulate Gyrus, anterior division | 3.05 | -2 | -14 | 30 |
| 32 |  |  |  |  |  |
|  | L Brain-Stem | 3.13 | -8 | -44 | -42 |
| 32 |  |  |  |  |  |
|  | R Brain-Stem | 2.46 | 0 | -16 | -38 |
| 32 |  |  |  |  |  |
|  | L Inferior Temporal Gyrus, posterior division | 3.11 | -60 | -24 | -26 |
| 32 |  |  |  |  |  |
|  | L Insular Cortex | 2.51 | -38 | -8 | -10 |
| 32 |  |  |  |  |  |
|  | L Inferior Frontal Gyrus, pars opercularis | 3.05 | -50 | 16 | 4 |
| 32 |  |  |  |  |  |
|  | L Supramarginal Gyrus, anterior division | 2.43 | -56 | -36 | 34 |
| 32 |  |  |  |  |  |
|  | L Precentral Gyrus | 3.05 | -30 | -12 | 66 |
| 24 |  |  |  |  |  |
|  | L Insular Cortex | 2.50 | -38 | 10 | -14 |
| 24 |  |  |  |  |  |
|  | L Lingual Gyrus | 3.14 | -26 | -42 | -8 |
| 24 |  |  |  |  |  |
|  | L Putamen | 3.31 | -18 | 18 | -8 |
| 24 |  |  |  |  |  |
|  | L Middle Temporal Gyrus, posterior division | 3.06 | -60 | -28 | -4 |
| 24 |  |  |  |  |  |
|  | R Pallidum | 3.51 | 16 | 6 | 2 |
| 24 |  |  |  |  |  |
|  | L Frontal Operculum Cortex | 2.36 | -40 | 12 | 6 |
| 24 |  |  |  |  |  |
|  | L Middle Temporal Gyrus, temporooccipital part | 2.47 | -52 | -58 | 8 |
| 24 |  |  |  |  |  |
|  | L Precuneus Cortex | 2.60 | -2 | -56 | 10 |
| 24 |  |  |  |  |  |
|  | L Paracingulate Gyrus | 2.47 | -10 | 10 | 38 |
| 24 |  |  |  |  |  |
|  | L Middle Frontal Gyrus | 2.99 | -26 | 30 | 40 |
| 24 |  |  |  |  |  |
|  | R Postcentral Gyrus | 3.00 | 48 | -28 | 56 |
| 16 |  |  |  |  |  |
|  | R Brain-Stem | 2.68 | 10 | -44 | -38 |
| 16 |  |  |  |  |  |
|  | R Brain-Stem | 2.70 | 12 | -28 | -38 |
| 16 |  |  |  |  |  |
|  | L Inferior Temporal Gyrus, posterior division | 2.71 | -60 | -36 | -28 |
| 16 |  |  |  |  |  |
|  | L Subcallosal Cortex | 3.16 | -4 | 18 | -24 |
| 16 |  |  |  |  |  |
|  | R Parahippocampal Gyrus, posterior division | 2.85 | 18 | -28 | -20 |
| 16 |  |  |  |  |  |
|  | L Planum Polare | 2.76 | -40 | -4 | -16 |
| 16 |  |  |  |  |  |
|  | L Insular Cortex | 2.69 | -32 | 14 | 6 |
| 16 |  |  |  |  |  |
|  | L Thalamus | 2.45 | -10 | -10 | 8 |
| 16 |  |  |  |  |  |
|  | R Thalamus | 2.61 | 8 | -30 | 10 |
| 16 |  |  |  |  |  |
|  | R Cingulate Gyrus, anterior division | 2.48 | 4 | 38 | 10 |
| 16 |  |  |  |  |  |
|  | L Lateral Occipital Cortex, superior division | 2.65 | -38 | -70 | 16 |
| 16 |  |  |  |  |  |
|  | L Thalamus | 2.57 | -10 | -26 | 16 |
| 16 |  |  |  |  |  |
|  | L Inferior Frontal Gyrus, pars opercularis | 2.50 | -52 | 14 | 16 |
| 16 |  |  |  |  |  |
|  | L Thalamus | 2.51 | -10 | -20 | 18 |
| 16 |  |  |  |  |  |
|  | L Middle Frontal Gyrus | 2.36 | -44 | 30 | 22 |
| 16 |  |  |  |  |  |
|  | R Cingulate Gyrus, anterior division | 2.66 | 8 | 16 | 34 |
| 16 |  |  |  |  |  |
|  | L Precentral Gyrus | 2.81 | -42 | -8 | 48 |
| 16 |  |  |  |  |  |
|  | R Precentral Gyrus | 2.58 | 6 | -22 | 78 |
| 8 |  |  |  |  |  |
|  | R Brain-Stem | 2.48 | 2 | -42 | -62 |
| 8 |  |  |  |  |  |
|  | R Brain-Stem | 2.66 | 0 | -36 | -44 |
| 8 |  |  |  |  |  |
|  | L Inferior Temporal Gyrus, anterior division | 2.75 | -54 | -6 | -42 |
| 8 |  |  |  |  |  |
|  | L Brain-Stem | 2.84 | -16 | -30 | -40 |
| 8 |  |  |  |  |  |
|  | L Brain-Stem | 2.47 | -10 | -42 | -38 |
| 8 |  |  |  |  |  |
|  | L Temporal Fusiform Cortex, posterior division | 2.31 | -38 | -20 | -34 |
| 8 |  |  |  |  |  |
|  | L Temporal Pole | 2.58 | -54 | 12 | -32 |
| 8 |  |  |  |  |  |
|  | R Brain-Stem | 2.50 | 6 | -42 | -30 |
| 8 |  |  |  |  |  |
|  | L Temporal Fusiform Cortex, posterior division | 2.48 | -40 | -32 | -30 |
| 8 |  |  |  |  |  |
|  | L Temporal Fusiform Cortex, posterior division | 2.44 | -38 | -28 | -30 |
| 8 |  |  |  |  |  |
|  | L Inferior Temporal Gyrus, posterior division | 2.55 | -52 | -24 | -28 |
| 8 |  |  |  |  |  |
|  | L Temporal Pole | 2.48 | -38 | 8 | -28 |
| 8 |  |  |  |  |  |
|  | R Frontal Orbital Cortex | 2.31 | 10 | 22 | -26 |
| 8 |  |  |  |  |  |
|  | L Para hippocampal Gyrus, posterior division | 2.43 | -22 | -28 | -24 |
| 8 |  |  |  |  |  |
|  | L Parahippocampal Gyrus, anterior division | 2.33 | -20 | -20 | -24 |
| 8 |  |  |  |  |  |
|  | L Frontal Orbital Cortex | 2.33 | -36 | 20 | -24 |
| 8 |  |  |  |  |  |
|  | L Parahippocampal Gyrus, posterior division | 2.75 | -16 | -28 | -20 |
| 8 |  |  |  |  |  |
|  | L Brain-Stem | 2.60 | -2 | -32 | -18 |
| 8 |  |  |  |  |  |
|  | R Temporal Fusiform Cortex, posterior division | 2.77 | 40 | -30 | -18 |
| 8 |  |  |  |  |  |
|  | L Planum Polare | 2.74 | -40 | 0 | -18 |
| 8 |  |  |  |  |  |
|  | L Parahippocampal Gyrus, posterior division | 2.37 | -20 | -28 | -14 |
| 8 |  |  |  |  |  |
|  | R Insular Cortex | 2.42 | 40 | 14 | -14 |
| 8 |  |  |  |  |  |
|  | L Middle Temporal Gyrus, posterior division | 2.35 | -56 | -16 | -10 |
| 8 |  |  |  |  |  |
|  | L Pallidum | 2.37 | -14 | 0 | -6 |
| 8 |  |  |  |  |  |
|  | L Frontal Orbital Cortex | 2.41 | -44 | 34 | -6 |
| 8 |  |  |  |  |  |
|  | L Paracingulate Gyrus | 2.65 | -2 | 46 | -6 |
| 8 |  |  |  |  |  |
|  | L Cingulate Gyrus, anterior division | 2.40 | -8 | 38 | -2 |
| 8 |  |  |  |  |  |
|  | R Frontal Operculum Cortex | 2.54 | 48 | 12 | 4 |
| 8 |  |  |  |  |  |
|  | R Lateral Occipital Cortex, inferior division | 2.64 | 42 | -74 | 6 |
| 8 |  |  |  |  |  |
|  | L Caudate | 2.66 | -8 | 16 | 6 |
| 8 |  |  |  |  |  |
|  | R Cingulate Gyrus, anterior division | 2.42 | 6 | 40 | 6 |
| 8 |  |  |  |  |  |
|  | R Frontal Pole | 2.35 | 10 | 56 | 8 |
| 8 |  |  |  |  |  |
|  | R Thalamus | 2.55 | 4 | -4 | 10 |
| 8 |  |  |  |  |  |
|  | L Supramarginal Gyrus, posterior division | 2.44 | -50 | -48 | 14 |
| 8 |  |  |  |  |  |
|  | L Thalamus | 2.44 | -2 | -14 | 14 |
| 8 |  |  |  |  |  |
|  | L Frontal Pole | 2.46 | -8 | 62 | 14 |
| 8 |  |  |  |  |  |
|  | L Thalamus | 2.44 | -6 | -16 | 18 |
| 8 |  |  |  |  |  |
|  | L Lateral Occipital Cortex, superior division | 2.55 | -30 | -80 | 20 |
| 8 |  |  |  |  |  |
|  | L Cingulate Gyrus, posterior division | 2.33 | -4 | -42 | 22 |
| 8 |  |  |  |  |  |
|  | R Precuneus Cortex | 2.46 | 8 | -54 | 30 |
| 8 |  |  |  |  |  |
|  | R Supramarginal Gyrus, posterior division | 2.40 | 66 | -44 | 32 |
| 8 |  |  |  |  |  |
|  | L Supramarginal Gyrus, anterior division | 2.44 | -54 | -24 | 34 |
| 8 |  |  |  |  |  |
|  | R Paracingulate Gyrus | 2.39 | 12 | 20 | 34 |
| 8 |  |  |  |  |  |
|  | L Middle Frontal Gyrus | 2.41 | -34 | 34 | 34 |
| 8 |  |  |  |  |  |
|  | L Middle Frontal Gyrus | 2.30 | -36 | 36 | 36 |
| 8 |  |  |  |  |  |
|  | R Superior Frontal Gyrus | 2.30 | 20 | -2 | 58 |
| 8 |  |  |  |  |  |
|  | R Postcentral Gyrus | 2.41 | 38 | -32 | 60 |
| 8 |  |  |  |  |  |
|  | L Superior Frontal Gyrus | 2.43 | -4 | 24 | 62 |

# Supplementary Table S21. [CT_onset _> CS_onset_]. Descriptive statistics for clusters and local maxima showing greater activation for Certain-Threat relative to Certain-Safety anticipation for the OSP Onset regressor (FDR *q*<0.05, whole-brain corrected). 

| ***mm***^***3***^ | **Label** | ***t*** | ***x*** | ***y*** | ***z*** |
| --- | --- | --- | --- | --- | --- |
| 33,112 |  |  |  |  |  |
|  | R Inferior Temporal Gyrus, temporooccipital part | 5.58 | 50 | -54 | -18 |
|  | R Middle Temporal Gyrus, temporooccipital part | 4.37 | 58 | -56 | -8 |
|  | R Temporal Occipital Fusiform Cortex | 3.47 | 44 | -56 | -18 |
|  | R Lateral Occipital Cortex, inferior division | 6.50 | 48 | -74 | -8 |
|  | L Lingual Gyrus | 6.55 | -4 | -86 | -8 |
|  | L Occipital Fusiform Gyrus | 3.54 | -24 | -86 | -18 |
|  | R Lingual Gyrus | 6.75 | 8 | -88 | -8 |
|  | R Occipital Pole | 8.52 | 14 | -102 | 12 |
|  | L Occipital Pole | 9.29 | -12 | -104 | 2 |
| 3,440 |  |  |  |  |  |
|  | L Supramarginal Gyrus, posterior division | 5.87 | -36 | -50 | 42 |
|  | L Angular Gyrus | 3.75 | -52 | -56 | 34 |
|  | L Lateral Occipital Cortex, superior division | 4.63 | -48 | -64 | 46 |
| 3,384 |  |  |  |  |  |
|  | R Frontal Pole | 3.68 | 10 | 60 | 24 |
|  | R Superior Frontal Gyrus | 6.03 | 4 | 48 | 40 |
|  | L Paracingulate Gyrus | 4.13 | -4 | 36 | 38 |
|  | R Paracingulate Gyrus | 4.13 | 6 | 36 | 40 |
|  | L Superior Frontal Gyrus | 4.69 | -2 | 34 | 48 |
| 2,888 |  |  |  |  |  |
|  | L Frontal Pole | 5.43 | -30 | 66 | 2 |
| 2,520 |  |  |  |  |  |
|  | L Precuneus Cortex | 5.13 | -2 | -72 | 52 |
|  | R Precuneus Cortex | 4.43 | 0 | -74 | 40 |
| 1,728 |  |  |  |  |  |
|  | R Lateral Occipital Cortex, superior division | 5.36 | 32 | -70 | 34 |
| 1,072 |  |  |  |  |  |
|  | R Supramarginal Gyrus, posterior division | 4.09 | 38 | -40 | 38 |
|  | R Superior Parietal Lobule | 5.66 | 38 | -50 | 48 |
| 1,032 |  |  |  |  |  |
|  | R Frontal Pole | 5.18 | 50 | 42 | 12 |
|  | R Middle Frontal Gyrus | 4.00 | 46 | 32 | 20 |
| 800 |  |  |  |  |  |
|  | R Cingulate Gyrus, posterior division | 4.09 | 0 | -34 | 40 |
|  | L Cingulate Gyrus, posterior division | 3.24 | -2 | -40 | 38 |
|  | R Precuneus Cortex | 3.35 | 4 | -42 | 46 |
| 696 |  |  |  |  |  |
|  | L Frontal Pole | 4.09 | -46 | 40 | 16 |
|  | L Middle Frontal Gyrus | 3.13 | -44 | 38 | 30 |
| 632 |  |  |  |  |  |
|  | L Middle Frontal Gyrus | 4.61 | -38 | 34 | 38 |
| 552 |  |  |  |  |  |
|  | R Inferior Frontal Gyrus, pars opercularis | 3.92 | 50 | 12 | 26 |
|  | R Precentral Gyrus | 4.15 | 48 | 6 | 24 |
| 440 |  |  |  |  |  |
|  | R Frontal Pole | 3.98 | 32 | 36 | -12 |
|  | R Frontal Orbital Cortex | 4.37 | 26 | 30 | -16 |
| 440 |  |  |  |  |  |
|  | R Frontal Pole | 4.19 | 32 | 54 | 8 |
| 416 |  |  |  |  |  |
|  | R Frontal Pole | 4.03 | 42 | 50 | 6 |
| 312 |  |  |  |  |  |
|  | L Paracingulate Gyrus | 4.07 | -8 | 44 | 16 |
| 272 |  |  |  |  |  |
|  | R Amygdala | 4.34 | 20 | -4 | -16 |
|  | R Amygdala | 4.51 | 28 | -6 | -14 |
|  | R Amygdala | 3.76 | 22 | -10 | -12 |
| 240 |  |  |  |  |  |
|  | L Paracingulate Gyrus | 4.36 | -6 | 40 | 24 |
| 216 |  |  |  |  |  |
|  | R Brain-Stem | 3.93 | 14 | -26 | -18 |
| 208 |  |  |  |  |  |
|  | R Cingulate Gyrus, posterior division | 3.80 | 0 | -34 | 30 |
| 184 |  |  |  |  |  |
|  | L Cingulate Gyrus, posterior division | 3.31 | -2 | -24 | 32 |
|  | R Cingulate Gyrus, posterior division | 3.46 | 2 | -24 | 34 |
| 176 |  |  |  |  |  |
|  | L Frontal Orbital Cortex | 4.03 | -22 | 30 | -22 |
| 144 |  |  |  |  |  |
|  | R Precuneus Cortex | 5.00 | 16 | -60 | 28 |
| 136 |  |  |  |  |  |
|  | L Middle Temporal Gyrus, posterior division | 3.95 | -70 | -30 | -10 |
| 136 |  |  |  |  |  |
|  | R Middle Frontal Gyrus | 3.25 | 46 | 30 | 40 |
| 128 |  |  |  |  |  |
|  | R Frontal Pole | 4.01 | 8 | 72 | -4 |
| 120 |  |  |  |  |  |
|  | L Brain-Stem | 4.61 | -8 | -26 | -20 |
| 120 |  |  |  |  |  |
|  | R Brain-Stem | 3.39 | 0 | -36 | -4 |
| 120 |  |  |  |  |  |
|  | L Cingulate Gyrus, posterior division | 4.02 | -10 | -28 | 40 |
| 112 |  |  |  |  |  |
|  | L Frontal Pole | 2.96 | -22 | 50 | -14 |
| 104 |  |  |  |  |  |
|  | R Hippocampus | 4.44 | 26 | -38 | 0 |
| 104 |  |  |  |  |  |
|  | L Lingual Gyrus | 4.48 | -20 | -54 | -2 |
| 88 |  |  |  |  |  |
|  | R Frontal Pole | 4.25 | 46 | 54 | -4 |
| 80 |  |  |  |  |  |
|  | L Temporal Pole | 3.71 | -36 | 4 | -18 |
| 80 |  |  |  |  |  |
|  | L Amygdala | 4.08 | -20 | -10 | -14 |
|  | L Hippocampus/Amygdala | 3.15 | -22 | -12 | -16 |
| 80 |  |  |  |  |  |
|  | R Superior Temporal Gyrus, posterior division | 3.50 | 52 | -18 | -6 |
| 72 |  |  |  |  |  |
|  | R Inferior Temporal Gyrus, temporooccipital part | 3.67 | 58 | -42 | -22 |
| 72 |  |  |  |  |  |
|  | L Insular Cortex | 3.20 | -38 | -6 | -8 |
|  | L Planum Polare | 4.35 | -42 | -10 | -10 |
| 72 |  |  |  |  |  |
|  | R Frontal Medial Cortex | 3.47 | 2 | 48 | -10 |
| 72 |  |  |  |  |  |
|  | L Planum Polare | 4.54 | -52 | 2 | -6 |
| 72 |  |  |  |  |  |
|  | R Caudate | 4.08 | 8 | 12 | 10 |
| 72 |  |  |  |  |  |
|  | L Frontal Pole | 3.60 | -44 | 48 | 16 |
| 64 |  |  |  |  |  |
|  | R Parahippocampal Gyrus, anterior division | 3.04 | 18 | -2 | -38 |
| 64 |  |  |  |  |  |
|  | R Temporal Pole | 3.67 | 40 | 10 | -26 |
| 64 |  |  |  |  |  |
|  | R Hippocampus | 3.29 | 34 | -14 | -16 |
| 64 |  |  |  |  |  |
|  | R Frontal Pole | 3.87 | 6 | 66 | -16 |
| 64 |  |  |  |  |  |
|  | R Hippocampus | 3.46 | 28 | -24 | -12 |
| 64 |  |  |  |  |  |
|  | R Insular Cortex | 3.59 | 38 | -2 | -10 |
| 64 |  |  |  |  |  |
|  | R Insular Cortex | 3.73 | 38 | -6 | 4 |
| 56 |  |  |  |  |  |
|  | R Brain-Stem | 3.58 | 18 | -34 | -42 |
| 56 |  |  |  |  |  |
|  | L Subcallosal Cortex | 3.24 | -4 | 10 | -8 |
| 56 |  |  |  |  |  |
|  | L Paracingulate Gyrus | 3.35 | -10 | 48 | 8 |
| 56 |  |  |  |  |  |
|  | R Middle Frontal Gyrus | 3.43 | 30 | 30 | 36 |
| 48 |  |  |  |  |  |
|  | L Temporal Pole | 3.52 | -54 | 2 | -34 |
| 48 |  |  |  |  |  |
|  | L Frontal Medial Cortex | 3.65 | -8 | 34 | -26 |
| 48 |  |  |  |  |  |
|  | L Amygdala | 3.31 | -30 | -8 | -16 |
|  | L Amygdala | 3.32 | -28 | -10 | -14 |
| 48 |  |  |  |  |  |
|  | L Frontal Pole | 3.59 | -50 | 40 | -14 |
| 48 |  |  |  |  |  |
|  | L Insular Cortex | 3.78 | -40 | 0 | -10 |
| 48 |  |  |  |  |  |
|  | R Insular Cortex | 3.51 | 34 | 22 | -4 |
| 48 |  |  |  |  |  |
|  | R Frontal Pole | 3.69 | 40 | 62 | 6 |
| 48 |  |  |  |  |  |
|  | R Insular Cortex | 3.20 | 36 | -12 | 16 |
| 48 |  |  |  |  |  |
|  | R Precuneus Cortex | 3.27 | 2 | -52 | 18 |
|  | R Cingulate Gyrus, posterior division | 2.95 | 2 | -52 | 24 |
| 48 |  |  |  |  |  |
|  | R Precentral Gyrus | 3.37 | 44 | 4 | 34 |
| 48 |  |  |  |  |  |
|  | R Supramarginal Gyrus, anterior division | 3.14 | 54 | -32 | 48 |
|  | R Supramarginal Gyrus, posterior division | 3.21 | 56 | -36 | 54 |
| 40 |  |  |  |  |  |
|  | L Temporal Pole | 3.32 | -30 | 4 | -28 |
| 40 |  |  |  |  |  |
|  | R Frontal Orbital Cortex | 3.32 | 10 | 28 | -26 |
| 40 |  |  |  |  |  |
|  | R Frontal Orbital Cortex | 3.16 | 20 | 28 | -16 |
| 40 |  |  |  |  |  |
|  | R Frontal Orbital Cortex | 3.36 | 26 | 18 | -18 |
| 40 |  |  |  |  |  |
|  | R Paracingulate Gyrus | 3.20 | 6 | 42 | -6 |
| 40 |  |  |  |  |  |
|  | R Lingual Gyrus | 3.33 | 20 | -52 | 0 |
| 40 |  |  |  |  |  |
|  | L Hippocampus | 3.31 | -18 | -38 | 0 |
| 40 |  |  |  |  |  |
|  | R Thalamus | 3.26 | 18 | -36 | 2 |
| 40 |  |  |  |  |  |
|  | R Frontal Pole | 3.10 | 28 | 66 | 0 |
| 40 |  |  |  |  |  |
|  | R Lateral Occipital Cortex, superior division | 3.71 | 42 | -70 | 26 |
| 40 |  |  |  |  |  |
|  | L Lateral Occipital Cortex, superior division | 3.27 | -44 | -64 | 54 |
| 32 |  |  |  |  |  |
|  | L Brain-Stem | 3.22 | -4 | -42 | -50 |
| 32 |  |  |  |  |  |
|  | L Temporal Fusiform Cortex, anterior division | 3.95 | -30 | -8 | -44 |
| 32 |  |  |  |  |  |
|  | R Brain-Stem | 3.49 | 12 | -28 | -34 |
| 32 |  |  |  |  |  |
|  | R Temporal Pole | 3.21 | 34 | 18 | -34 |
| 32 |  |  |  |  |  |
|  | L Inferior Temporal Gyrus, posterior division | 3.58 | -52 | -18 | -32 |
| 32 |  |  |  |  |  |
|  | L Inferior Temporal Gyrus, temporooccipital part | 3.05 | -62 | -50 | -18 |
| 32 |  |  |  |  |  |
|  | L Frontal Medial Cortex | 3.29 | -4 | 36 | -18 |
| 32 |  |  |  |  |  |
|  | R Thalamus | 3.62 | 6 | -26 | 2 |
| 32 |  |  |  |  |  |
|  | R Frontal Pole | 3.23 | 50 | 46 | 4 |
| 32 |  |  |  |  |  |
|  | L Planum Temporale | 3.12 | -56 | -20 | 4 |
| 32 |  |  |  |  |  |
|  | L Cingulate Gyrus, posterior division | 3.64 | -6 | -46 | 10 |
| 32 |  |  |  |  |  |
|  | R Frontal Pole | 2.89 | 2 | 68 | 10 |
| 32 |  |  |  |  |  |
|  | L Frontal Pole | 3.25 | -40 | 58 | 12 |
| 32 |  |  |  |  |  |
|  | L Occipital Pole | 3.43 | -2 | -96 | 24 |
| 32 |  |  |  |  |  |
|  | R Occipital Pole | 3.58 | 2 | -92 | 26 |
| 32 |  |  |  |  |  |
|  | R Supramarginal Gyrus, posterior division | 3.51 | 60 | -42 | 36 |
| 32 |  |  |  |  |  |
|  | L Lateral Occipital Cortex, superior division | 3.28 | -34 | -62 | 40 |
| 32 |  |  |  |  |  |
|  | R Middle Frontal Gyrus | 3.49 | 34 | 6 | 56 |
| 32 |  |  |  |  |  |
|  | R Lateral Occipital Cortex, superior division | 3.42 | 36 | -64 | 60 |
| 24 |  |  |  |  |  |
|  | R Brain-Stem | 3.44 | 0 | -44 | -64 |
| 24 |  |  |  |  |  |
|  | R Temporal Fusiform Cortex, anterior division | 2.97 | 30 | -6 | -42 |
| 24 |  |  |  |  |  |
|  | R Temporal Pole | 3.36 | 54 | 12 | -34 |
| 24 |  |  |  |  |  |
|  | L Hippocampus | 3.16 | -30 | -10 | -24 |
| 24 |  |  |  |  |  |
|  | R Temporal Pole | 3.37 | 28 | 6 | -24 |
| 24 |  |  |  |  |  |
|  | L Temporal Pole | 3.57 | -44 | 10 | -22 |
| 24 |  |  |  |  |  |
|  | R Frontal Orbital Cortex | 2.95 | 26 | 28 | -24 |
| 24 |  |  |  |  |  |
|  | R Brain-Stem | 3.05 | 0 | -20 | -22 |
| 24 |  |  |  |  |  |
|  | L Hippocampus | 3.32 | -20 | -12 | -22 |
| 24 |  |  |  |  |  |
|  | R Frontal Medial Cortex | 3.18 | 0 | 48 | -20 |
| 24 |  |  |  |  |  |
|  | R Frontal Orbital Cortex | 3.03 | 34 | 30 | -18 |
| 24 |  |  |  |  |  |
|  | L Brain-Stem | 3.17 | -4 | -34 | -12 |
| 24 |  |  |  |  |  |
|  | R Frontal Pole | 3.60 | 26 | 42 | -14 |
| 24 |  |  |  |  |  |
|  | L Hippocampus | 3.12 | -28 | -36 | -8 |
| 24 |  |  |  |  |  |
|  | R Middle Temporal Gyrus, posterior division | 3.05 | 68 | -16 | -6 |
| 24 |  |  |  |  |  |
|  | R Planum Polare | 3.48 | 48 | -8 | -6 |
| 24 |  |  |  |  |  |
|  | R Frontal Pole | 3.46 | 28 | 56 | 0 |
| 24 |  |  |  |  |  |
|  | R Insular Cortex | 2.89 | 40 | -14 | 4 |
| 24 |  |  |  |  |  |
|  | R Thalamus | 3.04 | 6 | -16 | 12 |
| 24 |  |  |  |  |  |
|  | L Central Opercular Cortex | 3.23 | -58 | -8 | 12 |
| 24 |  |  |  |  |  |
|  | R Angular Gyrus | 3.10 | 52 | -44 | 32 |
| 24 |  |  |  |  |  |
|  | R Cingulate Gyrus, anterior division | 3.25 | 2 | -4 | 34 |
| 24 |  |  |  |  |  |
|  | R Middle Frontal Gyrus | 3.24 | 28 | 32 | 42 |
| 24 |  |  |  |  |  |
|  | R Cingulate Gyrus, posterior division | 3.45 | 6 | -22 | 42 |
| 16 |  |  |  |  |  |
|  | R Brain-Stem | 3.04 | 0 | -34 | -42 |
| 16 |  |  |  |  |  |
|  | R Temporal Pole | 3.03 | 42 | 8 | -38 |
| 16 |  |  |  |  |  |
|  | L Temporal Pole | 2.98 | -30 | 12 | -40 |
| 16 |  |  |  |  |  |
|  | L Inferior Temporal Gyrus, posterior division | 3.15 | -64 | -20 | -28 |
| 16 |  |  |  |  |  |
|  | R Temporal Pole | 2.95 | 30 | 10 | -30 |
| 16 |  |  |  |  |  |
|  | L Brain-Stem | 2.96 | -2 | -28 | -26 |
| 16 |  |  |  |  |  |
|  | L Frontal Pole | 3.05 | -8 | 44 | -24 |
| 16 |  |  |  |  |  |
|  | R Hippocampus | 2.96 | 28 | -18 | -20 |
| 16 |  |  |  |  |  |
|  | R Frontal Orbital Cortex | 2.93 | 14 | 22 | -18 |
| 16 |  |  |  |  |  |
|  | L Amygdala | 3.19 | -18 | -6 | -16 |
| 16 |  |  |  |  |  |
|  | R Insular Cortex | 2.94 | 36 | 6 | -16 |
| 16 |  |  |  |  |  |
|  | R Frontal Medial Cortex | 2.87 | 0 | 38 | -16 |
| 16 |  |  |  |  |  |
|  | L Frontal Pole | 3.01 | -24 | 42 | -16 |
| 16 |  |  |  |  |  |
|  | L Frontal Pole | 3.40 | -32 | 42 | -16 |
| 16 |  |  |  |  |  |
|  | L Hippocampus | 3.00 | -34 | -22 | -14 |
| 16 |  |  |  |  |  |
|  | L Hippocampus | 3.00 | -28 | -28 | -12 |
| 16 |  |  |  |  |  |
|  | L Frontal Pole | 3.10 | -22 | 44 | -12 |
| 16 |  |  |  |  |  |
|  | L Frontal Pole | 3.17 | -14 | 64 | -4 |
| 16 |  |  |  |  |  |
|  | R Superior Temporal Gyrus, posterior division | 2.92 | 62 | -22 | -2 |
| 16 |  |  |  |  |  |
|  | R Frontal Pole | 3.18 | 40 | 44 | -2 |
| 16 |  |  |  |  |  |
|  | R Planum Temporale | 3.03 | 58 | -12 | 6 |
| 16 |  |  |  |  |  |
|  | R Thalamus | 2.90 | 4 | -6 | 10 |
| 16 |  |  |  |  |  |
|  | L Insular Cortex | 3.66 | -38 | -6 | 12 |
| 16 |  |  |  |  |  |
|  | L Thalamus | 4.50 | -4 | -8 | 12 |
| 16 |  |  |  |  |  |
|  | R Central Opercular Cortex | 3.77 | 54 | -10 | 14 |
| 16 |  |  |  |  |  |
|  | R Paracingulate Gyrus | 3.01 | 10 | 46 | 14 |
| 16 |  |  |  |  |  |
|  | R Frontal Pole | 3.18 | 28 | 54 | 14 |
| 16 |  |  |  |  |  |
|  | L Frontal Pole | 2.99 | -22 | 54 | 16 |
| 16 |  |  |  |  |  |
|  | R Occipital Pole | 3.02 | 4 | -98 | 18 |
| 16 |  |  |  |  |  |
|  | L Precuneus Cortex | 2.96 | -16 | -64 | 28 |
| 16 |  |  |  |  |  |
|  | R Cingulate Gyrus, anterior division | 3.05 | 0 | -12 | 30 |
| 16 |  |  |  |  |  |
|  | L Paracingulate Gyrus | 3.31 | -8 | 34 | 30 |
| 16 |  |  |  |  |  |
|  | R Cuneal Cortex | 3.64 | 2 | -88 | 34 |
| 16 |  |  |  |  |  |
|  | L Precuneus Cortex | 3.02 | -6 | -70 | 34 |
| 16 |  |  |  |  |  |
|  | R Middle Frontal Gyrus | 3.94 | 38 | 14 | 36 |
| 16 |  |  |  |  |  |
|  | R Precentral Gyrus | 3.30 | 52 | -4 | 42 |
| 16 |  |  |  |  |  |
|  | R Angular Gyrus | 2.94 | 54 | -56 | 50 |
| 8 |  |  |  |  |  |
|  | L Brain-Stem | 2.92 | -6 | -48 | -50 |
| 8 |  |  |  |  |  |
|  | R Brain-Stem | 2.88 | 10 | -42 | -46 |
| 8 |  |  |  |  |  |
|  | L Temporal Pole | 2.93 | -36 | 14 | -44 |
| 8 |  |  |  |  |  |
|  | R Temporal Pole | 3.03 | 28 | 10 | -42 |
| 8 |  |  |  |  |  |
|  | L Brain-Stem | 2.90 | -4 | -20 | -36 |
| 8 |  |  |  |  |  |
|  | L Parahippocampal Gyrus, anterior division | 2.95 | -22 | -8 | -36 |
| 8 |  |  |  |  |  |
|  | L Parahippocampal Gyrus, anterior division | 2.90 | -22 | 2 | -36 |
| 8 |  |  |  |  |  |
|  | R Temporal Pole | 2.88 | 42 | 20 | -36 |
| 8 |  |  |  |  |  |
|  | L Parahippocampal Gyrus, anterior division | 3.05 | -24 | -4 | -34 |
| 8 |  |  |  |  |  |
|  | L Parahippocampal Gyrus, anterior division | 2.89 | -28 | -4 | -34 |
| 8 |  |  |  |  |  |
|  | R Parahippocampal Gyrus, anterior division | 2.87 | 26 | -2 | -34 |
| 8 |  |  |  |  |  |
|  | R Brain-Stem | 2.85 | 0 | -16 | -32 |
| 8 |  |  |  |  |  |
|  | R Temporal Pole | 2.99 | 28 | 16 | -32 |
| 8 |  |  |  |  |  |
|  | R Temporal Pole | 3.06 | 42 | 12 | -30 |
| 8 |  |  |  |  |  |
|  | R Brain-Stem | 2.91 | 18 | -22 | -28 |
| 8 |  |  |  |  |  |
|  | R Amygdala | 2.93 | 26 | 2 | -28 |
|  | R Parahippocampal Gyrus, anterior division | 2.93 | 26 | 2 | -28 |
| 8 |  |  |  |  |  |
|  | R Parahippocampal Gyrus, posterior division | 2.86 | 22 | -24 | -26 |
| 8 |  |  |  |  |  |
|  | R Temporal Fusiform Cortex, posterior division | 2.90 | 38 | -14 | -26 |
| 8 |  |  |  |  |  |
|  | R Temporal Pole | 3.04 | 34 | 6 | -26 |
| 8 |  |  |  |  |  |
|  | R Subcallosal Cortex | 3.10 | 2 | 30 | -24 |
| 8 |  |  |  |  |  |
|  | L Frontal Pole | 2.89 | -6 | 60 | -24 |
| 8 |  |  |  |  |  |
|  | L Middle Temporal Gyrus, posterior division | 3.01 | -64 | -14 | -22 |
| 8 |  |  |  |  |  |
|  | L Frontal Orbital Cortex | 2.89 | -12 | 28 | -22 |
| 8 |  |  |  |  |  |
|  | L Frontal Medial Cortex | 2.88 | -10 | 34 | -22 |
| 8 |  |  |  |  |  |
|  | L Inferior Temporal Gyrus, posterior division | 3.30 | -56 | -42 | -20 |
| 8 |  |  |  |  |  |
|  | L Amygdala | 2.94 | -32 | 2 | -20 |
| 8 |  |  |  |  |  |
|  | R Amygdala | 3.01 | 26 | 0 | -18 |
| 8 |  |  |  |  |  |
|  | L Frontal Orbital Cortex | 2.94 | -30 | 14 | -18 |
| 8 |  |  |  |  |  |
|  | R Temporal Pole | 2.98 | 52 | 12 | -16 |
| 8 |  |  |  |  |  |
|  | L Hippocampus | 3.15 | -34 | -26 | -14 |
| 8 |  |  |  |  |  |
|  | L Frontal Pole | 2.92 | -28 | 58 | -14 |
| 8 |  |  |  |  |  |
|  | R Middle Temporal Gyrus, posterior division | 2.89 | 64 | -26 | -12 |
| 8 |  |  |  |  |  |
|  | R Frontal Pole | 2.95 | 38 | 56 | -10 |
| 8 |  |  |  |  |  |
|  | R Brain-Stem | 3.20 | 10 | -26 | -8 |
| 8 |  |  |  |  |  |
|  | L Middle Temporal Gyrus, posterior division | 2.86 | -70 | -24 | -6 |
| 8 |  |  |  |  |  |
|  | R Pallidum | 2.86 | 18 | 2 | -6 |
| 8 |  |  |  |  |  |
|  | R Planum Polare | 3.26 | 44 | -16 | -4 |
| 8 |  |  |  |  |  |
|  | R Superior Temporal Gyrus, anterior division | 2.96 | 64 | 0 | -4 |
| 8 |  |  |  |  |  |
|  | R Putamen | 2.90 | 28 | 14 | -2 |
| 8 |  |  |  |  |  |
|  | R Frontal Pole | 2.85 | 22 | 64 | -2 |
| 8 |  |  |  |  |  |
|  | L Hippocampus | 3.17 | -24 | -36 | 0 |
| 8 |  |  |  |  |  |
|  | L Thalamus | 2.98 | -2 | -4 | 0 |
| 8 |  |  |  |  |  |
|  | R Superior Temporal Gyrus, anterior division | 2.93 | 64 | 0 | 0 |
| 8 |  |  |  |  |  |
|  | R Insular Cortex | 2.92 | 34 | 22 | 0 |
| 8 |  |  |  |  |  |
|  | L Insular Cortex | 2.85 | -28 | 26 | 0 |
| 8 |  |  |  |  |  |
|  | R Frontal Pole | 2.92 | 22 | 58 | 0 |
| 8 |  |  |  |  |  |
|  | R Frontal Pole | 2.85 | 22 | 70 | 0 |
| 8 |  |  |  |  |  |
|  | L Frontal Pole | 2.93 | -22 | 68 | 4 |
| 8 |  |  |  |  |  |
|  | R Cingulate Gyrus, posterior division | 2.94 | 8 | -42 | 6 |
| 8 |  |  |  |  |  |
|  | R Thalamus | 2.95 | 10 | -24 | 6 |
| 8 |  |  |  |  |  |
|  | L Thalamus | 2.97 | -10 | -32 | 10 |
| 8 |  |  |  |  |  |
|  | L Planum Temporale | 3.19 | -44 | -30 | 10 |
| 8 |  |  |  |  |  |
|  | R Frontal Pole | 2.86 | 36 | 60 | 10 |
| 8 |  |  |  |  |  |
|  | R Cingulate Gyrus, posterior division | 2.94 | 6 | -44 | 12 |
| 8 |  |  |  |  |  |
|  | L Central Opercular Cortex | 2.97 | -52 | -14 | 12 |
| 8 |  |  |  |  |  |
|  | L Planum Temporale | 2.88 | -48 | -36 | 14 |
| 8 |  |  |  |  |  |
|  | R Thalamus | 2.95 | 16 | -22 | 14 |
| 8 |  |  |  |  |  |
|  | R Cingulate Gyrus, posterior division | 2.92 | 4 | -48 | 16 |
| 8 |  |  |  |  |  |
|  | R Central Opercular Cortex | 2.86 | 42 | -12 | 16 |
| 8 |  |  |  |  |  |
|  | R Frontal Pole | 2.96 | 2 | 66 | 18 |
| 8 |  |  |  |  |  |
|  | L Cuneal Cortex | 3.37 | -2 | -86 | 20 |
| 8 |  |  |  |  |  |
|  | R Frontal Pole | 3.19 | 2 | 64 | 26 |
| 8 |  |  |  |  |  |
|  | R Cingulate Gyrus, posterior division | 3.09 | 8 | -34 | 28 |
| 8 |  |  |  |  |  |
|  | L Supramarginal Gyrus, anterior division | 2.86 | -56 | -32 | 28 |
| 8 |  |  |  |  |  |
|  | R Cingulate Gyrus, anterior division | 2.87 | 4 | 8 | 28 |
| 8 |  |  |  |  |  |
|  | R Middle Frontal Gyrus | 2.86 | 56 | 20 | 34 |
| 8 |  |  |  |  |  |
|  | L Cingulate Gyrus, posterior division | 2.90 | -8 | -42 | 40 |
| 8 |  |  |  |  |  |
|  | L Middle Frontal Gyrus | 3.46 | -44 | 22 | 40 |
| 8 |  |  |  |  |  |
|  | R Superior Parietal Lobule | 2.92 | 30 | -42 | 42 |
| 8 |  |  |  |  |  |
|  | R Precuneus Cortex | 3.16 | 12 | -48 | 46 |
| 8 |  |  |  |  |  |
|  | R Supramarginal Gyrus, posterior division | 2.91 | 48 | -36 | 46 |
| 8 |  |  |  |  |  |
|  | R Superior Frontal Gyrus | 2.96 | 22 | 10 | 54 |
| 8 |  |  |  |  |  |
|  | R Precuneus Cortex | 2.88 | 4 | -50 | 70 |

# Supplementary Table S22. [UT_onset _> US_onset_] $\boldsymbol{∩}$ [CT_onset _> CS_onset_].  Descriptive statistics for clusters and local maxima showing greater activation for Uncertain-Threat relative to Uncertain-Safety anticipation for the OSP Onset regressor AND greater activation for Certain-Threat relative to Certain-Safety anticipation for the OSP Onset regressor (FDR *q*<0.05, whole-brain corrected). 

| ***mm***^***3***^ | **Label** | ***t*** | ***x*** | ***y*** | ***z*** |
| --- | --- | --- | --- | --- | --- |
| 28,256 |  |  |  |  |  |
|  | R Inferior Temporal Gyrus, temporooccipital part | 5.58 | 50 | -54 | -18 |
|  | R Temporal Occipital Fusiform Cortex | 3.47 | 44 | -56 | -18 |
|  | R Middle Temporal Gyrus, temporooccipital part | 3.52 | 50 | -58 | 6 |
|  | R Occipital Fusiform Gyrus | 3.85 | 40 | -70 | -18 |
|  | R Lateral Occipital Cortex, inferior division | 6.03 | 48 | -74 | -8 |
|  | L Lingual Gyrus | 6.55 | -4 | -86 | -8 |
|  | L Occipital Fusiform Gyrus | 3.54 | -24 | -86 | -18 |
|  | R Lingual Gyrus | 6.75 | 8 | -88 | -8 |
|  | R Occipital Pole | 7.87 | 14 | -100 | 12 |
|  | L Occipital Pole | 8.68 | -12 | -102 | 2 |
| 2,624 |  |  |  |  |  |
|  | R Superior Frontal Gyrus | 6.03 | 4 | 48 | 40 |
|  | R Paracingulate Gyrus | 5.32 | 6 | 44 | 34 |
|  | L Superior Frontal Gyrus | 4.08 | -6 | 38 | 36 |
|  | L Paracingulate Gyrus | 4.13 | -4 | 36 | 38 |
| 2,472 |  |  |  |  |  |
|  | L Precuneus Cortex | 5.13 | -2 | -72 | 52 |
|  | R Precuneus Cortex | 3.71 | 0 | -76 | 42 |
| 1,664 |  |  |  |  |  |
|  | R Lateral Occipital Cortex, superior division | 5.36 | 32 | -70 | 34 |
| 1,112 |  |  |  |  |  |
|  | L Supramarginal Gyrus, posterior division | 4.55 | -40 | -46 | 42 |
| 952 |  |  |  |  |  |
|  | R Supramarginal Gyrus, posterior division | 3.65 | 38 | -38 | 38 |
|  | R Superior Parietal Lobule | 4.78 | 40 | -50 | 48 |
| 648 |  |  |  |  |  |
|  | R Frontal Pole | 4.32 | 54 | 38 | 16 |
|  | R Middle Frontal Gyrus | 4 | 46 | 32 | 20 |
| 576 |  |  |  |  |  |
|  | R Cingulate Gyrus, posterior division | 3.39 | 8 | -40 | 34 |
|  | R Precuneus Cortex | 3.63 | 6 | -46 | 40 |
| 552 |  |  |  |  |  |
|  | R Inferior Frontal Gyrus, pars opercular is | 3.92 | 50 | 12 | 26 |
|  | R Precentral Gyrus | 4.15 | 48 | 6 | 24 |
| 352 |  |  |  |  |  |
|  | R Frontal Pole | 3.41 | 30 | 36 | -12 |
|  | R Frontal Orbital Cortex | 4.01 | 26 | 30 | -16 |
| 168 |  |  |  |  |  |
|  | R Middle Temporal Gyrus, temporooccipital part | 3.11 | 62 | -42 | -12 |
| 160 |  |  |  |  |  |
|  | L Paracingulate Gyrus | 3.39 | -6 | 44 | 16 |
| 136 |  |  |  |  |  |
|  | R Precuneus Cortex | 4.26 | 16 | -60 | 28 |
| 104 |  |  |  |  |  |
|  | L Lingual Gyrus | 4.48 | -20 | -54 | -2 |
| 96 |  |  |  |  |  |
|  | R Hippocampus | 4.44 | 26 | -38 | 0 |
| 88 |  |  |  |  |  |
|  | R Frontal Pole | 3.67 | 4 | 72 | -6 |
| 80 |  |  |  |  |  |
|  | L Frontal Orbital Cortex | 3.95 | -24 | 30 | -20 |
| 80 |  |  |  |  |  |
|  | R Brain-Stem | 2.93 | 0 | -36 | -6 |
| 80 |  |  |  |  |  |
|  | L Cingulate Gyrus, posterior division | 3.63 | -6 | -28 | 42 |
| 72 |  |  |  |  |  |
|  | R Amygdala | 3.68 | 18 | -6 | -16 |
| 64 |  |  |  |  |  |
|  | R Frontal Pole | 3.49 | 6 | 68 | -16 |
| 64 |  |  |  |  |  |
|  | R Hippocampus | 3.46 | 28 | -24 | -12 |
| 56 |  |  |  |  |  |
|  | L Subcallosal Cortex | 3.24 | -4 | 10 | -8 |
| 56 |  |  |  |  |  |
|  | L Paracingulate Gyrus | 3.35 | -10 | 48 | 8 |
| 56 |  |  |  |  |  |
|  | L Paracingulate Gyrus | 3.02 | -8 | 42 | 24 |
| 48 |  |  |  |  |  |
|  | R Frontal Medial Cortex | 3.38 | 2 | 50 | -12 |
| 48 |  |  |  |  |  |
|  | R Insular Cortex | 3.51 | 34 | 22 | -4 |
| 48 |  |  |  |  |  |
|  | R Caudate | 3.21 | 10 | 12 | 8 |
| 48 |  |  |  |  |  |
|  | R Cingulate Gyrus, posterior division | 3.04 | 2 | -52 | 20 |
| 48 |  |  |  |  |  |
|  | R Cingulate Gyrus, posterior division | 3.08 | 0 | -40 | 26 |
| 48 |  |  |  |  |  |
|  | R Precentral Gyrus | 3.37 | 44 | 4 | 34 |
| 48 |  |  |  |  |  |
|  | R Cingulate Gyrus, posterior division | 3.23 | 4 | -32 | 34 |
| 40 |  |  |  |  |  |
|  | R Frontal Orbital Cortex | 3.32 | 10 | 28 | -26 |
| 40 |  |  |  |  |  |
|  | R Right Hippocampus | 3.28 | 34 | -14 | -18 |
| 40 |  |  |  |  |  |
|  | R Lingual Gyrus | 3.33 | 20 | -52 | 0 |
| 40 |  |  |  |  |  |
|  | L Left Hippocampus | 3.31 | -18 | -38 | 0 |
| 40 |  |  |  |  |  |
|  | R Lateral Occipital Cortex, superior division | 3.71 | 42 | -70 | 26 |
| 32 |  |  |  |  |  |
|  | L Temporal Fusiform Cortex, anterior division | 3.95 | -30 | -8 | -44 |
| 32 |  |  |  |  |  |
|  | R Temporal Pole | 3.21 | 34 | 18 | -34 |
| 32 |  |  |  |  |  |
|  | R Temporal Pole | 3.43 | 40 | 10 | -24 |
| 32 |  |  |  |  |  |
|  | L Brain-Stem | 2.79 | -6 | -24 | -22 |
| 32 |  |  |  |  |  |
|  | R Frontal Orbital Cortex | 2.9 | 20 | 26 | -20 |
| 32 |  |  |  |  |  |
|  | R Amygdala | 3.33 | 28 | -4 | -14 |
| 32 |  |  |  |  |  |
|  | R Thalamus | 3.26 | 18 | -36 | 2 |
| 32 |  |  |  |  |  |
|  | R Occipital Pole | 3.03 | 2 | -92 | 26 |
| 32 |  |  |  |  |  |
|  | L Cingulate Gyrus, posterior division | 3.02 | -2 | -22 | 32 |
| 24 |  |  |  |  |  |
|  | R Temporal Fusiform Cortex, anterior division | 2.97 | 30 | -6 | -42 |
| 24 |  |  |  |  |  |
|  | R Inferior Temporal Gyrus, temporooccipital part | 3.06 | 56 | -42 | -22 |
| 24 |  |  |  |  |  |
|  | R Brain-Stem | 3.05 | 0 | -20 | -22 |
| 24 |  |  |  |  |  |
|  | R Frontal Orbital Cortex | 3.15 | 24 | 18 | -18 |
| 24 |  |  |  |  |  |
|  | L Hippocampus | 3 | -28 | -36 | -6 |
| 24 |  |  |  |  |  |
|  | L Cingulate Gyrus, posterior division | 3.64 | -6 | -46 | 10 |
| 24 |  |  |  |  |  |
|  | L Paracingulate Gyrus | 2.71 | -4 | 40 | 28 |
| 24 |  |  |  |  |  |
|  | R Cingulate Gyrus, anterior division | 3.21 | 2 | -4 | 34 |
| 16 |  |  |  |  |  |
|  | R Temporal Pole | 3.21 | 52 | 12 | -32 |
| 16 |  |  |  |  |  |
|  | L Temporal Pole | 3.18 | -30 | 4 | -28 |
| 16 |  |  |  |  |  |
|  | R Temporal Pole | 2.95 | 30 | 10 | -30 |
| 16 |  |  |  |  |  |
|  | R Hippocampus | 2.96 | 28 | -18 | -20 |
| 16 |  |  |  |  |  |
|  | R Frontal Medial Cortex | 2.8 | 0 | 50 | -20 |
| 16 |  |  |  |  |  |
|  | R Frontal Orbital Cortex | 2.93 | 14 | 22 | -18 |
| 16 |  |  |  |  |  |
|  | R Frontal Orbital Cortex | 2.98 | 36 | 32 | -18 |
| 16 |  |  |  |  |  |
|  | R Brain-Stem | 3.1 | 6 | -30 | -14 |
| 16 |  |  |  |  |  |
|  | L Amygdala | 2.46 | -30 | -10 | -16 |
| 16 |  |  |  |  |  |
|  | R Frontal Medial Cortex | 2.82 | 0 | 38 | -16 |
| 16 |  |  |  |  |  |
|  | L Hippocampus | 3 | -28 | -28 | -12 |
| 16 |  |  |  |  |  |
|  | L Frontal Pole | 2.46 | -28 | 66 | 8 |
| 16 |  |  |  |  |  |
|  | R Paracingulate Gyrus | 3.01 | 10 | 46 | 14 |
| 16 |  |  |  |  |  |
|  | R Occipital Pole | 3.02 | 4 | -98 | 18 |
| 16 |  |  |  |  |  |
|  | L Precuneus Cortex | 2.96 | -16 | -64 | 28 |
| 16 |  |  |  |  |  |
|  | L Cingulate Gyrus, anterior division | 2.88 | -4 | 0 | 28 |
| 16 |  |  |  |  |  |
|  | R Cuneal Cortex | 3.44 | 2 | -88 | 34 |
| 16 |  |  |  |  |  |
|  | L Precuneus Cortex | 3.02 | -6 | -70 | 34 |
| 16 |  |  |  |  |  |
|  | R Cingulate Gyrus, posterior division | 2.89 | 6 | -22 | 42 |
| 8 |  |  |  |  |  |
|  | R Temporal Pole | 2.48 | 28 | 10 | -42 |
| 8 |  |  |  |  |  |
|  | L Temporal Pole | 2.42 | -32 | 14 | -40 |
| 8 |  |  |  |  |  |
|  | R Para hippocampal Gyrus, anterior division | 2.91 | 28 | -10 | -38 |
| 8 |  |  |  |  |  |
|  | R Temporal Pole | 3.03 | 42 | 8 | -38 |
| 8 |  |  |  |  |  |
|  | L Parahippocampal Gyrus, anterior division | 2.74 | -22 | -8 | -36 |
| 8 |  |  |  |  |  |
|  | R Temporal Pole | 2.71 | 42 | 20 | -36 |
| 8 |  |  |  |  |  |
|  | R Parahippocampal Gyrus, anterior division | 2.89 | 22 | -4 | -34 |
| 8 |  |  |  |  |  |
|  | L Parahippocampal Gyrus, anterior division | 2.8 | -24 | -4 | -34 |
| 8 |  |  |  |  |  |
|  | L Parahippocampal Gyrus, anterior division | 2.89 | -28 | -4 | -34 |
| 8 |  |  |  |  |  |
|  | R Parahippocampal Gyrus, anterior division | 2.87 | 26 | -2 | -34 |
| 8 |  |  |  |  |  |
|  | R Temporal Pole | 2.99 | 28 | 16 | -32 |
| 8 |  |  |  |  |  |
|  | R Temporal Pole | 3.06 | 42 | 12 | -30 |
| 8 |  |  |  |  |  |
|  | R Temporal Occipital Fusiform Cortex | 3.26 | 44 | -42 | -26 |
| 8 |  |  |  |  |  |
|  | R Frontal Orbital Cortex | 2.49 | 26 | 28 | -24 |
| 8 |  |  |  |  |  |
|  | L Frontal Medial Cortex | 2.51 | -10 | 34 | -22 |
| 8 |  |  |  |  |  |
|  | L Inferior Temporal Gyrus, posterior division | 2.77 | -56 | -42 | -20 |
| 8 |  |  |  |  |  |
|  | L Frontal Pole | 2.37 | -16 | 44 | -20 |
| 8 |  |  |  |  |  |
|  | L Frontal Pole | 2.56 | -18 | 48 | -20 |
| 8 |  |  |  |  |  |
|  | R Lateral Occipital Cortex, inferior division | 3.04 | 48 | -64 | -18 |
| 8 |  |  |  |  |  |
|  | L Frontal Orbital Cortex | 2.94 | -30 | 14 | -18 |
| 8 |  |  |  |  |  |
|  | L Amygdala | 2.64 | -18 | -6 | -16 |
| 8 |  |  |  |  |  |
|  | R Temporal Pole | 2.98 | 52 | 12 | -16 |
| 8 |  |  |  |  |  |
|  | L Hippocampus | 2.36 | -34 | -22 | -12 |
| 8 |  |  |  |  |  |
|  | R Superior Temporal Gyrus, posterior division | 2.89 | 60 | -22 | -2 |
| 8 |  |  |  |  |  |
|  | L Thalamus | 2.94 | -2 | -22 | -2 |
| 8 |  |  |  |  |  |
|  | L Hippocampus | 3.17 | -24 | -36 | 0 |
| 8 |  |  |  |  |  |
|  | R Insular Cortex | 2.92 | 34 | 22 | 0 |
| 8 |  |  |  |  |  |
|  | R Cingulate Gyrus, posterior division | 2.94 | 8 | -42 | 6 |
| 8 |  |  |  |  |  |
|  | L Thalamus | 2.58 | -10 | -32 | 10 |
| 8 |  |  |  |  |  |
|  | R Cingulate Gyrus, posterior division | 2.94 | 6 | -44 | 12 |
| 8 |  |  |  |  |  |
|  | R Cingulate Gyrus, posterior division | 2.46 | 4 | -48 | 16 |
| 8 |  |  |  |  |  |
|  | L Cuneal Cortex | 2.87 | -2 | -86 | 20 |
| 8 |  |  |  |  |  |
|  | R Frontal Pole | 2.89 | 2 | 64 | 26 |
| 8 |  |  |  |  |  |
|  | R Cingulate Gyrus, anterior division | 2.41 | 4 | 8 | 28 |
| 8 |  |  |  |  |  |
|  | L Cingulate Gyrus, anterior division | 2.98 | -2 | -12 | 30 |
| 8 |  |  |  |  |  |
|  | R Middle Frontal Gyrus | 2.99 | 38 | 14 | 34 |
| 8 |  |  |  |  |  |
|  | R Middle Frontal Gyrus | 2.86 | 56 | 20 | 34 |
| 8 |  |  |  |  |  |
|  | R Superior Parietal Lobule | 2.92 | 30 | -42 | 42 |
| 8 |  |  |  |  |  |
|  | R Cingulate Gyrus, posterior division | 2.75 | 4 | -26 | 42 |
| 8 |  |  |  |  |  |
|  | R Precuneus Cortex | 2.75 | 12 | -48 | 46 |
| 8 |  |  |  |  |  |
|  | R Supramarginal Gyrus, posterior division | 2.91 | 48 | -36 | 46 |
| 8 |  |  |  |  |  |
|  | R Supramarginal Gyrus, anterior division | 2.9 | 54 | -32 | 48 |
| 8 |  |  |  |  |  |
|  | R Superior Frontal Gyrus | 2.77 | 22 | 10 | 54 |
| 8 |  |  |  |  |  |
|  | R Lateral Occipital Cortex, superior division | 2.66 | 36 | -64 | 58 |
| 8 |  |  |  |  |  |
|  | R Precuneus Cortex | 2.88 | 4 | -50 | 70 |

# Supplementary Table S23. [US_onset _> UT_onset_]. Descriptive statistics for clusters and local maxima showing greater activation for Uncertain-Safety relative to Uncertain-Threat anticipation for the OSP Onset regressor (FDR *q*<0.05, whole-brain corrected). 

| ***mm***^***3***^ | **Label** | ***t*** | ***x*** | ***y*** | ***z*** |
| --- | --- | --- | --- | --- | --- |
| 27,264 |  |  |  |  |  |
|  | L Lingual Gyrus | 5.6 | -16 | -52 | -10 |
|  | L Temporal Occipital Fusiform Cortex | 5.58 | -26 | -56 | -8 |
|  | R Temporal Occipital Fusiform Cortex | 4.1 | 24 | -58 | -16 |
|  | R Lingual Gyrus | 7.76 | 18 | -62 | -8 |
|  | R Supracalcarine Cortex | 3.98 | 18 | -64 | 16 |
|  | R Cuneal Cortex | 6.13 | 10 | -72 | 24 |
|  | R Intracalcarine Cortex | 10.58 | 14 | -74 | 12 |
|  | L Intracalcarine Cortex | 10.46 | -12 | -78 | 10 |
| 20,304 |  |  |  |  |  |
|  | R Temporal Pole | 3.36 | 46 | 10 | -12 |
|  | R Insular Cortex | 5.99 | 42 | 8 | -2 |
|  | R Precentral Gyrus | 2.84 | 60 | 6 | 12 |
|  | R Superior Temporal Gyrus, anterior division | 5.55 | 62 | 4 | 0 |
|  | R Central Opercular Cortex | 5.89 | 60 | -2 | 6 |
|  | R Planum Polare | 4.93 | 48 | -2 | -8 |
|  | R Right Amygdala | 4.3 | 24 | -6 | -10 |
|  | R Right Putamen | 8.61 | 32 | -14 | 0 |
|  | R Heschls Gyrus (includes H1 and H2) | 5.93 | 38 | -22 | 14 |
|  | R Planum Temporale | 5.9 | 64 | -22 | 12 |
|  | R Superior Temporal Gyrus, posterior division | 4.96 | 66 | -26 | 10 |
|  | R Parietal Operculum Cortex | 5.71 | 44 | -32 | 20 |
| 20,240 |  |  |  |  |  |
|  | L Frontal Pole | 4.26 | -34 | 44 | -16 |
|  | L Left Accumbens | 3.01 | -10 | 6 | -12 |
|  | L Insular Cortex | 5.05 | -38 | 2 | -2 |
|  | L Precentral Gyrus | 4.52 | -54 | 0 | 14 |
|  | L Left Amygdala | 8.32 | -26 | -2 | -12 |
|  | L Planum Polare | 5.52 | -48 | -8 | 2 |
|  | L Left Pallidum | 3.93 | -20 | -8 | -6 |
|  | L Central Opercular Cortex | 5.77 | -40 | -12 | 18 |
|  | L Left Putamen | 7.88 | -30 | -16 | 2 |
|  | L Heschls Gyrus (includes H1 and H2) | 5.99 | -42 | -18 | 10 |
|  | L Planum Temporale | 5.56 | -60 | -28 | 14 |
|  | L Parietal Operculum Cortex | 5.84 | -34 | -30 | 18 |
| 11,504 |  |  |  |  |  |
|  | R Frontal Pole | 6.27 | 32 | 58 | 24 |
|  | R Superior Frontal Gyrus | 5.78 | 18 | 30 | 58 |
|  | R Middle Frontal Gyrus | 3.86 | 30 | 30 | 50 |
| 6,072 |  |  |  |  |  |
|  | R Cingulate Gyrus, anterior division | 4.21 | 8 | -4 | 42 |
|  | R Juxtapositional Lobule Cortex (formerly Supplementary Motor Cortex) | 4.14 | 8 | -10 | 48 |
|  | L Juxtapositional Lobule Cortex (formerly Supplementary Motor Cortex) | 4.07 | -8 | -12 | 48 |
|  | R Precentral Gyrus | 5.32 | 2 | -18 | 60 |
|  | L Precentral Gyrus | 4.66 | -6 | -22 | 60 |
|  | R Postcentral Gyrus | 4.44 | 6 | -38 | 62 |
| 4,192 |  |  |  |  |  |
|  | L Frontal Pole | 5.33 | -18 | 42 | 50 |
|  | L Superior Frontal Gyrus | 5.07 | -18 | 32 | 58 |
|  | L Middle Frontal Gyrus | 5.25 | -34 | 26 | 52 |
| 4,136 |  |  |  |  |  |
|  | L Postcentral Gyrus | 4.82 | -26 | -30 | 68 |
|  | L Precentral Gyrus | 7.08 | -20 | -30 | 60 |
|  | L Superior Parietal Lobule | 3.09 | -32 | -40 | 60 |
| 3,120 |  |  |  |  |  |
|  | R Precentral Gyrus | 6.75 | 24 | -26 | 58 |
|  | R Postcentral Gyrus | 4.78 | 24 | -34 | 62 |
| 2,528 |  |  |  |  |  |
|  | L Angular Gyrus | 5.31 | -46 | -58 | 34 |
|  | L Lateral Occipital Cortex, superior division | 6.15 | -56 | -68 | 32 |
| 1,360 |  |  |  |  |  |
|  | L Precentral Gyrus | 6.23 | -36 | -18 | 44 |
|  | L Postcentral Gyrus | 5.84 | -40 | -18 | 48 |
| 1,312 |  |  |  |  |  |
|  | R Angular Gyrus | 3.43 | 50 | -56 | 42 |
|  | R Lateral Occipital Cortex, superior division | 5.67 | 56 | -60 | 36 |
| 1,056 |  |  |  |  |  |
|  | R Right Caudate | 5.1 | 14 | 20 | 12 |
|  | R Right Thalamus | 4.78 | 14 | -12 | 16 |
| 920 |  |  |  |  |  |
|  | R Brain-Stem | 3.22 | 18 | -30 | -30 |
| 600 |  |  |  |  |  |
|  | L Left Caudate | 4.39 | -16 | -4 | 24 |
|  | L Left Thalamus | 4.65 | -14 | -20 | 2 |
| 416 |  |  |  |  |  |
|  | L Frontal Pole | 3.67 | -10 | 70 | 16 |
| 392 |  |  |  |  |  |
|  | R Frontal Medial Cortex | 5.75 | 4 | 38 | -30 |
| 392 |  |  |  |  |  |
|  | L Postcentral Gyrus | 3.7 | -56 | -16 | 42 |
| 384 |  |  |  |  |  |
|  | R Right Thalamus | 4.58 | 16 | -24 | 0 |
| 288 |  |  |  |  |  |
|  | L Postcentral Gyrus | 3.33 | -52 | -8 | 26 |
| 288 |  |  |  |  |  |
|  | R Precentral Gyrus | 3.71 | 40 | -16 | 40 |
| 256 |  |  |  |  |  |
|  | L Subcallosal Cortex | 4.03 | -2 | 26 | -2 |
|  | R Cingulate Gyrus, anterior division | 4.51 | 0 | 32 | 0 |
| 248 |  |  |  |  |  |
|  | R Precentral Gyrus | 3.43 | 56 | -2 | 26 |
|  | R Postcentral Gyrus | 3.03 | 62 | -6 | 26 |
| 208 |  |  |  |  |  |
|  | R Brain-Stem | 4.94 | 2 | -46 | -54 |
| 144 |  |  |  |  |  |
|  | L Frontal Pole | 3.64 | -30 | 52 | 4 |
| 136 |  |  |  |  |  |
|  | L Lateral Occipital Cortex, superior division | 3.45 | -46 | -68 | 22 |
| 128 |  |  |  |  |  |
|  | R Paracingulate Gyrus | 3.28 | 4 | 30 | 34 |
| 120 |  |  |  |  |  |
|  | L Precuneus Cortex | 3.79 | -6 | -60 | 32 |
| 120 |  |  |  |  |  |
|  | R Juxtapositional Lobule Cortex (formerly Supplementary Motor Cortex) | 3.76 | 0 | 4 | 46 |
| 112 |  |  |  |  |  |
|  | L Frontal Pole | 4.44 | -40 | 46 | -6 |
| 112 |  |  |  |  |  |
|  | R Postcentral Gyrus | 3.66 | 54 | -14 | 38 |
| 112 |  |  |  |  |  |
|  | L Postcentral Gyrus | 3.58 | -12 | -44 | 60 |
| 96 |  |  |  |  |  |
|  | L Frontal Pole | 3.34 | -36 | 46 | 8 |
| 80 |  |  |  |  |  |
|  | R Paracingulate Gyrus | 3.56 | 0 | 34 | 28 |
| 80 |  |  |  |  |  |
|  | L Middle Frontal Gyrus | 3.55 | -42 | 32 | 40 |
| 72 |  |  |  |  |  |
|  | R Postcentral Gyrus | 3.72 | 66 | -16 | 26 |
| 64 |  |  |  |  |  |
|  | L Frontal Medial Cortex | 3.24 | -4 | 42 | -28 |
| 56 |  |  |  |  |  |
|  | L Frontal Pole | 3.34 | -34 | 58 | -10 |
| 56 |  |  |  |  |  |
|  | R Postcentral Gyrus | 3.22 | 62 | -6 | 16 |
| 48 |  |  |  |  |  |
|  | L Middle Temporal Gyrus, posterior division | 3.44 | -60 | -12 | -22 |
| 48 |  |  |  |  |  |
|  | L Frontal Pole | 3.1 | -22 | 58 | -6 |
| 48 |  |  |  |  |  |
|  | R Parietal Operculum Cortex | 3.36 | 60 | -34 | 28 |
| 40 |  |  |  |  |  |
|  | R Frontal Pole | 3.11 | 32 | 54 | -14 |
| 40 |  |  |  |  |  |
|  | L Left Caudate | 3.57 | -14 | 20 | 10 |
| 32 |  |  |  |  |  |
|  | L Middle Temporal Gyrus, posterior division | 3.36 | -66 | -28 | -18 |
| 32 |  |  |  |  |  |
|  | R Right Hippocampus | 3.43 | 18 | -16 | -18 |
| 32 |  |  |  |  |  |
|  | L Middle Temporal Gyrus, posterior division | 3.66 | -56 | -22 | -16 |
| 32 |  |  |  |  |  |
|  | R Lateral Occipital Cortex, inferior division | 2.83 | 42 | -64 | 10 |
| 24 |  |  |  |  |  |
|  | L Brain-Stem | 2.97 | -10 | -40 | -46 |
| 24 |  |  |  |  |  |
|  | L Frontal Medial Cortex | 3.09 | -4 | 46 | -24 |
| 24 |  |  |  |  |  |
|  | R Temporal Pole | 3.13 | 38 | 14 | -20 |
| 24 |  |  |  |  |  |
|  | L Temporal Pole | 2.87 | -42 | 12 | -20 |
| 24 |  |  |  |  |  |
|  | R Frontal Pole | 3.41 | 18 | 60 | -12 |
| 24 |  |  |  |  |  |
|  | R Angular Gyrus | 2.9 | 44 | -56 | 28 |
| 24 |  |  |  |  |  |
|  | L Frontal Pole | 3.14 | -28 | 42 | 28 |
| 24 |  |  |  |  |  |
|  | L Precentral Gyrus | 2.78 | -54 | 2 | 28 |
| 24 |  |  |  |  |  |
|  | R Precentral Gyrus | 3.16 | 44 | -6 | 50 |
| 24 |  |  |  |  |  |
|  | L Superior Parietal Lobule | 2.85 | -14 | -52 | 68 |
| 16 |  |  |  |  |  |
|  | L Inferior Temporal Gyrus, anterior division | 3.37 | -52 | -10 | -38 |
| 16 |  |  |  |  |  |
|  | L Middle Temporal Gyrus, anterior division | 2.97 | -62 | -4 | -26 |
| 16 |  |  |  |  |  |
|  | L Temporal Occipital Fusiform Cortex | 3.22 | -32 | -54 | -20 |
| 16 |  |  |  |  |  |
|  | L Middle Temporal Gyrus, posterior division | 2.7 | -60 | -32 | -16 |
| 16 |  |  |  |  |  |
|  | L Middle Temporal Gyrus, posterior division | 2.91 | -68 | -30 | -14 |
| 16 |  |  |  |  |  |
|  | R Insular Cortex | 3.1 | 34 | 14 | -8 |
| 16 |  |  |  |  |  |
|  | R Frontal Pole | 2.76 | 22 | 56 | -4 |
| 16 |  |  |  |  |  |
|  | L Frontal Pole | 2.71 | -32 | 56 | -4 |
| 16 |  |  |  |  |  |
|  | R Cingulate Gyrus, anterior division | 3.21 | 8 | 40 | 0 |
| 16 |  |  |  |  |  |
|  | R Left Thalamus | 2.88 | 0 | -8 | 0 |
| 16 |  |  |  |  |  |
|  | R Frontal Pole | 2.75 | 40 | 46 | 0 |
| 16 |  |  |  |  |  |
|  | L Cingulate Gyrus, anterior division | 3.84 | -8 | 38 | 10 |
| 16 |  |  |  |  |  |
|  | L Left Thalamus | 3.63 | -12 | -4 | 14 |
| 16 |  |  |  |  |  |
|  | R Precentral Gyrus | 2.9 | 56 | 4 | 12 |
| 16 |  |  |  |  |  |
|  | R Right Thalamus | 2.82 | 20 | -20 | 14 |
| 16 |  |  |  |  |  |
|  | L Left Thalamus | 3.26 | -16 | -20 | 18 |
| 16 |  |  |  |  |  |
|  | L Cuneal Cortex | 2.68 | -18 | -72 | 20 |
| 16 |  |  |  |  |  |
|  | R Cingulate Gyrus, anterior division | 3.38 | 12 | 30 | 22 |
| 16 |  |  |  |  |  |
|  | R Paracingulate Gyrus | 2.9 | 2 | 12 | 42 |
| 16 |  |  |  |  |  |
|  | L Precentral Gyrus | 2.85 | -30 | -20 | 74 |
| 8 |  |  |  |  |  |
|  | L Brain-Stem | 2.87 | -6 | -48 | -56 |
| 8 |  |  |  |  |  |
|  | L Brain-Stem | 2.72 | -14 | -28 | -34 |
| 8 |  |  |  |  |  |
|  | L Temporal Pole | 2.85 | -20 | 4 | -32 |
| 8 |  |  |  |  |  |
|  | L Inferior Temporal Gyrus, posterior division | 2.71 | -56 | -20 | -28 |
| 8 |  |  |  |  |  |
|  | R Inferior Temporal Gyrus, posterior division | 2.67 | 64 | -22 | -26 |
| 8 |  |  |  |  |  |
|  | L Brain-Stem | 2.81 | -14 | -22 | -22 |
| 8 |  |  |  |  |  |
|  | L Left Hippocampus | 3.46 | -12 | -12 | -18 |
| 8 |  |  |  |  |  |
|  | R Frontal Pole | 3.06 | 26 | 46 | -16 |
| 8 |  |  |  |  |  |
|  | R Periaqueductal gray | 2.69 | 0 | -32 | -12 |
| 8 |  |  |  |  |  |
|  | L Middle Temporal Gyrus, anterior division | 2.88 | -62 | -4 | -10 |
| 8 |  |  |  |  |  |
|  | R Right Hippocampus | 5.06 | 24 | -26 | -8 |
| 8 |  |  |  |  |  |
|  | R Accumbens | 2.8 | 12 | 18 | -8 |
| 8 |  |  |  |  |  |
|  | R Cingulate Gyrus, anterior division | 2.75 | 4 | 38 | -6 |
| 8 |  |  |  |  |  |
|  | L Left Thalamus | 3.01 | -2 | -12 | -4 |
| 8 |  |  |  |  |  |
|  | L Left Pallidum | 2.71 | -12 | 2 | 0 |
| 8 |  |  |  |  |  |
|  | L Left Caudate | 2.81 | -10 | 18 | 2 |
| 8 |  |  |  |  |  |
|  | L Lateral Occipital Cortex, inferior division | 3.05 | -42 | -76 | 10 |
| 8 |  |  |  |  |  |
|  | R Right Thalamus | 2.97 | 16 | -22 | 10 |
| 8 |  |  |  |  |  |
|  | L Frontal Pole | 2.76 | -28 | 48 | 12 |
| 8 |  |  |  |  |  |
|  | R Frontal Pole | 3.2 | 18 | 62 | 12 |
| 8 |  |  |  |  |  |
|  | R Cingulate Gyrus, anterior division | 2.71 | 2 | 30 | 14 |
| 8 |  |  |  |  |  |
|  | R Frontal Pole | 2.71 | 14 | 66 | 14 |
| 8 |  |  |  |  |  |
|  | R Right Thalamus | 3 | 18 | -24 | 16 |
| 8 |  |  |  |  |  |
|  | R Occipital Pole | 2.86 | 6 | -88 | 18 |
| 8 |  |  |  |  |  |
|  | L Lateral Occipital Cortex, superior division | 2.71 | -22 | -86 | 18 |
| 8 |  |  |  |  |  |
|  | L Planum Temporale | 3 | -56 | -40 | 18 |
| 8 |  |  |  |  |  |
|  | L Central Opercular Cortex | 2.86 | -40 | -4 | 18 |
| 8 |  |  |  |  |  |
|  | L Cingulate Gyrus, anterior division | 2.76 | -8 | 32 | 18 |
| 8 |  |  |  |  |  |
|  | R Frontal Pole | 2.78 | 6 | 70 | 18 |
| 8 |  |  |  |  |  |
|  | L Precentral Gyrus | 2.76 | -52 | 2 | 24 |
| 8 |  |  |  |  |  |
|  | L Precentral Gyrus | 2.94 | -62 | -2 | 26 |
| 8 |  |  |  |  |  |
|  | L Frontal Pole | 2.79 | -8 | 64 | 30 |
| 8 |  |  |  |  |  |
|  | R Supramarginal Gyrus, anterior division | 2.81 | 56 | -20 | 32 |
| 8 |  |  |  |  |  |
|  | L Angular Gyrus | 2.73 | -50 | -52 | 34 |
| 8 |  |  |  |  |  |
|  | R Postcentral Gyrus | 2.68 | 42 | -16 | 34 |
| 8 |  |  |  |  |  |
|  | L Precentral Gyrus | 2.72 | -14 | -30 | 44 |
| 8 |  |  |  |  |  |
|  | R Precentral Gyrus | 2.73 | 50 | -12 | 48 |
| 8 |  |  |  |  |  |
|  | R Middle Frontal Gyrus | 2.7 | 34 | 24 | 50 |
| 8 |  |  |  |  |  |
|  | R Middle Frontal Gyrus | 2.85 | 38 | 22 | 52 |
| 8 |  |  |  |  |  |
|  | R Postcentral Gyrus | 2.69 | 32 | -30 | 56 |
| 8 |  |  |  |  |  |
|  | L Superior Parietal Lobule | 2.7 | -20 | -48 | 62 |
| 8 |  |  |  |  |  |
|  | L Postcentral Gyrus | 2.73 | -44 | -38 | 62 |
| 8 |  |  |  |  |  |
|  | L Precentral Gyrus | 2.68 | -2 | -26 | 70 |

# Supplementary Table S24. [CS_onset _> CT_onset_]. Descriptive statistics for clusters and local maxima showing greater activation for Certain-Safety relative to Certain-Threat anticipation for the OSP Onset regressor (FDR *q*<0.05, whole-brain corrected). 

| ***mm***^***3***^ | **Label** | ***t*** | ***x*** | ***y*** | ***z*** |
| --- | --- | --- | --- | --- | --- |
| 52,856 |  |  |  |  |  |
|  | L Cingulate Gyrus, anterior division | 3.71 | -4 | 12 | 40 |
|  | R Paracingulate Gyrus | 4.07 | 2 | 12 | 50 |
|  | L Juxtapositional Lobule Cortex (formerly Supplementary Motor Cortex) | 6.58 | -4 | 6 | 56 |
|  | L Middle Frontal Gyrus | 7.51 | -38 | -2 | 62 |
|  | L Superior Frontal Gyrus | 7.55 | -6 | -8 | 74 |
|  | R Juxtapositional Lobule Cortex (formerly Supplementary Motor Cortex) | 7.77 | 4 | -8 | 72 |
|  | R Superior Frontal Gyrus | 5.45 | 14 | -8 | 74 |
|  | L Precentral Gyrus | 9.30 | -18 | -24 | 76 |
|  | R Precentral Gyrus | 8.61 | 20 | -26 | 74 |
|  | R Postcentral Gyrus | 6.57 | 24 | -34 | 72 |
|  | L Postcentral Gyrus | 8.72 | -20 | -36 | 74 |
|  | L Superior Parietal Lobule | 8.12 | -22 | -46 | 70 |
|  | R Superior Parietal Lobule | 5.52 | 22 | -48 | 70 |
|  | R Lateral Occipital Cortex, superior division | 4.95 | 26 | -58 | 60 |
|  | L Lateral Occipital Cortex, superior division | 4.92 | -24 | -66 | 56 |
| 21,776 |  |  |  |  |  |
|  | R Parahippocampal Gyrus, posterior division | 3.42 | 20 | -28 | -18 |
|  | L Temporal Fusiform Cortex, posterior division | 4.94 | -28 | -42 | -14 |
|  | R Lingual Gyrus | 8.44 | 24 | -56 | -8 |
|  | L Lingual Gyrus | 8.20 | -28 | -58 | -6 |
|  | L Precuneus Cortex | 3.53 | -6 | -62 | 14 |
|  | R Occipital Fusiform Gyrus | 5.76 | 26 | -70 | -8 |
|  | R Intracalcarine Cortex | 12.11 | 14 | -72 | 12 |
|  | R Cuneal Cortex | 7.16 | 8 | -72 | 22 |
|  | L Occipital Fusiform Gyrus | 5.91 | -28 | -74 | -8 |
|  | L Intracalcarine Cortex | 12.44 | -12 | -76 | 8 |
|  | L Cuneal Cortex | 4.29 | -6 | -78 | 22 |
| 6,392 |  |  |  |  |  |
|  | L Superior Temporal Gyrus, posterior division | 2.91 | -62 | -32 | 2 |
|  | L Middle Temporal Gyrus, temporooccipital part | 5.17 | -56 | -48 | 6 |
|  | L Supramarginal Gyrus, posterior division | 4.15 | -50 | -48 | 12 |
|  | L Angular Gyrus | 6.36 | -60 | -52 | 12 |
|  | L Lateral Occipital Cortex, inferior division | 3.71 | -44 | -68 | 12 |
|  | L Lateral Occipital Cortex, superior division | 4.93 | -54 | -70 | 16 |
|  | L Occipital Pole | 4.41 | -38 | -92 | 12 |
| 1,344 |  |  |  |  |  |
|  | R Accumbens | 4.57 | 10 | 20 | -4 |
|  | R Putamen | 4.90 | 18 | 16 | -6 |
|  | R Amygdala | 4.19 | 24 | 0 | -12 |
| 1,200 |  |  |  |  |  |
|  | R Postcentral Gyrus | 4.60 | 58 | -16 | 40 |
|  | R Precentral Gyrus | 4.50 | 48 | -10 | 58 |
| 1,160 |  |  |  |  |  |
|  | L Putamen | 4.35 | -28 | -2 | -2 |
|  | L Pallidum | 3.84 | -20 | 2 | 0 |
| 856 |  |  |  |  |  |
|  | L Accumbens | 5.16 | -10 | 10 | -12 |
| 832 |  |  |  |  |  |
|  | R Angular Gyrus | 4.84 | 62 | -48 | 14 |
|  | R Middle Temporal Gyrus, temporooccipital part | 3.83 | 54 | -50 | 10 |
|  | R Supramarginal Gyrus, posterior division | 2.89 | 64 | -42 | 20 |
| 800 |  |  |  |  |  |
|  | R Postcentral Gyrus | 5.53 | 12 | -42 | 60 |
|  | R Precuneus Cortex | 5.26 | 8 | -44 | 64 |
|  | R Superior Parietal Lobule | 3.03 | 10 | -48 | 70 |
| 680 |  |  |  |  |  |
|  | L Lateral Occipital Cortex, superior division | 5.13 | -32 | -84 | 22 |
|  | L Occipital Pole | 3.23 | -24 | -92 | 26 |
| 672 |  |  |  |  |  |
|  | R Superior Frontal Gyrus | 4.48 | 18 | 28 | 60 |
| 416 |  |  |  |  |  |
|  | L Cingulate Gyrus, anterior division | 3.47 | -2 | 18 | 34 |
|  | R Paracingulate Gyrus | 3.99 | 4 | 20 | 38 |
|  | R Cingulate Gyrus, anterior division | 3.87 | 8 | 14 | 38 |
| 384 |  |  |  |  |  |
|  | L Middle Temporal Gyrus, anterior division | 4.37 | -56 | -2 | -16 |
|  | L Superior Temporal Gyrus, anterior division | 4.08 | -60 | 0 | -12 |
| 368 |  |  |  |  |  |
|  | R Putamen | 5.06 | 32 | -14 | -2 |
| 264 |  |  |  |  |  |
|  | R Angular Gyrus | 3.96 | 48 | -58 | 22 |
|  | R Lateral Occipital Cortex, superior division | 3.18 | 56 | -62 | 28 |
| 216 |  |  |  |  |  |
|  | R Postcentral Gyrus | 3.39 | 54 | -22 | 50 |
| 144 |  |  |  |  |  |
|  | L Insular Cortex | 3.74 | -42 | 6 | 0 |
|  | L Central Opercular Cortex | 3.62 | -48 | 6 | 2 |
| 128 |  |  |  |  |  |
|  | R Precentral Gyrus | 3.39 | 64 | 6 | 26 |
| 112 |  |  |  |  |  |
|  | R Putamen | 4.47 | 26 | -4 | 6 |
| 104 |  |  |  |  |  |
|  | R Putamen | 3.63 | 28 | -4 | -6 |
| 104 |  |  |  |  |  |
|  | L Parietal Operculum Cortex | 3.70 | -40 | -28 | 20 |
| 88 |  |  |  |  |  |
|  | R Middle Temporal Gyrus, temporooccipital part | 4.11 | 58 | -42 | 6 |
| 88 |  |  |  |  |  |
|  | R Supramarginal Gyrus, anterior division | 3.32 | 62 | -20 | 26 |
| 80 |  |  |  |  |  |
|  | L Superior Temporal Gyrus, posterior division | 3.59 | -56 | -22 | -4 |
| 80 |  |  |  |  |  |
|  | R Lateral Occipital Cortex, superior division | 3.05 | 58 | -62 | 20 |
| 80 |  |  |  |  |  |
|  | R Superior Frontal Gyrus | 3.35 | 10 | 8 | 70 |
| 72 |  |  |  |  |  |
|  | L Central Opercular Cortex | 3.30 | -58 | -20 | 18 |
| 72 |  |  |  |  |  |
|  | L Frontal Pole | 3.25 | -12 | 42 | 52 |
| 64 |  |  |  |  |  |
|  | L Temporal Occipital Fusiform Cortex | 3.65 | -42 | -58 | -14 |
| 64 |  |  |  |  |  |
|  | L Hippocampus | 3.94 | -22 | -28 | -8 |
| 64 |  |  |  |  |  |
|  | L Precentral Gyrus | 3.25 | -56 | 2 | 28 |
| 56 |  |  |  |  |  |
|  | L Precentral Gyrus | 3.60 | -10 | -22 | 46 |
| 48 |  |  |  |  |  |
|  | L Temporal Fusiform Cortex, posterior division | 3.13 | -40 | -24 | -18 |
| 48 |  |  |  |  |  |
|  | L Central Opercular Cortex | 3.24 | -48 | -22 | 20 |
| 48 |  |  |  |  |  |
|  | R Cingulate Gyrus, anterior division | 3.22 | 4 | 34 | 22 |
| 48 |  |  |  |  |  |
|  | L Postcentral Gyrus | 3.23 | -54 | -20 | 30 |
| 40 |  |  |  |  |  |
|  | L Temporal Occipital Fusiform Cortex | 3.09 | -30 | -46 | -18 |
| 40 |  |  |  |  |  |
|  | L Thalamus | 3.18 | -10 | -16 | 4 |
| 40 |  |  |  |  |  |
|  | R Frontal Pole | 3.43 | 18 | 46 | 46 |
| 40 |  |  |  |  |  |
|  | R Lateral Occipital Cortex, superior division | 3.51 | 14 | -62 | 62 |
| 32 |  |  |  |  |  |
|  | L Temporal Fusiform Cortex, posterior division | 3.30 | -34 | -24 | -26 |
| 32 |  |  |  |  |  |
|  | L Thalamus | 2.96 | -16 | -24 | 12 |
| 32 |  |  |  |  |  |
|  | L Central Opercular Cortex | 2.97 | -50 | -22 | 14 |
|  | L Parietal Operculum Cortex | 2.94 | -48 | -24 | 16 |
| 32 |  |  |  |  |  |
|  | R Cingulate Gyrus, anterior division | 3.20 | 4 | 30 | 18 |
| 24 |  |  |  |  |  |
|  | L Inferior Temporal Gyrus, anterior division | 3.45 | -44 | -6 | -46 |
| 24 |  |  |  |  |  |
|  | L Brain-Stem | 3.84 | -12 | -34 | -36 |
| 24 |  |  |  |  |  |
|  | L Lateral Occipital Cortex, inferior division | 3.11 | -46 | -66 | -12 |
| 24 |  |  |  |  |  |
|  | R Temporal Occipital Fusiform Cortex | 2.99 | 32 | -42 | -10 |
| 24 |  |  |  |  |  |
|  | R Hippocampus | 5.79 | 24 | -26 | -8 |
| 24 |  |  |  |  |  |
|  | R Insular Cortex | 2.96 | 44 | 6 | 0 |
| 24 |  |  |  |  |  |
|  | R Caudate | 3.25 | 18 | 12 | 18 |
| 24 |  |  |  |  |  |
|  | L Lateral Occipital Cortex, superior division | 2.83 | -18 | -84 | 20 |
| 24 |  |  |  |  |  |
|  | R Lateral Occipital Cortex, superior division | 3.12 | 24 | -84 | 20 |
| 24 |  |  |  |  |  |
|  | R Occipital Pole | 2.77 | 12 | -90 | 28 |
| 24 |  |  |  |  |  |
|  | R Middle Frontal Gyrus | 3.32 | 30 | 24 | 48 |
| 24 |  |  |  |  |  |
|  | R Precentral Gyrus | 3.18 | 44 | 0 | 52 |
| 24 |  |  |  |  |  |
|  | L Middle Frontal Gyrus | 3.05 | -30 | 22 | 56 |
| 16 |  |  |  |  |  |
|  | L Brain-Stem | 2.99 | -20 | -28 | -38 |
| 16 |  |  |  |  |  |
|  | L Temporal Pole | 3.39 | -50 | 12 | -20 |
| 16 |  |  |  |  |  |
|  | L Parahippocampal Gyrus, posterior division | 3.21 | -20 | -34 | -20 |
| 16 |  |  |  |  |  |
|  | L Middle Temporal Gyrus, anterior division | 2.82 | -60 | -8 | -10 |
| 16 |  |  |  |  |  |
|  | L Putamen | 3.04 | -26 | 10 | -4 |
| 16 |  |  |  |  |  |
|  | R Inferior Frontal Gyrus, pars triangularis | 3.13 | 52 | 28 | -4 |
| 16 |  |  |  |  |  |
|  | R Precuneus Cortex | 3.00 | 18 | -50 | 6 |
| 16 |  |  |  |  |  |
|  | R Caudate | 2.91 | 16 | 20 | 8 |
| 16 |  |  |  |  |  |
|  | R Thalamus | 3.63 | 14 | -16 | 20 |
| 16 |  |  |  |  |  |
|  | R Supramarginal Gyrus, posterior division | 2.80 | 60 | -44 | 24 |
| 16 |  |  |  |  |  |
|  | R Lateral Occipital Cortex, superior division | 3.09 | 56 | -70 | 24 |
| 16 |  |  |  |  |  |
|  | L Supramarginal Gyrus, anterior division | 3.00 | -62 | -40 | 28 |
| 16 |  |  |  |  |  |
|  | R Paracingulate Gyrus | 3.56 | 2 | 28 | 32 |
| 16 |  |  |  |  |  |
|  | L Middle Frontal Gyrus | 2.96 | -40 | 10 | 38 |
| 16 |  |  |  |  |  |
|  | L Lateral Occipital Cortex, superior division | 3.15 | -24 | -74 | 40 |
| 16 |  |  |  |  |  |
|  | R Precentral Gyrus | 3.07 | 42 | -6 | 48 |
| 16 |  |  |  |  |  |
|  | L Middle Frontal Gyrus | 2.88 | -28 | 28 | 50 |
| 8 |  |  |  |  |  |
|  | R Brain-Stem | 2.79 | 12 | -34 | -40 |
| 8 |  |  |  |  |  |
|  | R Inferior Temporal Gyrus, posterior division | 2.74 | 50 | -14 | -32 |
| 8 |  |  |  |  |  |
|  | L Temporal Pole | 3.08 | -24 | 8 | -32 |
| 8 |  |  |  |  |  |
|  | R Temporal Pole | 2.94 | 56 | 14 | -26 |
| 8 |  |  |  |  |  |
|  | L Temporal Fusiform Cortex, posterior division | 3.01 | -34 | -44 | -24 |
| 8 |  |  |  |  |  |
|  | R Temporal Fusiform Cortex, posterior division | 3.01 | 34 | -34 | -20 |
| 8 |  |  |  |  |  |
|  | L Temporal Fusiform Cortex, posterior division | 2.81 | -40 | -36 | -18 |
| 8 |  |  |  |  |  |
|  | L Temporal Pole | 3.05 | -50 | 16 | -18 |
| 8 |  |  |  |  |  |
|  | L Temporal Fusiform Cortex, posterior division | 3.11 | -32 | -38 | -16 |
| 8 |  |  |  |  |  |
|  | L Frontal Orbital Cortex | 2.76 | -34 | 20 | -16 |
| 8 |  |  |  |  |  |
|  | L Temporal Pole | 2.78 | -58 | 8 | -14 |
| 8 |  |  |  |  |  |
|  | L Hippocampus | 2.88 | -24 | -32 | -12 |
| 8 |  |  |  |  |  |
|  | L Frontal Orbital Cortex | 3.29 | -30 | 32 | -12 |
| 8 |  |  |  |  |  |
|  | L Pallidum | 2.90 | -22 | -4 | -4 |
| 8 |  |  |  |  |  |
|  | R Pallidum | 2.86 | 24 | -6 | -2 |
| 8 |  |  |  |  |  |
|  | L Caudate | 3.11 | -10 | 20 | -2 |
| 8 |  |  |  |  |  |
|  | L Putamen | 2.73 | -28 | 8 | 2 |
| 8 |  |  |  |  |  |
|  | L Insular Cortex | 2.82 | -38 | 2 | 4 |
| 8 |  |  |  |  |  |
|  | L Precentral Gyrus | 2.84 | -52 | 8 | 6 |
| 8 |  |  |  |  |  |
|  | R Inferior Frontal Gyrus, pars opercularis | 2.83 | 58 | 12 | 6 |
| 8 |  |  |  |  |  |
|  | L Lateral Occipital Cortex, inferior division | 2.83 | -50 | -68 | 8 |
| 8 |  |  |  |  |  |
|  | R Supramarginal Gyrus, posterior division | 2.89 | 66 | -40 | 8 |
| 8 |  |  |  |  |  |
|  | R Caudate | 2.79 | 18 | 10 | 12 |
| 8 |  |  |  |  |  |
|  | R Angular Gyrus | 2.86 | 52 | -52 | 16 |
| 8 |  |  |  |  |  |
|  | L Lateral Occipital Cortex, superior division | 2.74 | -22 | -82 | 18 |
| 8 |  |  |  |  |  |
|  | R Lateral Occipital Cortex, superior division | 2.74 | 52 | -64 | 18 |
| 8 |  |  |  |  |  |
|  | R Supramarginal Gyrus, posterior division | 2.76 | 46 | -44 | 18 |
| 8 |  |  |  |  |  |
|  | R Caudate | 3.08 | 18 | -20 | 22 |
| 8 |  |  |  |  |  |
|  | R Frontal Pole | 2.98 | 34 | 56 | 28 |
| 8 |  |  |  |  |  |
|  | L Cingulate Gyrus, anterior division | 2.85 | -8 | 16 | 34 |
| 8 |  |  |  |  |  |
|  | R Cingulate Gyrus, anterior division | 2.75 | 6 | 4 | 42 |
| 8 |  |  |  |  |  |
|  | R Precentral Gyrus | 2.82 | 60 | -6 | 44 |
| 8 |  |  |  |  |  |
|  | R Frontal Pole | 2.88 | 16 | 52 | 44 |
| 8 |  |  |  |  |  |
|  | L Lateral Occipital Cortex, superior division | 2.73 | -20 | -82 | 48 |
| 8 |  |  |  |  |  |
|  | L Precuneus Cortex | 3.40 | -10 | -48 | 48 |
| 8 |  |  |  |  |  |
|  | R Superior Frontal Gyrus | 2.73 | 20 | 30 | 48 |
| 8 |  |  |  |  |  |
|  | L Lateral Occipital Cortex, superior division | 2.74 | -24 | -70 | 50 |
| 8 |  |  |  |  |  |
|  | L Precentral Gyrus | 2.93 | -44 | -8 | 50 |
| 8 |  |  |  |  |  |
|  | R Superior Frontal Gyrus | 2.81 | 22 | 22 | 54 |
| 8 |  |  |  |  |  |
|  | R Superior Frontal Gyrus | 2.85 | 16 | 20 | 56 |
| 8 |  |  |  |  |  |
|  | R Precentral Gyrus | 3.08 | 38 | -14 | 64 |
| 8 |  |  |  |  |  |
|  | L Lateral Occipital Cortex, superior division | 3.10 | -14 | -70 | 66 |
| 8 |  |  |  |  |  |
|  | R Lateral Occipital Cortex, superior division | 3.00 | 18 | -64 | 66 |
| 8 |  |  |  |  |  |
|  | R Precentral Gyrus | 2.89 | 28 | -20 | 66 |
| 8 |  |  |  |  |  |
|  | R Superior Frontal Gyrus | 2.79 | 4 | 14 | 68 |

# Supplementary Table S25. [CT_CB3 _> CT_CB2_]. Descriptive statistics for clusters and local maxima showing greater activation for Convolved Block 3 relative to Convolved Block 2 for Certain-Threat anticipation (FDR *q*<0.05, whole-brain corrected). 

| ***mm***^***3***^ | **Label** | ***t*** | ***x*** | ***y*** | ***z*** |
| --- | --- | --- | --- | --- | --- |
| 153,424 |  |  |  |  |  |
|  | L Paracingulate Gyrus | 4.45 | -4 | 28 | 30 |
|  | R Frontal Orbital Cortex | 6.93 | 40 | 28 | 0 |
|  | R Frontal Operculum Cortex | 8.48 | 34 | 24 | 8 |
|  | L Frontal Orbital Cortex | 4.75 | -36 | 22 | -6 |
|  | L Frontal Operculum Cortex | 9.83 | -34 | 20 | 8 |
|  | R Insular Cortex | 5.92 | 38 | 20 | -8 |
|  | R Cingulate Gyrus, anterior division | 4.90 | 0 | 16 | 26 |
|  | L Insular Cortex | 8.72 | -36 | 14 | 4 |
|  | L Cingulate Gyrus, anterior division | 8.11 | -8 | 14 | 36 |
|  | R Inferior Frontal Gyrus, pars opercularis | 4.93 | 60 | 14 | 8 |
|  | L Inferior Frontal Gyrus, pars opercularis | 6.35 | -54 | 12 | 0 |
|  | R Paracingulate Gyrus | 8.54 | 0 | 12 | 46 |
|  | L Caudate | 7.63 | -6 | 10 | -2 |
|  | L Precentral Gyrus | 8.53 | -60 | 8 | 12 |
|  | R Caudate | 8.23 | 8 | 8 | 2 |
|  | L Superior Frontal Gyrus | 5.66 | -14 | 6 | 70 |
|  | R Putamen | 11.18 | 22 | 6 | 0 |
|  | R Juxtapositional Lobule Cortex (formerly Supplementary Motor Cortex) | 11.46 | 10 | 4 | 44 |
|  | R Central Opercular Cortex | 7.57 | 52 | 4 | 2 |
|  | L Putamen | 9.71 | -22 | 2 | 2 |
|  | L Central Opercular Cortex | 7.38 | -50 | 2 | 2 |
|  | R Thalamus | 8.53 | 12 | -4 | 14 |
|  | R Amygdala | 4.90 | 30 | -6 | -18 |
|  | L Juxtapositional Lobule Cortex (formerly Supplementary Motor Cortex) | 10.43 | -4 | -8 | 66 |
|  | L Pallidum | 5.91 | -26 | -8 | -4 |
|  | R Amygdala | 6.56 | 28 | -10 | -12 |
|  | L Amygdala | 6.25 | -26 | -10 | -12 |
|  | R Superior Frontal Gyrus | 9.96 | 14 | -10 | 70 |
|  | R Amygdala | 6.56 | 28 | -10 | -12 |
|  | R Planum Polare | 3.96 | 40 | -10 | -10 |
|  | L Postcentral Gyrus | 5.22 | -36 | -18 | 42 |
|  | R Heschls Gyrus (includes H1 and H2) | 7.36 | 52 | -18 | 8 |
|  | L Thalamus | 6.75 | -4 | -22 | 0 |
|  | R Postcentral Gyrus | 13.03 | 36 | -24 | 56 |
|  | R Precentral Gyrus | 12.97 | 30 | -24 | 52 |
|  | R Planum Temporale | 6.80 | 54 | -24 | 14 |
|  | R Cingulate Gyrus, posterior division | 5.52 | 12 | -24 | 42 |
|  | L Cingulate Gyrus, posterior division | 6.07 | -10 | -26 | 40 |
|  | R Brain-Stem | 5.63 | 10 | -26 | -18 |
|  | R Parietal Operculum Cortex | 8.64 | 56 | -28 | 22 |
|  | R Supramarginal Gyrus, anterior division | 5.39 | 58 | -32 | 34 |
|  | R Superior Temporal Gyrus, posterior division | 4.63 | 68 | -32 | 12 |
|  | L Brain-Stem | 6.79 | -6 | -34 | -8 |
|  | R Supramarginal Gyrus, posterior division | 7.56 | 66 | -38 | 34 |
|  | R Superior Parietal Lobule | 8.25 | 24 | -42 | 60 |
|  | R Angular Gyrus | 7.07 | 58 | -46 | 30 |
| 15,840 |  |  |  |  |  |
|  | L Central Opercular Cortex | 3.01 | -44 | -18 | 14 |
|  | L Insular Cortex | 2.81 | -38 | -20 | 2 |
|  | L Postcentral Gyrus | 6.18 | -62 | -22 | 26 |
|  | L Heschls Gyrus (includes H1 and H2) | 6.47 | -46 | -26 | 6 |
|  | L Supramarginal Gyrus, anterior division | 7.82 | -62 | -34 | 24 |
|  | L Parietal Operculum Cortex | 6.41 | -46 | -36 | 22 |
|  | L Planum Temporale | 6.12 | -48 | -38 | 18 |
|  | L Supramarginal Gyrus, posterior division | 5.84 | -64 | -42 | 36 |
|  | L Angular Gyrus | 4.21 | -46 | -56 | 46 |
|  | L Lateral Occipital Cortex, superior division | 4.16 | -50 | -64 | 48 |
| 12,984 |  |  |  |  |  |
|  | R Occipital Fusiform Gyrus | 3.45 | 30 | -80 | -14 |
|  | R Lingual Gyrus | 3.99 | 8 | -88 | -8 |
|  | R Lateral Occipital Cortex, inferior division | 4.19 | 40 | -90 | -2 |
|  | R Occipital Pole | 11.33 | 26 | -94 | 10 |
| 6,888 |  |  |  |  |  |
|  | L Frontal Pole | 8.02 | -36 | 48 | 30 |
|  | L Middle Frontal Gyrus | 6.27 | -38 | 36 | 40 |
| 5,208 |  |  |  |  |  |
|  | R Frontal Pole | 6.87 | 36 | 46 | 32 |
| 1,504 |  |  |  |  |  |
|  | L Occipital Fusiform Gyrus | 5.18 | -22 | -84 | -10 |
|  | L Lingual Gyrus | 6.05 | -8 | -86 | -8 |
|  | L Occipital Pole | 3.96 | -20 | -96 | -12 |
| 1,056 |  |  |  |  |  |
|  | L Precuneus Cortex | 6.24 | -10 | -70 | 40 |
| 808 |  |  |  |  |  |
|  | L Frontal Pole | 4.58 | -24 | 68 | -10 |
| 808 |  |  |  |  |  |
|  | R Precuneus Cortex | 5.12 | 14 | -70 | 40 |
| 640 |  |  |  |  |  |
|  | R Middle Temporal Gyrus, posterior division | 4.33 | 60 | -22 | -10 |
| 600 |  |  |  |  |  |
|  | R Temporal Occipital Fusiform Cortex | 4.71 | 34 | -60 | -20 |
| 584 |  |  |  |  |  |
|  | R Frontal Pole | 4.21 | 24 | 68 | -6 |
| 424 |  |  |  |  |  |
|  | L Lateral Occipital Cortex, inferior division | 3.95 | -50 | -82 | -4 |
| 288 |  |  |  |  |  |
|  | L Precentral Gyrus | 4.99 | -8 | -24 | 58 |
| 280 |  |  |  |  |  |
|  | R Brain-Stem | 4.01 | 8 | -24 | -34 |
| 280 |  |  |  |  |  |
|  | L Frontal Orbital Cortex | 4.19 | -28 | 36 | -12 |
| 232 |  |  |  |  |  |
|  | R Middle Temporal Gyrus, anterior division | 3.72 | 58 | -4 | -34 |
|  | R Inferior Temporal Gyrus, anterior division | 3.06 | 50 | -6 | -38 |
| 200 |  |  |  |  |  |
|  | R Temporal Pole | 3.73 | 48 | 12 | -38 |
| 176 |  |  |  |  |  |
|  | L Planum Polare | 4.77 | -44 | -10 | -10 |
| 120 |  |  |  |  |  |
|  | R Inferior Temporal Gyrus, temporooccipital part | 2.77 | 54 | -46 | -26 |
| 112 |  |  |  |  |  |
|  | L Middle Temporal Gyrus, posterior division | 3.49 | -60 | -26 | -12 |
| 112 |  |  |  |  |  |
|  | R Angular Gyrus | 3.21 | 44 | -52 | 58 |
| 104 |  |  |  |  |  |
|  | R Temporal Fusiform Cortex, posterior division | 4.02 | 34 | -16 | -32 |
| 96 |  |  |  |  |  |
|  | L Precuneus Cortex | 3.01 | -8 | -54 | 60 |
| 96 |  |  |  |  |  |
|  | R Precuneus Cortex | 3.13 | 2 | -44 | 58 |
| 80 |  |  |  |  |  |
|  | L Inferior Temporal Gyrus, temporooccipital part | 3.30 | -60 | -50 | -24 |
| 80 |  |  |  |  |  |
|  | L Supramarginal Gyrus, posterior division | 3.31 | -36 | -48 | 40 |
| 80 |  |  |  |  |  |
|  | R Precuneus Cortex | 3.03 | 2 | -44 | 42 |
| 72 |  |  |  |  |  |
|  | L Inferior Temporal Gyrus, anterior division | 3.36 | -46 | -10 | -38 |
| 72 |  |  |  |  |  |
|  | L Angular Gyrus | 3.84 | -36 | -56 | 38 |
| 72 |  |  |  |  |  |
|  | L Precentral Gyrus | 3.42 | -6 | -26 | 50 |
| 64 |  |  |  |  |  |
|  | L Postcentral Gyrus | 3.28 | -20 | -40 | 66 |
| 56 |  |  |  |  |  |
|  | L Inferior Temporal Gyrus, posterior division | 2.79 | -54 | -16 | -38 |
| 56 |  |  |  |  |  |
|  | R Frontal Orbital Cortex | 3.23 | 34 | 26 | -16 |
| 56 |  |  |  |  |  |
|  | R Paracingulate Gyrus | 2.44 | 6 | 44 | 34 |
|  | R Superior Frontal Gyrus | 3.32 | 6 | 40 | 38 |
| 48 |  |  |  |  |  |
|  | L Brain-Stem | 3.24 | -4 | -40 | -56 |
| 48 |  |  |  |  |  |
|  | L Inferior Temporal Gyrus, posterior division | 3.23 | -50 | -40 | -22 |
| 48 |  |  |  |  |  |
|  | L Insular Cortex | 2.69 | -40 | -16 | -4 |
|  | L Planum Polare | 2.65 | -46 | -16 | -4 |
| 48 |  |  |  |  |  |
|  | L Middle Temporal Gyrus, temporooccipital part | 2.87 | -58 | -60 | 8 |
| 48 |  |  |  |  |  |
|  | L Precuneus Cortex | 2.96 | -2 | -64 | 56 |
| 48 |  |  |  |  |  |
|  | L Postcentral Gyrus | 3.36 | -20 | -46 | 70 |
| 40 |  |  |  |  |  |
|  | L Middle Temporal Gyrus, posterior division | 2.80 | -70 | -28 | -12 |
| 40 |  |  |  |  |  |
|  | L Occipital Pole | 3.87 | -12 | -94 | 0 |
| 40 |  |  |  |  |  |
|  | R Cingulate Gyrus, anterior division | 2.85 | 2 | 34 | 4 |
| 40 |  |  |  |  |  |
|  | R Middle Temporal Gyrus, temporooccipital part | 2.80 | 52 | -48 | 6 |
| 40 |  |  |  |  |  |
|  | R Precuneus Cortex | 3.02 | 2 | -50 | 50 |
| 32 |  |  |  |  |  |
|  | L Brain-Stem | 3.03 | -10 | -38 | -38 |
| 32 |  |  |  |  |  |
|  | L Temporal Pole | 2.76 | -52 | 4 | -34 |
| 32 |  |  |  |  |  |
|  | R Inferior Temporal Gyrus, temporooccipital part | 2.71 | 58 | -50 | -20 |
| 32 |  |  |  |  |  |
|  | L Middle Temporal Gyrus, posterior division | 3.09 | -58 | -34 | -12 |
| 32 |  |  |  |  |  |
|  | L Thalamus | 2.99 | -18 | -28 | 8 |
| 32 |  |  |  |  |  |
|  | R Cingulate Gyrus, anterior division | 3.04 | 2 | -4 | 34 |
| 32 |  |  |  |  |  |
|  | L Middle Frontal Gyrus | 3.06 | -38 | 22 | 42 |
| 32 |  |  |  |  |  |
|  | R Superior Parietal Lobule | 2.88 | 38 | -54 | 64 |
| 32 |  |  |  |  |  |
|  | R Precuneus Cortex | 2.80 | 6 | -48 | 62 |
| 24 |  |  |  |  |  |
|  | R Parahippocampal Gyrus, anterior division | 3.28 | 22 | -10 | -32 |
| 24 |  |  |  |  |  |
|  | R Inferior Temporal Gyrus, posterior division | 2.74 | 54 | -12 | -30 |
| 24 |  |  |  |  |  |
|  | R Brain-Stem | 2.75 | 14 | -20 | -28 |
| 24 |  |  |  |  |  |
|  | L Inferior Temporal Gyrus, posterior division | 2.96 | -56 | -42 | -24 |
| 24 |  |  |  |  |  |
|  | R Temporal Fusiform Cortex, posterior division | 3.46 | 42 | -32 | -24 |
| 24 |  |  |  |  |  |
|  | L Frontal Orbital Cortex | 3.42 | -22 | 28 | -22 |
| 24 |  |  |  |  |  |
|  | L Frontal Pole | 2.99 | -20 | 54 | -14 |
| 24 |  |  |  |  |  |
|  | R Middle Temporal Gyrus, posterior division | 2.83 | 66 | -32 | -14 |
| 24 |  |  |  |  |  |
|  | R Hippocampus | 2.60 | 36 | -20 | -12 |
| 24 |  |  |  |  |  |
|  | L Lateral Occipital Cortex, inferior division | 3.23 | -40 | -74 | 2 |
| 24 |  |  |  |  |  |
|  | L Middle Temporal Gyrus, temporooccipital part | 2.54 | -56 | -54 | 8 |
| 24 |  |  |  |  |  |
|  | L Putamen | 3.15 | -30 | -14 | 8 |
| 24 |  |  |  |  |  |
|  | L Middle Frontal Gyrus | 2.73 | -42 | 20 | 36 |
| 24 |  |  |  |  |  |
|  | R Precuneus Cortex | 2.68 | 10 | -70 | 52 |
| 24 |  |  |  |  |  |
|  | R Precuneus Cortex | 2.66 | 10 | -58 | 58 |
| 16 |  |  |  |  |  |
|  | R Brain-Stem | 2.46 | 6 | -44 | -46 |
| 16 |  |  |  |  |  |
|  | L Temporal Pole | 2.93 | -38 | 8 | -44 |
| 16 |  |  |  |  |  |
|  | R Brain-Stem | 2.86 | 6 | -28 | -42 |
| 16 |  |  |  |  |  |
|  | R Inferior Temporal Gyrus, anterior division | 2.67 | 48 | 0 | -34 |
| 16 |  |  |  |  |  |
|  | L Hippocampus | 2.74 | -28 | -8 | -24 |
| 16 |  |  |  |  |  |
|  | R Inferior Temporal Gyrus, posterior division | 2.66 | 50 | -32 | -22 |
| 16 |  |  |  |  |  |
|  | L Inferior Temporal Gyrus, posterior division | 2.55 | -56 | -34 | -18 |
| 16 |  |  |  |  |  |
|  | R Middle Temporal Gyrus, posterior division | 2.71 | 70 | -22 | -10 |
| 16 |  |  |  |  |  |
|  | L Occipital Pole | 2.59 | -12 | -102 | -4 |
| 16 |  |  |  |  |  |
|  | L Occipital Pole | 3.04 | -20 | -106 | -2 |
| 16 |  |  |  |  |  |
|  | R Lateral Occipital Cortex, inferior division | 2.69 | 58 | -66 | 4 |
| 16 |  |  |  |  |  |
|  | L Middle Temporal Gyrus, temporooccipital part | 2.59 | -54 | -60 | 4 |
| 16 |  |  |  |  |  |
|  | L Frontal Pole | 2.73 | -44 | 36 | 4 |
| 16 |  |  |  |  |  |
|  | R Frontal Pole | 2.48 | 34 | 56 | 8 |
| 16 |  |  |  |  |  |
|  | L Frontal Pole | 2.51 | -36 | 60 | 12 |
| 16 |  |  |  |  |  |
|  | R Frontal Pole | 2.60 | 24 | 50 | 14 |
| 16 |  |  |  |  |  |
|  | R Frontal Pole | 2.64 | 38 | 42 | 18 |
| 16 |  |  |  |  |  |
|  | R Middle Frontal Gyrus | 2.89 | 42 | 32 | 28 |
| 16 |  |  |  |  |  |
|  | L Precuneus Cortex | 2.61 | -10 | -46 | 46 |
| 8 |  |  |  |  |  |
|  | L Temporal Fusiform Cortex, anterior division | 2.65 | -36 | 0 | -46 |
| 8 |  |  |  |  |  |
|  | R Inferior Temporal Gyrus, anterior division | 2.81 | 46 | 4 | -46 |
| 8 |  |  |  |  |  |
|  | R Temporal Fusiform Cortex, posterior division | 2.43 | 30 | -10 | -44 |
| 8 |  |  |  |  |  |
|  | R Brain-Stem | 2.58 | 12 | -24 | -42 |
| 8 |  |  |  |  |  |
|  | L Temporal Pole | 2.44 | -46 | 4 | -42 |
| 8 |  |  |  |  |  |
|  | L Brain-Stem | 2.82 | -4 | -20 | -36 |
| 8 |  |  |  |  |  |
|  | R Parahippocampal Gyrus, anterior division | 2.45 | 22 | -8 | -36 |
| 8 |  |  |  |  |  |
|  | L Inferior Temporal Gyrus, anterior division | 2.67 | -56 | -6 | -36 |
| 8 |  |  |  |  |  |
|  | R Temporal Pole | 2.50 | 54 | 10 | -36 |
| 8 |  |  |  |  |  |
|  | L Brain-Stem | 2.90 | -18 | -24 | -34 |
| 8 |  |  |  |  |  |
|  | R Inferior Temporal Gyrus, posterior division | 2.50 | 58 | -34 | -28 |
| 8 |  |  |  |  |  |
|  | L Inferior Temporal Gyrus, posterior division | 2.60 | -54 | -32 | -28 |
| 8 |  |  |  |  |  |
|  | L Inferior Temporal Gyrus, posterior division | 2.77 | -58 | -28 | -20 |
| 8 |  |  |  |  |  |
|  | L Middle Temporal Gyrus, posterior division | 2.49 | -60 | -20 | -20 |
| 8 |  |  |  |  |  |
|  | L Frontal Orbital Cortex | 2.69 | -14 | 32 | -20 |
| 8 |  |  |  |  |  |
|  | L Frontal Orbital Cortex | 2.69 | -20 | 20 | -18 |
| 8 |  |  |  |  |  |
|  | R Frontal Pole | 2.89 | 26 | 58 | -18 |
| 8 |  |  |  |  |  |
|  | L Frontal Pole | 2.85 | -38 | 56 | -16 |
| 8 |  |  |  |  |  |
|  | R Middle Temporal Gyrus, posterior division | 2.61 | 62 | -34 | -14 |
| 8 |  |  |  |  |  |
|  | L Amygdala | 2.43 | -18 | -10 | -14 |
| 8 |  |  |  |  |  |
|  | L Hippocampus | 2.67 | -34 | -20 | -12 |
| 8 |  |  |  |  |  |
|  | R Lateral Occipital Cortex, inferior division | 2.50 | 40 | -78 | -10 |
| 8 |  |  |  |  |  |
|  | L Occipital Pole | 2.45 | -28 | -92 | -2 |
| 8 |  |  |  |  |  |
|  | L Thalamus | 2.60 | -12 | -26 | 0 |
| 8 |  |  |  |  |  |
|  | R Pallidum | 2.58 | 22 | -14 | 0 |
| 8 |  |  |  |  |  |
|  | L Hippocampus | 2.49 | -24 | -40 | 4 |
| 8 |  |  |  |  |  |
|  | R Thalamus | 2.60 | 22 | -28 | 8 |
| 8 |  |  |  |  |  |
|  | L Caudate | 2.55 | -18 | 14 | 10 |
| 8 |  |  |  |  |  |
|  | R Central Opercular Cortex | 2.51 | 44 | -4 | 14 |
| 8 |  |  |  |  |  |
|  | L Frontal Pole | 2.73 | -34 | 58 | 22 |
| 8 |  |  |  |  |  |
|  | L Paracingulate Gyrus | 2.47 | -4 | 36 | 28 |
| 8 |  |  |  |  |  |
|  | R Postcentral Gyrus | 2.62 | 54 | -14 | 32 |
| 8 |  |  |  |  |  |
|  | L Paracingulate Gyrus | 2.43 | -2 | 34 | 32 |
| 8 |  |  |  |  |  |
|  | L Middle Frontal Gyrus | 2.56 | -40 | 24 | 34 |
| 8 |  |  |  |  |  |
|  | R Middle Frontal Gyrus | 2.68 | 38 | 26 | 34 |
| 8 |  |  |  |  |  |
|  | R Superior Frontal Gyrus | 2.74 | 6 | 36 | 50 |
| 8 |  |  |  |  |  |
|  | R Superior Frontal Gyrus | 2.46 | 18 | 16 | 66 |

# Supplementary Table S26. [UT_CB3 _> UT_CB2_]. Descriptive statistics for clusters and local maxima showing greater activation for Convolved Block 3 relative to Convolved Block 2 of Uncertain-Threat anticipation (FDR *q*<0.05, whole-brain corrected). 

| ***mm***^***3***^ | **Label** | ***t*** | ***x*** | ***y*** | ***z*** |
| --- | --- | --- | --- | --- | --- |
| 63,520 |  |  |  |  |  |
|  | L Paracingulate Gyrus | 3.17 | -4 | 48 | 14 |
|  | R Paracingulate Gyrus | 4.97 | 0 | 38 | 28 |
|  | L Cingulate Gyrus, anterior division | 5.71 | -6 | 10 | 40 |
|  | R Cingulate Gyrus, anterior division | 8.59 | 2 | 8 | 44 |
|  | L Juxtapositional Lobule Cortex (formerly Supplementary Motor Cortex) | 5.12 | -8 | 2 | 46 |
|  | L Superior Frontal Gyrus | 5.75 | -20 | -2 | 70 |
|  | R Middle Frontal Gyrus | 4.40 | 36 | -4 | 56 |
|  | R Superior Frontal Gyrus | 7.95 | 22 | -8 | 70 |
|  | R Juxtapositional Lobule Cortex (formerly Supplementary Motor Cortex) | 8.41 | 4 | -12 | 48 |
|  | L Cingulate Gyrus, posterior division | 7.57 | -12 | -22 | 40 |
|  | R Cingulate Gyrus, posterior division | 9.91 | 12 | -24 | 40 |
|  | R Precentral Gyrus | 7.75 | 4 | -24 | 50 |
|  | R Postcentral Gyrus | 5.77 | 28 | -36 | 68 |
|  | R Superior Parietal Lobule | 6.47 | 26 | -42 | 66 |
|  | L Postcentral Gyrus | 2.85 | -10 | -46 | 66 |
|  | L Precuneus Cortex | 5.31 | -4 | -48 | 62 |
|  | R Precuneus Cortex | 6.04 | 8 | -48 | 58 |
|  | L Superior Parietal Lobule | 4.12 | -16 | -54 | 60 |
| 42,320 |  |  |  |  |  |
|  | R Frontal Pole | 3.98 | 56 | 36 | -4 |
|  | R Frontal Orbital Cortex | 4.28 | 46 | 30 | -4 |
|  | R Inferior Frontal Gyrus, pars triangularis | 4.97 | 48 | 24 | 6 |
|  | R Frontal Operculum Cortex | 6.50 | 42 | 18 | 4 |
|  | R Inferior Frontal Gyrus, pars opercularis | 4.60 | 60 | 14 | 14 |
|  | R Temporal Pole | 7.70 | 50 | 10 | -4 |
|  | R Precentral Gyrus | 6.11 | 64 | 8 | 10 |
|  | R Planum Polare | 8.70 | 58 | 2 | 2 |
|  | R Central Opercular Cortex | 7.46 | 58 | 0 | 8 |
|  | R Pallidum | 2.97 | 22 | 0 | -2 |
|  | R Putamen | 5.87 | 32 | -6 | -4 |
|  | R Insular Cortex | 6.26 | 42 | -8 | -6 |
|  | R Middle Temporal Gyrus, posterior division | 3.81 | 58 | -22 | -10 |
|  | R Planum Temporale | 6.38 | 56 | -24 | 14 |
|  | R Heschls Gyrus (includes H1 and H2) | 5.34 | 46 | -24 | 14 |
|  | R Superior Temporal Gyrus, posterior division | 6.17 | 70 | -26 | 6 |
|  | R Parietal Operculum Cortex | 10.23 | 52 | -30 | 24 |
|  | R Supramarginal Gyrus, anterior division | 7.18 | 56 | -32 | 34 |
|  | R Supramarginal Gyrus, posterior division | 6.69 | 58 | -44 | 32 |
|  | R Angular Gyrus | 3.77 | 46 | -50 | 40 |
| 26,904 |  |  |  |  |  |
|  | L Frontal Orbital Cortex | 4.34 | -38 | 24 | -6 |
|  | L Frontal Operculum Cortex | 5.70 | -46 | 16 | -4 |
|  | L Inferior Frontal Gyrus, pars opercularis | 5.74 | -56 | 12 | 0 |
|  | L Precentral Gyrus | 6.23 | -60 | 8 | 6 |
|  | L Central Opercular Cortex | 7.21 | -52 | 2 | 2 |
|  | L Heschls Gyrus (includes H1 and H2) | 5.06 | -50 | -16 | 8 |
|  | L Planum Polare | 5.01 | -40 | -20 | 0 |
|  | L Postcentral Gyrus | 5.48 | -64 | -22 | 18 |
|  | L Insular Cortex | 4.56 | -38 | -22 | 2 |
|  | L Planum Temporale | 6.20 | -58 | -24 | 12 |
|  | L Parietal Operculum Cortex | 6.78 | -62 | -32 | 20 |
|  | L Supramarginal Gyrus, anterior division | 6.91 | -66 | -34 | 34 |
|  | L Supramarginal Gyrus, posterior division | 5.89 | -58 | -42 | 24 |
|  | L Angular Gyrus | 3.07 | -58 | -54 | 40 |
| 16,584 |  |  |  |  |  |
|  | R Middle Temporal Gyrus, temporooccipital part | 5.14 | 50 | -56 | 4 |
|  | R Inferior Temporal Gyrus, temporooccipital part | 5.26 | 46 | -58 | -14 |
|  | R Occipital Fusiform Gyrus | 5.23 | 38 | -68 | -14 |
|  | R Lateral Occipital Cortex, inferior division | 9.79 | 46 | -76 | -10 |
|  | R Lateral Occipital Cortex, superior division | 3.51 | 30 | -86 | 12 |
| 6,576 |  |  |  |  |  |
|  | L Lateral Occipital Cortex, inferior division | 6.37 | -36 | -90 | -8 |
|  | L Occipital Pole | 6.53 | -38 | -92 | -10 |
| 1,640 |  |  |  |  |  |
|  | L Precentral Gyrus | 5.22 | -48 | -6 | 54 |
| 1,512 |  |  |  |  |  |
|  | R Caudate | 4.45 | 14 | 4 | 22 |
|  | R Thalamus | 5.29 | 10 | -8 | 6 |
| 736 |  |  |  |  |  |
|  | R Frontal Pole | 4.83 | 10 | 62 | 28 |
|  | R Superior Frontal Gyrus | 3.43 | 6 | 56 | 20 |
| 632 |  |  |  |  |  |
|  | R Frontal Pole | 3.71 | 34 | 40 | 40 |
|  | R Middle Frontal Gyrus | 2.89 | 32 | 34 | 36 |
| 552 |  |  |  |  |  |
|  | R Superior Frontal Gyrus | 3.82 | 2 | 24 | 54 |
| 512 |  |  |  |  |  |
|  | L Frontal Pole | 4.77 | -36 | 40 | 38 |
| 456 |  |  |  |  |  |
|  | R Brain-Stem | 4.31 | 12 | -26 | -34 |
| 320 |  |  |  |  |  |
|  | L Thalamus | 4.79 | -12 | -24 | 18 |
| 248 |  |  |  |  |  |
|  | L Putamen | 3.49 | -24 | 0 | -2 |
| 208 |  |  |  |  |  |
|  | L Frontal Pole | 3.40 | -38 | 44 | 28 |
| 200 |  |  |  |  |  |
|  | L Putamen | 3.24 | -30 | -14 | -2 |
| 144 |  |  |  |  |  |
|  | R Caudate | 3.92 | 12 | 12 | 18 |
| 120 |  |  |  |  |  |
|  | R Inferior Temporal Gyrus, posterior division | 3.45 | 52 | -36 | -18 |
|  | R Inferior Temporal Gyrus, temporooccipital part | 3.37 | 54 | -42 | -14 |
| 104 |  |  |  |  |  |
|  | R Brain-Stem | 3.01 | 2 | -16 | -36 |
|  | L Brain-Stem | 2.91 | -2 | -18 | -38 |
| 104 |  |  |  |  |  |
|  | L Insular Cortex | 3.48 | -36 | -6 | -2 |
|  | L Putamen | 2.92 | -32 | -8 | -4 |
| 96 |  |  |  |  |  |
|  | R Temporal Occipital Fusiform Cortex | 3.33 | 46 | -46 | -22 |
| 88 |  |  |  |  |  |
|  | R Frontal Orbital Cortex | 3.26 | 24 | 18 | -24 |
| 80 |  |  |  |  |  |
|  | R Brain-Stem | 4.24 | 12 | -16 | -32 |
| 80 |  |  |  |  |  |
|  | L Caudate | 3.56 | -12 | 22 | -4 |
| 80 |  |  |  |  |  |
|  | L Thalamus | 3.27 | -12 | -8 | 8 |
| 72 |  |  |  |  |  |
|  | L Frontal Orbital Cortex | 3.20 | -28 | 20 | -12 |
| 72 |  |  |  |  |  |
|  | L Middle Temporal Gyrus, temporooccipital part | 3.32 | -54 | -58 | 10 |
| 72 |  |  |  |  |  |
|  | R Frontal Pole | 2.87 | 26 | 54 | 28 |
| 72 |  |  |  |  |  |
|  | R Middle Frontal Gyrus | 3.12 | 34 | 28 | 40 |
| 64 |  |  |  |  |  |
|  | L Thalamus | 3.05 | -6 | -24 | 10 |
| 48 |  |  |  |  |  |
|  | R Temporal Pole | 3.42 | 40 | 24 | -36 |
| 48 |  |  |  |  |  |
|  | R Middle Temporal Gyrus, posterior division | 3.12 | 62 | -12 | -30 |
|  | R Inferior Temporal Gyrus, posterior division | 2.94 | 60 | -16 | -30 |
| 48 |  |  |  |  |  |
|  | R Subcallosal Cortex | 3.39 | 4 | 30 | -8 |
| 40 |  |  |  |  |  |
|  | L Inferior Temporal Gyrus, posterior division | 3.44 | -50 | -20 | -30 |
| 40 |  |  |  |  |  |
|  | R Inferior Temporal Gyrus, temporooccipital part | 2.81 | 58 | -52 | -20 |
| 40 |  |  |  |  |  |
|  | L Parahippocampal Gyrus, posterior division | 3.01 | -16 | -30 | -14 |
| 40 |  |  |  |  |  |
|  | L Caudate | 3.23 | -16 | 16 | 14 |
| 40 |  |  |  |  |  |
|  | R Superior Frontal Gyrus | 2.98 | 6 | 38 | 50 |
| 32 |  |  |  |  |  |
|  | L Brain-Stem | 2.90 | -10 | -28 | -32 |
| 32 |  |  |  |  |  |
|  | R Inferior Temporal Gyrus, posterior division | 3.01 | 62 | -24 | -28 |
| 32 |  |  |  |  |  |
|  | L Hippocampus | 3.22 | -28 | -20 | -18 |
| 32 |  |  |  |  |  |
|  | R Inferior Temporal Gyrus, temporooccipital part | 3.66 | 62 | -50 | -18 |
| 32 |  |  |  |  |  |
|  | L Caudate | 2.79 | -14 | 4 | 22 |
| 32 |  |  |  |  |  |
|  | L Frontal Pole | 2.99 | -4 | 62 | 26 |
| 32 |  |  |  |  |  |
|  | R Frontal Pole | 2.94 | 38 | 52 | 28 |
| 32 |  |  |  |  |  |
|  | R Middle Frontal Gyrus | 3.33 | 40 | 16 | 44 |
| 24 |  |  |  |  |  |
|  | L Brain-Stem | 2.63 | -8 | -44 | -38 |
| 24 |  |  |  |  |  |
|  | R Inferior Temporal Gyrus, posterior division | 2.93 | 44 | -22 | -30 |
| 24 |  |  |  |  |  |
|  | R Inferior Temporal Gyrus, posterior division | 3.01 | 46 | -36 | -18 |
| 24 |  |  |  |  |  |
|  | R Middle Temporal Gyrus, temporooccipital part | 2.70 | 62 | -46 | 2 |
| 24 |  |  |  |  |  |
|  | R Cingulate Gyrus, anterior division | 3.64 | 12 | 42 | 4 |
| 24 |  |  |  |  |  |
|  | L Caudate | 2.85 | -8 | 16 | 8 |
| 24 |  |  |  |  |  |
|  | L Caudate | 3.88 | -14 | -12 | 22 |
| 24 |  |  |  |  |  |
|  | R Frontal Pole | 2.91 | 16 | 56 | 22 |
| 24 |  |  |  |  |  |
|  | R Cingulate Gyrus, posterior division | 2.83 | 0 | -18 | 28 |
| 24 |  |  |  |  |  |
|  | R Frontal Pole | 2.83 | 28 | 52 | 38 |
| 24 |  |  |  |  |  |
|  | L Superior Frontal Gyrus | 2.69 | -18 | 12 | 58 |
| 24 |  |  |  |  |  |
|  | L Superior Frontal Gyrus | 2.75 | -6 | 14 | 66 |
| 16 |  |  |  |  |  |
|  | R Brain-Stem | 2.86 | 14 | -28 | -40 |
| 16 |  |  |  |  |  |
|  | R Brain-Stem | 2.66 | 6 | -24 | -38 |
| 16 |  |  |  |  |  |
|  | L Temporal Pole | 2.77 | -28 | 22 | -36 |
| 16 |  |  |  |  |  |
|  | R Brain-Stem | 2.58 | 6 | -26 | -24 |
| 16 |  |  |  |  |  |
|  | L Subcallosal Cortex | 2.89 | -8 | 16 | -22 |
| 16 |  |  |  |  |  |
|  | R Inferior Temporal Gyrus, posterior division | 2.59 | 44 | -32 | -22 |
| 16 |  |  |  |  |  |
|  | R Frontal Orbital Cortex | 2.82 | 34 | 30 | -20 |
| 16 |  |  |  |  |  |
|  | R Amygdala | 2.80 | 22 | 2 | -20 |
| 16 |  |  |  |  |  |
|  | R Hippocampus | 2.81 | 38 | -22 | -14 |
| 16 |  |  |  |  |  |
|  | R Frontal Pole | 2.61 | 26 | 58 | -10 |
| 16 |  |  |  |  |  |
|  | R Frontal Pole | 2.67 | 32 | 52 | -10 |
| 16 |  |  |  |  |  |
|  | L Subcallosal Cortex | 2.97 | -2 | 26 | -6 |
| 16 |  |  |  |  |  |
|  | L Hippocampus | 3.04 | -32 | -36 | -4 |
| 16 |  |  |  |  |  |
|  | R Pallidum | 2.69 | 16 | 6 | 2 |
| 16 |  |  |  |  |  |
|  | L Caudate | 3.21 | -18 | 22 | 4 |
| 16 |  |  |  |  |  |
|  | L Caudate | 2.65 | -8 | 0 | 10 |
| 16 |  |  |  |  |  |
|  | R Caudate | 2.67 | 16 | 18 | 14 |
| 16 |  |  |  |  |  |
|  | L Caudate | 3.13 | -16 | -18 | 22 |
| 16 |  |  |  |  |  |
|  | L Paracingulate Gyrus | 2.68 | -10 | 44 | 24 |
| 16 |  |  |  |  |  |
|  | R Precentral Gyrus | 2.94 | 60 | 4 | 26 |
| 16 |  |  |  |  |  |
|  | R Cingulate Gyrus, anterior division | 2.70 | 2 | -10 | 30 |
| 8 |  |  |  |  |  |
|  | R Brain-Stem | 2.53 | 4 | -26 | -34 |
| 8 |  |  |  |  |  |
|  | L Parahippocampal Gyrus, anterior division | 3.13 | -22 | -14 | -34 |
| 8 |  |  |  |  |  |
|  | L Brain-Stem | 3.08 | -6 | -34 | -28 |
| 8 |  |  |  |  |  |
|  | L Hippocampus/Amygdala | 2.55 | -28 | -6 | -24 |
| 8 |  |  |  |  |  |
|  | R Temporal Pole | 2.57 | 46 | 10 | -24 |
| 8 |  |  |  |  |  |
|  | L Frontal Orbital Cortex | 2.85 | -14 | 14 | -24 |
| 8 |  |  |  |  |  |
|  | R Temporal Pole | 2.76 | 50 | 18 | -18 |
| 8 |  |  |  |  |  |
|  | L Frontal Orbital Cortex | 2.87 | -36 | 22 | -18 |
| 8 |  |  |  |  |  |
|  | R Temporal Occipital Fusiform Cortex | 2.59 | 38 | -48 | -16 |
| 8 |  |  |  |  |  |
|  | L Frontal Orbital Cortex | 2.92 | -28 | 36 | -16 |
| 8 |  |  |  |  |  |
|  | R Brain-Stem | 2.62 | 4 | -38 | -12 |
| 8 |  |  |  |  |  |
|  | L Frontal Orbital Cortex | 2.55 | -28 | 36 | -10 |
| 8 |  |  |  |  |  |
|  | R Middle Temporal Gyrus, posterior division | 2.82 | 48 | -28 | -6 |
| 8 |  |  |  |  |  |
|  | L Cingulate Gyrus, anterior division | 2.66 | -2 | 36 | -6 |
| 8 |  |  |  |  |  |
|  | L Lingual Gyrus | 2.58 | -4 | -86 | -4 |
| 8 |  |  |  |  |  |
|  | L Pallidum | 2.55 | -12 | -2 | -4 |
| 8 |  |  |  |  |  |
|  | R Caudate | 2.95 | 14 | 24 | -4 |
| 8 |  |  |  |  |  |
|  | R Lingual Gyrus | 2.61 | 22 | -46 | -2 |
| 8 |  |  |  |  |  |
|  | R Accumbens | 2.74 | 8 | 20 | -2 |
| 8 |  |  |  |  |  |
|  | R Putamen | 2.63 | 28 | 6 | 0 |
| 8 |  |  |  |  |  |
|  | L Frontal Pole | 2.54 | -50 | 38 | 0 |
| 8 |  |  |  |  |  |
|  | L Thalamus | 2.57 | -12 | -10 | 2 |
| 8 |  |  |  |  |  |
|  | R Middle Temporal Gyrus, temporooccipital part | 2.59 | 52 | -44 | 4 |
| 8 |  |  |  |  |  |
|  | L Frontal Pole | 2.59 | -44 | 36 | 4 |
| 8 |  |  |  |  |  |
|  | R Thalamus | 2.93 | 0 | -8 | 6 |
| 8 |  |  |  |  |  |
|  | R Caudate | 2.55 | 16 | 14 | 6 |
| 8 |  |  |  |  |  |
|  | R Thalamus | 2.54 | 8 | -22 | 10 |
| 8 |  |  |  |  |  |
|  | L Thalamus | 2.54 | -2 | -20 | 14 |
| 8 |  |  |  |  |  |
|  | R Paracingulate Gyrus | 2.56 | 10 | 46 | 20 |
| 8 |  |  |  |  |  |
|  | R Caudate | 2.76 | 14 | -10 | 22 |
| 8 |  |  |  |  |  |
|  | R Inferior Frontal Gyrus, pars opercularis | 2.87 | 46 | 14 | 22 |
| 8 |  |  |  |  |  |
|  | R Caudate | 2.64 | 16 | -14 | 24 |
| 8 |  |  |  |  |  |
|  | R Paracingulate Gyrus | 2.57 | 2 | 48 | 24 |
| 8 |  |  |  |  |  |
|  | L Frontal Pole | 2.75 | -2 | 58 | 24 |
| 8 |  |  |  |  |  |
|  | R Superior Frontal Gyrus | 2.58 | 6 | 44 | 38 |
| 8 |  |  |  |  |  |
|  | R Frontal Pole | 2.77 | 8 | 54 | 38 |
| 8 |  |  |  |  |  |
|  | L Precentral Gyrus | 2.67 | -46 | -4 | 40 |
| 8 |  |  |  |  |  |
|  | R Superior Frontal Gyrus | 2.58 | 12 | 16 | 60 |
| 8 |  |  |  |  |  |
|  | L Superior Frontal Gyrus | 2.53 | -22 | 14 | 62 |
| 8 |  |  |  |  |  |
|  | R Superior Frontal Gyrus | 2.60 | 6 | 28 | 64 |
| 8 |  |  |  |  |  |
|  | R Precentral Gyrus | 2.77 | 14 | -20 | 70 |
| 8 |  |  |  |  |  |
|  | L Superior Frontal Gyrus | 2.71 | -10 | 8 | 70 |

# Supplementary Table S27. [CT_CB3 _- CT_CB2_] > [UT_CB3 _- UT_CB2_]. Descriptive statistics for clusters and local maxima showing greater increases in activation from Convolved Block 2 to Convolved Block 3 of Certain-Threat anticipation relative to increases in activation from Convolved Block 2 to Convolved Block 3 of Uncertain-Threat anticipation (FDR *q*<0.05, whole-brain corrected). 

| ***mm***^***3***^ | **Label** | ***t*** | ***x*** | ***y*** | ***z*** |
| --- | --- | --- | --- | --- | --- |
| 29,952 |  |  |  |  |  |
|  | L Insular Cortex | 7.11 | -32 | 18 | 8 |
|  | L Frontal Operculum Cortex | 6.36 | -38 | 16 | 2 |
|  | L Cingulate Gyrus, anterior division | 6.02 | -8 | 14 | 36 |
|  | R Paracingulate Gyrus | 4.56 | 6 | 14 | 50 |
|  | L Paracingulate Gyrus | 5.58 | -4 | 12 | 46 |
|  | L Inferior Frontal Gyrus, pars opercularis | 3.44 | -52 | 10 | 4 |
|  | R Cingulate Gyrus, anterior division | 5.25 | 10 | 10 | 40 |
|  | L Precentral Gyrus | 6.34 | -58 | 8 | 14 |
|  | L Superior Frontal Gyrus | 3.65 | -14 | 8 | 68 |
|  | L Juxtapositional Lobule Cortex (formerly Supplementary Motor Cortex) | 6.40 | -6 | 4 | 56 |
|  | R Juxtapositional Lobule Cortex (formerly Supplementary Motor Cortex) | 6.56 | 6 | 4 | 58 |
|  | R Superior Frontal Gyrus | 3.76 | 14 | 2 | 68 |
|  | L Postcentral Gyrus | 5.09 | -46 | -10 | 30 |
|  | R Postcentral Gyrus | 8.36 | 36 | -26 | 56 |
|  | R Precentral Gyrus | 5.66 | 26 | -26 | 66 |
| 10,416 |  |  |  |  |  |
|  | R Putamen | 10.29 | 22 | 8 | 0 |
|  | R Caudate | 6.61 | 8 | 8 | 2 |
|  | R Accumbens | 4.24 | 8 | 6 | -8 |
|  | R Thalamus | 5.16 | 6 | -4 | -2 |
|  | R Amygdala | 7.18 | 28 | -10 | -12 |
|  | R Pallidum | 3.00 | 24 | -10 | 6 |
| 9,240 |  |  |  |  |  |
|  | R Occipital Fusiform Gyrus | 3.10 | 22 | -82 | -12 |
|  | R Lingual Gyrus | 6.34 | 10 | -86 | -8 |
|  | R Occipital Pole | 10.61 | 16 | -96 | 2 |
| 9,008 |  |  |  |  |  |
|  | L Caudate | 6.96 | -8 | 8 | 0 |
|  | L Putamen | 7.88 | -22 | 0 | 2 |
|  | L Amygdala | 7.77 | -26 | -8 | -12 |
|  | L Pallidum | 4.87 | -24 | -10 | 0 |
| 6,376 |  |  |  |  |  |
|  | L Inferior Temporal Gyrus, temporooccipital part | 3.28 | -48 | -46 | -28 |
|  | L Temporal Occipital Fusiform Cortex | 3.59 | -30 | -54 | -20 |
|  | L Occipital Fusiform Gyrus | 3.75 | -24 | -70 | -18 |
| 3,904 |  |  |  |  |  |
|  | L Thalamus | 5.49 | -4 | -22 | 0 |
|  | R Brain-Stem | 5.18 | 10 | -26 | -20 |
|  | L Brain-Stem | 6.36 | -6 | -34 | -8 |
| 3,408 |  |  |  |  |  |
|  | R Temporal Occipital Fusiform Cortex | 4.90 | 34 | -60 | -20 |
|  | R Occipital Fusiform Gyrus | 3.70 | 30 | -66 | -18 |
| 3,224 |  |  |  |  |  |
|  | L Frontal Pole | 5.57 | -36 | 48 | 30 |
|  | L Middle Frontal Gyrus | 4.32 | -40 | 36 | 30 |
| 2,152 |  |  |  |  |  |
|  | L Occipital Fusiform Gyrus | 5.31 | -22 | -84 | -10 |
|  | L Lingual Gyrus | 5.54 | -8 | -86 | -8 |
|  | L Occipital Pole | 5.78 | -12 | -92 | -10 |
| 2,048 |  |  |  |  |  |
|  | R Frontal Operculum Cortex | 5.69 | 40 | 24 | 4 |
|  | R Frontal Orbital Cortex | 4.55 | 36 | 24 | -4 |
|  | R Central Opercular Cortex | 2.89 | 40 | 8 | 6 |
| 1,336 |  |  |  |  |  |
|  | R Thalamus | 6.41 | 4 | -22 | -2 |
| 1,312 |  |  |  |  |  |
|  | L Precuneus Cortex | 5.74 | -8 | -74 | 42 |
|  | L Lateral Occipital Cortex, superior division | 3.73 | -8 | -74 | 54 |
|  | L Angular Gyrus | 4.05 | -36 | -56 | 38 |
|  | L Superior Parietal Lobule | 3.86 | -36 | -56 | 52 |
|  | L Lateral Occipital Cortex, superior division | 3.88 | -34 | -60 | 50 |
| 1,144 |  |  |  |  |  |
|  | L Frontal Pole | 4.96 | -16 | 68 | -6 |
| 1,112 |  |  |  |  |  |
|  | R Precuneus Cortex | 6.02 | 12 | -70 | 40 |
| 872 |  |  |  |  |  |
|  | R Frontal Pole | 4.48 | 36 | 46 | 32 |
| 760 |  |  |  |  |  |
|  | R Frontal Pole | 4.69 | 20 | 70 | -8 |
| 640 |  |  |  |  |  |
|  | L Angular Gyrus | 4.62 | -46 | -56 | 46 |
|  | L Lateral Occipital Cortex, superior division | 3.91 | -48 | -60 | 40 |
| 632 |  |  |  |  |  |
|  | R Precentral Gyrus | 4.22 | 64 | 8 | 16 |
| 400 |  |  |  |  |  |
|  | L Heschls Gyrus (includes H1 and H2) | 4.82 | -46 | -26 | 6 |
|  | L Planum Temporale | 3.77 | -50 | -30 | 8 |
| 400 |  |  |  |  |  |
|  | R Precentral Gyrus | 3.99 | 48 | -8 | 32 |
|  | R Postcentral Gyrus | 4.26 | 54 | -12 | 38 |
| 400 |  |  |  |  |  |
|  | L Supramarginal Gyrus, posterior division | 3.82 | -54 | -48 | 50 |
| 328 |  |  |  |  |  |
|  | R Brain-Stem | 4.01 | 8 | -40 | -52 |
|  | L Brain-Stem | 4.02 | -4 | -46 | -58 |
| 312 |  |  |  |  |  |
|  | L Parietal Operculum Cortex | 4.10 | -56 | -32 | 26 |
|  | L Supramarginal Gyrus, anterior division | 3.80 | -62 | -34 | 24 |
| 296 |  |  |  |  |  |
|  | L Brain-Stem | 5.09 | -4 | -40 | -48 |
| 232 |  |  |  |  |  |
|  | R Heschls Gyrus (includes H1 and H2) | 4.41 | 50 | -18 | 8 |
|  | R Planum Temporale | 3.46 | 60 | -18 | 6 |
| 224 |  |  |  |  |  |
|  | L Postcentral Gyrus | 3.94 | -60 | -22 | 30 |
|  | L Supramarginal Gyrus, anterior division | 3.44 | -62 | -24 | 26 |
| 192 |  |  |  |  |  |
|  | R Brain-Stem | 4.62 | 2 | -36 | -38 |
| 192 |  |  |  |  |  |
|  | R Occipital Fusiform Gyrus | 3.07 | 24 | -72 | -16 |
| 192 |  |  |  |  |  |
|  | R Superior Parietal Lobule | 4.03 | 26 | -42 | 62 |
| 168 |  |  |  |  |  |
|  | L Cingulate Gyrus, posterior division | 3.77 | -4 | -22 | 28 |
| 136 |  |  |  |  |  |
|  | L Inferior Temporal Gyrus, anterior division | 4.26 | -48 | -8 | -38 |
| 136 |  |  |  |  |  |
|  | L Supramarginal Gyrus, posterior division | 3.64 | -60 | -50 | 42 |
| 120 |  |  |  |  |  |
|  | L Precuneus Cortex | 3.27 | -4 | -64 | 58 |
|  | R Precuneus Cortex | 3.24 | 0 | -64 | 58 |
| 112 |  |  |  |  |  |
|  | R Cingulate Gyrus, posterior division | 4.12 | 4 | -22 | 30 |
| 112 |  |  |  |  |  |
|  | L Precentral Gyrus | 4.95 | -8 | -24 | 58 |
| 112 |  |  |  |  |  |
|  | L Precentral Gyrus | 4.46 | -6 | -22 | 66 |
| 104 |  |  |  |  |  |
|  | L Superior Frontal Gyrus | 3.81 | -22 | 2 | 60 |
| 80 |  |  |  |  |  |
|  | L Insular Cortex | 3.48 | -28 | 20 | -6 |
| 80 |  |  |  |  |  |
|  | R Frontal Pole | 3.45 | 30 | 40 | 36 |
| 80 |  |  |  |  |  |
|  | L Supramarginal Gyrus, anterior division | 3.42 | -38 | -38 | 38 |
| 72 |  |  |  |  |  |
|  | R Central Opercular Cortex | 3.87 | 52 | 4 | 4 |
| 72 |  |  |  |  |  |
|  | L Middle Frontal Gyrus | 3.89 | -42 | 20 | 36 |
| 72 |  |  |  |  |  |
|  | R Precuneus Cortex | 3.24 | 0 | -46 | 42 |
| 56 |  |  |  |  |  |
|  | R Middle Temporal Gyrus, anterior division | 3.24 | 56 | -4 | -34 |
| 56 |  |  |  |  |  |
|  | L Brain-Stem | 3.72 | -4 | -14 | -24 |
| 56 |  |  |  |  |  |
|  | L Putamen | 3.74 | -30 | -18 | -2 |
| 56 |  |  |  |  |  |
|  | R Precentral Gyrus | 3.77 | 6 | -22 | 66 |
| 48 |  |  |  |  |  |
|  | L Frontal Pole | 4.03 | -20 | 52 | -14 |
| 48 |  |  |  |  |  |
|  | R Frontal Pole | 3.26 | 2 | 70 | -4 |
| 48 |  |  |  |  |  |
|  | L Postcentral Gyrus | 2.95 | -58 | -10 | 18 |
| 48 |  |  |  |  |  |
|  | L Cingulate Gyrus, posterior division | 4.17 | -6 | -38 | 26 |
| 48 |  |  |  |  |  |
|  | R Precentral Gyrus | 3.17 | 8 | -20 | 50 |
| 48 |  |  |  |  |  |
|  | L Precuneus Cortex | 2.91 | -2 | -58 | 66 |
| 40 |  |  |  |  |  |
|  | L Occipital Pole | 3.55 | -12 | -94 | 0 |
| 40 |  |  |  |  |  |
|  | R Parietal Operculum Cortex | 2.97 | 56 | -28 | 22 |
| 40 |  |  |  |  |  |
|  | R Supramarginal Gyrus, anterior division | 3.63 | 60 | -22 | 24 |
| 40 |  |  |  |  |  |
|  | L Juxtapositional Lobule Cortex (formerly Supplementary Motor Cortex) | 3.32 | -6 | -10 | 48 |
| 32 |  |  |  |  |  |
|  | R Temporal Fusiform Cortex, posterior division | 2.99 | 34 | -14 | -32 |
| 32 |  |  |  |  |  |
|  | L Inferior Temporal Gyrus, posterior division | 3.51 | -64 | -22 | -28 |
| 32 |  |  |  |  |  |
|  | L Inferior Temporal Gyrus, posterior division | 3.12 | -56 | -44 | -24 |
|  | L Inferior Temporal Gyrus, temporooccipital part | 3.17 | -54 | -46 | -26 |
| 32 |  |  |  |  |  |
|  | L Middle Temporal Gyrus, posterior division | 3.00 | -58 | -26 | -12 |
| 32 |  |  |  |  |  |
|  | L Frontal Pole | 3.59 | -2 | 70 | -2 |
| 32 |  |  |  |  |  |
|  | R Putamen | 3.34 | 30 | -12 | 6 |
| 32 |  |  |  |  |  |
|  | L Planum Temporale | 3.21 | -60 | -28 | 10 |
| 32 |  |  |  |  |  |
|  | R Frontal Pole | 3.06 | 40 | 56 | 16 |
| 32 |  |  |  |  |  |
|  | L Central Opercular Cortex | 3.09 | -44 | -22 | 16 |
| 32 |  |  |  |  |  |
|  | R Frontal Pole | 3.55 | 32 | 36 | 26 |
| 32 |  |  |  |  |  |
|  | R Supramarginal Gyrus, posterior division | 3.24 | 66 | -38 | 34 |
| 32 |  |  |  |  |  |
|  | L Postcentral Gyrus | 3.44 | -34 | -28 | 50 |
| 24 |  |  |  |  |  |
|  | L Brain-Stem | 3.21 | -2 | -32 | -44 |
| 24 |  |  |  |  |  |
|  | R Middle Temporal Gyrus, anterior division | 2.98 | 50 | 0 | -34 |
| 24 |  |  |  |  |  |
|  | L Inferior Temporal Gyrus, posterior division | 3.02 | -50 | -34 | -28 |
| 24 |  |  |  |  |  |
|  | R Inferior Temporal Gyrus, temporooccipital part | 2.85 | 54 | -46 | -26 |
| 24 |  |  |  |  |  |
|  | L Middle Temporal Gyrus, posterior division | 2.91 | -66 | -24 | -18 |
| 24 |  |  |  |  |  |
|  | L Middle Temporal Gyrus, posterior division | 3.13 | -70 | -28 | -12 |
| 24 |  |  |  |  |  |
|  | R Planum Temporale | 3.30 | 58 | -24 | 8 |
| 24 |  |  |  |  |  |
|  | L Central Opercular Cortex | 3.26 | -52 | -2 | 8 |
| 24 |  |  |  |  |  |
|  | L Precentral Gyrus | 3.28 | -58 | 0 | 10 |
| 24 |  |  |  |  |  |
|  | L Central Opercular Cortex | 2.84 | -38 | 2 | 10 |
| 24 |  |  |  |  |  |
|  | L Supramarginal Gyrus, posterior division | 2.95 | -52 | -40 | 52 |
| 24 |  |  |  |  |  |
|  | R Precentral Gyrus | 3.16 | 32 | -6 | 54 |
| 24 |  |  |  |  |  |
|  | R Lateral Occipital Cortex, superior division | 2.88 | 10 | -72 | 62 |
| 16 |  |  |  |  |  |
|  | R Brain-Stem | 3.00 | 16 | -22 | -36 |
| 16 |  |  |  |  |  |
|  | R Brain-Stem | 2.86 | 10 | -38 | -28 |
| 16 |  |  |  |  |  |
|  | R Brain-Stem | 2.76 | 4 | -28 | -24 |
| 16 |  |  |  |  |  |
|  | L Inferior Temporal Gyrus, temporooccipital part | 2.97 | -58 | -50 | -22 |
| 16 |  |  |  |  |  |
|  | L Inferior Temporal Gyrus, posterior division | 2.89 | -50 | -40 | -22 |
| 16 |  |  |  |  |  |
|  | R Temporal Pole | 2.79 | 32 | 6 | -22 |
| 16 |  |  |  |  |  |
|  | L Middle Temporal Gyrus, posterior division | 3.29 | -70 | -20 | -14 |
| 16 |  |  |  |  |  |
|  | L Frontal Pole | 3.01 | -28 | 40 | -14 |
| 16 |  |  |  |  |  |
|  | L Planum Polare | 3.44 | -44 | -10 | -10 |
| 16 |  |  |  |  |  |
|  | R Insular Cortex | 3.00 | 38 | -8 | -10 |
| 16 |  |  |  |  |  |
|  | L Insular Cortex | 2.83 | -36 | 20 | -6 |
| 16 |  |  |  |  |  |
|  | L Occipital Pole | 3.14 | -8 | -98 | 6 |
| 16 |  |  |  |  |  |
|  | R Central Opercular Cortex | 3.25 | 44 | 6 | 8 |
| 16 |  |  |  |  |  |
|  | R Frontal Pole | 2.94 | 38 | 40 | 18 |
| 16 |  |  |  |  |  |
|  | R Caudate | 2.89 | 18 | 0 | 22 |
| 16 |  |  |  |  |  |
|  | R Angular Gyrus | 3.12 | 58 | -48 | 30 |
| 16 |  |  |  |  |  |
|  | R Cingulate Gyrus, anterior division | 3.27 | 4 | 0 | 32 |
| 16 |  |  |  |  |  |
|  | L Frontal Pole | 2.90 | -28 | 54 | 32 |
| 16 |  |  |  |  |  |
|  | R Frontal Pole | 3.01 | 24 | 48 | 36 |
| 16 |  |  |  |  |  |
|  | L Lateral Occipital Cortex, superior division | 2.88 | -56 | -64 | 38 |
| 16 |  |  |  |  |  |
|  | L Cingulate Gyrus, anterior division | 3.05 | -4 | -2 | 40 |
| 16 |  |  |  |  |  |
|  | R Postcentral Gyrus | 3.01 | 50 | -22 | 48 |
| 16 |  |  |  |  |  |
|  | L Postcentral Gyrus | 3.14 | -18 | -40 | 64 |
| 8 |  |  |  |  |  |
|  | L Temporal Fusiform Cortex, anterior division | 2.86 | -38 | -4 | -48 |
| 8 |  |  |  |  |  |
|  | R Brain-Stem | 3.03 | 4 | -46 | -46 |
| 8 |  |  |  |  |  |
|  | R Inferior Temporal Gyrus, anterior division | 2.95 | 50 | -2 | -46 |
| 8 |  |  |  |  |  |
|  | R Inferior Temporal Gyrus, anterior division | 3.04 | 46 | 4 | -46 |
| 8 |  |  |  |  |  |
|  | R Brain-Stem | 3.11 | 2 | -26 | -44 |
| 8 |  |  |  |  |  |
|  | R Inferior Temporal Gyrus, anterior division | 2.85 | 54 | -2 | -42 |
| 8 |  |  |  |  |  |
|  | L Brain-Stem | 2.87 | -4 | -24 | -40 |
| 8 |  |  |  |  |  |
|  | R Temporal Fusiform Cortex, posterior division | 2.80 | 36 | -18 | -38 |
| 8 |  |  |  |  |  |
|  | L Inferior Temporal Gyrus, posterior division | 2.74 | -54 | -16 | -36 |
| 8 |  |  |  |  |  |
|  | L Inferior Temporal Gyrus, anterior division | 2.74 | -56 | -6 | -36 |
| 8 |  |  |  |  |  |
|  | L Temporal Pole | 2.78 | -36 | 10 | -36 |
| 8 |  |  |  |  |  |
|  | L Parahippocampal Gyrus, anterior division | 2.86 | -24 | -20 | -32 |
| 8 |  |  |  |  |  |
|  | L Brain-Stem | 2.82 | -6 | -32 | -30 |
| 8 |  |  |  |  |  |
|  | L  Hippocampus | 2.99 | -30 | -10 | -26 |
| 8 |  |  |  |  |  |
|  | L Brain-Stem | 2.99 | -4 | -30 | -24 |
| 8 |  |  |  |  |  |
|  | R Subcallosal Cortex | 3.48 | 0 | 30 | -24 |
| 8 |  |  |  |  |  |
|  | L  Amygdala | 2.73 | -32 | -2 | -22 |
| 8 |  |  |  |  |  |
|  | L Frontal Orbital Cortex | 2.72 | -12 | 22 | -22 |
| 8 |  |  |  |  |  |
|  | R Occipital Fusiform Gyrus | 2.81 | 24 | -90 | -18 |
| 8 |  |  |  |  |  |
|  | L Frontal Pole | 3.20 | -38 | 56 | -16 |
| 8 |  |  |  |  |  |
|  | R Frontal Pole | 2.91 | 4 | 66 | -16 |
| 8 |  |  |  |  |  |
|  | R Occipital Fusiform Gyrus | 2.73 | 30 | -78 | -12 |
| 8 |  |  |  |  |  |
|  | L Planum Polare | 2.75 | -46 | -16 | -4 |
| 8 |  |  |  |  |  |
|  | R Frontal Pole | 2.72 | 10 | 72 | 0 |
| 8 |  |  |  |  |  |
|  | R Inferior Frontal Gyrus, pars opercularis | 2.89 | 54 | 10 | 4 |
| 8 |  |  |  |  |  |
|  | R Thalamus | 2.77 | 14 | -12 | 6 |
| 8 |  |  |  |  |  |
|  | L Thalamus | 2.77 | -10 | -10 | 6 |
| 8 |  |  |  |  |  |
|  | L Heschls Gyrus (includes H1 and H2) | 2.91 | -34 | -26 | 10 |
| 8 |  |  |  |  |  |
|  | R Heschls Gyrus (includes H1 and H2) | 2.84 | 38 | -24 | 10 |
| 8 |  |  |  |  |  |
|  | R Frontal Pole | 2.75 | 42 | 52 | 12 |
| 8 |  |  |  |  |  |
|  | L Planum Temporale | 2.74 | -58 | -32 | 14 |
| 8 |  |  |  |  |  |
|  | L Thalamus | 2.84 | -6 | -26 | 14 |
| 8 |  |  |  |  |  |
|  | L Caudate | 2.98 | -16 | 10 | 14 |
| 8 |  |  |  |  |  |
|  | R Frontal Pole | 2.72 | 24 | 50 | 14 |
| 8 |  |  |  |  |  |
|  | R Frontal Pole | 2.84 | 30 | 52 | 16 |
| 8 |  |  |  |  |  |
|  | L Planum Temporale | 3.08 | -48 | -38 | 18 |
| 8 |  |  |  |  |  |
|  | R Caudate | 2.78 | 12 | 8 | 20 |
| 8 |  |  |  |  |  |
|  | L Frontal Pole | 2.83 | -24 | 44 | 20 |
| 8 |  |  |  |  |  |
|  | L Postcentral Gyrus | 2.77 | -54 | -20 | 26 |
| 8 |  |  |  |  |  |
|  | R Precentral Gyrus | 2.77 | 60 | 4 | 32 |
| 8 |  |  |  |  |  |
|  | R Middle Frontal Gyrus | 2.73 | 32 | 32 | 32 |
| 8 |  |  |  |  |  |
|  | R Precuneus Cortex | 2.73 | 14 | -52 | 36 |
| 8 |  |  |  |  |  |
|  | R Precuneus Cortex | 2.71 | 6 | -46 | 40 |
| 8 |  |  |  |  |  |
|  | R Cingulate Gyrus, posterior division | 2.78 | 8 | -42 | 40 |
| 8 |  |  |  |  |  |
|  | L Supramarginal Gyrus, posterior division | 2.75 | -54 | -48 | 42 |
| 8 |  |  |  |  |  |
|  | R Postcentral Gyrus | 2.80 | 36 | -30 | 44 |
| 8 |  |  |  |  |  |
|  | R Lateral Occipital Cortex, superior division | 2.75 | 54 | -60 | 46 |
| 8 |  |  |  |  |  |
|  | R Superior Parietal Lobule | 2.84 | 40 | -48 | 50 |
| 8 |  |  |  |  |  |
|  | R Juxtapositional Lobule Cortex (formerly Supplementary Motor Cortex) | 2.83 | 10 | -10 | 50 |
| 8 |  |  |  |  |  |
|  | L Lateral Occipital Cortex, superior division | 2.71 | -26 | -80 | 52 |
| 8 |  |  |  |  |  |
|  | R Postcentral Gyrus | 2.93 | 20 | -42 | 66 |
| 8 |  |  |  |  |  |
|  | R Postcentral Gyrus | 2.98 | 4 | -34 | 68 |
| 8 |  |  |  |  |  |
|  | L Precentral Gyrus | 2.93 | -24 | -20 | 68 |

# Supplementary Table S28. [CT_CB2 _> CT_CB3_]. Descriptive statistics for clusters and local maxima showing greater activation for Convolved Block 2 relative to Convolved Block 3 of Certain-Threat anticipation (FDR *q*<0.05, whole-brain corrected). 

| ***mm***^***3***^ | **Label** | ***t*** | ***x*** | ***y*** | ***z*** |
| --- | --- | --- | --- | --- | --- |
| 132,784 |  |  |  |  |  |
|  | R Amygdala | 3.59 | 12 | -8 | -16 |
|  | L Parahippocampal Gyrus, anterior division | 3.30 | -20 | -20 | -22 |
|  | R Hippocampus | 4.54 | 22 | -20 | -16 |
|  | L Postcentral Gyrus | 8.31 | -38 | -24 | 68 |
|  | R Inferior Temporal Gyrus, posterior division | 3.15 | 44 | -24 | -24 |
|  | L Hippocampus | 5.59 | -20 | -28 | -12 |
|  | L Precentral Gyrus | 4.15 | -10 | -28 | 80 |
|  | L Thalamus | 4.75 | -8 | -34 | 2 |
|  | R Thalamus | 4.57 | 14 | -34 | -2 |
|  | L Parahippocampal Gyrus, posterior division | 6.27 | -26 | -36 | -16 |
|  | R Parahippocampal Gyrus, posterior division | 8.46 | 30 | -36 | -14 |
|  | R Temporal Fusiform Cortex, posterior division | 7.52 | 30 | -38 | -22 |
|  | R Lingual Gyrus | 9.30 | 32 | -40 | -10 |
|  | L Temporal Fusiform Cortex, posterior division | 6.80 | -34 | -44 | -20 |
|  | L Cingulate Gyrus, posterior division | 6.29 | -2 | -46 | 12 |
|  | R Cingulate Gyrus, posterior division | 6.76 | 8 | -46 | 4 |
|  | L Temporal Occipital Fusiform Cortex | 7.49 | -28 | -52 | -10 |
|  | R Temporal Occipital Fusiform Cortex | 6.88 | 24 | -52 | -12 |
|  | R Superior Parietal Lobule | 3.33 | 30 | -54 | 60 |
|  | L Lingual Gyrus | 8.27 | -22 | -56 | 2 |
|  | R Angular Gyrus | 3.62 | 52 | -56 | 28 |
|  | L Superior Parietal Lobule | 5.44 | -24 | -58 | 62 |
|  | L Middle Temporal Gyrus, temporooccipital part | 4.01 | -56 | -58 | -8 |
|  | L Inferior Temporal Gyrus, temporooccipital part | 6.81 | -42 | -62 | -8 |
|  | L Precuneus Cortex | 6.00 | -10 | -62 | 12 |
|  | L Intracalcarine Cortex | 7.40 | -18 | -64 | 6 |
|  | L Lateral Occipital Cortex, inferior division | 7.13 | -44 | -64 | -10 |
|  | R Precuneus Cortex | 7.67 | 10 | -64 | 18 |
|  | R Occipital Fusiform Gyrus | 5.62 | 22 | -66 | -12 |
|  | L Occipital Fusiform Gyrus | 6.10 | -36 | -74 | -14 |
|  | R Lateral Occipital Cortex, inferior division | 6.04 | 50 | -76 | 12 |
|  | R Intracalcarine Cortex | 7.68 | 14 | -78 | 8 |
|  | L Cuneal Cortex | 8.06 | -2 | -84 | 36 |
|  | R Lateral Occipital Cortex, superior division | 6.25 | 34 | -84 | 32 |
|  | R Cuneal Cortex | 8.62 | 6 | -86 | 38 |
|  | R Supracalcarine Cortex | 7.29 | 2 | -86 | 12 |
|  | L Lateral Occipital Cortex, superior division | 6.94 | -14 | -88 | 40 |
|  | L Occipital Pole | 8.80 | -6 | -90 | 34 |
|  | R Occipital Pole | 7.13 | 10 | -92 | 32 |
| 10,656 |  |  |  |  |  |
|  | L Frontal Orbital Cortex | 3.70 | -40 | 22 | -16 |
|  | L Temporal Pole | 5.97 | -36 | 18 | -26 |
|  | L Superior Temporal Gyrus, anterior division | 5.39 | -58 | 0 | -14 |
|  | L Planum Polare | 2.80 | -56 | -2 | -2 |
|  | L Middle Temporal Gyrus, anterior division | 5.01 | -54 | -4 | -18 |
|  | L Superior Temporal Gyrus, posterior division | 4.04 | -56 | -24 | -4 |
|  | L Middle Temporal Gyrus, posterior division | 3.78 | -68 | -42 | 2 |
|  | L Middle Temporal Gyrus, temporooccipital part | 3.05 | -62 | -44 | 2 |
| 8,272 |  |  |  |  |  |
|  | R Frontal Pole | 6.56 | 50 | 36 | 18 |
|  | R Middle Frontal Gyrus | 5.73 | 50 | 32 | 22 |
|  | R Frontal Orbital Cortex | 3.48 | 48 | 28 | -8 |
|  | R Inferior Frontal Gyrus, pars opercularis | 6.47 | 48 | 14 | 30 |
| 6,528 |  |  |  |  |  |
|  | R Frontal Orbital Cortex | 3.13 | 30 | 24 | -20 |
|  | R Temporal Pole | 6.29 | 48 | 20 | -22 |
|  | R Superior Temporal Gyrus, anterior division | 5.36 | 60 | 2 | -10 |
|  | R Middle Temporal Gyrus, anterior division | 4.48 | 60 | -2 | -16 |
|  | R Middle Temporal Gyrus, posterior division | 4.12 | 68 | -10 | -10 |
|  | R Planum Temporale | 3.56 | 66 | -10 | 6 |
| 5,536 |  |  |  |  |  |
|  | R Frontal Pole | 4.81 | 12 | 46 | 50 |
|  | R Superior Frontal Gyrus | 6.44 | 22 | 30 | 48 |
|  | R Middle Frontal Gyrus | 4.77 | 36 | 16 | 56 |
| 4,904 |  |  |  |  |  |
|  | L Frontal Pole | 4.64 | -48 | 44 | -12 |
|  | L Frontal Orbital Cortex | 4.33 | -48 | 34 | -8 |
|  | L Frontal Operculum Cortex | 2.68 | -46 | 28 | -2 |
|  | L Inferior Frontal Gyrus, pars triangularis | 3.70 | -50 | 26 | 24 |
|  | L Middle Frontal Gyrus | 4.20 | -48 | 22 | 28 |
|  | L Inferior Frontal Gyrus, pars opercularis | 4.20 | -46 | 8 | 28 |
| 3,768 |  |  |  |  |  |
|  | L Frontal Pole | 5.14 | -8 | 66 | 22 |
|  | R Superior Frontal Gyrus | 2.91 | 0 | 42 | 42 |
|  | L Superior Frontal Gyrus | 3.40 | -16 | 36 | 56 |
| 2,352 |  |  |  |  |  |
|  | L Frontal Pole | 2.97 | -2 | 60 | -14 |
|  | R Frontal Pole | 4.66 | 8 | 60 | 0 |
|  | R Frontal Medial Cortex | 3.71 | 10 | 54 | -8 |
|  | L Frontal Medial Cortex | 4.45 | -8 | 46 | -10 |
|  | L Paracingulate Gyrus | 4.19 | -10 | 46 | -6 |
|  | R Paracingulate Gyrus | 2.99 | 6 | 42 | -8 |
| 1,880 |  |  |  |  |  |
|  | R Postcentral Gyrus | 4.77 | 58 | -18 | 38 |
|  | R Supramarginal Gyrus, anterior division | 4.60 | 54 | -26 | 46 |
| 1,048 |  |  |  |  |  |
|  | L Superior Frontal Gyrus | 4.04 | -22 | 28 | 50 |
|  | L Middle Frontal Gyrus | 3.08 | -28 | 18 | 56 |
| 664 |  |  |  |  |  |
|  | R Frontal Pole | 4.77 | 10 | 68 | 18 |
| 568 |  |  |  |  |  |
|  | L Temporal Pole | 3.18 | -24 | 4 | -26 |
|  | L Amygdala | 3.35 | -22 | 2 | -24 |
|  | L Amygdala | 3.61 | -28 | 0 | -28 |
|  | L Parahippocampal Gyrus, anterior division | 4.16 | -18 | -12 | -28 |
|  | L Hippocampus | 3.06 | -18 | -16 | -22 |
| 504 |  |  |  |  |  |
|  | R Precentral Gyrus | 4.73 | 6 | -24 | 80 |
| 472 |  |  |  |  |  |
|  | L Temporal Fusiform Cortex, posterior division | 5.13 | -40 | -14 | -28 |
| 456 |  |  |  |  |  |
|  | R Brain-Stem | 3.90 | 10 | -40 | -58 |
| 400 |  |  |  |  |  |
|  | R Supramarginal Gyrus, posterior division | 4.15 | 42 | -40 | 40 |
| 352 |  |  |  |  |  |
|  | R Middle Temporal Gyrus, temporooccipital part | 4.60 | 58 | -48 | -8 |
| 312 |  |  |  |  |  |
|  | R Subcallosal Cortex | 4.16 | 0 | 10 | -8 |
| 304 |  |  |  |  |  |
|  | L Postcentral Gyrus | 3.85 | -64 | -14 | 34 |
| 288 |  |  |  |  |  |
|  | L Brain-Stem | 3.81 | -8 | -36 | -60 |
| 240 |  |  |  |  |  |
|  | R Insular Cortex | 4.74 | 38 | -6 | 12 |
| 232 |  |  |  |  |  |
|  | R Paracingulate Gyrus | 3.70 | 6 | 46 | 22 |
| 192 |  |  |  |  |  |
|  | L Superior Frontal Gyrus | 4.30 | -4 | 18 | 62 |
| 176 |  |  |  |  |  |
|  | L Postcentral Gyrus | 3.59 | -60 | -26 | 42 |
| 168 |  |  |  |  |  |
|  | R Parahippocampal Gyrus, anterior division | 3.99 | 18 | -8 | -28 |
| 160 |  |  |  |  |  |
|  | R Middle Temporal Gyrus, posterior division | 4.23 | 48 | -36 | -2 |
| 160 |  |  |  |  |  |
|  | L Angular Gyrus | 3.89 | -44 | -58 | 24 |
| 152 |  |  |  |  |  |
|  | R Brain-Stem | 3.69 | 0 | -38 | -22 |
| 144 |  |  |  |  |  |
|  | R Brain-Stem | 4.20 | 2 | -46 | -52 |
| 104 |  |  |  |  |  |
|  | R Temporal Pole | 3.08 | 26 | 12 | -44 |
| 104 |  |  |  |  |  |
|  | L Frontal Pole | 3.60 | -20 | 60 | -8 |
| 96 |  |  |  |  |  |
|  | R Subcallosal Cortex | 4.24 | 4 | 30 | -20 |
| 96 |  |  |  |  |  |
|  | R Occipital Fusiform Gyrus | 3.44 | 38 | -64 | -12 |
| 96 |  |  |  |  |  |
|  | L Middle Frontal Gyrus | 3.47 | -40 | 12 | 46 |
| 96 |  |  |  |  |  |
|  | R Postcentral Gyrus | 3.04 | 6 | -42 | 72 |
| 88 |  |  |  |  |  |
|  | R Brain-Stem | 3.32 | 2 | -36 | -66 |
| 88 |  |  |  |  |  |
|  | R Planum Polare | 3.95 | 50 | 0 | -6 |
| 80 |  |  |  |  |  |
|  | R Brain-Stem | 3.67 | 16 | -32 | -28 |
| 80 |  |  |  |  |  |
|  | L Insular Cortex | 3.61 | -36 | -8 | 12 |
| 80 |  |  |  |  |  |
|  | L Angular Gyrus | 3.25 | -60 | -56 | 22 |
| 80 |  |  |  |  |  |
|  | R Superior Frontal Gyrus | 3.12 | 24 | 8 | 52 |
| 72 |  |  |  |  |  |
|  | R Middle Temporal Gyrus, temporooccipital part | 3.05 | 68 | -40 | -10 |
| 72 |  |  |  |  |  |
|  | L Insular Cortex | 2.93 | -40 | -6 | 2 |
| 72 |  |  |  |  |  |
|  | R Postcentral Gyrus | 3.16 | 20 | -30 | 78 |
| 64 |  |  |  |  |  |
|  | R Frontal Pole | 3.34 | 12 | 60 | -22 |
| 64 |  |  |  |  |  |
|  | R Paracingulate Gyrus | 2.86 | 10 | 44 | -4 |
| 64 |  |  |  |  |  |
|  | L Central Opercular Cortex | 3.03 | -56 | -14 | 12 |
| 64 |  |  |  |  |  |
|  | R Postcentral Gyrus | 3.75 | 68 | -10 | 24 |
| 64 |  |  |  |  |  |
|  | L Cingulate Gyrus, posterior division | 3.17 | -4 | -42 | 36 |
| 56 |  |  |  |  |  |
|  | R Frontal Orbital Cortex | 3.59 | 14 | 16 | -22 |
| 56 |  |  |  |  |  |
|  | L Hippocampus | 3.86 | -14 | -12 | -18 |
| 56 |  |  |  |  |  |
|  | L Subcallosal Cortex | 3.08 | -2 | 28 | -12 |
| 56 |  |  |  |  |  |
|  | L Frontal Pole | 3.89 | -46 | 44 | 8 |
| 56 |  |  |  |  |  |
|  | L Cingulate Gyrus, anterior division | 2.81 | -8 | 44 | 8 |
| 48 |  |  |  |  |  |
|  | L Brain-Stem | 3.49 | -4 | -42 | -26 |
| 48 |  |  |  |  |  |
|  | R Temporal Fusiform Cortex, posterior division | 3.16 | 42 | -12 | -26 |
| 48 |  |  |  |  |  |
|  | R Frontal Orbital Cortex | 2.90 | 16 | 8 | -22 |
| 48 |  |  |  |  |  |
|  | R Cingulate Gyrus, anterior division | 2.76 | 4 | 42 | 6 |
| 48 |  |  |  |  |  |
|  | L Paracingulate Gyrus | 2.68 | -2 | 50 | 10 |
| 48 |  |  |  |  |  |
|  | R Cingulate Gyrus, posterior division | 3.41 | 6 | -34 | 38 |
| 40 |  |  |  |  |  |
|  | L Frontal Orbital Cortex | 2.77 | -22 | 14 | -26 |
| 40 |  |  |  |  |  |
|  | L Frontal Pole | 2.82 | -8 | 58 | -24 |
| 40 |  |  |  |  |  |
|  | R Middle Temporal Gyrus, posterior division | 2.90 | 62 | -12 | -22 |
| 40 |  |  |  |  |  |
|  | R Paracingulate Gyrus | 3.25 | 10 | 48 | 8 |
| 40 |  |  |  |  |  |
|  | R Central Opercular Cortex | 3.04 | 64 | -12 | 12 |
| 40 |  |  |  |  |  |
|  | R Cingulate Gyrus, posterior division | 3.09 | 2 | -48 | 30 |
| 40 |  |  |  |  |  |
|  | L Frontal Pole | 2.67 | -18 | 42 | 36 |
| 32 |  |  |  |  |  |
|  | L Temporal Fusiform Cortex, posterior division | 3.22 | -40 | -16 | -36 |
| 32 |  |  |  |  |  |
|  | L Frontal Medial Cortex | 3.50 | -8 | 40 | -22 |
| 32 |  |  |  |  |  |
|  | L Planum Polare | 2.72 | -42 | 2 | -18 |
| 32 |  |  |  |  |  |
|  | R Temporal Pole | 3.87 | 48 | 8 | -12 |
| 32 |  |  |  |  |  |
|  | R Subcallosal Cortex | 3.11 | 4 | 20 | -10 |
| 24 |  |  |  |  |  |
|  | L Temporal Fusiform Cortex, anterior division | 3.09 | -38 | -8 | -44 |
| 24 |  |  |  |  |  |
|  | L Frontal Medial Cortex | 2.53 | -6 | 48 | -24 |
| 24 |  |  |  |  |  |
|  | L Subcallosal Cortex | 2.99 | -10 | 28 | -20 |
| 24 |  |  |  |  |  |
|  | R Planum Polare | 3.09 | 44 | 2 | -12 |
| 24 |  |  |  |  |  |
|  | R Frontal Pole | 3.02 | 30 | 62 | -6 |
| 24 |  |  |  |  |  |
|  | R Cingulate Gyrus, anterior division | 3.06 | 4 | 38 | -2 |
| 24 |  |  |  |  |  |
|  | R Inferior Frontal Gyrus, pars opercularis | 2.80 | 60 | 20 | 14 |
| 24 |  |  |  |  |  |
|  | L Lateral Occipital Cortex, superior division | 3.09 | -56 | -70 | 16 |
| 24 |  |  |  |  |  |
|  | R Frontal Pole | 3.03 | 4 | 64 | 30 |
| 24 |  |  |  |  |  |
|  | R Middle Frontal Gyrus | 3.65 | 52 | 26 | 38 |
| 24 |  |  |  |  |  |
|  | L Postcentral Gyrus | 2.97 | -40 | -28 | 40 |
| 24 |  |  |  |  |  |
|  | L Precuneus Cortex | 2.85 | -10 | -54 | 46 |
| 24 |  |  |  |  |  |
|  | L Middle Frontal Gyrus | 3.05 | -38 | 10 | 54 |
| 16 |  |  |  |  |  |
|  | L Temporal Fusiform Cortex, posterior division | 2.97 | -32 | -12 | -40 |
| 16 |  |  |  |  |  |
|  | L Inferior Temporal Gyrus, posterior division | 2.96 | -46 | -18 | -34 |
| 16 |  |  |  |  |  |
|  | L Inferior Temporal Gyrus, posterior division | 2.53 | -44 | -32 | -24 |
| 16 |  |  |  |  |  |
|  | L Inferior Temporal Gyrus, posterior division | 3.06 | -48 | -30 | -24 |
| 16 |  |  |  |  |  |
|  | L Brain-Stem | 3.33 | -14 | -22 | -22 |
| 16 |  |  |  |  |  |
|  | R Subcallosal Cortex | 2.92 | 8 | 12 | -18 |
| 16 |  |  |  |  |  |
|  | L Inferior Temporal Gyrus, temporooccipital part | 2.74 | -60 | -60 | -16 |
| 16 |  |  |  |  |  |
|  | R Middle Temporal Gyrus, posterior division | 2.65 | 48 | -16 | -14 |
| 16 |  |  |  |  |  |
|  | R Frontal Pole | 2.63 | 20 | 58 | -6 |
| 16 |  |  |  |  |  |
|  | R Middle Temporal Gyrus, temporooccipital part | 2.77 | 70 | -42 | -4 |
| 16 |  |  |  |  |  |
|  | L Frontal Pole | 2.67 | -42 | 58 | -4 |
| 16 |  |  |  |  |  |
|  | L Frontal Pole | 2.73 | -38 | 54 | -2 |
| 16 |  |  |  |  |  |
|  | L Planum Polare | 2.80 | -50 | -8 | 0 |
| 16 |  |  |  |  |  |
|  | R Thalamus | 2.81 | 16 | -30 | 6 |
| 16 |  |  |  |  |  |
|  | L Insular Cortex | 2.73 | -42 | -10 | 8 |
| 16 |  |  |  |  |  |
|  | L Frontal Pole | 2.79 | -8 | 62 | 8 |
| 16 |  |  |  |  |  |
|  | L Central Opercular Cortex | 3.21 | -44 | -12 | 16 |
| 16 |  |  |  |  |  |
|  | R Frontal Pole | 2.69 | 22 | 58 | 18 |
| 16 |  |  |  |  |  |
|  | R Frontal Pole | 2.61 | 4 | 60 | 20 |
| 16 |  |  |  |  |  |
|  | L Inferior Frontal Gyrus, pars opercularis | 2.93 | -36 | 14 | 26 |
| 16 |  |  |  |  |  |
|  | L Middle Frontal Gyrus | 3.47 | -34 | 2 | 50 |
| 16 |  |  |  |  |  |
|  | L Middle Frontal Gyrus | 2.73 | -30 | 16 | 58 |
| 16 |  |  |  |  |  |
|  | L Superior Parietal Lobule | 2.62 | -18 | -56 | 70 |
| 8 |  |  |  |  |  |
|  | R Brain-Stem | 2.93 | 4 | -46 | -66 |
| 8 |  |  |  |  |  |
|  | L Inferior Temporal Gyrus, anterior division | 2.53 | -52 | -6 | -44 |
| 8 |  |  |  |  |  |
|  | L Temporal Pole | 2.59 | -28 | 8 | -34 |
| 8 |  |  |  |  |  |
|  | L Temporal Fusiform Cortex, anterior division | 2.67 | -36 | -8 | -32 |
| 8 |  |  |  |  |  |
|  | L Temporal Pole | 2.67 | -24 | 10 | -32 |
| 8 |  |  |  |  |  |
|  | L Frontal Pole | 2.50 | -12 | 40 | -28 |
| 8 |  |  |  |  |  |
|  | R Inferior Temporal Gyrus, posterior division | 2.56 | 52 | -34 | -26 |
| 8 |  |  |  |  |  |
|  | R Middle Temporal Gyrus, posterior division | 2.48 | 62 | -16 | -26 |
| 8 |  |  |  |  |  |
|  | R Hippocampus | 2.67 | 24 | -10 | -24 |
| 8 |  |  |  |  |  |
|  | R Frontal Medial Cortex | 2.48 | 0 | 50 | -24 |
| 8 |  |  |  |  |  |
|  | R Hippocampus | 2.63 | 32 | -14 | -22 |
| 8 |  |  |  |  |  |
|  | L Hippocampus | 2.51 | -16 | -8 | -22 |
| 8 |  |  |  |  |  |
|  | L Frontal Orbital Cortex | 2.55 | -28 | 34 | -22 |
| 8 |  |  |  |  |  |
|  | R Planum Polare | 2.74 | 42 | 2 | -20 |
| 8 |  |  |  |  |  |
|  | L Frontal Orbital Cortex | 2.57 | -24 | 8 | -20 |
| 8 |  |  |  |  |  |
|  | L Subcallosal Cortex | 2.66 | -4 | 24 | -18 |
| 8 |  |  |  |  |  |
|  | L Frontal Pole | 2.51 | -26 | 54 | -18 |
| 8 |  |  |  |  |  |
|  | R Frontal Pole | 2.50 | 14 | 56 | -18 |
| 8 |  |  |  |  |  |
|  | L Amygdala | 2.82 | -14 | -4 | -16 |
| 8 |  |  |  |  |  |
|  | R Subcallosal Cortex | 2.69 | 12 | 14 | -16 |
| 8 |  |  |  |  |  |
|  | R Subcallosal Cortex | 3.01 | 4 | 26 | -16 |
| 8 |  |  |  |  |  |
|  | R Frontal Pole | 2.48 | 18 | 50 | -16 |
| 8 |  |  |  |  |  |
|  | R Inferior Temporal Gyrus, temporooccipital part | 2.52 | 62 | -54 | -14 |
| 8 |  |  |  |  |  |
|  | L Frontal Pole | 3.13 | -12 | 60 | -14 |
| 8 |  |  |  |  |  |
|  | L Insular Cortex | 2.86 | -40 | 2 | -12 |
| 8 |  |  |  |  |  |
|  | R Cingulate Gyrus, anterior division | 2.56 | 6 | 38 | -6 |
| 8 |  |  |  |  |  |
|  | L Middle Temporal Gyrus, temporooccipital part | 2.54 | -66 | -50 | -4 |
| 8 |  |  |  |  |  |
|  | L Pallidum | 2.84 | -24 | -16 | -4 |
| 8 |  |  |  |  |  |
|  | R Subcallosal Cortex | 2.84 | 2 | 24 | -4 |
| 8 |  |  |  |  |  |
|  | L Subcallosal Cortex | 2.55 | -2 | 16 | -2 |
| 8 |  |  |  |  |  |
|  | L Frontal Pole | 2.58 | -26 | 54 | -2 |
| 8 |  |  |  |  |  |
|  | L Frontal Pole | 2.50 | -6 | 60 | -2 |
| 8 |  |  |  |  |  |
|  | R Superior Temporal Gyrus, posterior division | 2.60 | 66 | -18 | 0 |
| 8 |  |  |  |  |  |
|  | R Insular Cortex | 2.54 | 44 | 0 | 0 |
| 8 |  |  |  |  |  |
|  | R Frontal Pole | 2.75 | 26 | 62 | 4 |
| 8 |  |  |  |  |  |
|  | R Paracingulate Gyrus | 2.81 | 6 | 54 | 8 |
| 8 |  |  |  |  |  |
|  | R Cingulate Gyrus, anterior division | 2.51 | 12 | 42 | 12 |
| 8 |  |  |  |  |  |
|  | R Central Opercular Cortex | 2.61 | 48 | -8 | 16 |
| 8 |  |  |  |  |  |
|  | R Angular Gyrus | 2.62 | 48 | -52 | 20 |
| 8 |  |  |  |  |  |
|  | L Central Opercular Cortex | 2.86 | -40 | -18 | 20 |
| 8 |  |  |  |  |  |
|  | L Inferior Frontal Gyrus, pars opercularis | 2.59 | -44 | 14 | 22 |
| 8 |  |  |  |  |  |
|  | L Lateral Occipital Cortex, superior division | 2.62 | -50 | -62 | 26 |
| 8 |  |  |  |  |  |
|  | R Middle Frontal Gyrus | 2.51 | 50 | 22 | 32 |
| 8 |  |  |  |  |  |
|  | R Frontal Pole | 2.64 | 10 | 56 | 40 |
| 8 |  |  |  |  |  |
|  | R Precuneus Cortex | 2.55 | 0 | -56 | 42 |
| 8 |  |  |  |  |  |
|  | R Middle Frontal Gyrus | 2.49 | 40 | 10 | 44 |
| 8 |  |  |  |  |  |
|  | R Superior Parietal Lobule | 2.58 | 28 | -52 | 52 |
| 8 |  |  |  |  |  |
|  | L Superior Frontal Gyrus | 2.88 | -22 | -2 | 52 |
| 8 |  |  |  |  |  |
|  | L Superior Frontal Gyrus | 2.65 | -16 | 18 | 58 |
| 8 |  |  |  |  |  |
|  | R Superior Frontal Gyrus | 2.74 | 12 | 20 | 60 |
| 8 |  |  |  |  |  |
|  | L Precentral Gyrus | 2.72 | -28 | -20 | 66 |
| 8 |  |  |  |  |  |
|  | L Postcentral Gyrus | 2.58 | -12 | -34 | 78 |

# Supplementary Table S29. [UT_CB2 _> UT_CB3_]. Descriptive statistics for clusters and local maxima showing greater activation for Convolved Block 2 relative to Convolved Block 3 of Uncertain-Threat anticipation (FDR *q*<0.05, whole-brain corrected). 

| ***mm***^***3***^ | **Label** | ***t*** | ***x*** | ***y*** | ***z*** |
| --- | --- | --- | --- | --- | --- |
| 50,152 |  |  |  |  |  |
|  | R Temporal Fusiform Cortex, posterior division | 4.42 | 32 | -34 | -20 |
|  | R Parahippocampal Gyrus, posterior division | 3.46 | 14 | -34 | -12 |
|  | L Temporal Fusiform Cortex, posterior division | 4.67 | -28 | -36 | -22 |
|  | R Temporal Occipital Fusiform Cortex | 6.47 | 32 | -50 | -20 |
|  | L Temporal Occipital Fusiform Cortex | 5.00 | -32 | -52 | -18 |
|  | L Inferior Temporal Gyrus, temporooccipital part | 3.55 | -52 | -52 | -26 |
|  | R Lingual Gyrus | 5.27 | 16 | -70 | -12 |
|  | L Occipital Fusiform Gyrus | 5.56 | -18 | -74 | -14 |
|  | R Occipital Fusiform Gyrus | 6.36 | 22 | -80 | -18 |
|  | L Occipital Pole | 5.53 | -12 | -92 | -12 |
|  | R Occipital Pole | 2.95 | 6 | -92 | -4 |
| 30,432 |  |  |  |  |  |
|  | L Precentral Gyrus | 4.50 | -30 | -26 | 54 |
|  | L Postcentral Gyrus | 5.28 | -38 | -28 | 50 |
|  | L Supramarginal Gyrus, anterior division | 4.50 | -46 | -40 | 44 |
|  | L Superior Parietal Lobule | 4.33 | -38 | -40 | 44 |
|  | L Supramarginal Gyrus, posterior division | 4.79 | -42 | -44 | 44 |
|  | R Cingulate Gyrus, posterior division | 3.62 | 0 | -48 | 14 |
|  | L Precuneus Cortex | 4.53 | -4 | -60 | 38 |
|  | L Lingual Gyrus | 4.42 | -10 | -62 | 0 |
|  | L Lateral Occipital Cortex, superior division | 5.95 | -24 | -64 | 48 |
|  | R Intracalcarine Cortex | 6.81 | 8 | -66 | 16 |
|  | R Precuneus Cortex | 5.27 | 10 | -66 | 22 |
|  | R Lingual Gyrus | 3.25 | 10 | -66 | -4 |
|  | R Cuneal Cortex | 5.06 | 6 | -70 | 22 |
|  | R Supracalcarine Cortex | 6.02 | 0 | -72 | 16 |
|  | L Intracalcarine Cortex | 6.06 | -12 | -74 | 12 |
|  | L Cuneal Cortex | 3.26 | -2 | -80 | 34 |
| 2,288 |  |  |  |  |  |
|  | L Frontal Pole | 4.51 | -10 | 72 | -6 |
|  | R Frontal Pole | 5.55 | 8 | 72 | 2 |
| 1,704 |  |  |  |  |  |
|  | R Lateral Occipital Cortex, superior division | 5.76 | 30 | -64 | 40 |
| 1,680 |  |  |  |  |  |
|  | R Frontal Pole | 3.93 | 18 | 38 | 50 |
|  | R Superior Frontal Gyrus | 5.12 | 24 | 32 | 50 |
|  | R Middle Frontal Gyrus | 4.29 | 32 | 16 | 58 |
| 1,216 |  |  |  |  |  |
|  | R Middle Frontal Gyrus | 4.36 | 46 | 34 | 32 |
| 1,008 |  |  |  |  |  |
|  | L Middle Frontal Gyrus | 4.55 | -44 | 16 | 34 |
|  | L Precentral Gyrus | 3.42 | -42 | 4 | 32 |
| 760 |  |  |  |  |  |
|  | L Middle Temporal Gyrus, posterior division | 3.81 | -64 | -40 | 2 |
| 712 |  |  |  |  |  |
|  | L Middle Frontal Gyrus | 4.22 | -46 | 32 | 20 |
|  | L Inferior Frontal Gyrus, pars triangularis | 3.95 | -48 | 26 | 20 |
| 648 |  |  |  |  |  |
|  | L Lateral Occipital Cortex, superior division | 4.03 | -38 | -86 | 20 |
| 608 |  |  |  |  |  |
|  | R Postcentral Gyrus | 4.28 | 58 | -8 | 26 |
| 552 |  |  |  |  |  |
|  | R Lateral Occipital Cortex, superior division | 3.62 | 36 | -80 | 20 |
| 544 |  |  |  |  |  |
|  | R Brain-Stem | 4.03 | 0 | -42 | -58 |
|  | L Brain-Stem | 4.18 | -2 | -48 | -62 |
| 488 |  |  |  |  |  |
|  | R Angular Gyrus | 4.02 | 46 | -56 | 32 |
| 488 |  |  |  |  |  |
|  | R Precentral Gyrus | 3.15 | 0 | -28 | 70 |
|  | R Postcentral Gyrus | 4.26 | 4 | -34 | 66 |
| 448 |  |  |  |  |  |
|  | R Accumbens | 4.84 | 8 | 6 | -6 |
| 440 |  |  |  |  |  |
|  | L Postcentral Gyrus | 4.30 | -62 | -8 | 30 |
| 384 |  |  |  |  |  |
|  | R Amygdala | 3.35 | 14 | -8 | -16 |
|  | R Hippocampus | 5.15 | 18 | -16 | -16 |
| 376 |  |  |  |  |  |
|  | L Postcentral Gyrus | 4.31 | -6 | -36 | 74 |
| 296 |  |  |  |  |  |
|  | L Brain-Stem | 3.88 | -6 | -40 | -46 |
|  | R Brain-Stem | 3.81 | 0 | -42 | -42 |
| 288 |  |  |  |  |  |
|  | R Hippocampus | 4.34 | 28 | -32 | -6 |
|  | R Thalamus | 3.67 | 18 | -34 | -2 |
| 264 |  |  |  |  |  |
|  | L Thalamus | 4.59 | -22 | -34 | -2 |
| 256 |  |  |  |  |  |
|  | L Thalamus | 4.18 | -16 | -24 | 4 |
| 248 |  |  |  |  |  |
|  | L Insular Cortex | 4.23 | -36 | -10 | 14 |
| 232 |  |  |  |  |  |
|  | L Lateral Occipital Cortex, inferior division | 3.66 | -48 | -62 | -8 |
| 192 |  |  |  |  |  |
|  | L Amygdala | 4.82 | -26 | -8 | -12 |
| 176 |  |  |  |  |  |
|  | R Amygdala | 3.26 | 26 | -6 | -12 |
|  | R Putamen | 3.61 | 32 | -10 | -10 |
|  | R Amygdala | 3.44 | 26 | -10 | -10 |
| 168 |  |  |  |  |  |
|  | L Hippocampus | 4.32 | -16 | -16 | -18 |
| 160 |  |  |  |  |  |
|  | L Postcentral Gyrus | 3.53 | -48 | -24 | 38 |
| 152 |  |  |  |  |  |
|  | L Temporal Pole | 3.57 | -58 | 6 | -12 |
| 136 |  |  |  |  |  |
|  | L Parahippocampal Gyrus, posterior division | 3.64 | -24 | -42 | -12 |
|  | L Temporal Occipital Fusiform Cortex | 3.19 | -24 | -48 | -10 |
| 128 |  |  |  |  |  |
|  | L Temporal Fusiform Cortex, posterior division | 3.77 | -38 | -32 | -28 |
| 128 |  |  |  |  |  |
|  | L Frontal Pole | 3.85 | -4 | 68 | 4 |
| 112 |  |  |  |  |  |
|  | L Superior Temporal Gyrus, anterior division | 3.43 | -56 | -8 | -8 |
| 112 |  |  |  |  |  |
|  | R Superior Frontal Gyrus | 3.19 | 22 | 18 | 52 |
|  | R Middle Frontal Gyrus | 3.04 | 28 | 18 | 46 |
| 112 |  |  |  |  |  |
|  | R Lateral Occipital Cortex, superior division | 3.57 | 32 | -64 | 52 |
| 104 |  |  |  |  |  |
|  | L Brain-Stem | 4.42 | -18 | -34 | -42 |
| 104 |  |  |  |  |  |
|  | L Temporal Pole | 3.31 | -36 | 6 | -22 |
| 104 |  |  |  |  |  |
|  | R Angular Gyrus | 3.37 | 52 | -58 | 26 |
| 96 |  |  |  |  |  |
|  | L Middle Temporal Gyrus, posterior division | 2.88 | -66 | -12 | -10 |
| 88 |  |  |  |  |  |
|  | R Frontal Pole | 3.21 | 2 | 56 | -22 |
| 88 |  |  |  |  |  |
|  | L Brain-Stem | 4.12 | -14 | -26 | -16 |
| 88 |  |  |  |  |  |
|  | L Postcentral Gyrus | 3.94 | -6 | -42 | 68 |
| 80 |  |  |  |  |  |
|  | R Brain-Stem | 3.49 | 6 | -34 | -50 |
| 80 |  |  |  |  |  |
|  | L Inferior Temporal Gyrus, anterior division | 3.30 | -42 | -6 | -40 |
| 80 |  |  |  |  |  |
|  | L Temporal Fusiform Cortex, posterior division | 3.82 | -32 | -22 | -32 |
| 72 |  |  |  |  |  |
|  | L Lateral Occipital Cortex, superior division | 3.31 | -38 | -78 | 20 |
| 72 |  |  |  |  |  |
|  | R Frontal Pole | 3.35 | 20 | 48 | 36 |
| 72 |  |  |  |  |  |
|  | R Supramarginal Gyrus, posterior division | 3.13 | 50 | -40 | 50 |
| 64 |  |  |  |  |  |
|  | L Frontal Pole | 3.63 | -4 | 64 | -22 |
| 64 |  |  |  |  |  |
|  | R Cingulate Gyrus, posterior division | 3.69 | 6 | -46 | 4 |
| 64 |  |  |  |  |  |
|  | L Frontal Pole | 3.24 | -16 | 66 | 16 |
| 64 |  |  |  |  |  |
|  | L Occipital Pole | 3.04 | -12 | -94 | 30 |
| 56 |  |  |  |  |  |
|  | L Thalamus | 3.33 | -12 | -36 | 6 |
| 56 |  |  |  |  |  |
|  | R Central Opercular Cortex | 4.41 | 38 | -6 | 18 |
| 56 |  |  |  |  |  |
|  | R Middle Frontal Gyrus | 2.89 | 30 | 8 | 50 |
| 48 |  |  |  |  |  |
|  | R Temporal Pole | 2.79 | 32 | 14 | -30 |
| 48 |  |  |  |  |  |
|  | L Frontal Pole | 3.33 | -24 | 58 | 0 |
| 48 |  |  |  |  |  |
|  | R Occipital Pole | 3.42 | 4 | -98 | 6 |
| 48 |  |  |  |  |  |
|  | L Lateral Occipital Cortex, superior division | 3.57 | -46 | -68 | 26 |
| 48 |  |  |  |  |  |
|  | L Superior Frontal Gyrus | 3.44 | -6 | 46 | 42 |
| 40 |  |  |  |  |  |
|  | R Brain-Stem | 4.27 | 6 | -36 | -64 |
| 40 |  |  |  |  |  |
|  | R Brain-Stem | 3.12 | 10 | -36 | -44 |
| 40 |  |  |  |  |  |
|  | L Brain-Stem | 3.44 | -18 | -26 | -38 |
| 40 |  |  |  |  |  |
|  | L Middle Temporal Gyrus, anterior division | 3.59 | -58 | -2 | -18 |
| 40 |  |  |  |  |  |
|  | R Superior Temporal Gyrus, anterior division | 2.98 | 54 | -2 | -18 |
| 40 |  |  |  |  |  |
|  | L Accumbens | 2.94 | -10 | 8 | -10 |
|  | L Putamen | 3.02 | -14 | 6 | -8 |
| 40 |  |  |  |  |  |
|  | R Lingual Gyrus | 3.56 | 12 | -56 | -4 |
| 40 |  |  |  |  |  |
|  | R Insular Cortex | 2.98 | 38 | -4 | 10 |
| 40 |  |  |  |  |  |
|  | R Occipital Pole | 3.55 | 2 | -94 | 18 |
| 40 |  |  |  |  |  |
|  | L Cingulate Gyrus, posterior division | 3.28 | -4 | -40 | 30 |
| 40 |  |  |  |  |  |
|  | L Cingulate Gyrus, anterior division | 3.32 | -4 | 2 | 30 |
| 40 |  |  |  |  |  |
|  | R Lateral Occipital Cortex, superior division | 3.42 | 48 | -72 | 42 |
| 32 |  |  |  |  |  |
|  | L Inferior Temporal Gyrus, anterior division | 3.11 | -50 | -10 | -36 |
| 32 |  |  |  |  |  |
|  | R Brain-Stem | 3.12 | 20 | -34 | -32 |
| 32 |  |  |  |  |  |
|  | L Parahippocampal Gyrus, anterior division | 3.02 | -18 | 0 | -32 |
| 32 |  |  |  |  |  |
|  | L Amygdala | 2.94 | -14 | -2 | -20 |
| 32 |  |  |  |  |  |
|  | L Superior Temporal Gyrus, anterior division | 3.06 | -50 | -6 | -16 |
| 32 |  |  |  |  |  |
|  | L Amygdala | 3.40 | -26 | 0 | -18 |
| 32 |  |  |  |  |  |
|  | L Frontal Pole | 2.91 | -4 | 62 | -12 |
| 32 |  |  |  |  |  |
|  | L Lingual Gyrus | 2.83 | -28 | -44 | -6 |
| 32 |  |  |  |  |  |
|  | R Lingual Gyrus | 3.89 | 10 | -72 | -2 |
| 32 |  |  |  |  |  |
|  | L Angular Gyrus | 3.13 | -42 | -58 | 30 |
| 32 |  |  |  |  |  |
|  | R Lateral Occipital Cortex, superior division | 2.99 | 54 | -68 | 34 |
| 32 |  |  |  |  |  |
|  | R Cingulate Gyrus, posterior division | 2.98 | 10 | -42 | 38 |
| 32 |  |  |  |  |  |
|  | L Postcentral Gyrus | 2.93 | -58 | -18 | 50 |
| 32 |  |  |  |  |  |
|  | L Superior Frontal Gyrus | 3.63 | -22 | -6 | 56 |
| 24 |  |  |  |  |  |
|  | L Brain-Stem | 2.87 | -6 | -38 | -64 |
| 24 |  |  |  |  |  |
|  | L Temporal Pole | 3.67 | -22 | 6 | -40 |
| 24 |  |  |  |  |  |
|  | L Parahippocampal Gyrus, anterior division | 3.70 | -18 | -8 | -36 |
| 24 |  |  |  |  |  |
|  | L Inferior Temporal Gyrus, posterior division | 3.01 | -50 | -36 | -24 |
| 24 |  |  |  |  |  |
|  | L Middle Temporal Gyrus, anterior division | 2.86 | -56 | -2 | -24 |
| 24 |  |  |  |  |  |
|  | L Brain-Stem | 2.95 | -4 | -36 | -20 |
| 24 |  |  |  |  |  |
|  | L Frontal Pole | 3.11 | -22 | 40 | -22 |
| 24 |  |  |  |  |  |
|  | R Frontal Pole | 2.79 | 12 | 48 | -22 |
| 24 |  |  |  |  |  |
|  | R Middle Temporal Gyrus, posterior division | 3.24 | 54 | -10 | -18 |
| 24 |  |  |  |  |  |
|  | L Occipital Pole | 3.51 | -6 | -100 | 6 |
| 24 |  |  |  |  |  |
|  | L Frontal Pole | 3.11 | -20 | 70 | 8 |
| 24 |  |  |  |  |  |
|  | R Frontal Pole | 2.99 | 38 | 38 | 16 |
| 24 |  |  |  |  |  |
|  | L Occipital Pole | 3.11 | -6 | -90 | 18 |
| 24 |  |  |  |  |  |
|  | L Cuneal Cortex | 3.20 | -8 | -84 | 34 |
| 24 |  |  |  |  |  |
|  | L Precuneus Cortex | 3.61 | -6 | -74 | 42 |
| 24 |  |  |  |  |  |
|  | R Superior Frontal Gyrus | 3.31 | 20 | 24 | 42 |
| 24 |  |  |  |  |  |
|  | L Frontal Pole | 3.17 | -14 | 42 | 44 |
| 16 |  |  |  |  |  |
|  | L Temporal Fusiform Cortex, anterior division | 3.28 | -38 | -2 | -48 |
| 16 |  |  |  |  |  |
|  | R Temporal Fusiform Cortex, anterior division | 2.78 | 34 | -8 | -46 |
| 16 |  |  |  |  |  |
|  | L Temporal Fusiform Cortex, anterior division | 2.72 | -28 | -8 | -42 |
| 16 |  |  |  |  |  |
|  | L Brain-Stem | 2.91 | -6 | -24 | -40 |
| 16 |  |  |  |  |  |
|  | L Parahippocampal Gyrus, anterior division | 2.81 | -24 | 0 | -40 |
| 16 |  |  |  |  |  |
|  | L Temporal Pole | 3.09 | -42 | 2 | -40 |
| 16 |  |  |  |  |  |
|  | L Temporal Pole | 2.94 | -38 | 14 | -40 |
| 16 |  |  |  |  |  |
|  | L Temporal Pole | 3.28 | -36 | 10 | -36 |
| 16 |  |  |  |  |  |
|  | L Temporal Fusiform Cortex, posterior division | 2.87 | -38 | -14 | -26 |
| 16 |  |  |  |  |  |
|  | R Subcallosal Cortex | 2.81 | 8 | 22 | -24 |
| 16 |  |  |  |  |  |
|  | L Temporal Fusiform Cortex, posterior division | 2.99 | -40 | -18 | -22 |
| 16 |  |  |  |  |  |
|  | L Inferior Temporal Gyrus, temporooccipital part | 3.00 | -48 | -56 | -20 |
| 16 |  |  |  |  |  |
|  | L Amygdala | 3.94 | -32 | -4 | -20 |
| 16 |  |  |  |  |  |
|  | R Temporal Pole | 3.43 | 30 | 6 | -20 |
| 16 |  |  |  |  |  |
|  | L Frontal Medial Cortex | 3.79 | -4 | 52 | -20 |
| 16 |  |  |  |  |  |
|  | L Subcallosal Cortex | 3.51 | -4 | 18 | -16 |
| 16 |  |  |  |  |  |
|  | L Occipital Fusiform Gyrus | 2.97 | -28 | -80 | -14 |
| 16 |  |  |  |  |  |
|  | L Inferior Temporal Gyrus, temporooccipital part | 3.01 | -46 | -58 | -14 |
| 16 |  |  |  |  |  |
|  | R Hippocampus | 2.94 | 30 | -18 | -12 |
| 16 |  |  |  |  |  |
|  | L Hippocampus | 3.54 | -28 | -18 | -12 |
| 16 |  |  |  |  |  |
|  | R Occipital Pole | 2.89 | 4 | -98 | -2 |
| 16 |  |  |  |  |  |
|  | R Occipital Fusiform Gyrus | 3.32 | 28 | -72 | -4 |
| 16 |  |  |  |  |  |
|  | L Frontal Pole | 2.91 | -34 | 60 | 2 |
| 16 |  |  |  |  |  |
|  | R Frontal Pole | 3.12 | 42 | 48 | 2 |
| 16 |  |  |  |  |  |
|  | L Central Opercular Cortex | 3.32 | -44 | -10 | 16 |
| 16 |  |  |  |  |  |
|  | L Cingulate Gyrus, posterior division | 3.06 | -6 | -46 | 20 |
| 16 |  |  |  |  |  |
|  | L Angular Gyrus | 3.40 | -50 | -52 | 24 |
| 16 |  |  |  |  |  |
|  | L Precentral Gyrus | 2.93 | -52 | -4 | 24 |
| 16 |  |  |  |  |  |
|  | L Middle Frontal Gyrus | 3.14 | -38 | 28 | 24 |
| 16 |  |  |  |  |  |
|  | R Cuneal Cortex | 2.86 | 16 | -72 | 28 |
| 16 |  |  |  |  |  |
|  | L Postcentral Gyrus | 3.05 | -58 | -18 | 36 |
| 16 |  |  |  |  |  |
|  | R Frontal Pole | 2.84 | 16 | 54 | 38 |
| 16 |  |  |  |  |  |
|  | R Superior Parietal Lobule | 2.84 | 38 | -48 | 46 |
| 16 |  |  |  |  |  |
|  | R Lateral Occipital Cortex, superior division | 2.74 | 38 | -64 | 54 |
| 16 |  |  |  |  |  |
|  | R Lateral Occipital Cortex, superior division | 2.80 | 18 | -68 | 58 |
| 16 |  |  |  |  |  |
|  | L Middle Frontal Gyrus | 2.99 | -30 | 20 | 56 |
| 8 |  |  |  |  |  |
|  | R Brain-Stem | 2.83 | 10 | -38 | -58 |
| 8 |  |  |  |  |  |
|  | L Brain-Stem | 2.75 | -2 | -30 | -54 |
| 8 |  |  |  |  |  |
|  | R Temporal Pole | 2.91 | 30 | 4 | -48 |
| 8 |  |  |  |  |  |
|  | R Temporal Pole | 2.87 | 44 | 14 | -36 |
| 8 |  |  |  |  |  |
|  | R Temporal Pole | 2.78 | 38 | 18 | -36 |
| 8 |  |  |  |  |  |
|  | R Brain-Stem | 2.71 | 8 | -40 | -28 |
| 8 |  |  |  |  |  |
|  | R Frontal Medial Cortex | 2.77 | 2 | 32 | -24 |
| 8 |  |  |  |  |  |
|  | L Brain-Stem | 2.93 | -4 | -14 | -22 |
| 8 |  |  |  |  |  |
|  | L Temporal Pole | 3.05 | -44 | 12 | -22 |
| 8 |  |  |  |  |  |
|  | L Frontal Pole | 2.74 | -10 | 64 | -20 |
| 8 |  |  |  |  |  |
|  | L Hippocampus | 2.92 | -12 | -12 | -18 |
| 8 |  |  |  |  |  |
|  | L Frontal Pole | 2.70 | -14 | 66 | -18 |
| 8 |  |  |  |  |  |
|  | L Parahippocampal Gyrus, posterior division | 2.95 | -20 | -30 | -16 |
| 8 |  |  |  |  |  |
|  | L Frontal Pole | 2.71 | -6 | 56 | -16 |
| 8 |  |  |  |  |  |
|  | L Lateral Occipital Cortex, inferior division | 2.76 | -56 | -64 | -14 |
| 8 |  |  |  |  |  |
|  | L Brain-Stem | 2.78 | -2 | -28 | -14 |
| 8 |  |  |  |  |  |
|  | L Frontal Pole | 2.73 | -20 | 50 | -14 |
| 8 |  |  |  |  |  |
|  | R Frontal Pole | 3.20 | 22 | 66 | -14 |
| 8 |  |  |  |  |  |
|  | L Frontal Pole | 2.85 | -18 | 68 | -12 |
| 8 |  |  |  |  |  |
|  | R Brain-Stem | 2.68 | 10 | -28 | -8 |
| 8 |  |  |  |  |  |
|  | R Frontal Pole | 3.27 | 32 | 36 | -8 |
| 8 |  |  |  |  |  |
|  | L Frontal Pole | 2.68 | -10 | 64 | -8 |
| 8 |  |  |  |  |  |
|  | L Lingual Gyrus | 3.01 | -22 | -60 | -6 |
| 8 |  |  |  |  |  |
|  | L Inferior Temporal Gyrus, temporooccipital part | 2.74 | -42 | -54 | -6 |
| 8 |  |  |  |  |  |
|  | R Subcallosal Cortex | 3.49 | 4 | 22 | -6 |
| 8 |  |  |  |  |  |
|  | L Frontal Pole | 2.69 | -40 | 46 | -6 |
| 8 |  |  |  |  |  |
|  | L Middle Temporal Gyrus, temporooccipital part | 2.71 | -68 | -48 | -4 |
| 8 |  |  |  |  |  |
|  | R Pallidum | 3.45 | 20 | -8 | -4 |
| 8 |  |  |  |  |  |
|  | L Frontal Pole | 2.95 | -20 | 66 | 4 |
| 8 |  |  |  |  |  |
|  | R Postcentral Gyrus | 2.77 | 66 | -8 | 14 |
| 8 |  |  |  |  |  |
|  | L Frontal Pole | 2.69 | -8 | 60 | 18 |
| 8 |  |  |  |  |  |
|  | R Lateral Occipital Cortex, superior division | 2.72 | 46 | -62 | 24 |
| 8 |  |  |  |  |  |
|  | L Angular Gyrus | 2.72 | -40 | -56 | 24 |
| 8 |  |  |  |  |  |
|  | L Cingulate Gyrus, posterior division | 2.71 | -14 | -46 | 32 |
| 8 |  |  |  |  |  |
|  | R Cingulate Gyrus, anterior division | 2.81 | 6 | -4 | 32 |
| 8 |  |  |  |  |  |
|  | R Cingulate Gyrus, anterior division | 2.71 | 4 | 0 | 32 |
| 8 |  |  |  |  |  |
|  | L Lateral Occipital Cortex, superior division | 2.70 | -44 | -62 | 36 |
| 8 |  |  |  |  |  |
|  | L Middle Frontal Gyrus | 2.80 | -50 | 26 | 36 |
| 8 |  |  |  |  |  |
|  | R Frontal Pole | 2.69 | 14 | 52 | 36 |
| 8 |  |  |  |  |  |
|  | L Frontal Pole | 2.73 | -18 | 40 | 38 |
| 8 |  |  |  |  |  |
|  | L Cuneal Cortex | 2.80 | -6 | -86 | 40 |
| 8 |  |  |  |  |  |
|  | L Superior Frontal Gyrus | 2.84 | -20 | 26 | 40 |
| 8 |  |  |  |  |  |
|  | R Lateral Occipital Cortex, superior division | 2.80 | 38 | -78 | 42 |
| 8 |  |  |  |  |  |
|  | L Lateral Occipital Cortex, superior division | 2.69 | -34 | -74 | 42 |
| 8 |  |  |  |  |  |
|  | L Frontal Pole | 2.83 | -24 | 40 | 42 |
| 8 |  |  |  |  |  |
|  | L Precuneus Cortex | 2.70 | -8 | -70 | 44 |
| 8 |  |  |  |  |  |
|  | R Lateral Occipital Cortex, superior division | 2.81 | 38 | -70 | 46 |
| 8 |  |  |  |  |  |
|  | L Precuneus Cortex | 2.91 | -6 | -56 | 46 |
| 8 |  |  |  |  |  |
|  | L Paracingulate Gyrus | 2.99 | -8 | 16 | 46 |
| 8 |  |  |  |  |  |
|  | L Lateral Occipital Cortex, superior division | 2.78 | -18 | -82 | 48 |
| 8 |  |  |  |  |  |
|  | R Lateral Occipital Cortex, superior division | 2.86 | 38 | -74 | 50 |
| 8 |  |  |  |  |  |
|  | L Superior Parietal Lobule | 2.71 | -28 | -50 | 54 |
| 8 |  |  |  |  |  |
|  | R Superior Frontal Gyrus | 3.37 | 16 | 28 | 54 |
| 8 |  |  |  |  |  |
|  | L Lateral Occipital Cortex, superior division | 2.86 | -14 | -68 | 62 |
| 8 |  |  |  |  |  |
|  | R Postcentral Gyrus | 2.69 | 14 | -36 | 72 |
| 8 |  |  |  |  |  |
|  | R Postcentral Gyrus | 2.74 | 8 | -38 | 74 |
| 8 |  |  |  |  |  |
|  | R Postcentral Gyrus | 2.91 | 10 | -34 | 74 |
| 8 |  |  |  |  |  |
|  | R Precentral Gyrus | 2.78 | 6 | -28 | 78 |

# Supplementary Table S30. [UT_CB3 _- UT_CB2_] > [CT_CB3 _- CT_CB2_]. Descriptive statistics for clusters and local maxima showing greater increases in activation from Convolved Block to Convolved Block of Uncertain-Threat anticipation relative to increases in activation from Convolved Block 2 to Convolved Block 3 of Certain-Threat anticipation (FDR *q*<0.05, whole-brain corrected). 

| **mm**^**3**^ | **Label** | **t** | **x** | **y** | **z** |
| --- | --- | --- | --- | --- | --- |
| 57,120 |  |  |  |  |  |
|  | R Hippocampus | 4.13 | 30 | -22 | -16 |
|  | R Parahippocampal Gyrus, posterior division | 5.53 | 26 | -28 | -22 |
|  | L Parahippocampal Gyrus, posterior division | 4.89 | -26 | -32 | -20 |
|  | R Temporal Fusiform Cortex, posterior division | 4.76 | 26 | -34 | -20 |
|  | R Thalamus | 4.07 | 8 | -34 | 6 |
|  | L Temporal Fusiform Cortex, posterior division | 3.11 | -32 | -36 | -22 |
|  | L Cingulate Gyrus, posterior division | 4.25 | -6 | -46 | 4 |
|  | R Cingulate Gyrus, posterior division | 4.45 | 8 | -46 | 4 |
|  | L Temporal Occipital Fusiform Cortex | 5.79 | -30 | -48 | -8 |
|  | L Inferior Temporal Gyrus, temporooccipital part | 4.42 | -42 | -48 | -12 |
|  | R Lingual Gyrus | 7.75 | 22 | -50 | 0 |
|  | R Temporal Occipital Fusiform Cortex | 4.24 | 24 | -52 | -12 |
|  | L Precuneus Cortex | 4.77 | -2 | -54 | 10 |
|  | L Lingual Gyrus | 7.06 | -22 | -56 | 2 |
|  | R Inferior Temporal Gyrus, temporooccipital part | 3.79 | 46 | -56 | -14 |
|  | R Precuneus Cortex | 5.22 | 20 | -58 | 14 |
|  | R Occipital Fusiform Gyrus | 5.19 | 38 | -64 | -12 |
|  | R Lateral Occipital Cortex, superior division | 5.89 | 32 | -72 | 32 |
|  | L Occipital Fusiform Gyrus | 5.11 | -36 | -74 | -14 |
|  | L Lateral Occipital Cortex, superior division | 5.56 | -26 | -76 | 30 |
|  | R Lateral Occipital Cortex, inferior division | 5.79 | 48 | -78 | 8 |
|  | R Intracalcarine Cortex | 5.04 | 14 | -78 | 8 |
|  | R Cuneal Cortex | 6.57 | 6 | -82 | 40 |
|  | L Cuneal Cortex | 5.79 | -4 | -86 | 36 |
|  | L Intracalcarine Cortex | 5.07 | -8 | -86 | 0 |
|  | R Supracalcarine Cortex | 5.43 | 2 | -86 | 12 |
|  | L Lateral Occipital Cortex, inferior division | 7.31 | -38 | -88 | -10 |
|  | R Occipital Pole | 6.01 | 8 | -88 | 34 |
|  | L Occipital Pole | 6.68 | -6 | -94 | 30 |
| 5,936 |  |  |  |  |  |
|  | R Temporal Pole | 6.00 | 46 | 20 | -20 |
|  | R Planum Polare | 5.43 | 50 | 4 | -4 |
|  | R Superior Temporal Gyrus, anterior division | 4.65 | 58 | 4 | -8 |
|  | R Middle Temporal Gyrus, anterior division | 3.61 | 60 | -2 | -16 |
|  | R Insular Cortex | 3.06 | 44 | -4 | -8 |
|  | R Planum Temporale | 4.19 | 66 | -10 | 6 |
|  | R Central Opercular Cortex | 3.64 | 64 | -12 | 12 |
|  | R Middle Temporal Gyrus, posterior division | 3.53 | 68 | -14 | -12 |
|  | R Superior Temporal Gyrus, posterior division | 3.71 | 66 | -18 | 0 |
| 2,216 |  |  |  |  |  |
|  | L Frontal Orbital Cortex | 4.45 | -40 | 22 | -16 |
|  | L Temporal Pole | 5.95 | -36 | 18 | -24 |
| 2,088 |  |  |  |  |  |
|  | R Inferior Frontal Gyrus, pars triangularis | 3.88 | 58 | 28 | 10 |
|  | R Inferior Frontal Gyrus, pars opercularis | 5.52 | 52 | 12 | 22 |
|  | R Middle Frontal Gyrus | 3.04 | 48 | 12 | 42 |
|  | R Precentral Gyrus | 3.78 | 44 | 8 | 34 |
| 2,056 |  |  |  |  |  |
|  | L Superior Temporal Gyrus, anterior division | 3.16 | -60 | 0 | -6 |
|  | L Planum Polare | 4.67 | -52 | -8 | 2 |
|  | L Superior Temporal Gyrus, posterior division | 4.46 | -66 | -14 | 0 |
|  | L Heschls Gyrus (includes H1 and H2) | 4.07 | -52 | -14 | 8 |
|  | L Central Opercular Cortex | 4.45 | -62 | -20 | 12 |
| 1,800 |  |  |  |  |  |
|  | R Frontal Pole | 5.23 | 12 | 62 | 28 |
|  | R Superior Frontal Gyrus | 3.64 | 6 | 54 | 20 |
|  | L Paracingulate Gyrus | 2.99 | -4 | 52 | 8 |
|  | R Paracingulate Gyrus | 3.86 | 10 | 48 | 10 |
|  | R Cingulate Gyrus, anterior division | 3.89 | 4 | 38 | 8 |
| 1,592 |  |  |  |  |  |
|  | R Postcentral Gyrus | 5.13 | 66 | -14 | 34 |
|  | R Supramarginal Gyrus, anterior division | 4.28 | 56 | -22 | 40 |
| 1,440 |  |  |  |  |  |
|  | R Frontal Pole | 4.27 | 48 | 38 | -12 |
|  | R Frontal Orbital Cortex | 4.82 | 48 | 28 | -8 |
| 1,432 |  |  |  |  |  |
|  | R Frontal Pole | 4.12 | 50 | 36 | 18 |
|  | R Middle Frontal Gyrus | 3.24 | 50 | 30 | 24 |
| 1,080 |  |  |  |  |  |
|  | L Inferior Frontal Gyrus, pars triangularis | 3.88 | -52 | 36 | 6 |
|  | L Frontal Orbital Cortex | 4.64 | -48 | 34 | -8 |
|  | L Frontal Operculum Cortex | 4.01 | -46 | 24 | 0 |
| 1,024 |  |  |  |  |  |
|  | L Precentral Gyrus | 2.88 | -42 | -16 | 66 |
|  | L Postcentral Gyrus | 4.12 | -46 | -20 | 60 |
| 616 |  |  |  |  |  |
|  | L Frontal Pole | 4.60 | -8 | 66 | 24 |
|  | L Superior Frontal Gyrus | 2.90 | -2 | 56 | 26 |
| 504 |  |  |  |  |  |
|  | R Superior Frontal Gyrus | 4.00 | 20 | 28 | 48 |
|  | R Middle Frontal Gyrus | 3.88 | 26 | 26 | 48 |
| 432 |  |  |  |  |  |
|  | L Paracingulate Gyrus | 4.20 | -12 | 46 | -4 |
|  | L Frontal Medial Cortex | 3.84 | -8 | 46 | -10 |
| 432 |  |  |  |  |  |
|  | L Postcentral Gyrus | 3.57 | -12 | -42 | 54 |
|  | L Precuneus Cortex | 4.50 | -6 | -46 | 64 |
| 400 |  |  |  |  |  |
|  | R Frontal Pole | 4.05 | 10 | 46 | 50 |
| 368 |  |  |  |  |  |
|  | L Superior Temporal Gyrus, posterior division | 3.67 | -52 | -36 | 2 |
| 344 |  |  |  |  |  |
|  | L Parahippocampal Gyrus, anterior division | 3.26 | -18 | -22 | -20 |
|  | L Hippocampus | 4.61 | -20 | -28 | -12 |
|  | L Parahippocampal Gyrus, posterior division | 3.49 | -16 | -30 | -14 |
| 256 |  |  |  |  |  |
|  | L Temporal Fusiform Cortex, posterior division | 3.90 | -34 | -44 | -20 |
|  | L Temporal Occipital Fusiform Cortex | 3.39 | -42 | -48 | -18 |
| 256 |  |  |  |  |  |
|  | L Frontal Pole | 3.79 | -4 | 58 | 34 |
| 256 |  |  |  |  |  |
|  | R Postcentral Gyrus | 3.54 | 20 | -30 | 78 |
| 232 |  |  |  |  |  |
|  | L Frontal Pole | 3.33 | -46 | 44 | -12 |
| 216 |  |  |  |  |  |
|  | L Superior Frontal Gyrus | 4.62 | -4 | 16 | 62 |
| 192 |  |  |  |  |  |
|  | L Temporal Pole | 4.21 | -48 | 10 | -10 |
| 184 |  |  |  |  |  |
|  | R Middle Temporal Gyrus, temporooccipital part | 4.47 | 56 | -48 | -8 |
| 184 |  |  |  |  |  |
|  | L Postcentral Gyrus | 3.09 | -18 | -30 | 78 |
| 176 |  |  |  |  |  |
|  | R Temporal Occipital Fusiform Cortex | 3.54 | 38 | -44 | -20 |
| 160 |  |  |  |  |  |
|  | L Superior Parietal Lobule | 3.20 | -26 | -56 | 64 |
| 144 |  |  |  |  |  |
|  | R Middle Temporal Gyrus, temporooccipital part | 4.17 | 58 | -58 | 12 |
| 144 |  |  |  |  |  |
|  | R Lateral Occipital Cortex, superior division | 3.66 | 18 | -58 | 60 |
| 136 |  |  |  |  |  |
|  | R Frontal Pole | 3.78 | 10 | 68 | 18 |
| 128 |  |  |  |  |  |
|  | R Middle Temporal Gyrus, posterior division | 4.09 | 48 | -36 | -2 |
| 112 |  |  |  |  |  |
|  | L Paracingulate Gyrus | 3.38 | -6 | 48 | 14 |
| 104 |  |  |  |  |  |
|  | L Occipital Fusiform Gyrus | 4.14 | -28 | -74 | -14 |
| 104 |  |  |  |  |  |
|  | R Cuneal Cortex | 3.58 | 14 | -76 | 30 |
| 96 |  |  |  |  |  |
|  | R Parahippocampal Gyrus, anterior division | 3.55 | 18 | -8 | -28 |
| 96 |  |  |  |  |  |
|  | L Frontal Pole | 3.73 | -12 | 68 | 12 |
| 96 |  |  |  |  |  |
|  | R Precuneus Cortex | 3.35 | 4 | -62 | 20 |
| 88 |  |  |  |  |  |
|  | L Temporal Pole | 3.29 | -54 | 6 | -20 |
| 80 |  |  |  |  |  |
|  | R Temporal Pole | 3.27 | 44 | 12 | -28 |
| 80 |  |  |  |  |  |
|  | L Temporal Fusiform Cortex, posterior division | 3.74 | -42 | -38 | -18 |
| 80 |  |  |  |  |  |
|  | L Superior Temporal Gyrus, posterior division | 3.49 | -62 | -30 | 4 |
| 72 |  |  |  |  |  |
|  | R Frontal Pole | 3.36 | 32 | 40 | -12 |
| 71 |  |  |  |  |  |
|  | L Middle Frontal Gyrus | 3.46 | -40 | 12 | 46 |
| 56 |  |  |  |  |  |
|  | L Temporal Pole | 3.01 | -26 | 4 | -28 |
|  | L Amygdala | 3.28 | -24 | 2 | -26 |
|  | L Parahippocampal Gyrus, anterior division | 3.21 | -24 | 2 | -30 |
| 56 |  |  |  |  |  |
|  | L Superior Temporal Gyrus, anterior division | 3.02 | -52 | -2 | -16 |
|  | L Middle Temporal Gyrus, anterior division | 3.10 | -54 | -4 | -18 |
| 56 |  |  |  |  |  |
|  | L Superior Temporal Gyrus, anterior division | 3.91 | -58 | 0 | -14 |
| 56 |  |  |  |  |  |
|  | R Middle Temporal Gyrus, temporooccipital part | 3.08 | 68 | -40 | -10 |
| 56 |  |  |  |  |  |
|  | L Lateral Occipital Cortex, inferior division | 3.91 | -56 | -66 | -6 |
| 56 |  |  |  |  |  |
|  | R Central Opercular Cortex | 3.24 | 40 | -6 | 12 |
| 48 |  |  |  |  |  |
|  | L Planum Polare | 2.91 | -48 | 0 | -6 |
| 48 |  |  |  |  |  |
|  | R Middle Frontal Gyrus | 2.86 | 42 | 12 | 44 |
| 48 |  |  |  |  |  |
|  | R Postcentral Gyrus | 3.44 | 8 | -44 | 68 |
| 48 |  |  |  |  |  |
|  | R Precentral Gyrus | 3.93 | 6 | -24 | 80 |
| 40 |  |  |  |  |  |
|  | L Temporal Pole | 3.30 | -30 | 4 | -36 |
|  | L Parahippocampal Gyrus, anterior division | 3.17 | -30 | 0 | -34 |
| 40 |  |  |  |  |  |
|  | L Temporal Fusiform Cortex, posterior division | 3.91 | -40 | -14 | -28 |
| 40 |  |  |  |  |  |
|  | L Parahippocampal Gyrus, anterior division | 3.45 | -20 | -6 | -30 |
| 40 |  |  |  |  |  |
|  | R Subcallosal Cortex | 3.66 | 4 | 30 | -20 |
| 40 |  |  |  |  |  |
|  | L Inferior Temporal Gyrus, temporooccipital part | 3.17 | -50 | -56 | -14 |
| 40 |  |  |  |  |  |
|  | R Inferior Frontal Gyrus, pars triangularis | 2.94 | 52 | 28 | 4 |
| 40 |  |  |  |  |  |
|  | R Cingulate Gyrus, anterior division | 2.89 | 0 | 34 | 14 |
| 40 |  |  |  |  |  |
|  | L Inferior Frontal Gyrus, pars opercularis | 3.39 | -46 | 8 | 28 |
| 40 |  |  |  |  |  |
|  | R Superior Frontal Gyrus | 3.60 | 0 | 42 | 42 |
| 40 |  |  |  |  |  |
|  | L Superior Frontal Gyrus | 3.14 | -22 | 28 | 52 |
| 40 |  |  |  |  |  |
|  | R Superior Parietal Lobule | 3.14 | 26 | -56 | 60 |
| 32 |  |  |  |  |  |
|  | R Temporal Pole | 3.12 | 28 | 14 | -42 |
| 32 |  |  |  |  |  |
|  | R Amygdala | 3.04 | 24 | 4 | -20 |
| 32 |  |  |  |  |  |
|  | R Planum Polare | 3.76 | 42 | -2 | -16 |
| 32 |  |  |  |  |  |
|  | R Insular Cortex | 3.72 | 38 | 6 | -16 |
| 32 |  |  |  |  |  |
|  | L Insular Cortex | 3.89 | -40 | 2 | -12 |
| 32 |  |  |  |  |  |
|  | L Subcallosal Cortex | 2.99 | -2 | 16 | -6 |
| 32 |  |  |  |  |  |
|  | R Frontal Pole | 3.33 | 30 | 62 | -6 |
| 32 |  |  |  |  |  |
|  | L Superior Temporal Gyrus, posterior division | 3.23 | -68 | -24 | 2 |
| 32 |  |  |  |  |  |
|  | L Thalamus | 3.46 | -6 | -34 | 2 |
| 32 |  |  |  |  |  |
|  | R Central Opercular Cortex | 2.86 | 56 | -12 | 14 |
| 32 |  |  |  |  |  |
|  | R Parietal Operculum Cortex | 3.65 | 36 | -28 | 20 |
| 32 |  |  |  |  |  |
|  | R Superior Frontal Gyrus | 3.00 | 2 | 44 | 48 |
| 32 |  |  |  |  |  |
|  | L Lateral Occipital Cortex, superior division | 2.89 | -16 | -64 | 58 |
| 32 |  |  |  |  |  |
|  | R Superior Frontal Gyrus | 3.06 | 26 | 14 | 58 |
| 24 |  |  |  |  |  |
|  | R Brain-Stem | 3.22 | 2 | -36 | -66 |
| 24 |  |  |  |  |  |
|  | R Brain-Stem | 3.31 | 8 | -30 | -34 |
| 24 |  |  |  |  |  |
|  | L Parahippocampal Gyrus, anterior division | 3.59 | -18 | -12 | -28 |
| 24 |  |  |  |  |  |
|  | L Frontal Orbital Cortex | 3.14 | -24 | 14 | -24 |
| 24 |  |  |  |  |  |
|  | L Temporal Pole | 3.25 | -52 | 12 | -20 |
| 24 |  |  |  |  |  |
|  | R Frontal Orbital Cortex | 3.46 | 14 | 16 | -22 |
| 24 |  |  |  |  |  |
|  | L Temporal Occipital Fusiform Cortex | 3.10 | -34 | -60 | -16 |
| 24 |  |  |  |  |  |
|  | R Subcallosal Cortex | 3.09 | 2 | 12 | -10 |
| 24 |  |  |  |  |  |
|  | R Subcallosal Cortex | 3.07 | 4 | 30 | -8 |
| 24 |  |  |  |  |  |
|  | R Paracingulate Gyrus | 3.25 | 10 | 46 | -6 |
| 24 |  |  |  |  |  |
|  | R Superior Temporal Gyrus, posterior division | 3.02 | 60 | -28 | 2 |
| 24 |  |  |  |  |  |
|  | R Paracingulate Gyrus | 3.40 | 10 | 44 | 0 |
| 24 |  |  |  |  |  |
|  | L Paracingulate Gyrus | 3.38 | -4 | 44 | 2 |
| 24 |  |  |  |  |  |
|  | R Central Opercular Cortex | 2.93 | 60 | -8 | 10 |
| 24 |  |  |  |  |  |
|  | R Lateral Occipital Cortex, superior division | 3.31 | 36 | -82 | 12 |
| 24 |  |  |  |  |  |
|  | R Lateral Occipital Cortex, superior division | 3.01 | 28 | -80 | 18 |
| 24 |  |  |  |  |  |
|  | L Frontal Pole | 2.92 | -6 | 48 | 50 |
| 16 |  |  |  |  |  |
|  | L Brain-Stem | 2.84 | -2 | -32 | -60 |
| 16 |  |  |  |  |  |
|  | L Parahippocampal Gyrus, anterior division | 3.44 | -22 | -14 | -34 |
| 16 |  |  |  |  |  |
|  | R Brain-Stem | 2.87 | 16 | -32 | -28 |
| 16 |  |  |  |  |  |
|  | L Amygdala | 3.59 | -28 | 0 | -28 |
| 16 |  |  |  |  |  |
|  | L Temporal Fusiform Cortex, posterior division | 3.24 | -34 | -24 | -24 |
| 16 |  |  |  |  |  |
|  | R Inferior Temporal Gyrus, posterior division | 3.01 | 44 | -24 | -22 |
| 16 |  |  |  |  |  |
|  | R Frontal Medial Cortex | 3.09 | 8 | 48 | -20 |
| 16 |  |  |  |  |  |
|  | R Frontal Orbital Cortex | 2.89 | 22 | 16 | -18 |
| 16 |  |  |  |  |  |
|  | R Frontal Pole | 3.03 | 6 | 56 | -10 |
| 16 |  |  |  |  |  |
|  | L Frontal Pole | 2.89 | -40 | 58 | -10 |
| 16 |  |  |  |  |  |
|  | R Hippocampus | 2.89 | 24 | -28 | -8 |
| 16 |  |  |  |  |  |
|  | L Frontal Pole | 2.89 | -54 | 40 | -6 |
| 16 |  |  |  |  |  |
|  | R Frontal Pole | 3.49 | 4 | 62 | -4 |
| 16 |  |  |  |  |  |
|  | R Lateral Occipital Cortex, inferior division | 3.10 | 56 | -62 | -4 |
| 16 |  |  |  |  |  |
|  | L Middle Temporal Gyrus, posterior division | 3.07 | -62 | -44 | -2 |
| 16 |  |  |  |  |  |
|  | R Insular Cortex | 2.90 | 42 | -2 | 2 |
| 16 |  |  |  |  |  |
|  | R Frontal Pole | 3.19 | 42 | 38 | 6 |
| 16 |  |  |  |  |  |
|  | L Frontal Pole | 3.11 | -46 | 44 | 8 |
| 16 |  |  |  |  |  |
|  | L Inferior Frontal Gyrus, pars opercularis | 2.87 | -56 | 22 | 16 |
| 16 |  |  |  |  |  |
|  | R Cingulate Gyrus, anterior division | 3.30 | 2 | 26 | 16 |
| 16 |  |  |  |  |  |
|  | L Angular Gyrus | 3.05 | -60 | -56 | 22 |
| 16 |  |  |  |  |  |
|  | R Middle Frontal Gyrus | 3.46 | 44 | 28 | 22 |
| 16 |  |  |  |  |  |
|  | L Lateral Occipital Cortex, superior division | 2.92 | -50 | -70 | 24 |
| 16 |  |  |  |  |  |
|  | L Frontal Pole | 2.87 | -10 | 60 | 36 |
| 16 |  |  |  |  |  |
|  | L Postcentral Gyrus | 3.39 | -60 | -26 | 42 |
| 16 |  |  |  |  |  |
|  | R Middle Frontal Gyrus | 3.17 | 36 | 16 | 56 |
| 16 |  |  |  |  |  |
|  | L Superior Frontal Gyrus | 3.42 | -18 | 22 | 56 |
| 16 |  |  |  |  |  |
|  | R Postcentral Gyrus | 3.11 | 10 | -44 | 60 |
| 16 |  |  |  |  |  |
|  | R Superior Frontal Gyrus | 3.00 | 20 | 22 | 60 |
| 16 |  |  |  |  |  |
|  | R Superior Parietal Lobule | 3.31 | 24 | -52 | 68 |
| 8 |  |  |  |  |  |
|  | L Brain-Stem | 3.06 | -8 | -36 | -60 |
| 8 |  |  |  |  |  |
|  | L Temporal Pole | 3.01 | -24 | 10 | -32 |
| 8 |  |  |  |  |  |
|  | L Brain-Stem | 2.91 | -14 | -22 | -24 |
| 8 |  |  |  |  |  |
|  | L Temporal Pole | 3.20 | -48 | 4 | -24 |
| 8 |  |  |  |  |  |
|  | L Hippocampus | 2.84 | -16 | -8 | -22 |
| 8 |  |  |  |  |  |
|  | L Frontal Medial Cortex | 3.06 | -8 | 38 | -22 |
| 8 |  |  |  |  |  |
|  | L Frontal Medial Cortex | 3.09 | -2 | 42 | -22 |
| 8 |  |  |  |  |  |
|  | R Temporal Pole | 3.32 | 42 | 6 | -20 |
| 8 |  |  |  |  |  |
|  | L Frontal Orbital Cortex | 3.07 | -24 | 8 | -20 |
| 8 |  |  |  |  |  |
|  | R Frontal Orbital Cortex | 2.84 | 32 | 26 | -20 |
| 8 |  |  |  |  |  |
|  | L Occipital Fusiform Gyrus | 2.75 | -20 | -82 | -18 |
| 8 |  |  |  |  |  |
|  | L Temporal Occipital Fusiform Cortex | 2.82 | -34 | -54 | -18 |
| 8 |  |  |  |  |  |
|  | R Temporal Fusiform Cortex, posterior division | 2.92 | 40 | -26 | -18 |
| 8 |  |  |  |  |  |
|  | R Frontal Medial Cortex | 2.83 | 0 | 40 | -14 |
| 8 |  |  |  |  |  |
|  | L Frontal Pole | 2.96 | -12 | 60 | -14 |
| 8 |  |  |  |  |  |
|  | L Subcallosal Cortex | 2.82 | -2 | 28 | -12 |
| 8 |  |  |  |  |  |
|  | L Occipital Fusiform Gyrus | 2.88 | -30 | -80 | -10 |
| 8 |  |  |  |  |  |
|  | L Inferior Temporal Gyrus, temporooccipital part | 2.84 | -56 | -58 | -10 |
| 8 |  |  |  |  |  |
|  | R Subcallosal Cortex | 3.16 | 6 | 20 | -10 |
| 8 |  |  |  |  |  |
|  | R Frontal Medial Cortex | 2.81 | 8 | 52 | -10 |
| 8 |  |  |  |  |  |
|  | L Middle Temporal Gyrus, temporooccipital part | 2.85 | -58 | -54 | -8 |
| 8 |  |  |  |  |  |
|  | R Frontal Medial Cortex | 2.94 | 10 | 54 | -8 |
| 8 |  |  |  |  |  |
|  | L Frontal Pole | 3.13 | -44 | 56 | -8 |
| 8 |  |  |  |  |  |
|  | R Middle Temporal Gyrus, temporooccipital part | 2.83 | 70 | -42 | -4 |
| 8 |  |  |  |  |  |
|  | R Inferior Frontal Gyrus, pars triangularis | 3.09 | 52 | 30 | -4 |
| 8 |  |  |  |  |  |
|  | L Cingulate Gyrus, anterior division | 2.84 | -2 | 36 | -4 |
| 8 |  |  |  |  |  |
|  | R Frontal Pole | 3.00 | 8 | 58 | -4 |
| 8 |  |  |  |  |  |
|  | R Frontal Pole | 2.99 | 36 | 60 | -4 |
| 8 |  |  |  |  |  |
|  | R Thalamus | 3.14 | 14 | -34 | -2 |
| 8 |  |  |  |  |  |
|  | R Lingual Gyrus | 2.82 | 4 | -64 | 0 |
| 8 |  |  |  |  |  |
|  | L Middle Temporal Gyrus, temporooccipital part | 2.80 | -60 | -48 | 0 |
| 8 |  |  |  |  |  |
|  | L Caudate | 3.09 | -18 | 22 | 6 |
| 8 |  |  |  |  |  |
|  | R Precuneus Cortex | 2.81 | 4 | -54 | 10 |
| 8 |  |  |  |  |  |
|  | L Lateral Occipital Cortex, superior division | 2.79 | -54 | -70 | 16 |
| 8 |  |  |  |  |  |
|  | R Central Opercular Cortex | 2.93 | 50 | -8 | 16 |
| 8 |  |  |  |  |  |
|  | R Angular Gyrus | 2.85 | 56 | -54 | 18 |
| 8 |  |  |  |  |  |
|  | R Angular Gyrus | 2.85 | 54 | -56 | 20 |
| 8 |  |  |  |  |  |
|  | L Angular Gyrus | 2.82 | -62 | -54 | 20 |
| 8 |  |  |  |  |  |
|  | L Central Opercular Cortex | 2.86 | -40 | -18 | 20 |
| 8 |  |  |  |  |  |
|  | R Superior Frontal Gyrus | 2.88 | 6 | 54 | 28 |
| 8 |  |  |  |  |  |
|  | L Middle Frontal Gyrus | 2.85 | -50 | 14 | 30 |
| 8 |  |  |  |  |  |
|  | R Precentral Gyrus | 2.76 | 48 | 4 | 32 |
| 8 |  |  |  |  |  |
|  | R Supramarginal Gyrus, anterior division | 2.75 | 66 | -26 | 38 |
| 8 |  |  |  |  |  |
|  | R Frontal Pole | 2.90 | 8 | 56 | 38 |
| 8 |  |  |  |  |  |
|  | R Frontal Pole | 2.85 | 10 | 58 | 40 |
| 8 |  |  |  |  |  |
|  | L Middle Frontal Gyrus | 2.78 | -30 | 22 | 46 |
| 8 |  |  |  |  |  |
|  | L Frontal Pole | 2.75 | -18 | 46 | 46 |
| 8 |  |  |  |  |  |
|  | R Juxtapositional Lobule Cortex (formerly Supplementary Motor Cortex) | 2.76 | 0 | -12 | 48 |
| 8 |  |  |  |  |  |
|  | R Middle Frontal Gyrus | 2.79 | 36 | 12 | 48 |
| 8 |  |  |  |  |  |
|  | L Lateral Occipital Cortex, superior division | 2.82 | -20 | -76 | 50 |
| 8 |  |  |  |  |  |
|  | L Superior Frontal Gyrus | 2.86 | -22 | -2 | 52 |
| 8 |  |  |  |  |  |
|  | R Superior Parietal Lobule | 3.10 | 34 | -54 | 56 |
| 8 |  |  |  |  |  |
|  | L Superior Parietal Lobule | 2.99 | -16 | -56 | 60 |
| 8 |  |  |  |  |  |
|  | R Postcentral Gyrus | 2.92 | 6 | -32 | 80 |
| 8 |  |  |  |  |  |
|  | L Precentral Gyrus | 2.81 | -10 | -28 | 80 |
| 8 |  |  |  |  |  |
|  | L Precentral Gyrus | 2.88 | -8 | -20 | 80 |

**SUPPLEMENTARY REFERENCES**

Anderson, J. S., Druzgal, T. J., Lopez-Larson, M., Jeong, E. K., Desai, K., & Yurgelun-Todd, D. (2011). Network anticorrelations, global regression, and phase-shifted soft tissue correction. *Hum Brain Mapp*, *32*(6), 919-934. [https://doi.org/10.1002/hbm.21079](https://doi.org/10.1002/hbm.21079) 

Avants, B. B., Tustison, N. J., Song, G., Cook, P. A., Klein, A., & Gee, J. C. (2011). A reproducible evaluation of ANTs similarity metric performance in brain image registration [Article]. *Neuroimage*, *54*, 2033-2044. [https://doi.org/10.1016/j.neuroimage.2010.09.025](https://doi.org/10.1016/j.neuroimage.2010.09.025) 

Bas-Hoogendam, J. M., Bernstein, R., Benson, B. E., Buss, K. A., Gunther, K. E., Pérez-Edgar, K., . . . Pine, D. S. (2022). Structural brain correlates of childhood inhibited temperament: An ENIGMA-anxiety mega-analysis [Registered Report]. *J Am Acad Child Adolesc Psychiatry*, *61*, 1182-1188. [https://doi.org/10.1016/j.jaac.2022.04.023](https://doi.org/10.1016/j.jaac.2022.04.023) 

BIAC. (2022). *IXI Dataset*. Imperial College London. Retrieved April 19 from [https://brain-development.org/ixi-dataset/](https://brain-development.org/ixi-dataset/)

Čeko, M., Kragel, P. A., Woo, C.-W., López-Solà, M., & Wager, T. D. (2022). Common and stimulus-type-specific brain representations of negative affect. *Nature Neuroscience*, *25*, 760-770. [https://doi.org/10.1038/s41593-022-01082-w](https://doi.org/10.1038/s41593-022-01082-w) 

Charpentier, C. J., Faulkner, P., Pool, E. R., Ly, V., Tollenaar, M. S., Kluen, L. M., . . . O’Doherty, J. P. (2021). How representative are neuroimaging samples? Large-scale evidence for trait anxiety differences between fMRI and behaviour-only research participants. *Social cognitive and affective neuroscience*, *16*, 1057-1070. [https://doi.org/10.1093/scan/nsab057](https://doi.org/10.1093/scan/nsab057) 

Cohen, J. (1988). *Statistical power analysis for the behavioral sciences* (2nd ed.). Lawrence Erlbaum Associates. 

Conway, C. C., Grogans, S. E., Anderson, A. S., Islam, S., Craig, L. E., Wedlock, J., . . . Shackman, A. J. (*in press*). Neuroticism is selectively associated with longitudinal increases in broadband internalizing symptoms, but not narrow-band positive affect or anxious arousal, in emerging adulthood. *Clinical Psychological Science [Preprint at PsyArXiv]*. [https://doi.org/https://doi.org/10.31234/osf.io/2awth](https://doi.org/https://doi.org/10.31234/osf.io/2awth) 

Cox, R. W. (1996). AFNI: Software for analysis and visualization of functional magnetic resonance neuroimages. *Computers and Biomedical Research*, *29*, 162-173. 

Daldrup, T., Remmes, J., Lesting, J., Gaburro, S., Fendt, M., Meuth, P., . . . Seidenbecher, T. (2015). Expression of freezing and fear-potentiated startle during sustained fear in mice. *Genes Brain Behav*, *14*, 281-291. [https://doi.org/10.1111/gbb.12211](https://doi.org/10.1111/gbb.12211) 

Desikan, R. S., Ségonne, F., Fischl, B., Quinn, B. T., Dickerson, B. C., Blacker, D., . . . Killiany, R. J. (2006). An automated labeling system for subdividing the human cerebral cortex on MRI scans into gyral based regions of interest. *Neuroimage*, *31*, 968-980. 

Edlow, B. L., Takahashi, E., Wu, O., Benner, T., Dai, G., Bu, L., . . . Folkerth, R. D. (2012). Neuroanatomic connectivity of the human ascending arousal system critical to consciousness and its disorders. *J Neuropathol Exp Neurol*, *71*(6), 531-546. [https://doi.org/10.1097/NEN.0b013e3182588293](https://doi.org/10.1097/NEN.0b013e3182588293) 

Erdfelder, E., Faul, F., & Buchner, A. (1996). GPOWER: A general power analysis program. *Behavior Research Methods, Instruments & Computers*, *28*, 1-11. [https://doi.org/10.3758/BF03203630](https://doi.org/10.3758/BF03203630) 

Eskildsen, S. F., Coupé, P., Fonov, V., Manjón, J. V., Leung, K. K., Guizard, N., . . . Alzheimer's Disease Neuroimaging Initiative. (2012). BEaST: brain extraction based on nonlocal segmentation technique. *Neuroimage*, *59*, 2362-2373. 

First, M. B., Williams, J. B. W., Karg, R. S., & Spitzer, R. L. (2015). Structured clinical interview for DSM-5—Research version (SCID-5 for DSM-5, research version; SCID-5-RV). *Arlington, VA: American Psychiatric Association*. 

Frazier, J. A., Chiu, S., Breeze, J. L., Makris, N., Lange, N., Kennedy, D. N., . . . Biederman, J. (2005). Structural brain magnetic resonance imaging of limbic and thalamic volumes in pediatric bipolar disorder. *American Journal of Psychiatry*, *162*, 1256-1265. 

Grabner, G., Janke, A. L., Budge, M. M., Smith, D., Pruessner, J., & Collins, D. L. (2006). Symmetric atlasing and model based segmentation: an application to the hippocampus in older adults. *Med Image Comput Comput Assist Interv Int Conf Med Image Comput Comput Assist Interv*, *9*, 58–66. 

Grillon, C., Ameli, R., Foot, M., & Davis, M. (1993). Fear-potentiated startle: relationship to the level of state/trait anxiety in healthy subjects. *Biol Psychiatry*, *33*, 566-574. 

Grillon, C., Ameli, R., Merikangas, K., Woods, S. W., & Davis, M. (1993). Measuring the time course of anticipatory anxiety using the fear-potentiated startle reflex. *Psychophysiology*, *30*, 340-346. 

Grogans, S. E., Bliss-Moreau, E., Buss, K. A., Clark, L. A., Fox, A. S., Keltner, D., . . . Shackman, A. J. (2023). The nature and neurobiology of fear and anxiety: State of the science and opportunities for accelerating discovery. *Neuroscience & Biobehavioral Reviews*, *151*, 105237. [https://doi.org/https://doi.org/10.1016/j.neubiorev.2023.105237](https://doi.org/https://doi.org/10.1016/j.neubiorev.2023.105237) 

Grogans, S. E., Hur, J., Barstead, M. G., Anderson, A. S., Islam, S., Kim, H. C., . . . Shackman, A. J. (2023). Neuroticism/negative emotionality is associated with increased reactivity to uncertain threat in the bed nucleus of the stria terminalis, not the amygdala. *bioRxiv*. [https://doi.org/https://doi.org/10.1101/2023.02.09.527767](https://doi.org/https://doi.org/10.1101/2023.02.09.527767) 

Grogans, S. E., Hur, J., Barstead, M. G., Anderson, A. S., Islam, S., Kuhn, M., . . . Shackman, A. J. (*in press*). Neuroticism/negative emotionality is associated with increased reactivity to uncertain threat in the bed nucleus of the stria terminalis, not the amygdala. *Journal of Neuroscience*. 

Hauner, K. K., Zinbarg, R. E., & Revelle, W. (2014). A latent variable model approach to estimating systematic bias in the oversampling method. *Behav Res*, *46*, 786-797. 

Hefner, K. R., Moberg, C. A., Hachiya, L. Y., & Curtin, J. J. (2013). Alcohol stress response dampening during imminent versus distal, uncertain threat. *J Abnorm Psychol*, *122*, 756-769. [https://doi.org/10.1037/a0033407](https://doi.org/10.1037/a0033407) 

Henson, R. (2007). Efficient experimental design for fMRI. In K. Friston, J. Ashburner, S. Kiebel, T. Nichols, & W. Penny (Eds.), *Statistical Parametric Mapping: The Analysis of Functional Brain Images* (pp. 193-210). Academic Press. 

Hur, J., DeYoung, K. A., Islam, S., Anderson, A. S., Barstead, M. G., & Shackman, A. J. (2020). Social context and the real-world consequences of social anxiety. *Psychological Medicine*, *50*, 1989-2000. 

Hur, J., Kuhn, M., Grogans, S. E., Anderson, A. S., Islam, S., Kim, H. C., . . . Shackman, A. J. (2022). Anxiety-related frontocortical activity is associated with dampened stressor reactivity in the real world. *Psychological Science*, *33*, 906-924. [https://doi.org/10.1101/2021.03.17.435791](https://doi.org/10.1101/2021.03.17.435791) 

Hur, J., Smith, J. F., DeYoung, K. A., Anderson, A. S., Kuang, J., Kim, H. C., . . . Shackman, A. J. (2020). Anxiety and the neurobiology of temporally uncertain threat anticipation. *Journal of Neuroscience*, *40*, 7949-7964. [https://doi.org/10.1101/2020.02.25.964734](https://doi.org/10.1101/2020.02.25.964734) 

Jenkinson, M., Beckmann, C. F., Behrens, T. E., Woolrich, M. W., & Smith, S. M. (2012). FSL. *Neuroimage*, *62*, 782-790. [https://doi.org/10.1016/j.neuroimage.2011.09.015](https://doi.org/10.1016/j.neuroimage.2011.09.015) 

Kassambara, A. (2023). *ggpubr: 'ggplot2' based publication ready plots*. In [https://rpkgs.datanovia.com/ggpubr/](https://rpkgs.datanovia.com/ggpubr/).

Kim, H. C., Kaplan, C. M., Islam, S., Anderson, A. S., Piper, M. E., Bradford, D. E., . . . Shackman, A. J. (2023). Acute nicotine abstinence amplifies subjective withdrawal symptoms and threat-evoked fear and anxiety, but not extended amygdala reactivity. *PLoS One*, *18*, e0288544. [https://doi.org/https://doi.org/10.1371/journal.pone.0288544](https://doi.org/https://doi.org/10.1371/journal.pone.0288544) 

Kruger, O., Shiozawa, T., Kreifelts, B., Scheffler, K., & Ethofer, T. (2015). Three distinct fiber pathways of the bed nucleus of the stria terminalis to the amygdala and prefrontal cortex. *Cortex*, *66*, 60-68. [https://doi.org/10.1016/j.cortex.2015.02.007](https://doi.org/10.1016/j.cortex.2015.02.007) 

Lange, M. D., Daldrup, T., Remmers, F., Szkudlarek, H. J., Lesting, J., Guggenhuber, S., . . . Pape, H. C. (2017). Cannabinoid CB1 receptors in distinct circuits of the extended amygdala determine fear responsiveness to unpredictable threat. *Mol Psychiatry*, *22*, 1422-1430. [https://doi.org/10.1038/mp.2016.156](https://doi.org/10.1038/mp.2016.156) 

Lemon, J. (2006). Plotrix: a package in the red light district of R. *R-News*, *6*, 8-12. 

Lorio, S., Fresard, S., Adaszewski, S., Kherif, F., Chowdhury, R., Frackowiak, R. S., . . . Draganski, B. (2016). New tissue priors for improved automated classification of subcortical brain structures on MRI. *Neuroimage*, *130*, 157-166. [https://doi.org/10.1016/j.neuroimage.2016.01.062](https://doi.org/10.1016/j.neuroimage.2016.01.062) 

Love, J., Selker, R., Marsman, M., Jamil, T., Dropmann, D., Verhagen, J., . . . Wagenmakers, E.-J. (2019). JASP: Graphical statistical software for common statistical designs. *Journal of Statistical Software*, *88*, 1-17. [https://doi.org/10.18637/jss.v088.i02](https://doi.org/10.18637/jss.v088.i02) 

Löw, A., Weymar, M., & Hamm, A. O. (2015). When threat is near, get out of here: Dynamics of defensive behavior during freezing and active avoidance. *Psychol Sci*, *26*, 1706-1716. [https://doi.org/10.1177/0956797615597332](https://doi.org/10.1177/0956797615597332) 

Mai, J. K., Majtanik, M., & Paxinos, G. (2015). *Atlas of the human brain* (4th ed.). Academic Press. 

Makris, N., Goldstein, J. M., Kennedy, D., Hodge, S. M., Caviness, V. S., Faraone, S. V., . . . Seidman, L. J. (2006). Decreased volume of left and total anterior insular lobule in schizophrenia. *Schizophrenia Research*, *83*, 155-171. 

McCormick, M., Liu, X., Jomier, J., Marion, C., & Ibanez, L. (2014). ITK: enabling reproducible research and open science. *Frontiers in Neuroinformatics*, *8*, 13. [https://doi.org/10.3389/fninf.2014.00013](https://doi.org/10.3389/fninf.2014.00013) 

Menon, V. (2015). Salience network. In A. W. Toga (Ed.), *Brain Mapping* (Vol. 2, pp. 597-611). Academic Press. [https://doi.org/https://doi.org/10.1016/B978-0-12-397025-1.00052-X](https://doi.org/https://doi.org/10.1016/B978-0-12-397025-1.00052-X) 

Miles, L., Davis, M., & Walker, D. (2011). Phasic and sustained fear are pharmacologically dissociable in rats. *Neuropsychopharmacology*, *36*, 1563-1574. [https://doi.org/10.1038/npp.2011.29](https://doi.org/10.1038/npp.2011.29) 

Moberg, C. A., Bradford, D. E., Kaye, J. T., & Curtin, J. J. (2017). Increased startle potentiation to unpredictable stressors in alcohol dependence: Possible stress neuroadaptation in humans. *Journal of Abnormal Psychology*, *126*, 441-453. [https://doi.org/10.1037/abn0000265](https://doi.org/10.1037/abn0000265) 

Mumford, J. A., Poline, J. B., & Poldrack, R. A. (2015). Orthogonalization of regressors in FMRI models. *PLoS One*, *10*, e0126255. [https://doi.org/10.1371/journal.pone.0126255](https://doi.org/10.1371/journal.pone.0126255) 

Nichols, T., Brett, M., Andersson, J., Wager, T., & Poline, J. B. (2005). Valid conjunction inference with the minimum statistic. *Neuroimage*, *25*, 653-660. [https://doi.org/S1053-8119(04)00750-5](https://doi.org/S1053-8119(04)00750-5) [pii]

10.1016/j.neuroimage.2004.12.005 [doi] 

Poldrack, R. A. (2011). Inferring mental states from neuroimaging data: from reverse inference to large-scale decoding. *Neuron*, *72*, 692-697. [https://doi.org/10.1016/j.neuron.2011.11.001](https://doi.org/10.1016/j.neuron.2011.11.001) 

Poldrack, R. A., Baker, C. I., Durnez, J., Gorgolewski, K. J., Matthews, P. M., Munafò, M. R., . . . Yarkoni, T. (2017). Scanning the horizon: towards transparent and reproducible neuroimaging research. *Nature Reviews Neuroscience*, *18*, 115-126. 

Poline, J.-B., Kherif, F., & Penny, W. (2007). Contrasts and Classical Inference. *Statistical Parametric Mapping: The Analysis of Functional Brain Images*. [https://doi.org/10.1016/B978-012372560-8/50009-7](https://doi.org/10.1016/B978-012372560-8/50009-7) 

Pruim, R. H. R., Mennes, M., van Rooij, D., Llera, A., Buitelaar, J. K., & Beckmann, C. F. (2015). ICA-AROMA: a robust ICA-based strategy for removing motion artifacts from fMRI data. *Neuroimage*, *112*, 267-277. 

R Core Team. (2022). R: A language and environment for statistical computing. In. Vienna, Austria: R Foundation for Statistical Computing.

Rorden, C. (2019, September 2, 2019). *MRIcron*. NITRC. Retrieved April 18 from [https://www.nitrc.org/projects/mricron](https://www.nitrc.org/projects/mricron)

RStudio Team. (2022). RStudio: Integrated Development for R. In. Boston, MA: RStudio PBC.

Schimmack, U. (2019, December 18, 2019). *Statistics wars: Don’t change alpha. Change the null-hypothesis!* Retrieved June 1 from [https://replicationindex.com/category/nil-hypothesis/](https://replicationindex.com/category/nil-hypothesis/)

Shackman, A. J., & Fox, A. S. (2016). Contributions of the central extended amygdala to fear and anxiety. *Journal of Neuroscience*, *36*, 8050-8063. [https://doi.org/10.1523/JNEUROSCI.0982-16.2016](https://doi.org/10.1523/JNEUROSCI.0982-16.2016) 

Shackman, A. J., & Fox, A. S. (2021). Two decades of anxiety neuroimaging research: New insights and a look to the future. *American Journal of Psychiatry*, *178*, 106-109. 

Shackman, A. J., Grogans, S. E., & Fox, A. S. (*in press*). Fear, anxiety, and the functional architecture of the human central extended amygdala. *Nature Reviews Neuroscience*. [https://doi.org/https://doi.org/10.1038/s41583-024-00832-y](https://doi.org/https://doi.org/10.1038/s41583-024-00832-y) 

Sokolov, E. N., Spinks, J. A., Näätänen, R., & Lyytinen, H. (2002). *The orienting response in information processing*. Lawrence Erlbaum Associates. 

Theiss, J. D., Ridgewell, C., McHugo, M., Heckers, S., & Blackford, J. U. (2017). Manual segmentation of the human bed nucleus of the stria terminalis using 3T MRI. *Neuroimage*, *146*, 288-292. [https://doi.org/10.1016/j.neuroimage.2016.11.047](https://doi.org/10.1016/j.neuroimage.2016.11.047) 

Tillman, R. M., Stockbridge, M. D., Nacewicz, B. M., Torrisi, S., Fox, A. S., Smith, J. F., & Shackman, A. J. (2018). Intrinsic functional connectivity of the central extended amygdala. *Human Brain Mapping*, *39*, 1291-1312. 

Tukey, J. W. (1977). *Exploratory data analysis*. Addison Wesley. 

Tustison, N. J., Avants, B. B., Cook, P. A., Zheng, Y. J., Egan, A., Yushkevich, P. A., & Gee, J. C. (2010). N4ITK: Improved N3 bias correction [Article]. *IEEE Transactions on Medical Imaging*, *29*, 1310-1320. [https://doi.org/10.1109/tmi.2010.2046908](https://doi.org/10.1109/tmi.2010.2046908) 

van Doorn, J., van den Bergh, D., Böhm, U., Dablander, F., Derks, K., Draws, T., . . . Wagenmakers, E.-J. (2021). The JASP guidelines for conducting and reporting a Bayesian analysis. *Psychonomic Bulletin & Review*, *28*, 813-826. [https://doi.org/10.3758/s13423-020-01798-5](https://doi.org/10.3758/s13423-020-01798-5) 

Wang, L., Tseng, Y.-T., Schaefke, B., Wei, P., & He, S. (*in press*). Reply to ‘Fear, anxiety and the functional architecture of the human central extended amygdala’. *Nature Reviews Neuroscience*. [https://doi.org/10.1038/s41583-024-00834-w](https://doi.org/10.1038/s41583-024-00834-w) 

Wellcome Centre for Human Neuroimaging. (2022). *SPM*. University College London. Retrieved April 18 from [https://fil.ion.ucl.ac.uk/spm/](https://fil.ion.ucl.ac.uk/spm/)

Wickham, H. (2016). *ggplot2: Elegant graphics for data analysis* (2nd ed.). Springer-Verlag. 

Wickham, H., Averick, M., Bryan, J., Chang, W., McGowan, L. D., François, R., . . . Yutani, H. (2019). Welcome to the tidyverse. *Journal of Open Source Software*, *4*, 1686. [https://doi.org/doi:10.21105/joss.01686](https://doi.org/doi:10.21105/joss.01686).
